# Supplementary material for: Dynamically chiral phosphonic acid-type metallo-β-lactamase inhibitors
Source: Commun Chem. 2025 Apr 19;8:119. doi: 10.1038/s42004-025-01510-5 (PMC12009420; doi:10.1038/s42004-025-01510-5)
Supplement: Supplementary file 2 — Supplemental Material [file 42004_2025_1510_MOESM2_ESM.pdf]

Dynamically chiral phosphonic acid-type metallo-β-lactamase inhibitors

Kinga Virág Gulyás, Liping Zhou, Daniel Salamonsen, Andreas Prester, Kim Bartels, Robert Bosman, Paul Haffke, Jintian Li, Viola Tamási, Fritz Deufel, Johannes Thoma, Anna Andersson Rasmussen, Miklós Csala, Hanna-Kirsti Schroder Leiros, Zhijian Xu, Mikael Widersten, Holger Rohde, Eike C. Schulz, Weiliang Zhu and Máté Erdélyi

Table of Contents

1. Metallo-β-lactamase numbering 2
2. Synthetic procedures 3
3. Analytical purity of the final compounds 20
4. Long term stability of the final compounds in buffer with 2.5% DMSO 27
5. Chiral HPLC 37
6. Metallo-β-lactamase inhibition (IC_{50}, K_i) on purified enzymes 38
7. Enzyme kinetic studies 46
8. Cytotoxocity against HepG2 cells 47
9. Inhibitor binding comparison to previous phosphorous-containing metallo-β-lactamase inhibitors 51
10. Membrane permeability on *in situ* and on permeabilized membrane 54
11. Prediction of ADME properties 55
12. ^{1}H, ^{15}N-HSQC titration experiments and chemical shift perturbations 63
13. X-ray diffraction of VIM-2 inhibitor complexes 94
14. Computational simulations 114
15. Protein expression and purification 127
16. NMR spectra 129
17. References 187

The original FIDs for the synthesized compounds, the crystallographic cif files and the computed structures of the enzyme-inhibitor complexes (pdb) are freely available on Zenodo (DOI:10.5281/zenodo.12571911).

1. Metallo-β-lactamase numbering

Table S1. *Metallo-β-lactamase numbering used in this paper and the corresponding consensus BBL numbering.*

**VIM-2**<br>(PDB ID: 9F0Q, 9F0P, 9F0S, 9F0R)	**NDM-1**<br>(PDB ID: 6D1A)	**GIM-1**<br>(PDB ID: 2YNT)	**Consensus BBL numbering**[1]
61S	66D	60N	60
62F	67M	61I	61
63D	68P	62E	62
67Y	73V	67V	67
69S	75S	69S	69
70N	76N	70N	70
85T	91T	85T	85
87W	93W	87W	87
114H	120H	116H	116
116H	122H	118H	118
117D	123Q	119E	119
118D	124D	120D	120
146E	152E	149E	149
178A	188G	195G	195
179H	189H	196H	196
180S	190T	197T	197
181T	191S	198E	198
198C	208C	221C	221
201Y	211K	224R	224
202E	213D	225S	225
205R	215A	228W	228
209G	219G	232G	232
210N	220N	233Y	233
213D	223D	236D	236
240H	250H	263H	263

2. Synthetic procedures

A general description of the synthetic procedures is given under Methods in the main text. Information on specific reactions is given here below.

2-Methoxybenzaldehyde (1)[2]

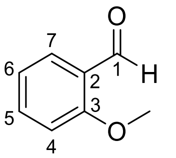

The synthetic procedure was adapted from Chen et al.[3] Sodium hydride (60% dispersion in mineral oil, 570 mg, 14.3 mmol, 1.0 equiv.) was dissolved in a mixture of DMF (16 mL) and THF (4 mL). 2-Hydroxybenzaldehyde (1.5 mL, 14.1 mmol) followed by iodomethane (2.65 ml, 42.6 mmol, 3.0 equiv.) in THF (10 mL) were added dropwise to the NaH solution at 0°C. The reaction was stirred at 0°C for 2 h and subsequently allowed to warm to r.t. and further stirred overnight. The reaction mixture was poured into ice (50 mL) and extracted with Et_2O (3x 50 mL). The combined organic layers were washed with brine and dried over Na_2SO_4 and the solvent was removed under reduced pressure. The crude product was purified with flash column chromatography (SiO_2, gradient EtOAc in hexane, 0% to 20%). Compound **1** was obtained as a colorless oil (1.80 g, 13.2 mmol, 94%). MS (*m/z*): 137.3 $[M+H]^+$ (137.1 calcd. for C_8H_8O).

^{1}H NMR (400 MHz, $CDCl_3$) δ 10.46 (d, *J* = 1.0 Hz, 1H, O=CH), 7.81 (dd, *J* = 7.7, 1.8 Hz, 1H, 4-H), 7.54 (ddd, *J* = 8.4, 7.3, 1.8 Hz, 1H, 6-H), 7.01 (ddd, *J* = 7.7, 7.3, 1.0 Hz, 1H, 5-H), 6.98 (ddd, *J* = 8.4, 1.0, 1.0 Hz, 1H, 7-H), 3.91 (s, 3H, CH_3). ^{13}C NMR (101 MHz, $CDCl_3$) δ 189.9 (O=CH), 161.9 (3-C), 136.1 (6-CH), 128.6 (4-CH), 124.9 (2-C), 120.8 (5-CH), 111.7 (7-CH), 55.7 (CH_3).

Diethyl ((benzylamino)(2-methoxyphenyl)methyl)phosphonate (2)

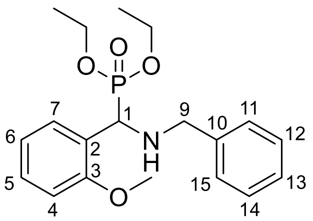

The synthetic procedure was adapted from that described by Palica et al.[4] Compound **1** (1.75 g, 12.8 mmol), benzylamine (1.45 mL, 13.3 mmol, 1.0 equiv.) and diethyl phosphite (1.70 mL, 13.2 mmol, 1.0 equiv.) were dissolved in absolute EtOH (13 mL). $HfCl_4$ (82 mg, 0.3 mmol, 2 mol%) was added in one portion and the reaction was stirred at 60°C for 18h. The solvent was removed under reduced pressure and the crude product was purified by flash column chromatography (SiO_2, gradient EtOAc in hexane, 20% to 100%). Compound **2** was obtained as a white solid (3.40 g, 9.4 mmol, 73%). HRESIMS (*m/z*): 364.1682 $[M+H]^+$ (364.1599 calcd. for $C_{19}H_{26}NO_4P$). ^{1}H NMR (500 MHz, CD_3OD) δ 7.51 (dd, *J* = 7.8, 1.9 Hz, 1H, 7-H), 7.33 (ddd, *J* = 8.3, 7.4, 1.9 Hz, 1H, 5-H), 7.31 – 7.27 (m, 2H, 11-H and 15-H), 7.28 – 7.20 (m, 3H, 12-H, 13-H and 14-H), 7.02 (ddd, *J* = 7.8, 7.4, 1.2 Hz, 1H, 6-H), 7.01 (dd, *J* = 8.3, 1.2 Hz, 1H, 4-H), 4.61 (d, $^2J_{HP}$ = 21.8 Hz, 1H, 1-H), 4.14 (dq, *J* = 10.1, 7.1 Hz, 1H, OCH_2), 4.08 (dq, *J* =

10.1, 7.1 Hz, 1H, OCH_2), 3.91 (dq, J = 10.2, 7.1 Hz, 1H, OCH_2), 3.84 – 3.72 (m, 5H, OCH_2, 9-H, and OCH_3), 3.52 (d, J = 13.1 Hz, 1H, 9-H), 1.29 (t, J = 7.1 Hz, 3H, CH_3), 1.08 (t, J = 7.1 Hz, 3H, CH_3). ^{13}C NMR (126 MHz, CD_3OD) δ 159.3 (d, $^3J_{CP}$ = 6.7 Hz, 3-C), 140.0 (10-C), 130.5 (d, $^5J_{CP}$ = 3.0 Hz, 5-CH), 130.0 (d, $^3J_{CP}$ = 5.1 Hz, 7-CH), 129.8 (11-CH and 15-CH), 129.3 (12-CH and 14-CH), 128.3 (13-CH), 124.5 (2-C), 121.8 (d, $^4J_{CP}$ = 2.8 Hz, 6-CH), 112.0 (d, $^4J_{CP}$ = 2.1 Hz, 4-CH), 64.4 (d, $^2J_{CP}$ = 7.1 Hz, OCH_2), 64.2 (d, $^2J_{CP}$ = 7.2 Hz, OCH_2), 56.0 (OCH_3), 52.10 (d, $^1J_{CP}$ = 158.5 Hz, 1-CH) 52.0 (d, $^3J_{CP}$ = 16.8 Hz, 9-CH_2), 17.0 (d, $^3J_{CP}$ = 5.9 Hz, CH_3), 16.5 (d, $^3J_{CP}$ = 5.7 Hz, CH_3). ^{31}P NMR (162 MHz, CD_3OD) δ 24.6.

Diethyl (amino(2-methoxyphenyl)methyl)phosphonate (3)

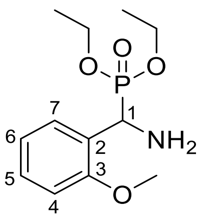

The synthetic procedure was adapted from that described by Palica et al.[4] Compound **2** (2.74 g, 7.5 mmol) and $Pd(OH)_2$/C, (20% w/w, nominally 50% water, 1.46 g, 1.0 mmol, 0.14 equiv.) were dissolved in EtOH (250 mL). A strong argon flow was bubbled through the reaction mixture for 5 min followed by H_2 for 30 s and the reaction vial was equipped with an H_2 filled balloon. The reaction was stirred at r.t. for 24h, filtered through a syringe filter and the solvent was removed under reduced pressure. The product was obtained as a colorless liquid (1.98 g, 0.428 mmol, 92%). HRESIMS (*m/z*): 274.1219 $[M+H]^+$ (274.1178 calcd. for $C_{12}H_2NO_4P$). ^{1}H NMR (400 MHz, CD_3OD) δ 7.46 (dd, J = 7.6, 1.9 Hz, 1H, 7-H), 7.30 (ddd, J = 8.7, 7.6, 1.9 Hz, 1H, 5-H), 7.00 (d, J = 8.7 Hz, 1H, 4-H), 6.98 (dd, J = 7.6, 7.6 Hz, 1H, 6-H), 4.73 (d, $^2J_{HP}$ = 18.0 Hz, 1H, 1-H), 4.10 (q, J = 7.2 Hz, 2H, OCH_2), 3.94 (dq, J = 9.9, 7.1 Hz, 1H, OCH_2), 3.86 (s, 3H, OCH_3), 3.84 (m, 1H, OCH_2), 1.31 (t, J = 7.2 Hz, 3H, CH_3), 1.15 (t, J = 7.1 Hz, 3H, CH_3).^{13}C NMR (126 MHz, CD_3OD) δ 158.3 (d, $^3J_{CP}$ = 6.8 Hz, 3-C), 130.3 (d, $^5J_{CP}$ = 3.2 Hz, 5-CH), 129.6 (d, $^3J_{CP}$ = 5.0 Hz, 7-CH), 126.8 (2-C), 121.7 (d, $^4J_{CP}$ = 2.8 Hz, 6-CH), 111.8 (d, $^4J_{CP}$ = 2.2 Hz, 4-CH), 64.2 (d, $^2J_{CP}$ = 7.3 Hz, OCH_2), 64.0 (d, $^2J_{CP}$ = 7.4 Hz, OCH_2), 56.1 (OCH_3), 47.1 (d, $^1J_{CP}$ = 150.1 Hz, 1-CH), 16.7 (d, $^3J_{CP}$ = 5.6 Hz, CH_3), 16.6 (d, $^3J_{CP}$ = 5.7 Hz, CH_3). ^{31}P NMR (162 MHz, CD_3OD) δ 26.0.

General procedure A for 4a-m.

The synthetic procedure was adapted from that described by Palica et al.[4] The carboxylic acid (2.0 equiv.) and HATU or COMU (2.1 equiv.) were dissolved in ethyl acetate. DIPEA (4.0 equiv.) was added dropwise, and the reaction mixture was stirred at r.t. for 40 min. The reaction mixture was added dropwise to a solution of compound **3** in ethyl acetate. The reaction was stirred at r.t. for 18 h, then diluted with EtOAc and washed three times with a sat. solution of $NaHCO_3$. The aqueous layer was re-extracted with EtOAc twice and the combined organic layers were washed with brine and dried over Na_2SO_4. The solvent was removed under reduced pressure and the crude product was purified by flash column chromatography (SiO_2, gradient EtOAc in hexane, 20% to 100%). The products **4a-m** were obtained in a yield of 61-95%.

Diethyl ((2-methoxyphenyl)(thiophene-2-carboxamido)methyl)phosphonate (4a)

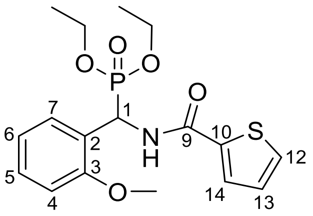

The synthesis was performed according to general procedure A, compound **3** (100 mg, 0.37 mmol) and thiophene-2-carboxylic acid (95 mg, 0.74 mmol, 2.0 equiv.) gave the product **4a** as a white solid (128 mg, 0.33 mmol, 91%). HRESIMS (*m/z*): 384.1057 $[M+H]^+$ (384.1034 calcd. for $C_{17}H_{22}NO_5PS$). ^{1}H NMR (500 MHz, CD_3OD) δ 7.84 (dd, *J* = 3.8, 1.2 Hz, 1H, 14-H), 7.69 (dd, *J* = 5.0, 1.2 Hz, 1H, 12-H), 7.64 (dd, *J* = 7.6, 1.9 Hz, 1H, 7-H), 7.34 (ddd, *J* = 8.8, 7.6, 1.9 Hz, 1H, 5-H), 7.15 (dd, *J* = 5.0, 3.8 Hz, 1H, 13-H), 7.04 (dt, *J* = 8.8, 1.1 Hz, 1H, 4-H), 7.00 (ddd, *J* = 7.6, 7.6, 1.1 Hz, 1H, 6-H), 6.29 (d, $^2J_{HP}$ = 21.5 Hz, 1H, 1-H), 4.15 (dqd, *J* = 8.3, 7.1, 1.2 ($^3J_{HP}$) Hz, 2H, OCH_2), 3.99 (dq, *J* = 10.1, 7.1 Hz, 1H, OCH_2), 3.91 (s, 3H, OCH_3), 3.88 (dq, *J* = 10.1, 7.1 Hz, 1H, OCH_2), 1.30 (t, *J* = 7.1 Hz, 3H, CH_3), 1.14 (t, *J* = 7.1 Hz, 3H, CH_3). ^{13}C NMR (126 MHz, CD_3OD) δ 163.5 (d, $^3J_{CP}$ = 7.0 Hz, 9-C=O), 158.5 (d, $^3J_{CP}$ = 6.7 Hz, 3-C), 139.4 (10-C), 132.4 (12-CH), 130.9 (d, $^5J_{CP}$ = 2.6 Hz, 5-CH), 130.7 (d, $^3J_{CP}$ = 4.7 Hz, 7-CH), 130.3 (14-CH), 128.9 (13-CH), 124.1 (2-C), 121.8 (d, $^4J_{CP}$ = 2.4 Hz, 6-CH), 112.2 (d, $^4J_{CP}$ = 1.4 Hz, 4-CH), 64.7 (d, $^2J_{CP}$ = 7.2 Hz, OCH_2), 64.6 (d, $^2J_{CP}$ = 7.2 Hz, OCH_2), 56.3 (OCH_3), 45.4 (d, $^1J_{CP}$ = 159.4 Hz, 1-CH), 16.8 (d, $^3J_{CP}$ = 5.6 Hz, CH_3), 16.5 (d, $^3J_{CP}$ = 5.8 Hz, CH_3). ^{31}P NMR (162 MHz, CD_3OD) δ 22.2.

Diethyl ((2-methoxyphenyl)(thiophene-3-carboxamido)methyl)phosphonate (4b)

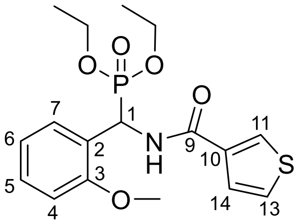

The synthesis was performed according to general procedure A, compound **3** (100 mg, 0.37 mmol) and thiophene-3-carboxylic acid (95 mg, 0.74 mmol, 2.0 equiv.) gave the product as a white solid (130 mg, 0.34 mmol, 94%). HRESIMS (*m/z*): 384.1035 $[M+H]^+$ (384.1034 calcd. for $C_{17}H_{22}NO_5PS$). ^{1}H NMR (500 MHz, CD_3OD) δ 8.16 (dd, *J* = 2.9, 1.3 Hz, 1H, 11-H), 7.63 (dd, *J* = 7.6, 1.9 Hz, 1H, 7-H), 7.54 (dd, *J* = 5.1, 1.3 Hz, 1H, 14-H), 7.49 (dd, *J* = 5.1, 2.9 Hz, 1H, 13-H), 7.34 (ddd, *J* = 8.7, 7.6, 1.9 Hz, 1H, 5-H), 7.04 (d, *J* = 8.7 Hz, 1H, 4-H), 6.99 (dd, *J* = 7.6, 7.6 Hz, 1H, 6-H), 6.32 (d, $^2J_{HP}$ = 21.5 Hz, 1H, 1-H), 4.15 (dqd, *J* = 8.5, 7.1, 1.3 ($^3J_{HP}$) Hz, 2H, OCH_2), 3.99 (dq, *J* = 10.1, 7.1 Hz, 1H, OCH_2), 3.92 (s, 3H, OCH_3), 3.87 (dq, *J* = 10.1, 7.1 Hz, 1H, OCH_2), 1.30 (t, *J* = 7.1 Hz, 3H, CH_3), 1.14 (t, *J* = 7.1 Hz, 3H, CH_3). ^{13}C NMR (126 MHz, CD_3OD) δ 164.7 (d, $^3J_{CP}$ = 6.8 Hz, 9-C=O), 158.5 (d, $^3J_{CP}$ = 6.7 Hz, 3-C), 137.7 (10-C), 130.9 (d, $^5J_{CP}$ = 2.8 Hz, 5-CH), 130.6 (d, $^3J_{CP}$ = 4.8 Hz, 7-CH), 130.5 (11-CH), 127.8 (14-CH), 127.6 (13-CH), 124.2 (2-C), 121.8 (d, $^4J_{CP}$ = 2.5 Hz, 6-CH), 112.2 (d, $^4J_{CP}$ = 1.7 Hz, 4-CH), 64.7 (d, $^2J_{CP}$ = 7.2 Hz, OCH_2), 64.6 (d, $^2J_{CP}$ = 7.4 Hz, OCH_2), 56.3 (OCH_3), 45.2 (d, $^1J_{CP}$ =

159.1 Hz, 1-CH), 16.8 (d, $^{3}J_{CP}$ = 5.6 Hz, CH_3), 16.5 (d, $^{3}J_{CP}$ = 5.9 Hz, CH_3). ^{31}P NMR (162 MHz, CD_3OD) δ 22.4.

Diethyl ((benzo[*b*]thiophene-2-carboxamido)(2-methoxyphenyl)methyl)phosphonate (4c)

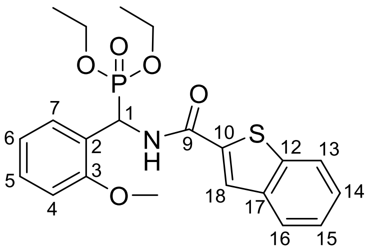

The synthesis was performed according to general procedure A, compound **3** (100 mg, 0.37 mmol) and benzo*[b]*thiophene-2-carboxylic acid (130 mg, 0.74 mmol, 2.0 equiv.) gave the product as a white solid (122 mg, 0.28 mmol, 77%). HRESIMS (*m/z*): 434.1187 $[M+H]^+$ (434.1191 calcd. for $C_{21}H_{24}NO_5PS$). ^{1}H NMR (400 MHz, CD_3OD) δ 8.11 (s, 1H, 18-H), 7.95 – 7.87 (m, 2H, 13-H and 16-H), 7.68 (dd, *J* = 7.6, 1.9 Hz, 1H, 7-H), 7.49 – 7.40 (m, 2H, 14-H and 15-H), 7.35 (ddd, *J* = 8.7, 7.6, 1.9 Hz, 1H, 5-H), 7.06 (d, *J* = 8.7 Hz, 1H, 4-H), 7.02 (dd, *J* = 7.6, 7.6 Hz, 1H, 6-H), 6.33 (d, $^{2}J_{HP}$ = 21.4 Hz, 1H, 1-H), 4.18 (dqd, *J* = 8.5, 7.1, 1.2 ($^{3}J_{HP}$) Hz, 2H, OCH_2), 4.01 (dq, *J* = 10.1, 7.1 Hz, 1H, OCH_2), 3.93 (s, 3H, OCH_3), 3.90 (dq, *J* = 10.1, 7.1 Hz, 1H, OCH_2), 1.31 (t, *J* = 7.1 Hz, 3H, CH_3), 1.15 (t, *J* = 7.1 Hz, 3H, CH_3). ^{13}C NMR (126 MHz, CD_3OD) δ 163.9 (d, $^{3}J_{CP}$ = 6.8 Hz, 9-C=O), 158.5 (d, $^{3}J_{CP}$ = 6.8 Hz, 3-C), 142.6 (12-C or 17-C), 140.7 (12-C or 17-C), 139.2 (10-C), 131.0 (d, $^{5}J_{CP}$ = 2.8 Hz, 5-CH), 130.7 (d, $^{3}J_{CP}$ = 4.8 Hz, 7-CH), 127.7 (14-CH or 15-CH), 127.3 (18-CH), 126.4 (13-CH or 16-CH), 126.1 (14-CH or 15-CH), 124.0 (2-C), 123.6 (13-CH or 16-CH), 121.8 (d, $^{4}J_{CP}$ = 2.5 Hz, 6-CH), 112.2 (d, $^{4}J_{CP}$ = 1.8 Hz, 4-CH), 64.7 (d, $^{2}J_{CP}$ = 7.2 Hz, OCH_2), 64.7 (d, $^{2}J_{CP}$ = 7.3 Hz, OCH_2), 56.4 (OCH_3), 45.6 (d, $^{1}J_{CP}$ = 159.4 Hz, 1-CH), 16.8 (d, $^{3}J_{CP}$ = 5.6 Hz, CH_3), 16.5 (d, $^{3}J_{CP}$ = 5.9 Hz, CH_3). ^{31}P NMR (162 MHz, CD_3OD) δ 22.0.

Diethyl ((benzo[*b*]thiophene-3-carboxamido)(2-methoxyphenyl)methyl)phosphonate (4d)

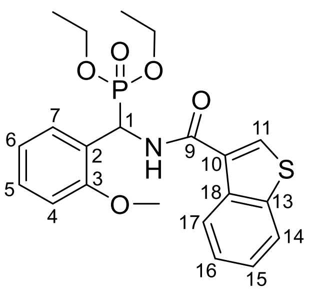

The synthesis was performed according to general procedure A, compound **3** (200 mg, 0.73 mmol) and benzo*[b]*thiophene-3-carboxylic acid (265 mg, 1.5 mmol, 2.0 equiv.) gave the product as a white solid (290 mg, 0.67 mmol, 91%). HRESIMS (*m/z*): 434.1182 $[M+H]^+$ (434.1191 calcd. for $C_{21}H_{24}NO_5PS$). ^{1}H NMR (400 MHz, CD_3OD) δ 8.30 (dd, *J* = 7.0, 2.1 Hz,

1H, 17-H), 8.23 (s, 1H, 11-H), 7.94 (dd, J = 6.8, 2.0 Hz, 1H, 14-H), 7.64 (dd, J = 7.6, 1.9 Hz, 1H, 7-H), 7.47 – 7.38 (m, 2H, 15-H and 16-H), 7.35 (ddd, J = 8.6, 7.5, 1.9 Hz, 1H, 5-H), 7.06 (d, J = 8.6 Hz, 1H, 4-H), 7.01 (dd, J = 7.6, 7.5 Hz, 1H, 6-H), 6.40 (d, $^2J_{HP}$ = 21.4 Hz, 1H, 1-H), 4.18 (dqd, J = 8.3, 7.2, 1.2 ($^3J_{HP}$) Hz, 2H, OCH_2), 4.03 (dq, J = 10.1, 7.1 Hz, 1H, OCH_2), 3.94 (s, 3H, OCH_3), 3.92 (dq, J = 10.1, 7.1 Hz, 1H, OCH_2), 1.32 (t, J = 7.2 Hz, 3H, CH_3), 1.17 (t, J = 7.1 Hz, 3H, CH_3). ^{13}C NMR (126 MHz, CD_3OD) δ 165.6 (d, $^3J_{CP}$ = 6.4 Hz, 9-C=O), 158.5 (d, $^3J_{CP}$ = 6.6 Hz, 3-C), 141.5 (13-C), 138.4 (18-C), 132.0 (11-CH), 131.9 (10-C), 130.9 (d, $^5J_{CP}$ = 2.8 Hz, 5-CH), 130.6 (d, $^3J_{CP}$ = 4.7 Hz, 7-CH), 126.2 (15-CH or 16-CH), 126.0 (15-CH or 16-CH), 125.1 (17-CH), 124.2 (2-C), 123.6 (14-CH), 121.8 (d, $^4J_{CP}$ = 2.5 Hz, 6-CH), 112.2 (d, $^4J_{CP}$ = 1.9 Hz, 4-CH), 64.7 (d, $^2J_{CP}$ = 6.8 Hz, OCH_2), 64.6 (d, $^2J_{CP}$ = 7.6 Hz, OCH_2), 56.3 (OCH_3), 45.1 (d, $^1J_{CP}$ = 158.7 Hz, 1-CH), 16.8 (d, $^3J_{CP}$ = 5.8 Hz, CH_3), 16.6 (d, $^3J_{CP}$ = 5.9 Hz, CH_3). ^{31}P NMR (162 MHz, CD_3OD) δ 22.3.

Diethyl ((2-methoxyphenyl)(4,5,6,7-tetrahydrobenzo[*b*]thiophene-2-carboxamido)methyl)phosphonate (4e)

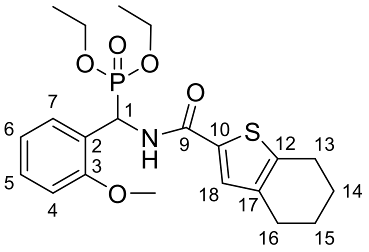

The synthesis was performed according to general procedure A, compound **3** (46 mg, 0.17 mmol) and 4,5,6,7-tetrahydrobenzo*[b]*thiophene-2-carboxylic acid (62 mg, 0.74 mmol, 2.0 equiv.) gave the product as a white solid (70 mg, 0.28 mmol, 95%). HRESIMS (*m/z*): 438.1497 $[M+H]^+$ (438.1504 calcd. for $C_{21}H_{28}NO_5PS$). ^{1}H NMR (500 MHz, CD_3OD) δ 7.61 (dd, J = 7.6, 1.9 Hz, 1H, 7-H), 7.49 (s, 1H, 18-H), 7.33 (ddd, J = 8.7, 7.6, 1.9 Hz, 1H, 5-H), 7.03 (d, J = 8.7 Hz, 1H, 4-H), 6.99 (dd, J = 7.6, 7.6 Hz, 1H, 6-H), 6.25 (d, $^2J_{HP}$ = 21.6 Hz, 1H, 1-H), 4.14 (dqd, J = 8.3, 7.1, 1.3 ($^3J_{HP}$) Hz, 2H, OCH_2), 3.98 (dq, J = 10.2, 7.1 Hz, 1H, OCH_2), 3.91 (s, 3H, OCH_3), 3.86 (dq, J = 10.2, 7.1 Hz, 1H, OCH_2), 2.78 (t, J = 5.8 Hz, 2H, 13-H), 2.64 (t, J = 5.9 Hz, 2H, 16-H), 1.90 – 1.77 (m, 4H, 14-H and 15-H), 1.29 (t, J = 7.1 Hz, 3H, CH_3), 1.13 (t, J = 7.1 Hz, 3H, CH_3). ^{13}C NMR (126 MHz, CD_3OD) δ 163.8 (d, $^3J_{CP}$ = 6.9 Hz, 9-C=O), 158.5 (d, $^3J_{CP}$ = 6.5 Hz, 3-C), 143.8 (12-C), 137.7 (17-C), 134.9 (10-C), 131.2 (18-CH), 130.9 (d, $^5J_{CP}$ = 2.8 Hz, 5-CH), 130.6 (d, $^3J_{CP}$ = 4.8 Hz, 7-CH), 124.2 (2-C), 121.8 (d, $^4J_{CP}$ = 2.5 Hz, 6-CH), 112.2 (d, $^3J_{CP}$ = 1.7 Hz, 4-CH), 64.7 (d, $^2J_{CP}$ = 5.9 Hz, OCH_2), 64.6 (d, $^2J_{CP}$ = 6.3 Hz, OCH_2), 56.3 (OCH_3), 45.5 (d, $^1J_{CP}$ = 159.0 Hz, 1-CH), 26.4 (16-CH_2), 26.1 (13-CH_2), 24.4 (14-CH_2 or 15-CH_2), 23.8 (14-CH_2 or 15-CH_2), 16.8 (d, $^3J_{CP}$ = 5.7 Hz, CH_3), 16.5 (d, $^3J_{CP}$ = 5.7 Hz, CH_3). ^{31}P NMR (162 MHz, CD_3OD) δ 22.3.

Diethyl ((2-methoxyphenyl)(4,5,6,7-tetrahydrobenzo[*b*]thiophene-3-carboxamido)methyl)phosphonate (4f)

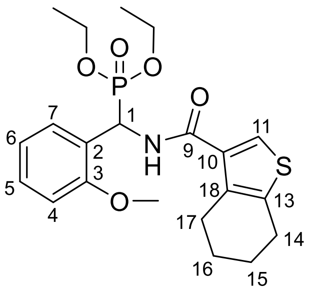

The synthesis was performed according to general procedure A, compound **3** (200 mg, 0.73 mmol) and 4,5,6,7-tetrahydrobenzo*[b]*thiophene-3-carboxylic acid (267 mg, 1.5 mmol, 2.0 equiv.) gave the product as a white solid (299 mg, 0.68 mmol, 93%). HRESIMS (*m/z*): 438.1482 $[M+H]^+$ (438.1504 calcd. for $C_{21}H_{28}NO_5PS$). ^{1}H NMR (500 MHz, MeOD) δ 7.68 (s, 1H, 11-H), 7.57 (dd, J = 7.7, 1.9 Hz, 1H, 7-H), 7.33 (ddd, J = 8.5, 7.6, 1.9 Hz, 1H, 5-H), 7.04 (d, J = 8.5 Hz, 1H, 4-H), 6.99 (dd, J = 7.7, 7.6 Hz, 1H, 6-H), 6.25 (d, $^2J_{HP}$ = 21.5 Hz, 1H, 1-H), 4.15 (dqd, J = 8.6, 7.1, 1.2 ($^3J_{HP}$) Hz, 2H, OCH_2), 3.99 (dq, J = 10.2, 7.1 Hz, 1H, OCH_2), 3.91 (s, 3H, OCH_3), 3.87 (dq, J = 10.2, 7.1 Hz, 1H, OCH_2), 2.82 – 2.67 (m, 4H, 14-H and 17-H), 1.88 – 1.72 (m, 4H, 15-H and 16-H), 1.31 (t, J = 7.1 Hz, 3H, CH_3), 1.15 (t, J = 7.1 Hz, 3H, CH_3). ^{13}C NMR (126 MHz, CD_3OD) δ 166.3 (d, $^3J_{CP}$ = 6.7 Hz, 9-C=O), 158.5 (d, $^3J_{CP}$ = 6.4 Hz, 3-C), 138.6 (13-C), 136.4 (10-C), 135.7 (18-C), 130.8 (d, $^5J_{CP}$ = 2.8 Hz, 5-CH), 130.5 (d, $^3J_{CP}$ = 4.9 Hz, 7-CH), 126.4 (11-CH), 124.3 (2-C), 121.8 (d, $^4J_{CP}$ = 2.5 Hz, 6-CH), 112.2 (d, $^4J_{CP}$ = 1.6 Hz, 4-CH), 64.6 (d, $^2J_{CP}$ = 7.1 Hz, OCH_2), 64.5 (d, $^2J_{CP}$ = 7.3 Hz, OCH_2), 56.3 (OCH_3), 45.2 (d, $^1J_{CP}$ = 158.5 Hz, 1-CH), 26.5 (14-CH_2 or 17-CH_2), 26.1 (14-CH_2 or 17-CH_2), 24.3 15-CH_2 or 16-CH_2), 23.7 (15-CH_2 or 16-CH_2), 16.8 (d, $^3J_{CP}$ = 5.6 Hz, CH_3), 16.5 (d, $^3J_{CP}$ = 5.8 Hz, CH_3). ^{31}P NMR (162 MHz, CD_3OD) δ 22.3.

Diethyl ((2-methoxyphenyl)(4,5,6,7-tetrahydrobenzo[*c*]thiophene-1-carboxamido)methyl)phosphonate (4g)

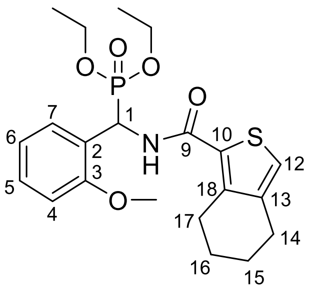

The synthesis was performed according to general procedure A, compound **3** (400 mg, 1.5 mmol) and 4,5,6,7-tetrahydrobenzo*[c]*thiophene-1-carboxylic acid (544 mg, 3.0 mmol, 2.0 equiv.) gave the product as a white solid (590 mg, 1.4 mmol, 92%). HRESIMS (*m/z*): 438.1484 $[M+H]^+$ (438.1504 calcd. for $C_{21}H_{28}NO_5PS$). ^{1}H NMR (500 MHz, CD_3OD) δ 7.46 (dd, J = 7.6, 1.8 Hz, 1H, 7-H), 7.35 (ddd, J = 8.4, 7.5, 1.8 Hz, 1H, 5-H), 7.17 (d, J = 1.2 Hz, 1H, 12-H), 7.06

(dd, J = 8.4, 1.0 Hz, 1H, 4-H), 6.99 (ddd, J = 7.6, 7.5, 1.0 Hz, 1H, 6-H), 6.09 (d, $^{2}J_{HP}$ = 21.5 Hz, 1H, 1-H), 4.14 (dqd, J = 7.3, 7.0, 1.2 ($^{3}J_{HP}$) Hz, 2H, OCH_2), 3.99 (dq, J = 10.2, 7.1 Hz, 1H, OCH_2), 3.87 (dq, J = 10.2, 7.1 Hz, 1H, OCH_2), 2.97 (ddd, J = 17.4, 16.6, 14.1, 6.5, 6.5 Hz, 2H, 17-H), 2.73 (ddd, J = 6.3, 6.3, 1.2 Hz, 2H, 14-H), 1.86 – 1.71 (m, 4H, 15-H and 16-H), 1.31 (t, J = 7.1 Hz, 3H, CH_3), 1.15 (t, J = 7.0 Hz, 3H, CH_3). ^{13}C NMR (126 MHz, CD_3OD) δ 164.4 (d, $^{3}J_{CP}$ = 6.9 Hz, 9-C=O), 158.8 (d, $^{3}J_{CP}$ = 5.6 Hz, 3-C), 142.8 (13-C), 141.4 (18-C), 131.0 (10-C), 131.0 (d, $^{5}J_{CP}$ = 3.0 Hz, 5-CH), 131.0 (d, $^{3}J_{CP}$ = 5.5 Hz, 7-CH), 124.6 (12-CH), 123.9 (2-C), 121.9 (d, $^{4}J_{CP}$ = 2.4 Hz, 6-CH), 112.5 (d, J_{CP} = 2.2 Hz, 4-CH), 64.7 (d, $^{2}J_{CP}$ = 7.1 Hz, OCH_2), 64.6 (d, $^{2}J_{CP}$ = 7.2 Hz, OCH_2), 56.3 (OCH_3), 47.4 (d, $^{1}J_{CP}$ = 158.3 Hz, 1-CH), 27.8 (17-CH_2), 27.3 (14-CH_2), 24.2 (15-CH_2 or 16-CH_2), 24.0 (15-CH_2 or 16-CH_2), 16.8 (d, $^{3}J_{CP}$ = 5.8 Hz, CH_3), 16.5 (d, $^{3}J_{CP}$ = 5.9 Hz, CH_3). ^{31}P NMR (162 MHz, CD_3OD) δ 21.9.

Diethyl (benzamido(2-methoxyphenyl)methyl)phosphonate (4h)

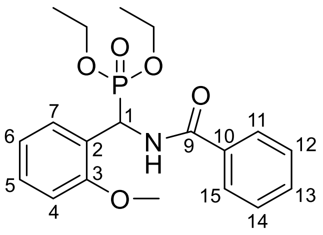

The synthesis was performed according to general procedure A, compound **3** (169 mg, 0.62 mmol) and benzoic acid (149 mg, 1.2 mmol, 2.0 equiv.) gave the product as a white solid (203 mg, 0.54 mmol, 87%). HRESIMS (*m/z*): 378.1465 $[M+H]^+$ (378.1470 calcd. for $C_{19}H_{24}NO_5P$). ^{1}H NMR (500 MHz, CD_3OD) δ 7.85 – 7.79 (m, 2H, 11-H and 15-H), 7.63 (dd, J = 7.6, 1.9 Hz, 1H, 7-H), 7.55 (tt, J = 6.9, 1.3 Hz, 1H, 13-H), 7.51 – 7.44 (m, 2H, 12-H and 14-H), 7.34 (ddd, J = 8.7, 7.6, 1.9 Hz, 1H, 5-H), 7.04 (d, J = 8.7 Hz, 1H, 4-H), 6.99 (dd, J = 7.6, 7.6 Hz, 1H, 6-H), 6.3 (d, $^{2}J_{HP}$ = 21.5 Hz, 1H, 1-H), 4.15 (dqd, J = 8.3, 7.1, 1.3 ($^{3}J_{HP}$) Hz, 2H, OCH_2), 4.00 (dq, J = 10.2, 7.1 Hz, 1H, OCH_2), 3.92 (s, 3H, OCH_3), 3.88 (dq, J = 10.2, 7.1 Hz, 1H, OCH_2), 1.30 (t, J = 7.1 Hz, 3H, CH_3), 1.15 (t, J = 7.1 Hz, 3H, CH_3). ^{13}C NMR (126 MHz, CD_3OD) δ 169.7 (d, $^{3}J_{CP}$ = 6.5 Hz, 9-C=O), 158.5 (d, $^{3}J_{CP}$ = 6.6 Hz, 3-C), 135.4 (d, $^{4}J_{CP}$ = 5.3 Hz, 10-C), 133.0 (13-CH), 130.8 (d, $^{5}J_{CP}$ = 3.0 Hz, 5-CH), 130.6 (d, $^{3}J_{CP}$ = 4.8 Hz, 7-CH), 129.6 (12-CH and 14-CH), 128.6 (11-CH and 15-CH), 124.2 (d, $^{2}J_{CP}$ = 3.2 Hz, 2-C), 121.8 (d, $^{4}J_{CP}$ = 2.5 Hz, 6-CH), 112.2 (d, $^{4}J_{CP}$ = 1.8 Hz, 4-CH), 64.7 (d, $^{2}J_{CP}$ = 7.2 Hz, OCH_2), 64.6 (d, $^{2}J_{CP}$ = 7.2 Hz, OCH_2), 56.3 (OCH_3), 45.7 (d, $^{1}J_{CP}$ = 158.7 Hz, 1-CH), 16.8 (d, $^{3}J_{CP}$ = 5.7 Hz, CH_3), 16.5 (d, $^{3}J_{CP}$ = 6.0 Hz, CH_3). ^{31}P NMR (162 MHz, CD_3OD) δ 22.3.

Diethyl ((2-methoxyphenyl)(2-(thiophen-2-yl)acetamido)methyl)phosphonate (4i)

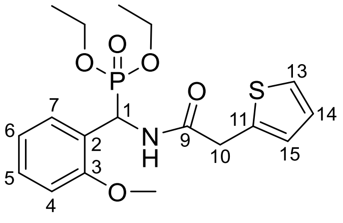

The synthesis was performed according to general procedure A, compound **3** (150 mg, 0.55 mmol) and 2-(thiophen-2-yl)acetic acid (159 mg, 1.1 mmol, 2.0 equiv.) gave the product as a light orange solid (161 mg, 0.41 mmol, 74%). HRESIMS (*m/z*): 398.1223 $[M+H]^+$ (398.1191 calcd. for $C_{18}H_{24}NO_5PS$). ^1H NMR (500 MHz, CD_3OD) δ 7.48 (dd, J = 7.7, 1.9 Hz, 1H, 7-H), 7.31 (ddd, J = 8.2, 7.7, 1.9 Hz, 1H, 5-H), 7.27 (dd, J = 4.8, 1.6 Hz, 1H, 13-H), 7.00 (d, J = 8.2 Hz, 1H, 4-H), 6.98 (dd, J = 7.7, 7.7 Hz, 1H, 6-H), 6.97 – 6.93 (m, 2H, 14-H and 15-H), 6.07 (d, $^2J_{HP}$ = 21.0 Hz, 1H, 1-H), 4.07 (dq, J = 10.2, 7.1 Hz, 1H, OCH_2), 4.02 (dq, J = 10.2, 7.1 Hz, 1H, OCH_2), 3.95 (dq, J = 10.1, 7.1 Hz, 1H, OCH_2), 3.89 – 3.80 (m, 5H, 10-H, OCH_2 and OCH_3), 3.78 (d, $^2J_{HH}$ = 15.3 Hz, 1H, 10-H), 1.24 (t, J = 7.1 Hz, 3H, CH_3), 1.12 (t, J = 7.1 Hz, 3H, CH_3). ^{13}C NMR (126 MHz, CD_3OD) δ 171.9 (d, $^3J_{CP}$ = 6.3 Hz, 9-C=O), 158.4 (d, $^3J_{CP}$ = 6.5 Hz, 3-C), 137.7 (11-C), 130.8 (d, $^5J_{CP}$ = 2.9 Hz, 5-CH), 130.4 (d, $^3J_{CP}$ = 4.8 Hz, 7-CH), 127.8 (d, J = 3.5 Hz, 14-CH and 15-CH), 126.0 (13-CH), 124.0 (2-C), 121.7 (d, $^4J_{CP}$ = 2.6 Hz, 6-CH), 112.1 (d, $^4J_{CP}$ = 1.9 Hz, 4-CH), 64.5 (d, $^2J_{CP}$ = 7.3 Hz, OCH_2), 64.5 (d, $^2J_{CP}$ = 6.8 Hz, OCH_2), 56.2 (OCH_3), 45.2 (d, $^1J_{CP}$ = 158.5 Hz, 1-CH), 37.5 (10-CH_2), 16.7 (d, $^3J_{CP}$ = 5.8 Hz, CH_3), 16.5 (d, $^3J_{CP}$ = 6.0 Hz, CH_3). ^{31}P NMR (162 MHz, CD_3OD) δ 21.8.

Diethyl ((2-methoxyphenyl)(2-(thiophen-3-yl)acetamido)methyl)phosphonate (4j)

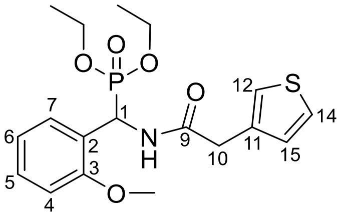

The synthesis was performed according to general procedure A, compound **3** (80 mg, 0.29 mmol) and 2-(thiophen-3-yl)acetic acid (85 mg, 0.60 mmol, 2.0 equiv.) gave the product as a white solid (95 mg, 0.24 mmol, 82%). HRESIMS (*m/z*): 398.1195 $[M+H]^+$ (398.1191 calcd. for $C_{18}H_{24}NO_5PS$). ^1H NMR (500 MHz, CD_3OD) δ 7.47 (dd, J = 7.6, 1.9 Hz, 1H, 7-H), 7.36 (dd, J = 5.0, 3.0 Hz, 1H, 14-H), 7.32 (ddd, J = 8.7, 7.6, 1.9 Hz, 1H, 5-H), 7.22 (dd, J = 3.0, 1.6 Hz, 1H, 12-H), 7.04 (dd, J = 5.0, 1.6 Hz, 1H, 15-H), 7.00 (d, J = 8.7 Hz, 1H, 4-H), 6.97 (dd, J = 7.6, 7.6 Hz, 1H, 6-H), 6.08 (d, $^2J_{HP}$ = 21.0 Hz, 1H, 1-H), 4.06 (dq, J = 10.1, 7.1 Hz, 1H, OCH_2), 4.01 (dq, J = 10.1, 7.1 Hz, 1H, OCH_2), 3.94 (dq, J = 10.1, 7.0 Hz, 1H, OCH_2), 3.84 (s, 3H, OCH_3), 3.84 (dq, J = 10.1, 7.0 Hz, 1H, OCH_2), 3.67 (d, $^2J_{HH}$ = 14.7 Hz, 1H, 10-H), 3.59 (d, $^2J_{HH}$ = 14.7 Hz, 1H, 10-H), 1.23 (t, J = 7.1 Hz, 3H, CH_3), 1.11 (t, J = 7.0 Hz, 3H, CH_3). ^{13}C NMR (126 MHz, CD_3OD) δ 172.6 (d, $^3J_{CP}$ = 6.3 Hz, 9-C=O), 158.4 (d, $^3J_{CP}$ = 6.5 Hz, 3-C), 136.3 (11-C), 130.8 (d, $^5J_{CP}$ = 2.9 Hz, 5-CH), 130.4 (d, $^3J_{CP}$ = 4.8 Hz, 7-CH), 129.4 (15-CH), 126.8 (14-CH), 124.1 (2-C), 123.7 (12-CH), 121.7 (d, $^4J_{CP}$ = 2.6 Hz, 6-CH), 112.1 (d, $^4J_{CP}$ = 1.9 Hz, 4-CH), 64.5 (d, $^2J_{CP}$ = 7.0 Hz, OCH_2), 64.5 (d, $^2J_{CP}$ = 7.4 Hz, OCH_2), 56.2 (OCH_3), 45.2 (d, $^1J_{CP}$ = 158.5 Hz, 1-CH), 38.1 (10-CH_2), 16.7 (d, $^3J_{CP}$ = 5.8 Hz, CH_3), 16.5 (d, $^3J_{CP}$ = 6.0 Hz, CH_3). ^{31}P NMR (162 MHz, CD_3OD) δ 21.9.

Diethyl ((2-(benzo[*b*]thiophen-2-yl)acetamido)(2-methoxyphenyl)methyl)phosphonate (4k)

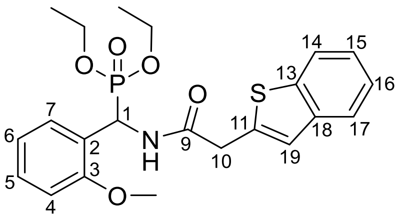

The synthesis was performed according to general procedure A, compound **3** (400 mg, 1.5 mmol) and 2-(benzo*[b]*thiophen-2-yl)acetic acid (574 mg, 3.0 mmol, 2.0 equiv.) gave the product as a light orange solid (400 mg, 0.89 mmol, 61%). HRESIMS (*m/z*): 448.1341 $[M+H]^+$ (448.1347 calcd. for $C_{22}H_{26}NO_5PS$). ^1H NMR (500 MHz, CD_3OD) δ 7.78 (ddt, *J* = 7.6, 1.5, 0.9 Hz, 1H, 14-H), 7.71 (dt, *J* = 8.0, 0.9 Hz, 1H, 17-H), 7.50 (dd, *J* = 7.6, 2.0 Hz, 1H, 7-H), 7.32 (ddd, *J* = 8.4, 7.6, 2.0 Hz, 1H, 5-H), 7.32 – 7.25 (m, 2H, 15-H and 16-H), 7.22 (d, *J* = 0.9 Hz, 1H, 19-H), 6.99 (dd, *J* = 8.4, 0.9 Hz, 1H, 4-H), 6.97 (ddd, *J* = 7.6, 7.6, 0.9 Hz, 1H, 6-H), 6.10 (d, $^2J_{HP}$ = 20.9 Hz, 1H, 1-H), 4.02 (m, 2H, OCH_2), 3.95 (d, $^2J_{HH}$ = 15.2 Hz, 1H, 10-H), 3.94 (m, 1H, OCH_2), 3.87 (d, $^2J_{HH}$ = 15.2 Hz, 1H, 10-H), 3.85 (m, 1H, OCH_2), 3.79 (s, 3H, OCH_3), 1.18 (t, *J* = 7.0 Hz, 3H, CH_3), 1.10 (t, *J* = 7.1 Hz, 3H, CH_3). ^{13}C NMR (126 MHz, CD_3OD) δ 171.3 (d, $^3J_{CP}$ = 6.4 Hz, 9-C=O), 158.3 (d, $^3J_{CP}$ = 6.8 Hz, 3-C), 141.4 (13-C), 141.3 (18-C), 139.0 (11-C), 130.8 (d, $^5J_{CP}$ = 2.8 Hz, 5-CH), 130.4 (d, $^3J_{CP}$ = 4.7 Hz, 7-CH), 125.4 (16-CH), 125.2 (15-CH), 124.5 (19-CH), 124.2 (17-CH), 124.0 (2-C), 123.0 (14-CH), 121.7 (d, $^4J_{CP}$ = 2.5 Hz, 6-CH), 112.1 (d, $^4J_{CP}$ = 1.8 Hz, 4-CH), 64.5 (d, $^2J_{CP}$ = 6.9 Hz, OCH_2), 64.5 (d, $^2J_{CP}$ = 7.3 Hz, OCH_2), 56.2 (OCH_3), 45.3 (d, $^1J_{CP}$ = 158.6 Hz, 1-CH), 38.4 (10-CH_2), 16.7 (d, $^3J_{CP}$ = 5.8 Hz, CH_3), 16.5 (d, $^3J_{CP}$ = 6.0 Hz, CH_3). ^{31}P NMR (162 MHz, CD_3OD) δ 21.7.

Diethyl ((2-(benzo[*b*]thiophen-3-yl)acetamido)(2-methoxyphenyl)methyl)phosphonate (4l)

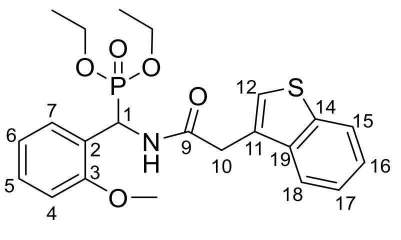

The synthesis was performed according to general procedure A, compound **3** (400 mg, 1.5 mmol) and 2-(benzo*[b]*thiophen-3-yl)acetic acid (573 mg, 3.0 mmol, 2.0 equiv.) gave the product as a white solid (603 mg, 1.3 mmol, 92%). HRESIMS (*m/z*): 448.1334 $[M+H]^+$ (448.1347 calcd. for $C_{22}H_{26}NO_5PS$). ^1H NMR (500 MHz, CD_3OD) δ 7.87 (ddt, *J* = 6.5, 4.1, 2.1 Hz, 1H, 15-H), 7.79 (ddt, *J* = 6.5, 4.1, 2.1 Hz, 1H, 18-H), 7.47 (dd, *J* = 7.5, 1.9 Hz, 1H, 7-H), 7.44 (s, 1H, 12-H), 7.37 – 7.32 (m, 2H, 16-H and 17-H), 7.31 (ddd, *J* = 8.2, 7.5, 1.9 Hz, 1H, 5-H), 6.97 (dd, *J* = 7.5, 7.5 Hz, 1H, 6-H), 6.96 (d, *J* = 8.2 Hz, 1H, 4-H), 6.07 (d, $^2J_{HP}$ = 21.0 Hz, 1H, 1-H), 4.05 – 3.87 (m, 3H, OCH_2), 3.91 (d, $^2J_{HH}$ = 15.7 Hz, 1H, 10-H), 3.84 (d, $^2J_{HH}$ = 15.7

Hz, 1H, 10-H), 3.82 (m, 1H, OCH_2), 3.74 (s, 3H, OCH_3), 1.16 (t, J = 7.1 Hz, 3H, CH_3), 1.08 (t, J = 7.1 Hz, 3H, CH_3). ^{13}C NMR (126 MHz, CD_3OD) δ 172.0 (d, $^3J_{CP}$ = 6.3 Hz, 9-C=O), 158.4 (d, $^3J_{CP}$ = 6.4 Hz, 3-C), 141.7 (14-C), 140.0 (19-C), 130.9 (11-C), 130.8 (d, $^5J_{CP}$ = 2.8 Hz, 5-CH), 130.5 (d, $^3J_{CP}$ = 4.8 Hz, 7-CH), 125.8 (12-CH), 125.5 (16-CH or 17-CH), 125.2 (16-CH or 17-CH), 123.9 (2-C), 123.7 (15-CH), 123.0 (18-CH), 121.7 (d, $^4J_{CP}$ = 2.6 Hz, 6-CH), 112.1 (d, ^4J = 2.1 Hz, 4-CH), 64.5 (d, $^2J_{CP}$ = 6.9 Hz, OCH_2), 64.4 (d, $^2J_{CP}$ = 7.2 Hz, OCH_2), 56.1 (OCH_3), 45.6 (d, $^1J_{CP}$ = 158.6 Hz, 1-CH), 36.6 (10-CH_2), 16.6 (d, $^3J_{CP}$ = 5.9 Hz, CH_3), 16.5 (d, $^3J_{CP}$ = 5.9 Hz, CH_3). ^{31}P NMR (162 MHz, CD_3OD) δ 21.7.

Diethyl ((2-methoxyphenyl)(2-phenylacetamido)methyl)phosphonate (4m)

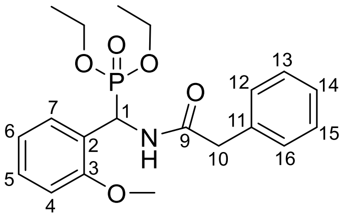

The synthesis was performed according to general procedure A, compound **3** (150 mg, 0.55 mmol) and phenylacetic acid (152 mg, 1.1 mmol, 2.0 equiv.) gave the product as a white solid (173 mg, 0.44 mmol, 81%). HRESIMS (*m/z*): 392.1623 $[M+H]^+$ (392.163 calcd. for $C_{20}H_{26}NO_5P$). ^{1}H NMR (500 MHz, CD_3OD) δ 7.50 (dd, J = 7.5, 2.0 Hz, 1H, 7-H), 7.31 (ddd, J = 8.3, 7.5, 2.0 Hz, 1H, 5-H), 7.31 – 7.27 (m, 4H, 12-H, 13-H, 15-H and 16-H), 7.24 (tt, J = 4.9, 3.8 Hz, 1H, 14-H), 6.99 (d, J = 8.3 Hz, 1H, 4-H), 6.97 (dd, J = 7.5, 7.5 Hz, 1H, 6-H), 6.09 (d, $^2J_{HP}$ = 21.0 Hz, 1H, 1-H), 4.04 (dq, J = 10.2, 7.1 Hz, 1H, OCH_2) 3.98 (dq, J = 10.2, 7.1 Hz, 1H, OCH_2), 3.93 (dq, J = 10.2, 7.1 Hz, 1H, OCH_2), 3.83 (dq, J = 10.2, 7.1 Hz, 1H, OCH_2), 3.81 (s, 3H, OCH_3), 3.64 (d, $^2J_{HH}$ = 14.2 Hz, 1H, 10-H), 3.56 (d, $^2J_{HH}$ = 14.3 Hz, 1H, 10-H), 1.21 (t, J = 7.1 Hz, 3H, CH_3), 1.10 (t, J = 7.1 Hz, 3H, CH_3). ^{13}C NMR (126 MHz, CD_3OD) δ 171.6 (d, $^3J_{CP}$ = 6.2 Hz, 9-C=O), 156.9 (d, $^3J_{CP}$ = 6.7 Hz, 3-C), 135.3 (11-C), 129.4 (d, $^5J_{CP}$ = 2.8 Hz, 5-CH), 129.0 (d, $^3J_{CP}$ = 4.8 Hz, 7-CH), 128.8 (12-CH and 16-CH or 13-CH and 15-CH), 128.2 (12-CH and 16-CH or 13-CH and 15-CH), 126.6 (14-CH), 122.7 (2-C), 120.3 (d, $^4J_{CP}$ = 2.6 Hz, 6-CH), 110.7 (d, $^4J_{CP}$ = 2.0 Hz, 4-CH), 63.0 (d, $^2J_{CP}$ = 7.0 Hz, OCH_2), 63.0 (d, $^2J_{CP}$ = 7.1 Hz, OCH_2), 54.8 (OCH_3), 43.7 (d, $^1J_{CP}$ = 158.5 Hz, 1-CH), 42.0 (10-CH_2), 15.3 (d, $^3J_{CP}$ = 5.8 Hz, CH_3), 15.1 (d, $^3J_{CP}$ = 6.0 Hz, CH_3). ^{31}P NMR (162 MHz, CD_3OD) δ 21.9.

General procedure B for 5a-m.

The synthetic procedure was adapted from that described by Gauvry et al.[5] BBr_3 (5 equiv., 1M in DCM) was added dropwise to the solution of compound **4a-m** in toluene at -78 °C and the reaction mixture was stirred for 1 h. The mixture was allowed to warm to r.t. and was stirred for 1 h. Then the mixture was heated to 60 °C and was stirred for 7h. An excess of MeOH was added at r.t. The solvens were removed under reduced pressure. The crude product was washed with toluene (3x) and was purified by preparative HPLC (C8 column, MeCN:H_2O with 0.1% formic acid). The products **5a-m** were obtained in a yield of 46-86%.

((2-Hydroxyphenyl)(thiophene-2-carboxamido)methyl)phosphonic acid (5a)

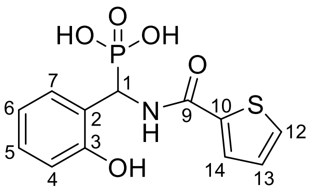

The synthesis was performed according to general procedure B, compound **4a** (40 mg, 0.1 mmol, in 5 mL toluene) gave the product as a white solid (15 mg, 0.05 mmol, 46%). HRESIMS (*m/z*): 314.0258 $[M+H]^+$ (314.0252 calcd. for $C_{12}H_{12}NO_5PS$). ^{1}H NMR (500 MHz, D_2O) δ 7.80 (dd, J = 4.4, 1.0 Hz, 1H, 14-H), 7.69 (dd, J = 4.4, 1.0 Hz, 1H, 12-H), 7.39 (dd, J = 7.8, 1.7 Hz, 1H, 7-H), 7.21 (ddd, J = 8.1, 7.8, 1.7 Hz, 1H, 5-H), 7.16 (dd, J = 4.4, 4.4 Hz, 1H, 13-H), 6.95 (dd, J = 7.8, 7.8 Hz, 1H, 6-H), 6.92 (d, J = 8.1 Hz, 1H, 4-H), 5.61 (d, $^2J_{HP}$ = 20.5 Hz, 1H, 1-H). ^{13}C NMR (151 MHz, D_2O) δ 164.0 (d, $^3J_{CP}$ = 8.7 Hz, 9-C=O), 153.5 (d, $^3J_{CP}$ = 4.6 Hz, 3-C), 136.8 (10-C), 131.8 (12-CH), 130.0 (14-CH), 129.0 (d, $^5J_{CP}$ = 1.7 Hz, 5-CH), 128.6 (d, $^3J_{CP}$ = 4.5 Hz, 7-CH), 128.2 (13-CH), 124.4 (d, $^2J_{CP}$ = 1.2 Hz, 2-C), 120.9 (d, $^4J_{CP}$ = 1.5 Hz, 6-CH), 116.9 (d, $^4J_{CP}$ = 1.6 Hz, 4-CH), 48.6 (d, $^1J_{CP}$ = 144.8 Hz, 1-CH). ^{31}P NMR (162 MHz, D_2O) δ 16.4.

((2-Hydroxyphenyl)(thiophene-3-carboxamido)methyl)phosphonic acid (5b)

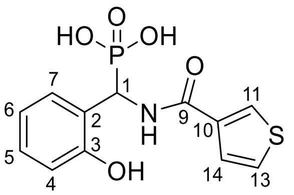

The synthesis was performed according to general procedure B, compound **4b** (40 mg, 0.1 mmol, in 5 mL toluene) gave the product as a white solid (20 mg, 0.06 mmol, 61%). HRESIMS (*m/z*): 314.0258 $[M+H]^+$ (314.0252 calcd. for $C_{12}H_{12}NO_5PS$). ^{1}H NMR (400 MHz, D_2O) δ 8.14 (s, 1H, 11-H), 7.55 – 7.48 (m, 2H, 13-H and 14-H), 7.40 (d, J = 7.7 Hz, 1H, 7-H), 7.25 (dd, J = 8.0, 7.7 Hz, 1H, 5-H), 6.99 (dd, J = 7.7, 7.7 Hz, 1H, 6-H), 6.96 (d, J = 8.0 Hz, 1H, 4-H), 5.61 (d, $^2J_{HP}$ = 20.6 Hz, 1H, 1-H). ^{13}C NMR (126 MHz, D_2O) δ 165.4 (d, $^3J_{CP}$ = 8.6 Hz, 9-C=O), 153.4 (d, $^3J_{CP}$ = 4.8 Hz, 3-C), 135.4 (10-C), 130.0 (11-CH), 129.0 (d, $^5J_{CP}$ = 2.4 Hz, 5-CH), 128.4 (d, $^3J_{CP}$ = 4.6 Hz, 7-CH), 127.2 (13-CH), 126.0 (14-CH), 124.4 (d, $^2J_{CP}$ = 1.0 Hz, 2-C), 120.9 (d, $^4J_{CP}$ = 1.2 Hz, 6-CH), 116.8 (d, $^4J_{CP}$ = 1.7 Hz, 4-CH), 48.4 (d, $^1J_{CP}$ = 144.4 Hz, 1-CH). ^{31}P NMR (162 MHz, D_2O) δ 16.5.

((Benzo[*b*]thiophene-2-carboxamido)(2-hydroxyphenyl)methyl)phosphonic acid (5c)

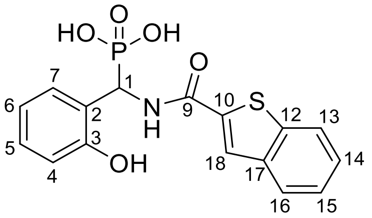

The synthesis was performed according to general procedure B, compound **4c** (57 mg, 0.1 mmol, in 7 mL toluene) gave the product as a white solid (26 mg, 0.07 mmol, 54%). HRESIMS (*m/z*): 364.0415 $[M+H]^+$ (364.0408 calcd. for $C_{16}H_{14}NO_5PS$). ^{1}H NMR (601 MHz, D_2O) δ 8.01 (s, 1H, 18-H), 7.95 – 7.90 (m, 2H, 13-H and 16-H), 7.48 – 7.39 (m, 3H, 7-H, 14-H and 15-H), 7.26 (dd, J = 8.1, 7.7 Hz, 1H, 5-H), 7.01 (dd, J = 7.7, 7.7 Hz, 1H, 6-H), 6.98 (d, J = 8.1 Hz, 1H, 4-H), 5.66 (d, $^2J_{HP}$ = 20.5 Hz, 1H, 1-H). ^{13}C NMR (151 MHz, D_2O) δ 164.2 (d, $^3J_{CP}$ = 8.2 Hz, 9-C=O), 153.6 (d, $^3J_{CP}$ = 3.6 Hz, 3-C), 140.7 (12-C), 138.9 (17-C), 136.8 (10-C), 129.2 (d, $^5J_{CP}$ = 1.9 Hz, 5-CH), 128.8 (d, $^3J_{CP}$ = 4.9 Hz, 7-CH), 126.8 (18-CH), 126.8 (14-CH or 15-CH), 125.5 (16-CH), 125.2 (14-CH or 15-CH), 124.1 (d, $^2J_{CP}$ = 1.8 Hz, 2-C), 122.7 (13-CH), 120.9 (d, $^4J_{CP}$ = 1.9 Hz, 6-CH), 116.8 (d, $^4J_{CP}$ = 1.8 Hz, 4-CH), 48.8 (d, $^1J_{CP}$ = 145.0 Hz, 1-CH). ^{31}P NMR (162 MHz, D_2O) δ 16.4.

((Benzo[*b*]thiophene-3-carboxamido)(2-hydroxyphenyl)methyl)phosphonic acid (5d)

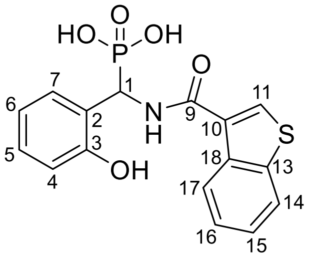

The synthesis was performed according to general procedure B, compound **4d** (290 mg, 0.7 mmol, in 38 mL toluene) gave the product as a white solid (196 mg, 0.5 mmol, 81%). HRESIMS (*m/z*): 364.0415 $[M+H]^+$ (364.0408 calcd. for $C_{16}H_{14}NO_5PS$). ^{1}H NMR (500 MHz, D_2O) δ 8.21 (s, 1H, 11-H), 8.05 (dd, J = 7.2, 2.0 Hz, 1H, 14-H or 17-H), 7.93 (dd, J = 6.9, 1.8 Hz, 1H, 14-H or 17-H), 7.46 – 7.37 (m, 3H, 7-H, 15-H and 16-H), 7.23 (ddd, J = 8.5, 7.6, 1.6 Hz, 1H, 5-H), 6.97 (dd, J = 7.6, 7.6 Hz, 1H, 6-H), 6.95 (d, J = 8.5 Hz, 1H, 4-H), 5.70 (d, $^2J_{HP}$ = 20.6 Hz, 1H, 1-H). ^{13}C NMR (126 MHz, D_2O) δ 166.2 (d, $^3J_{CP}$ = 8.9 Hz, 9-C=O), 153.6 (d, $^3J_{CP}$ = 5.1 Hz, 3-C), 139.8 (13-C), 136.1 (18-C), 131.8 (11-C), 129.9 (10-C), 129.1 (d, $^5J_{CP}$ = 2.4 Hz, 5-CH), 128.6 (d, $^3J_{CP}$ = 4.7 Hz, 7-CH), 125.1 (2 x CH, 15-CH and 16-CH), 124.0 (d, $^2J_{CP}$ = 1.5 Hz, 2-C), 123.2 (14-CH or 17-CH), 122.8 (14-CH or 17-CH), 120.9 (d, $^4J_{CP}$ = 1.8 Hz, 6-CH), 116.7 (d, $^4J_{CP}$ = 1.3 Hz, 4-CH), 48.2 (d, $^1J_{CP}$ = 145.8 Hz, 1-CH). ^{31}P NMR (162 MHz, D_2O) δ 16.8.

((2-Hydroxyphenyl)(4,5,6,7-tetrahydrobenzo[*b*]thiophene-2-carboxamido)methyl)phosphonic acid (5e)

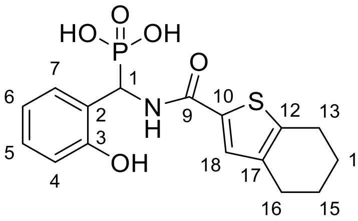

The synthesis was performed according to general procedure B, compound **4e** (75 mg, 0.2 mmol, in 9 mL toluene) gave the product as a white solid (46 mg, 0.1 mmol, 73%). HRESIMS (*m/z*): 368.0725 $[M+H]^+$ (368.0721 calcd. for $C_{16}H_{18}NO_5PS$). ^{1}H NMR (500 MHz, D_2O) δ 7.48 (s, 1H, 18-H), 7.35 (d, *J* = 7.7 Hz, 1H, 7-H), 7.21 (dd, *J* = 8.2, 7.6 Hz, 1H, 5-H), 6.95 (dd, *J* = 7.7, 7.6 Hz, 1H, 6-H), 6.92 (d, *J* = 8.2 Hz, 1H, 4-H), 5.53 (d, $^2J_{HP}$ = 20.7 Hz, 1H, 1-H), 2.73 (dd, *J* = 6.0, 6.0 Hz, 2H, 13-H), 2.58 (dd, *J* = 7.1, 7.1 Hz, 2H, 16-H), 1.82 – 1.68 (m, 4H, 14-H and 15-H). ^{13}C NMR (126 MHz, D_2O) δ 164.1 (d, $^3J_{CP}$ = 8.9 Hz, 9-C=O), 153.4 (d, $^3J_{CP}$ = 5.0 Hz, 3-C), 143.5 (12-C), 137.1 (17-C), 132.3 (10-C), 130.8 (18-CH), 129.0 (d, $^5J_{CP}$ = 2.0 Hz, 5-CH), 128.4 (d, $^3J_{CP}$ = 4.6 Hz, 7-CH), 124.4 (d, $^2J_{CP}$ = 1.7 Hz, 2-C), 120.9 (d, $^4J_{CP}$ = 2.0 Hz, 6-CH), 116.8 (d, $^4J_{CP}$ = 2.0 Hz, 4-CH), 48.6 (d, $^1J_{CP}$ = 144.2 Hz, 1-CH), 24.8 (16-CH_2), 24.7 (13-CH_2), 22.7 (14-CH_2 or 15-CH_2), 22.0 (14-CH_2 or 15-CH_2). ^{31}P NMR (162 MHz, D_2O) δ 16.4.

((2-Hydroxyphenyl)(4,5,6,7-tetrahydrobenzo[*b*]thiophene-3-carboxamido)methyl)phosphonic acid (5f)

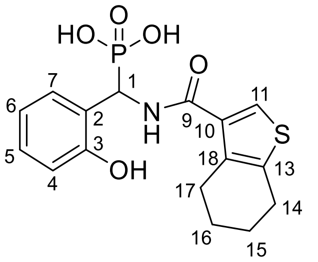

The synthesis was performed according to general procedure B, compound **4f** (40 mg, 0.1 mmol, in 5 mL toluene) gave the product as a white solid (23 mg, 0.06 mmol, 69%). HRESIMS (*m/z*): 368.0723 $[M+H]^+$ (368.0721 calcd. for $C_{16}H_{18}NO_5PS$). ^{1}H NMR (601 MHz, D_2O) δ 7.75 (s, 1H, 11-H), 7.37 (dd, *J* = 7.7, 1.8 Hz, 1H, 7-H), 7.25 (ddd, *J* = 8.5, 7.6, 1.8 Hz, 1H, 5-H), 6.99 (dd, *J* = 7.7, 7.6 Hz, 1H, 6-H), 6.95 (d, *J* = 8.5 Hz, 1H, 4-H), 5.53 (d, $^2J_{HP}$ = 20.5 Hz, 1H, 1-H), 2.75 (dd, *J* = 6.1, 6.1 Hz, 2H, 14-H), 2.63 (ddd, *J* = 22.8, 16.8, 6.2 Hz, 2H, 17-H), 1.80 (dddd, *J* = 6.1, 6.1, 6.0, 6.0 Hz, 2H, 15-H), 1.73 (ddddd, *J* = 16.8, 6.2, 6.2, 6.0, 6.0 Hz, 2H, 16-H). ^{13}C NMR (151 MHz, D_2O) δ 167.1 (d, $^3J_{CP}$ = 8.0 Hz, 9-C=O), 153.5 (d, $^3J_{CP}$ = 4.3 Hz, 3-C), 138.3 (13-C), 134.7 (10-C), 133.8 (18-C), 129.0 (d, $^5J_{CP}$ = 2.1 Hz, 5-CH), 128.5 (d, $^3J_{CP}$ = 3.5 Hz, 7-CH), 125.9 (11-CH), 124.8 (d, $^2J_{CP}$ = 1.7 Hz, 2-C), 121.0 (d, $^4J_{CP}$ = 1.6 Hz, 6-CH), 117.0 (d, $^4J_{CP}$ = 1.7 Hz, 4-CH), 48.7 (d, $^1J_{CP}$ = 143.4 Hz, 1-CH), 24.8 (17-CH_2), 24.6 (14-CH_2), 22.6 (15-CH_2), 22.1 (16-CH_2). ^{31}P NMR (162 MHz, D_2O) δ 16.2.

((2-Hydroxyphenyl)(4,5,6,7-tetrahydrobenzo[*c*]thiophene-1-carboxamido)methyl)-phosphonic acid (5g)

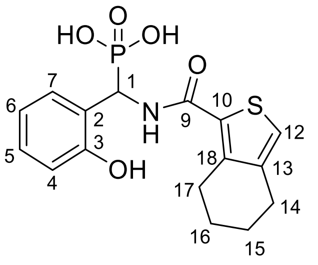

The synthesis was performed according to general procedure B, compound **4g** (47 mg, 0.1 mmol, in 6 mL toluene) gave the product as a pale yellow solid (33 mg, 0.09 mmol, 84%). HRESIMS (*m/z*): 368.0727 $[M+H]^+$ (368.0721 calcd. for $C_{16}H_{18}NO_5PS$). ^{1}H NMR (601 MHz, D_2O) δ 7.36 (dd, *J* = 7.6, 1.9 Hz, 1H, 7-H), 7. 25 (ddd, *J* = 8.1, 7.6, 1.9 Hz, 1H, 5-H), 7.24 (m, 1H, 12-H), 6.98 (dd, *J* = 7.6, 7.6 Hz, 1H, 6-H), 6.95 (d, *J* = 8.1 Hz, 1H, 4-H), 5.53 (d, $^2J_{HP}$ = 20.3 Hz, 1H, 1-H), 2.94 (ddd, *J* = 25.1, 16.9, 16.8, 6.5 Hz, 2H, 17-H), 2.70 (dd, *J* = 6.3, 6.3 Hz, 2H, 14-H), 1.77 (dddd, *J* = 16.9, 6.5, 3.6, 3.6 Hz, 2H, 16-H), 1.71 (dddd, *J* = 6.3, 6.3, 3.6, 3.6 Hz, 2H, 15-H). ^{13}C NMR (151 MHz, D_2O) δ 164.8 (d, $^3J_{CP}$ = 8.2 Hz, 9-C=O), 153.6 (d, $^3J_{CP}$ = 3.8 Hz, 3-C), 142.0 (13-C), 140.8 (18-C), 129.4 (10-C), 129.1 (d, $^5J_{CP}$ = 2.2 Hz, 5-CH), 128.8 (d, $^3J_{CP}$ = 4.2 Hz, 7-CH), 124.5 (d, $^2J_{CP}$ = 2.1 Hz, 2-C), 124.2 (12-CH), 121.0 (d, $^4J_{CP}$ = 1.5 Hz, 6-CH), 117.0 (d, $^4J_{CP}$ = 1.7 Hz, 4-CH), 49.4 (d, $^1J_{CP}$ = 143.3 Hz, 1-CH), 26.4 (17-CH_2), 25.8 (14-CH_2), 22.5 (15-CH_2 or 16-CH_2), 22.2 (15-CH_2 or 16-CH_2). ^{31}P NMR (162 MHz, D_2O) δ 16.1.

(Benzamido(2-hydroxyphenyl)methyl)phosphonic acid (5h)

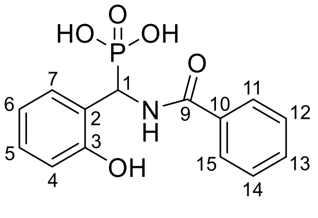

The synthesis was performed according to general procedure B, compound **4h** (100 mg, 0.3 mmol, in 12 mL toluene) gave the product as a white solid (60 mg, 0.2 mmol, 74%). HRESIMS (*m/z*): 308.0696 $[M+H]^+$ (308.0610 calcd. for $C_{14}H_{14}NO_5P$). ^{1}H NMR (500 MHz, D_2O) δ 7.77 (d, *J* = 7.8 Hz, 2H, 11-H and 15-H), 7.59 (t, *J* = 7.8 Hz, 1H, 13-H), 7.50 (t, *J* = 7.8 Hz, 2H, 12-H and 14-H), 7.38 (dd, *J* = 7.7, 1.7 Hz, 1H, 7-H), 7.21 (ddd, *J* = 8.0, 7.6, 1.7 Hz, 1H, 5-H), 6.94 (dd, *J* = 7.7, 7.6 Hz, 1H, 6-H), 6.93 (d, *J* = 8.0 Hz, 1H, 4-H), 5.63 (d, $^2J_{HP}$ = 20.5 Hz, 1H, 1-H). ^{13}C NMR (126 MHz, D_2O) δ 170.5 (d, $^3J_{CP}$ = 8.5 Hz, 9-C=O), 153.5 (d, $^3J_{CP}$ = 4.5 Hz, 3-C), 133.4 (10-C), 132.2 (13-CH), 129.0 (5-CH), 128.7 (2 x CH, 12-CH and 14-CH), 128.5 (d, $^3J_{CP}$ = 4.8 Hz, 7-CH), 127.2 (2 x CH, 11-CH and 15-CH), 124.2 (2-C), 120.9 (6-CH), 116.7 (4-CH), 48.6 (d, $^1J_{CP}$ = 144.7 Hz, 1-CH). ^{31}P NMR (162 MHz, D_2O) δ 16.8.

((2-Hydroxyphenyl)(2-(thiophen-2-yl)acetamido)methyl)phosphonic acid (5i)

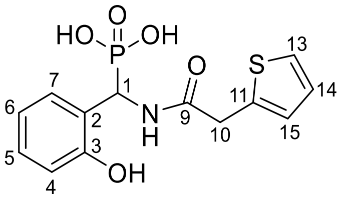

The synthesis was performed according to general procedure B, compound **4i** (56 mg, 0.1 mmol, in 7 mL toluene) gave the product as a pale orange solid (28 mg, 0.09 mmol, 61%). HRESIMS (*m/z*): 328.0419 $[M+H]^+$ (328.0408 calcd. for $C_{13}H_{14}NO_5PS$). ^1H NMR (500 MHz, D_2O) δ 7.30 (d, J = 5.1 Hz, 1H, 13-H), 7.24 (d, J = 7.6 Hz, 1H, 7-H), 7.19 (dd, J = 8.1, 7.5 Hz, 1H, 5-H), 6.99 (dd, J = 5.1, 3.4 Hz, 1H, 14-H), 6.96 (d, J = 3.4 Hz, 1H, 15-H), 6.92 (dd, J = 7.6, 7.5 Hz, 1H, 6-H), 6.88 (d, J = 8.1 Hz, 1H, 4-H), 5.39 (d, $^2J_{HP}$ = 20.4 Hz, 1H, 1-H), 3.92 (d, $^2J_{HH}$ = 15.9 Hz, 1H, 10-H), 3.83 (d, $^2J_{HH}$ = 15.9 Hz, 1H, 10-H). ^{13}C NMR (126 MHz, D_2O) δ 172.8 (d, $^3J_{CP}$ = 8.3 Hz, 9-C=O), 153.4 (d, $^3J_{CP}$ = 4.9 Hz, 3-C), 135.9 (11-C), 129.0 (d, $^5J_{CP}$ = 2.0 Hz, 5-CH), 128.4 (d, $^3J_{CP}$ = 4.6 Hz, 7-CH), 127.2 (14-CH or 15-CH), 127.2 (14-CH or 15-CH), 125.6 (13-CH), 124.0 (d, $^2J_{CP}$ = 1.6 Hz, 2-C), 120.8 (d, $^4J_{CP}$ = 1.6 Hz, 6-CH), 116.7 (d, $^4J_{CP}$ = 1.3 Hz, 4-CH), 48.3 (d, $^1J_{CP}$ = 145.0 Hz, 1-CH), 36.3 (10-CH_2). ^{31}P NMR (162 MHz, D_2O) δ 16.6.

((2-Hydroxyphenyl)(2-(thiophen-3-yl)acetamido)methyl)phosphonic acid (5j)

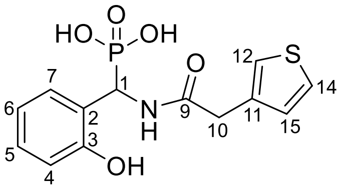

The synthesis was performed according to general procedure B, compound **4j** (87 mg, 0.2 mmol, in 11 mL toluene) gave the product as a white solid (52 mg, 0.2 mmol, 73%). HRESIMS (*m/z*): 328.0418 $[M+H]^+$ (328.0408 calcd. for $C_{13}H_{14}NO_5PS$). ^1H NMR (500 MHz, D_2O) δ 7.39 (dd, J = 4.9, 3.0 Hz, 1H, 14-H), 7.22 (d, J = 7.7 Hz, 1H, 7-H), 7.21 (d, J = 3.0 Hz, 1H, 12-H), 7.19 (dd, J = 8.1, 7.7 Hz, 1H, 5-H), 7.00 (d, J = 4.9 Hz, 1H, 15-H), 6.92 (dd, J = 7.7, 7.7 Hz, 1H, 6-H), 6.88 (d, J = 8.1 Hz, 1H, 4-H), 5.38 (d, $^2J_{HP}$ = 20.4 Hz, 1H, 1-H), 3.72 (d, $^2J_{HH}$ = 15.4 Hz, 1H, 10-H), 3.65 (d, $^2J_{HH}$ = 15.4 Hz, 1H, 10-H). ^{13}C NMR (126 MHz, D_2O) δ 173.6 (d, $^3J_{CP}$ = 8.5 Hz, 9-C=O), 153.4 (d, $^3J_{CP}$ = 4.8 Hz, 3-C), 134.3 (11-C), 129.0 (d, $^5J_{CP}$ = 1.7 Hz, 5-CH), 128.4 (d, $^3J_{CP}$ = 4.8 Hz, 7-CH), 128.2 (15-CH), 126.6 (14-CH), 124.1 (d, $^2J_{CP}$ = 1.8 Hz, 2-C), 123.4 (12-CH), 120.8 (d, $^4J_{CP}$ = 1.7 Hz, 6-CH), 116.7 (d, $^4J_{CP}$ = 1.4 Hz, 4-CH), 48.3 (d, $^1J_{CP}$ = 144.5 Hz, 1-CH), 36.8 (10-CH_2). ^{31}P NMR (162 MHz, D_2O) δ 16.6.

((2-(Benzo[*b*]thiophen-2-yl)acetamido)(2-hydroxyphenyl)methyl)phosphonic acid (5k)

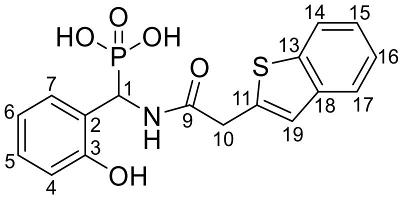

The synthesis was performed according to general procedure B, compound **4k** (150 mg, 0.3 mmol, in 16 mL toluene) gave the product as a pale orange solid (91 mg, 0.2 mmol, 72%). HRESIMS (*m/z*): 378.0562 $[M+H]^+$ (378.0565 calcd. for $C_{17}H_{16}NO_5PS$). ^{1}H NMR (601 MHz, D_2O) δ 7.87 (d, J = 7.6 Hz, 1H, 14-H), 7.79 (d, J = 7.6 Hz, 1H, 17-H), 7.40 (t, J = 7.6 Hz, 1H, 16-H), 7.36 (t, J = 7.6 Hz, 1H, 15-H), 7.27 (dd, J = 7.7, 1.8 Hz, 1H, 7-H), 7.24 (s, 1H, 19-H), 7.20 (ddd, J = 8.4, 7.7, 1.8 Hz, 1H, 5-H), 6.91 (dd, J = 7.7, 7.7 Hz, 1H, 6-H), 6.89 (d, J = 8.4 Hz, 1H, 4-H), 5.46 (d, $^2J_{HP}$ = 20.5 Hz, 1H, 1-H), 4.03 (d, $^2J_{HH}$ = 15.7 Hz, 1H, 10-H), 3.94 (d, $^2J_{HH}$ = 15.7 Hz, 1H, 10-H). ^{13}C NMR (151 MHz, D_2O) δ 172.2 (d, $^3J_{CP}$ = 8.4 Hz, 9-C=O), 153.5 (d, $^3J_{CP}$ = 4.1 Hz, 3-C), 139.7 (13-C), 139.6 (18-C), 137.5 (11-C), 129.1 (d, $^5J_{CP}$ = 2.0 Hz, 5-CH), 128.5 (d, $^3J_{CP}$ = 4.6 Hz, 7-CH), 124.6 (16-CH), 124.3 (15-CH), 124.0 (d, $^2J_{CP}$ = 1.9 Hz, 2-C), 123.8 (19-CH), 123.3 (17-CH), 122.3 (14-CH), 120.8 (d, $^4J_{CP}$ = 1.4 Hz, 6-CH), 116.7 (d, $^4J_{CP}$ = 1.6 Hz, 4-CH), 48.4 (d, $^1J_{CP}$ = 145.4 Hz, 1-CH), 37.3 (10-CH_2). ^{31}P NMR (162 MHz, D_2O) δ 16.6.

((2-(Benzo[*b*]thiophen-3-yl)acetamido)(2-hydroxyphenyl)methyl)phosphonic acid (5l)

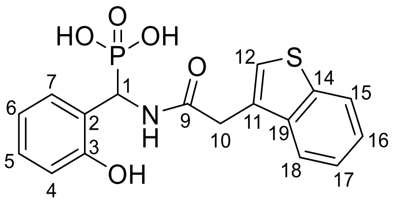

The synthesis was performed according to general procedure B, compound **4l** (102 mg, 0.2 mmol, in 12 mL toluene) gave the product as a pale yellow solid (74 mg, 0.2 mmol, 86%). HRESIMS (*m/z*): 378.0575 $[M+H]^+$ (378.0565 calcd. for $C_{17}H_{16}NO_5PS$). ^{1}H NMR (400 MHz, D_2O) δ 7.98 (d, J = 8.0 Hz, 1H, 15-H), 7.68 (d, J = 8.0 Hz, 1H, 18-H), 7.49 (s, 1H, 12-H), 7.42 (t, J = 8.0 Hz, 1H, 16-H), 7.37 (t, J = 8.0 Hz, 1H, 17-H), 7.19 (dd, J = 8.0, 7.5 Hz, 1H, 5-H), 7.17 (d, J = 7.5 Hz, 1H, 7-H), 6.88 (dd, J = 7.5, 7.5 Hz, 1H, 6-H), 6.86 (d, J = 8.0 Hz, 1H, 4-H), 5.44 (d, $^2J_{HP}$ = 20.5 Hz, 1H, 1-H), 4.02 (d, $^2J_{HH}$ = 16.1 Hz, 1H, 10-H), 3.93 (d, $^2J_{HH}$ = 16.1 Hz, 1H, 10-H). ^{13}C NMR (151 MHz, D_2O) δ 173.0 (d, $^3J_{CP}$ = 7.6 Hz, 9-C=O), 153.5 (d, $^3J_{CP}$ = 4.1 Hz, 3-C), 140.0 (14-C), 138.1 (19-C), 129.0 (d, $^5J_{CP}$ = 2.4 Hz, 5-CH), 128.9 (11-C), 128.7 (d, $^3J_{CP}$ = 4.8 Hz, 7-CH), 125.8 (12-CH), 124.6 (16-CH), 124.4 (16-CH), 124.0 (d, $^2J_{CP}$ = 1.6 Hz, 2-C), 123.0 (15-CH), 121.6 (18-CH), 120.7 (d, $^4J_{CP}$ = 1.5 Hz, 6-CH), 116.8 (d, $^4J_{CP}$ = 1.8 Hz, 4-CH), 48.6 (d, $^1J_{CP}$ = 144.8 Hz, 1-CH), 35.5 (10-CH_2). ^{31}P NMR (162 MHz, D_2O) δ 16.5.

((2-Hydroxyphenyl)(2-phenylacetamido)methyl)phosphonic acid (5m)

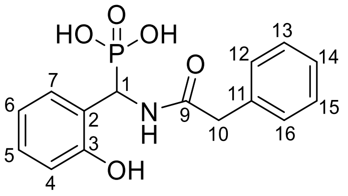

The synthesis was performed according to general procedure B, compound **4m** (100 mg, 0.3 mmol, in 12 mL toluene) gave the product as a white solid (60 mg, 0.2 mmol, 73%). HRESIMS (*m/z*): 322.0852 $[M+H]^+$ (322.0849 calcd. for $C_{15}H_{16}NO_5P$). ^{1}H NMR (500 MHz, D_2O) δ 7.38 – 7.31 (m, 2H, 13-H and 15-H), 7.30 (m, 1H, 14-H), 7.29 – 7.24 (m, 2H, 12-H and 16-H), 7.23 (d, J = 7.6 Hz, 1H, 7-H), 7.18 (ddd, J = 8.0, 7.6 Hz, 1H, 5-H), 6.91 (dd, J = 7.6, 7.6 Hz, 1H, 6-H), 6.87 (d, J = 8.0 Hz, 1H, 4-H), 5.41 (d, $^2J_{HP}$ = 20.5 Hz, 1H, 1-H), 3.70 (d, $^2J_{HH}$ = 15.0 Hz, 1H, 10-H), 3.63 (d, $^2J_{HH}$ = 15.0 Hz, 1H, 10-H). ^{13}C NMR (126 MHz, D_2O) δ 174.0 (d, $^3J_{CP}$ = 8.2 Hz, 9-C=O), 153.4 (d, $^3J_{CP}$ = 4.9 Hz, 3-C), 134.8 (11-C), 129.1 (2 x CH, 12-CH and 16-CH), 129.0 (d, $^5J_{CP}$ = 2.4 Hz, 5-CH), 128.8 (2 x CH, 13-CH and 15-CH), 128.4 (d, $^3J_{CP}$ = 4.8 Hz, 7-CH), 127.2 (14-CH), 124.1 (d, $^2J_{CP}$ = 1.9 Hz, 2-C), 120.8 (d, $^4J_{CP}$ = 2.0 Hz, 6-CH), 116.7 (d, $^4J_{CP}$ = 2.0 Hz, 4-CH), 48.2 (d, $^1J_{CP}$ = 145.1 Hz, 1-CH), 42.2 (10-CH_2). ^{31}P NMR (162 MHz, D_2O) δ 16.8.

3. Analytical purity of the final compounds

The analytical purity of the final compounds was measured using LC-MS (Agilent 1260 Infinity II) equipped with an ESI-Q detector (Infinitylab LC-MSD) using Agilent InfinityLab Poroshell 120 C18 columns (2.7 µm, ϕ 2.1 mm L 50 mm) at 254 nm with an 8 min long gradient method (5% to 95% CH_3CN) of CH_3CN/H_2O + 0.1% formic acid as the mobile phase at a 0.6 mL/min flow rate or with a VWR LaChrome ELITE analytical HPLC system using a Kinetex C18 column (5 µm, 100 Å, ϕ 3.0 mm L 150 mm) at 254 nm with a 20 min long gradient method (5% to 95% CH_3CN) of CH_3CN/H_2O + 0.1% formic acid as the mobile phase at a 1.0 mL/min flow rate. The main peaks (> 95%) and impurities with > 1% are indicated in the tables.

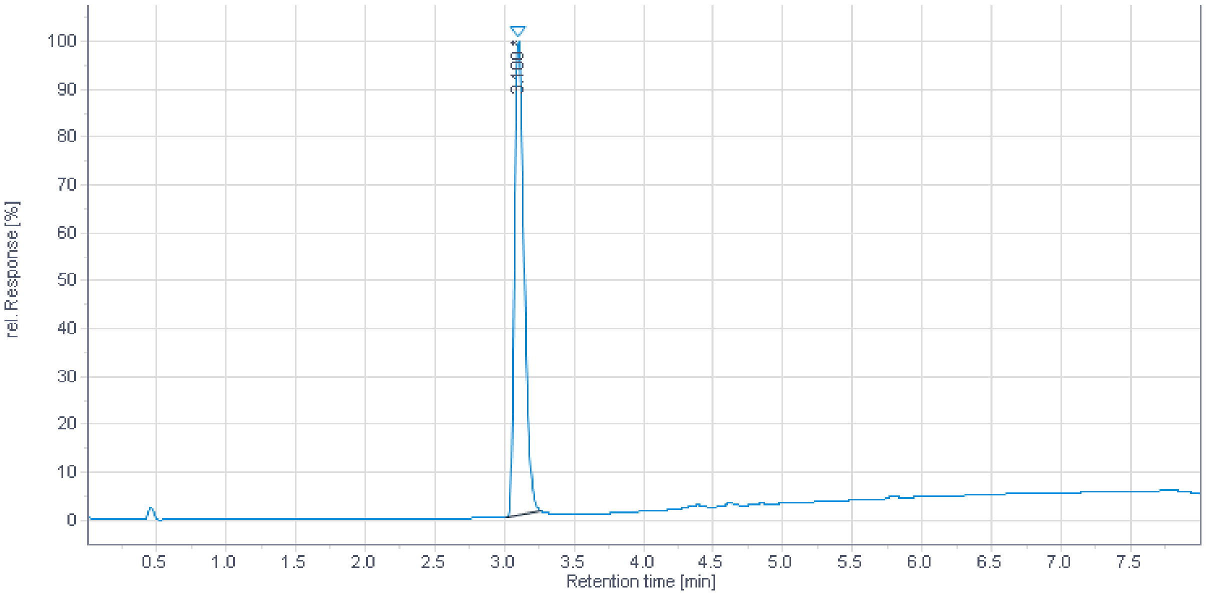

Peak Results

RT (min)	Area	Area%
0.461	13.9	1.11
3.100	1230.7	98.89

Figure S1. *Analytical HPLC UV-chromatogram signal of compound* ***5a*** *at 254 nm.*

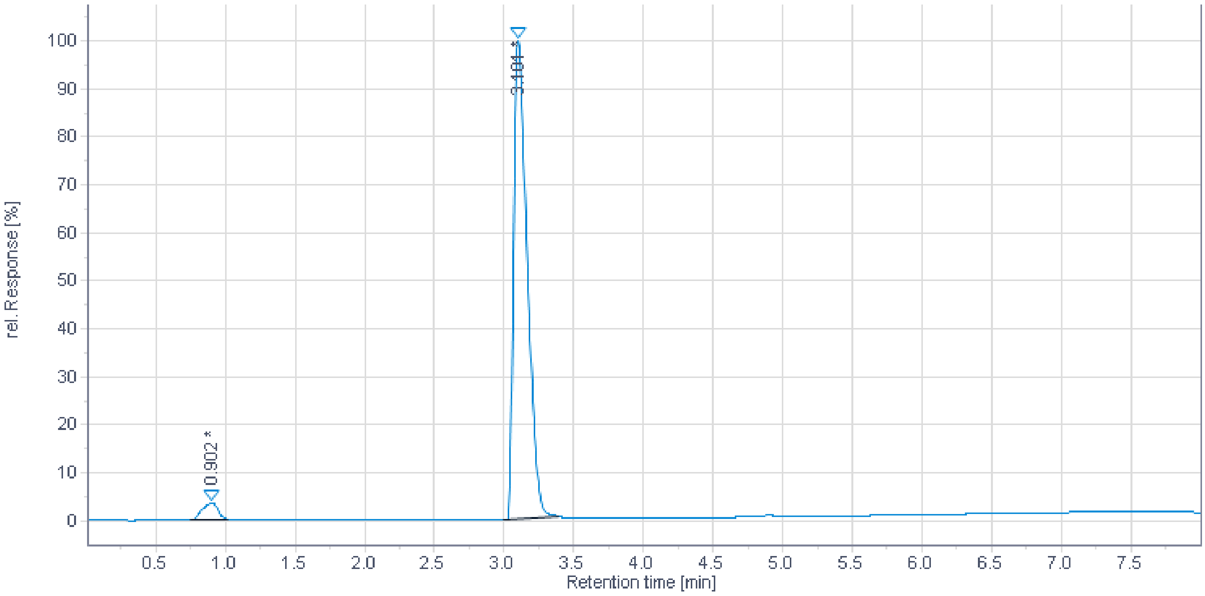

Peak Results

RT (min)	Area	Area%
0.902	175.4	3.82
3.101	4415.8	96.18

Figure S2. *Analytical HPLC UV-chromatogram signal of compound* ***5b*** *at 254 nm.*

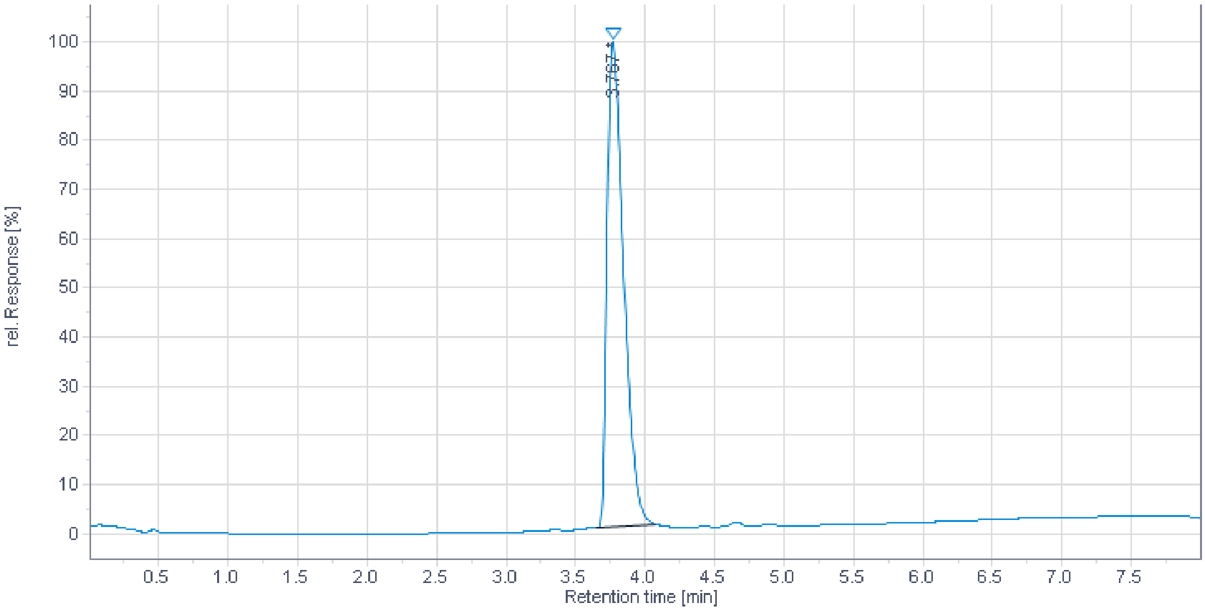

Peak Results

RT (min)	Area	Area%
3.767	2680.5	100.00

Figure S3. *Analytical HPLC UV-chromatogram signal of compound* ***5c*** *at 254 nm.*

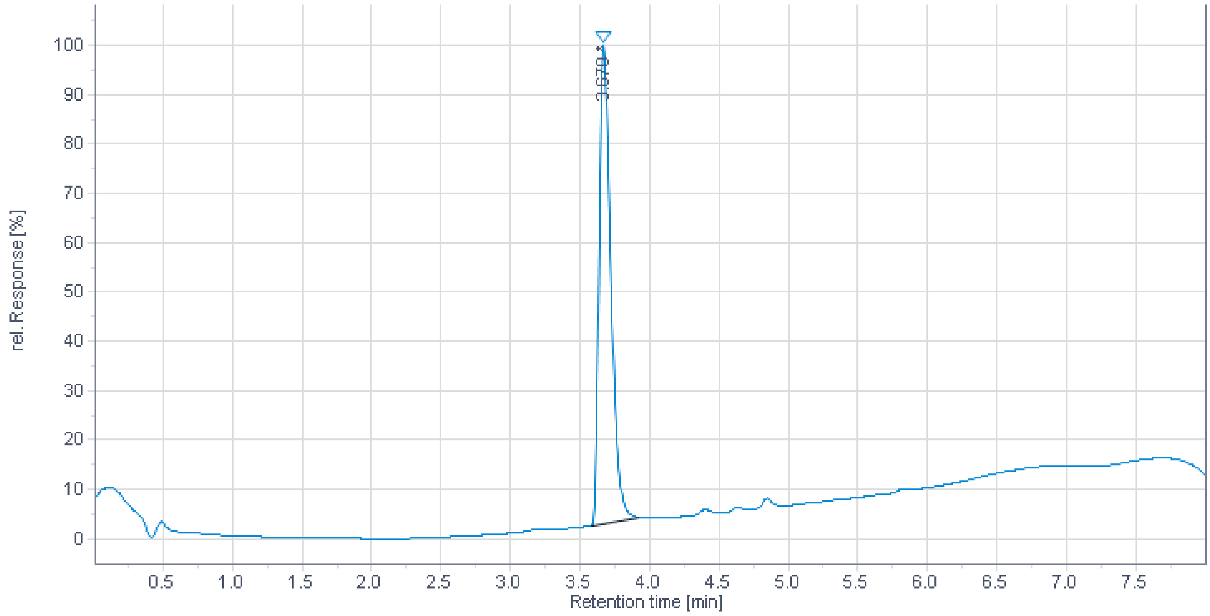

Peak Results

RT (min)	Area	Area%
3.670	394.2	98.27
4.846	4.6	1.15

Figure S4. *Analytical HPLC UV-chromatogram signal of compound* ***5d*** *at 254 nm.*

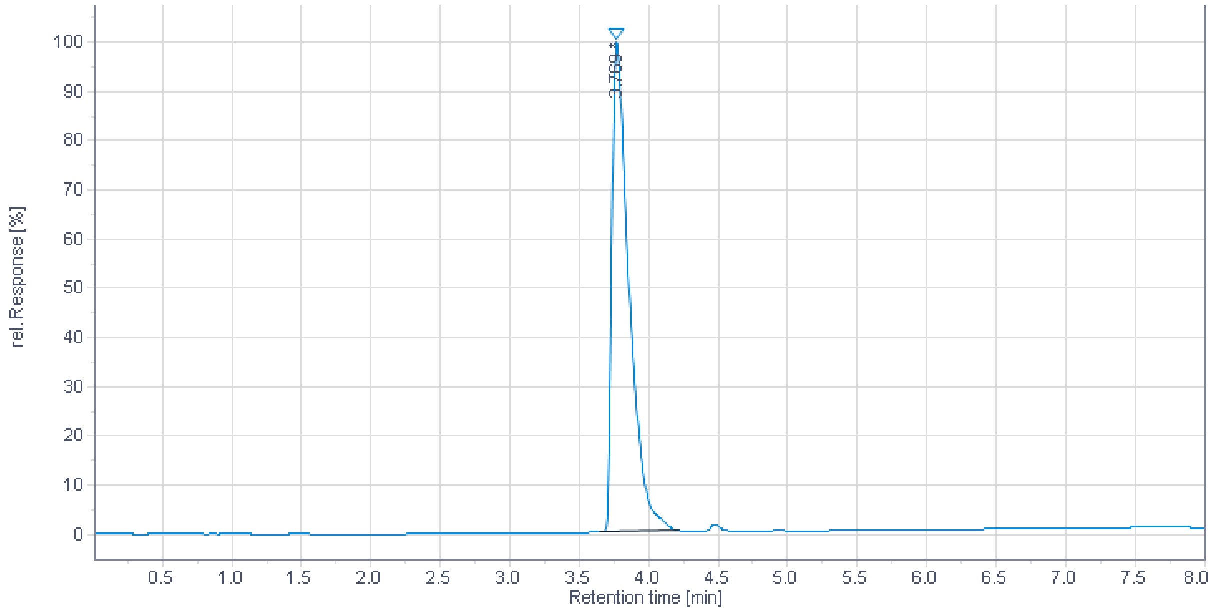

Peak Results

RT (min)	Area	Area%
3.769	7490.6	99.36

Figure S5. *Analytical HPLC UV-chromatogram signal of compound* ***5e*** *at 254 nm.*

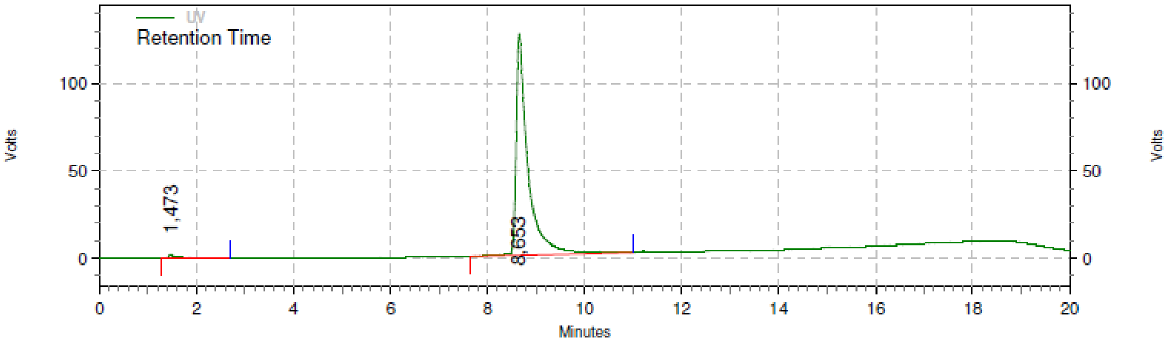

Peak Results

RT (min)	Area	Area%
1.473	105293	1.20
8.653	8683296	98.80

Figure S6. *Analytical HPLC UV-chromatogram signal of compound* ***5f*** *at 254 nm.*

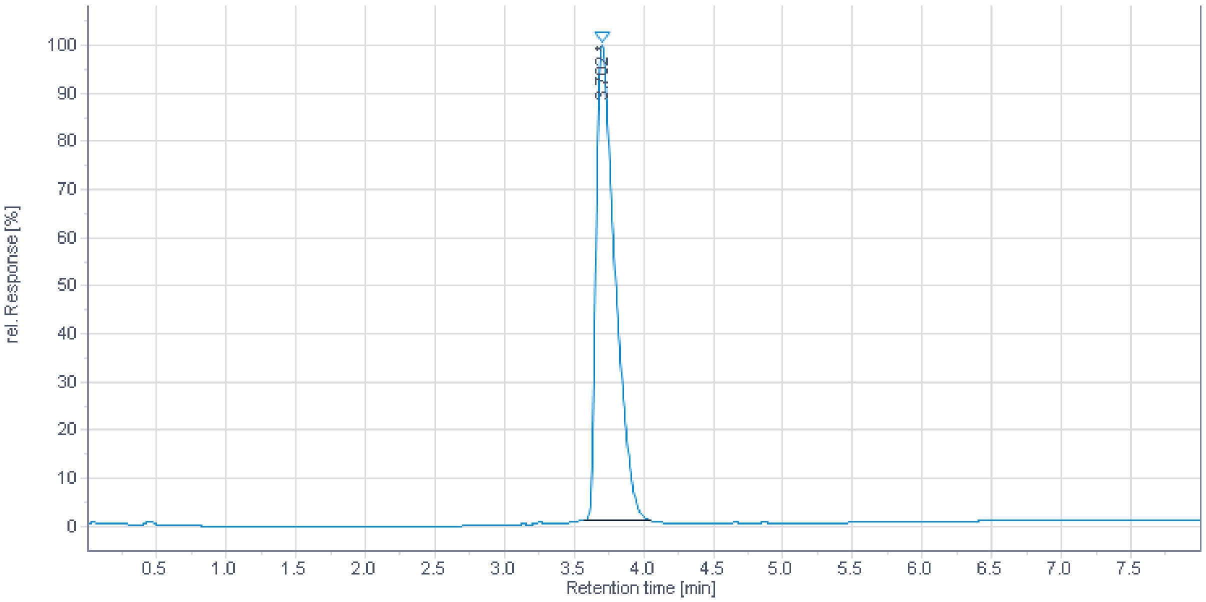

Peak Results

RT (min)	Area	Area%
3.702	7919.4	99.62

Figure S7. *Analytical HPLC UV-chromatogram signal of compound* ***5g*** *at 254 nm.*

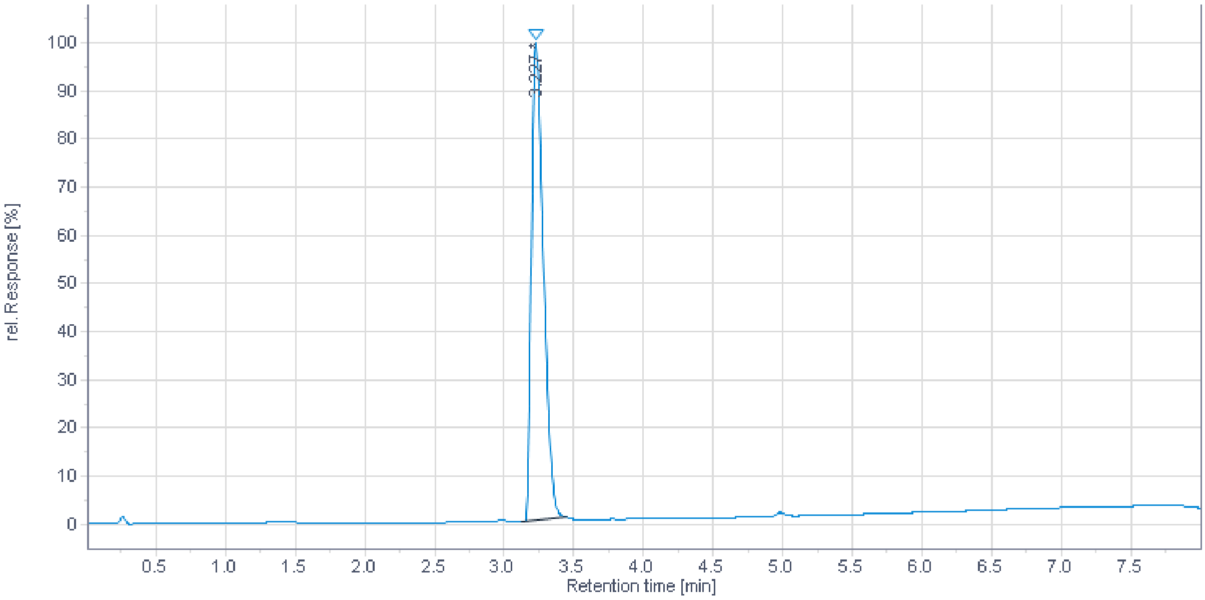

Peak Results

RT (min)	Area	Area%
3.227	2084.0	98.94

Figure S8. *Analytical HPLC UV-chromatogram signal of compound* ***5h*** *at 254 nm.*

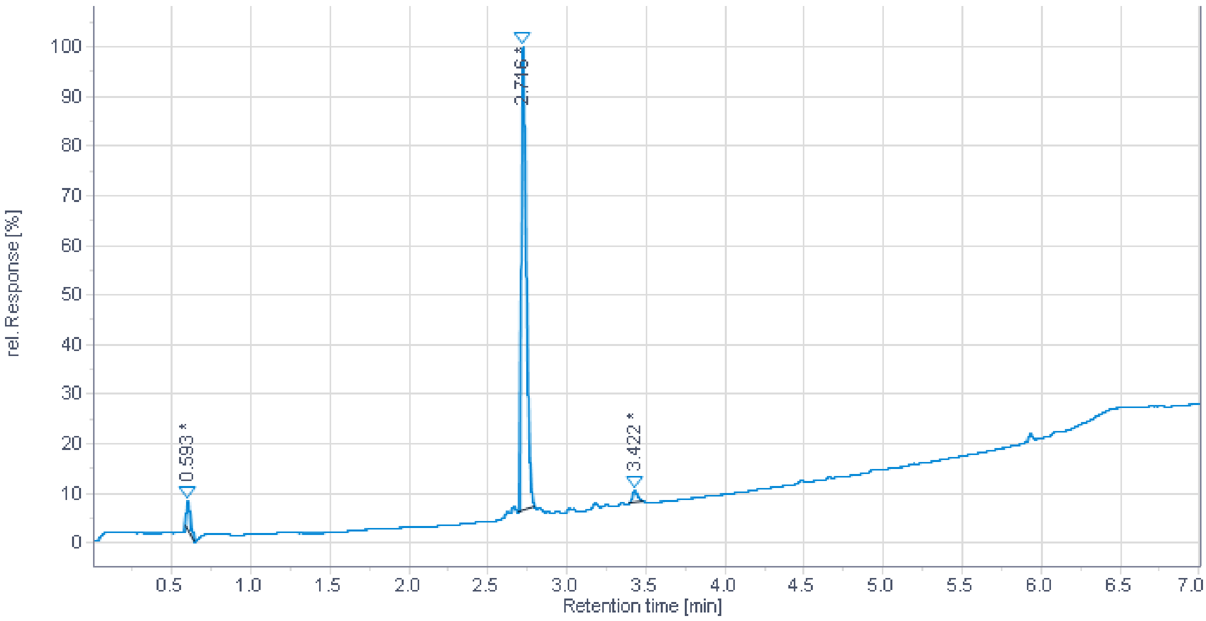

Peak Results

RT (min)	Area	Area%
0.593	3.2	2.75
2.716	111.8	95.16
3.422	2.5	2.09

Figure S9. *Analytical HPLC UV-chromatogram signal of compound* ***5i*** *at 254 nm.*

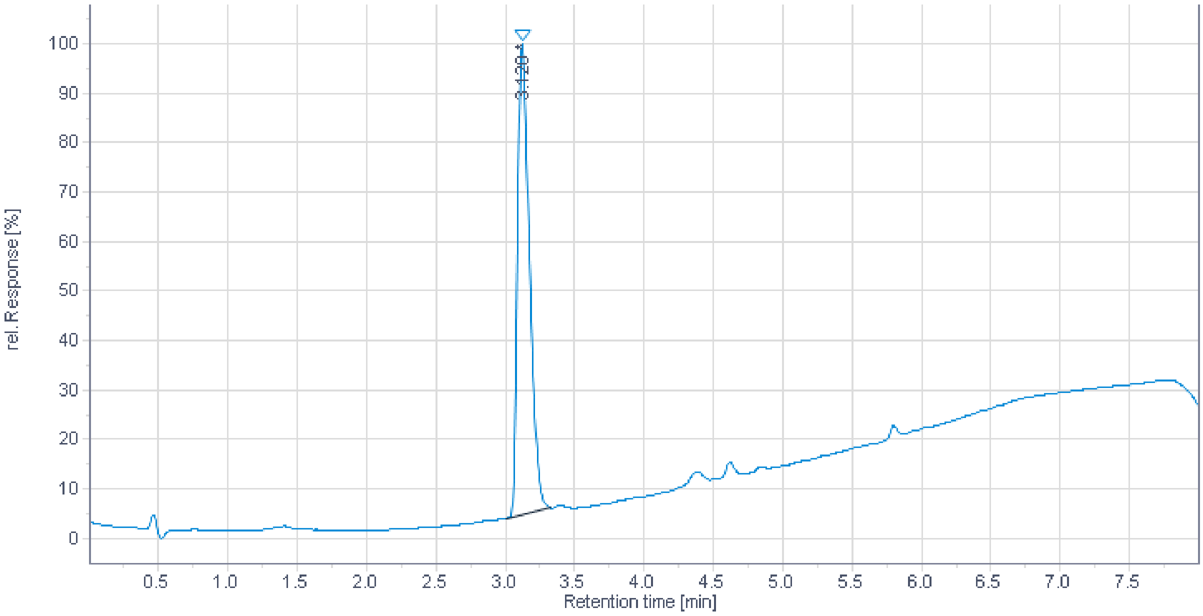

Peak Results

RT (min)	Area	Area%
3.120	282.0	95.77
4.386	5.3	1.80
4.621	4.0	1.36
5.800	3.2	1.07

Figure S10. *Analytical HPLC UV-chromatogram signal of compound* ***5j*** *at 254 nm.*

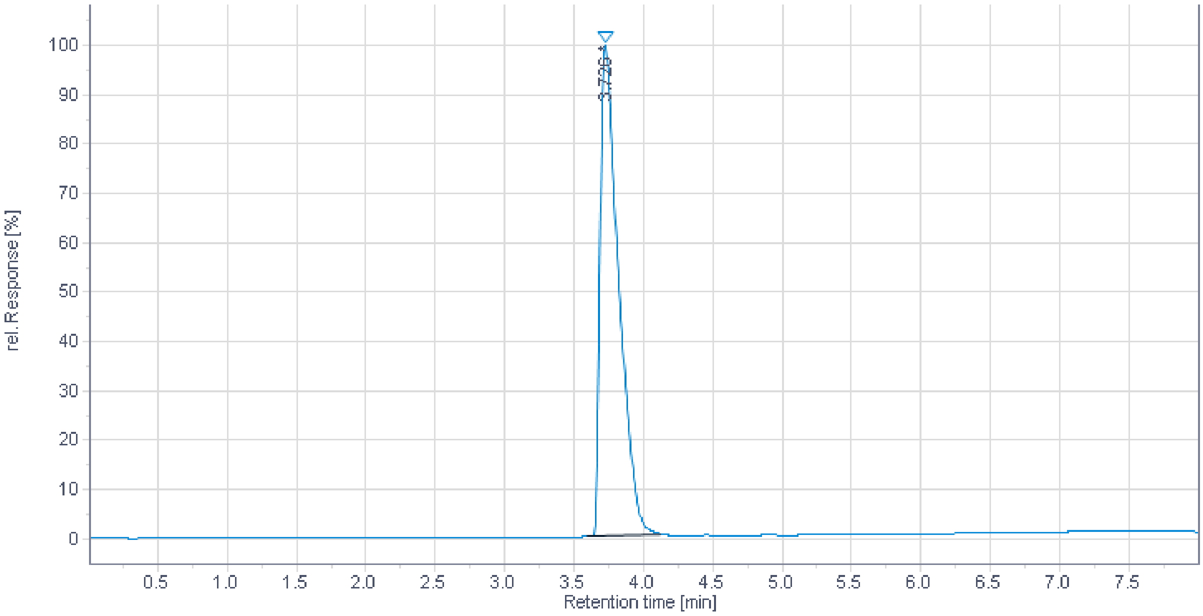

Peak Results

RT (min)	Area	Area%
3.726	7506.4	100.00

Figure S11. *Analytical HPLC UV-chromatogram signal of compound* ***5k*** *at 254 nm.*

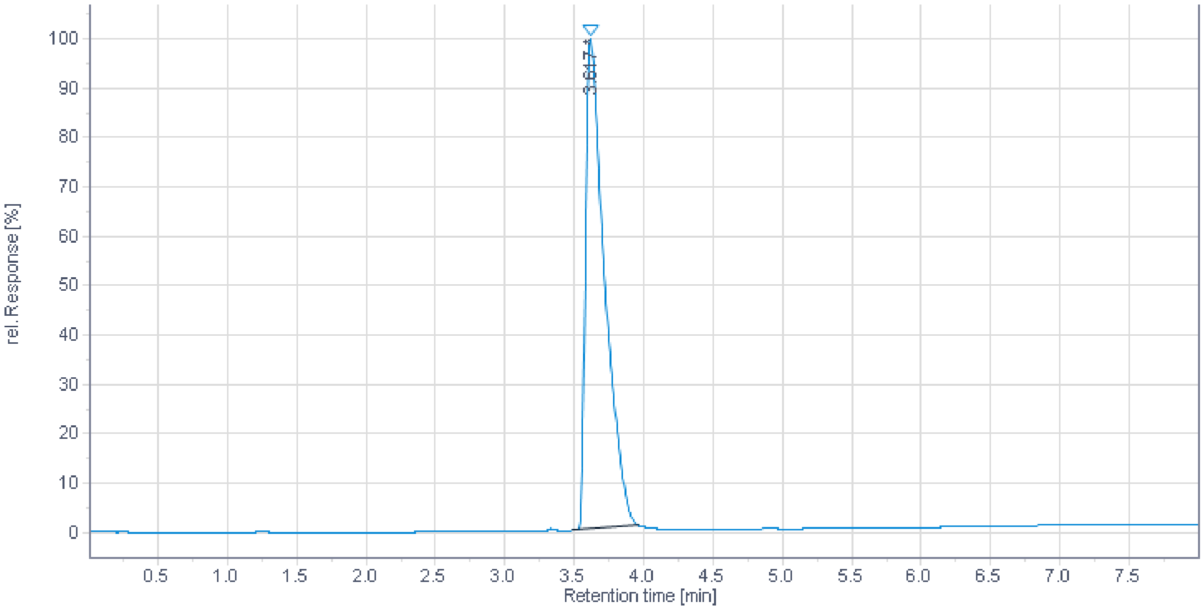

Peak Results

RT (min)	Area	Area%
3.617	7314.5	100.00

Figure S12. *Analytical HPLC UV-chromatogram signal of compound* ***5l*** *at 254 nm.*

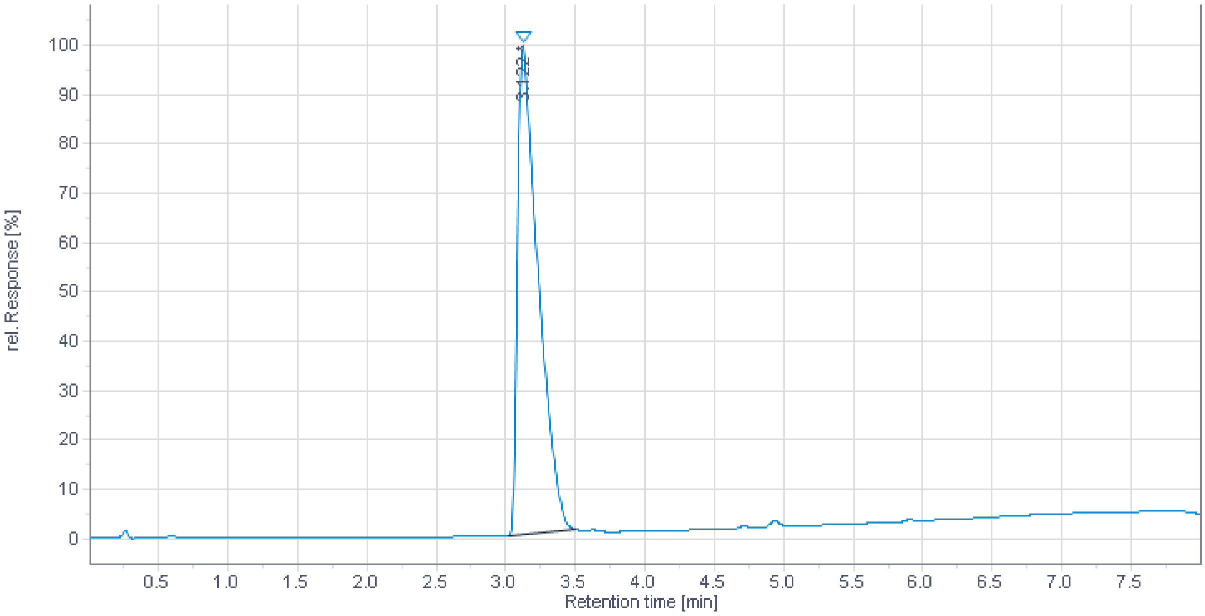

Peak Results

RT (min)	Area	Area%
3.122	2140.1	99.28

Figure S13. *Analytical HPLC UV-chromatogram signal of compound* ***5m*** *at 254 nm.*

4. Long term stability of the final compounds in buffer with 2.5% DMSO

The long term stability of the final compounds in buffer (20 mM KPO_4, 0.1 mM $ZnCl_2$, pH 7.0 + 2.5% DMSO) were monitored using LC-MS (Agilent 1260 Infinity II) equipped with an ESI-Q detector (Infinitylab LC-MSD) using Agilent InfinityLab Poroshell 120 C18 column (2.7 µm, ϕ 4.6 mm L 50 mm) at 254 nm with a 7 min long gradient method (5% to 95% CH_3CN) of CH_3CN/H_2O + 0.1% formic acid as the mobile phase at a 1.0 mL/min flow rate. The samples were measured right after dissolving them in buffer, and after 1 month of keeping the same samples dissolved in the same buffer. The main peaks (> 95%) and impurities with > 1% are indicated in the tables.

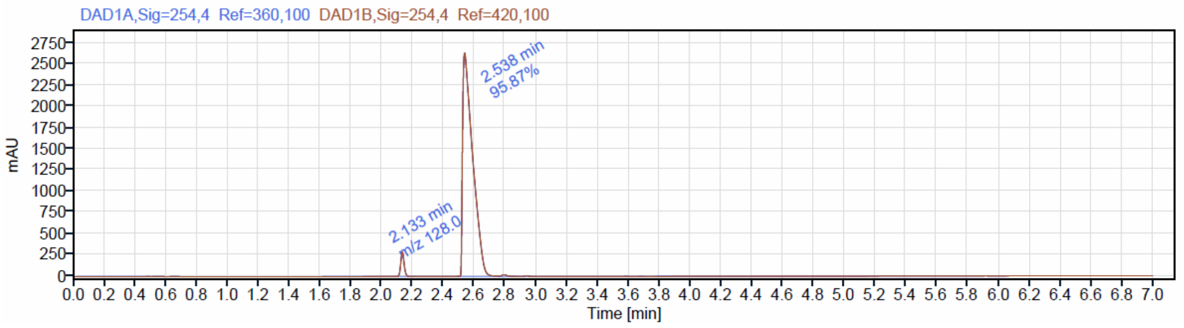

Peak Results

RT (min)	Area	Area%
2.133	444.9	3.54
2.538	12053.5	95.87

Figure S14. *Compound **5a** right after dissolving it in buffer.*

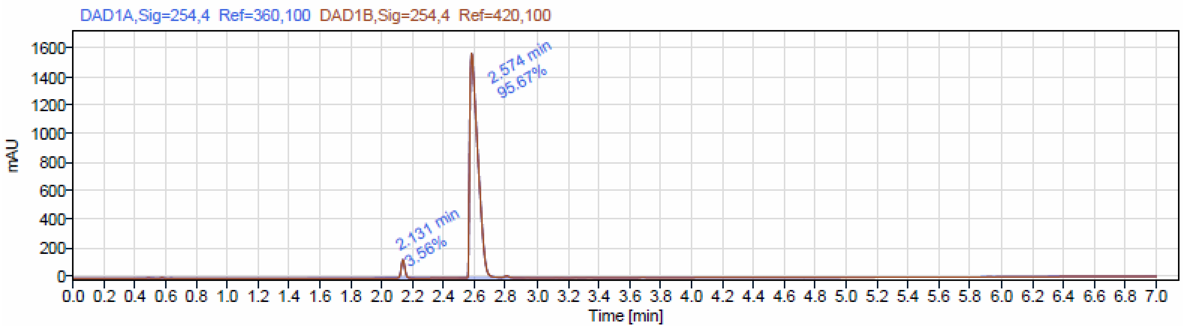

Peak Results

RT (min)	Area	Area%
2.131	213.4	3.56
2.574	5740.7	95.67

Figure S15. *Compound **5a** kept in buffer for 1 month.*

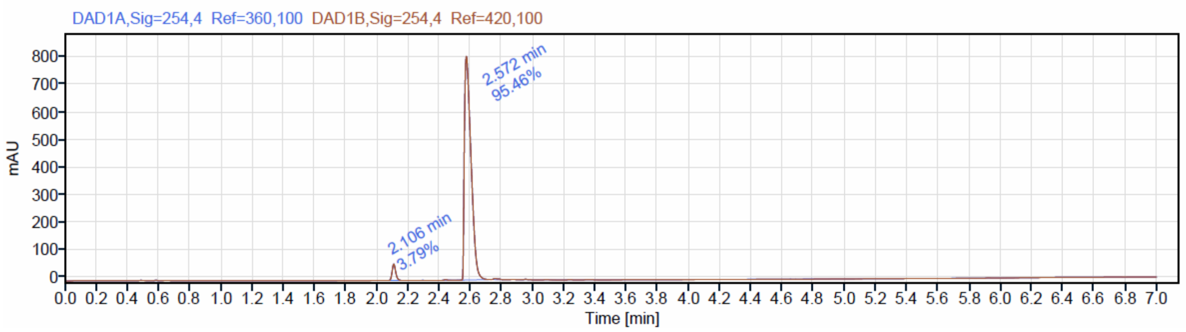

Peak Results

RT (min)	Area	Area%
2.106	95.7	3.79
2.572	2409.9	95.46

***Figure S16**. Compound **5b** right after dissolving it in buffer.*

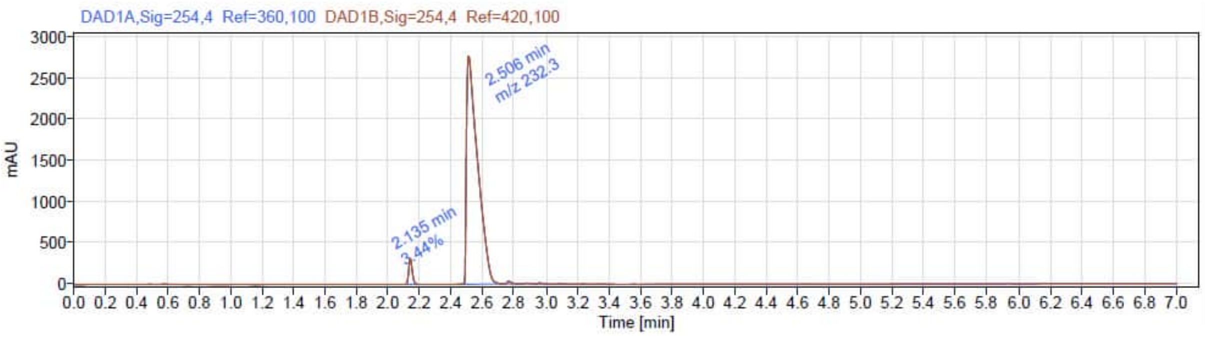

Peak Results

RT (min)	Area	Area%
2.135	498.6	3.44
2.506	13771.8	95.03

***Figure S17**. Compound **5b** kept in buffer for 1 month.*

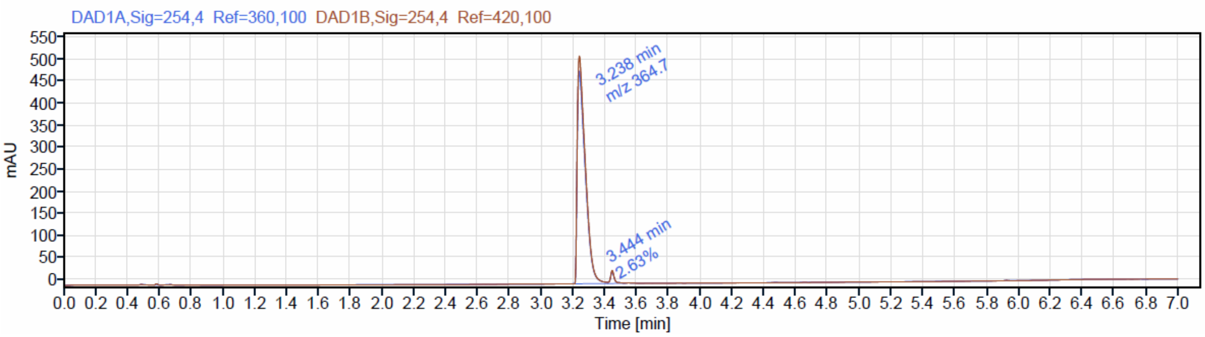

Peak Results

RT (min)	Area	Area%
3.238	1716.3	97.20
3.444	46.5	2.63

***Figure S18**. Compound **5c** right after dissolving it in buffer.*

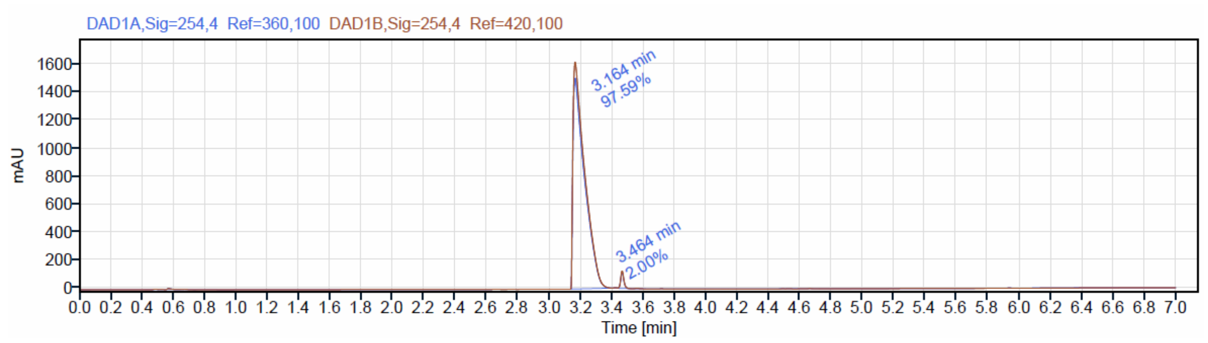

Peak Results

RT (min)	Area	Area%
3.164	7890.5	97.59
3.464	161.8	2.00

***Figure S19**. Compound **5c** kept in buffer for 1 month.*

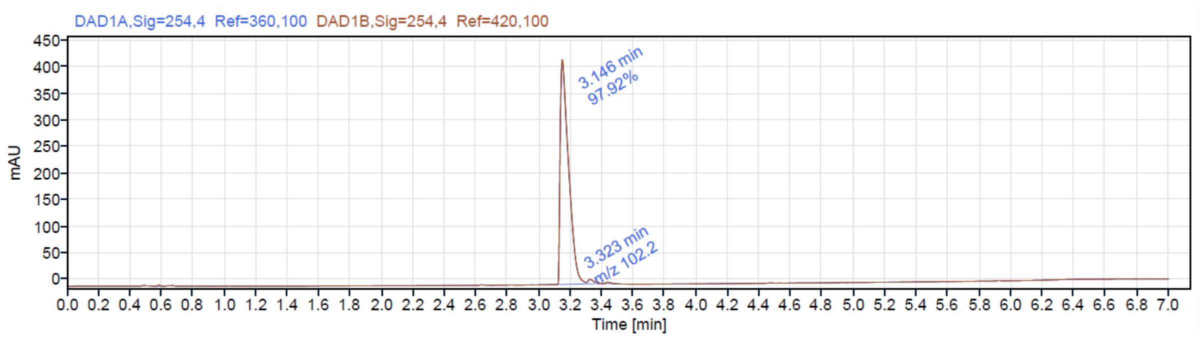

Peak Results

RT (min)	Area	Area%
3.146	1551.9	97.92
3.323	24.5	1.55

***Figure S20**. Compound **5d** right after dissolving it in buffer.*

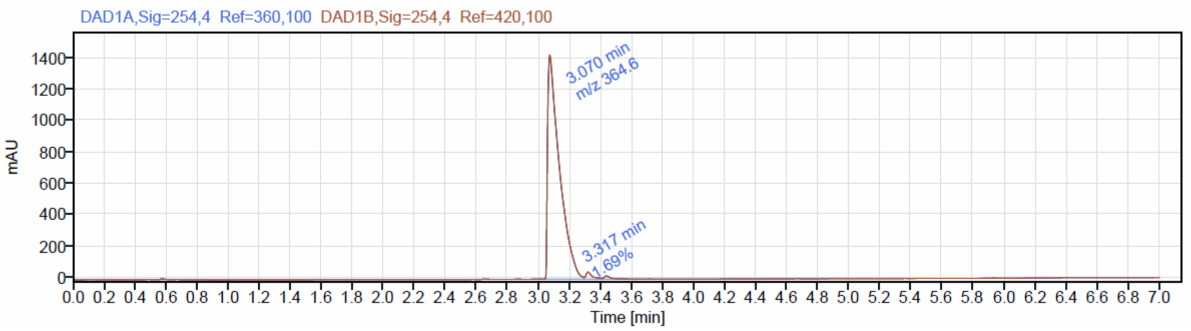

Peak Results

RT (min)	Area	Area%
3.070	7496.1	97.15
3.317	130.6	1.69

***Figure S21**. Compound **5d** kept in buffer for 1 month.*

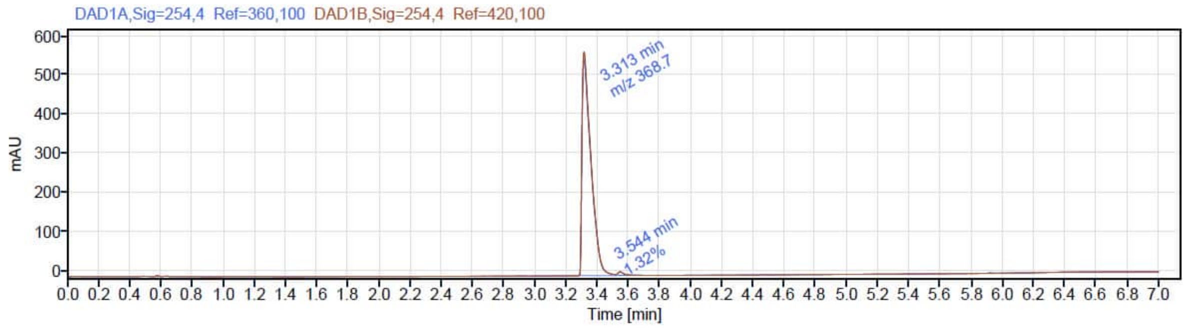

Peak Results

RT (min)	Area	Area%
3.313	2229.8	98.53
3.544	29.8	1.32

Figure S22. *Compound* **5e** *right after dissolving it in buffer.*

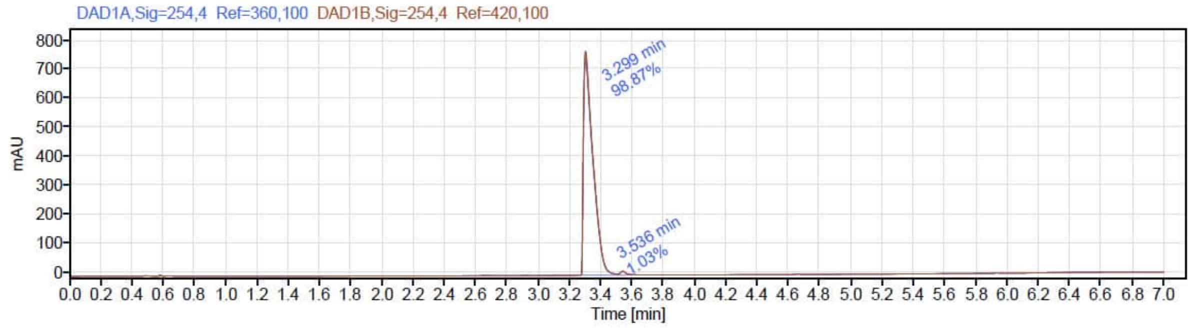

Peak Results

RT (min)	Area	Area%
3.299	3200.1	98.87
3.536	33.4	1.03

Figure S23. *Compound* **5e** *kept in buffer for 1 month.*

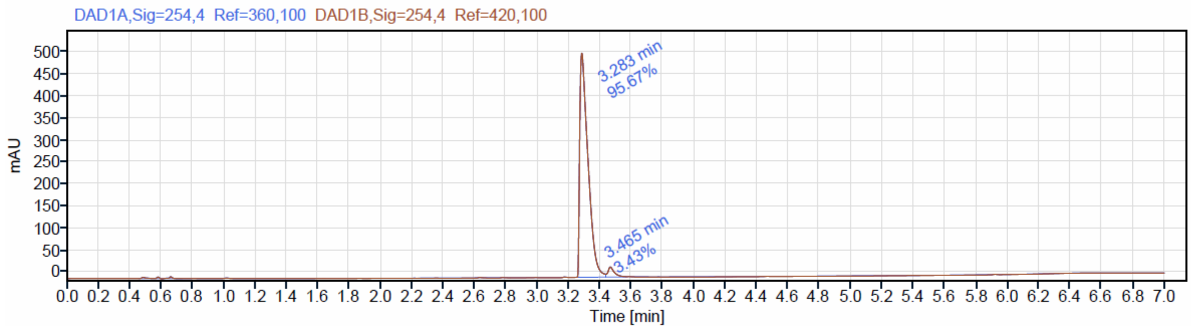

Peak Results

RT (min)	Area	Area%
3.283	1877.9	95.67
3.465	67.3	3.43

Figure S24. *Compound* **5f** *right after dissolving it in buffer.*

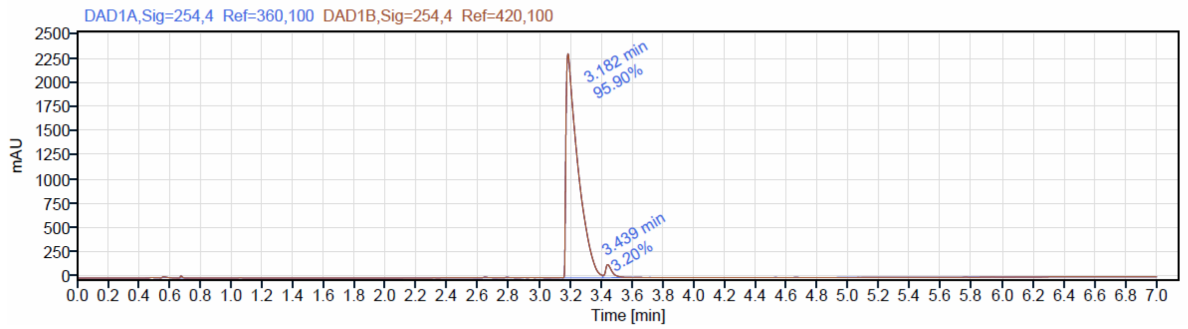

Peak Results

RT (min)	Area	Area%
3.182	12466.9	95.90
3.439	416.2	3.20

Figure S25. *Compound* ***5f*** *kept in buffer for 1 month.*

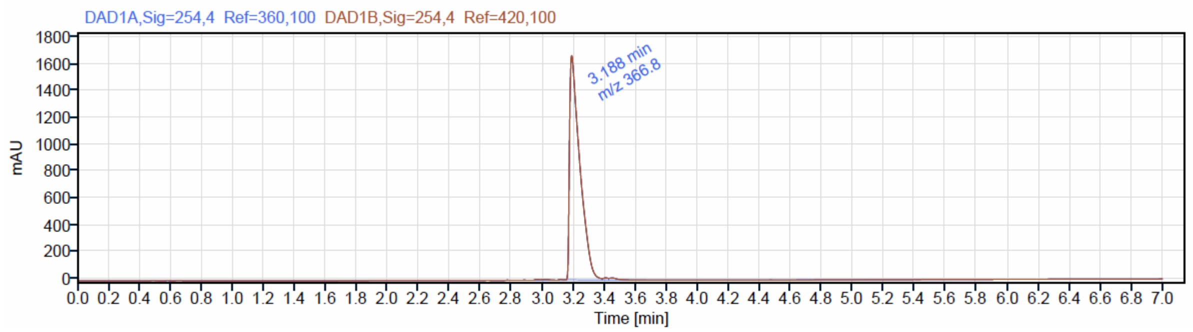

Peak Results

RT (min)	Area	Area%
3.188	7702.3	98.21

Figure S26. *Compound* ***5g*** *right after dissolving it in buffer.*

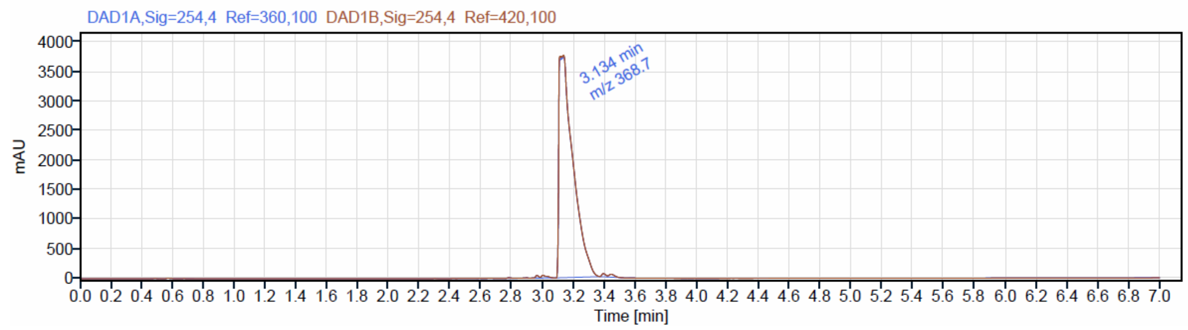

Peak Results

RT (min)	Area	Area%
3.134	24027.1	98.05

Figure S27. *Compound* ***5g*** *kept in buffer for 1 month.*

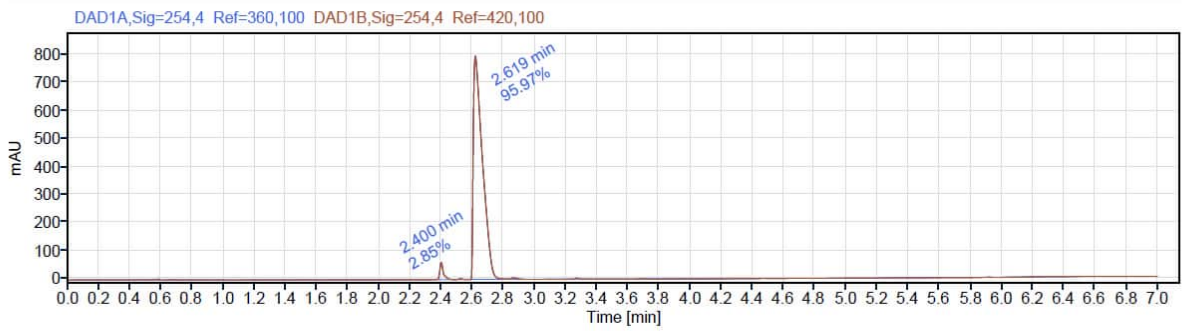

Peak Results

RT (min)	Area	Area%
2.400	97.0	2.85
2.619	3261.6	95.97

***Figure S28**. Compound **5h** right after dissolving it in buffer.*

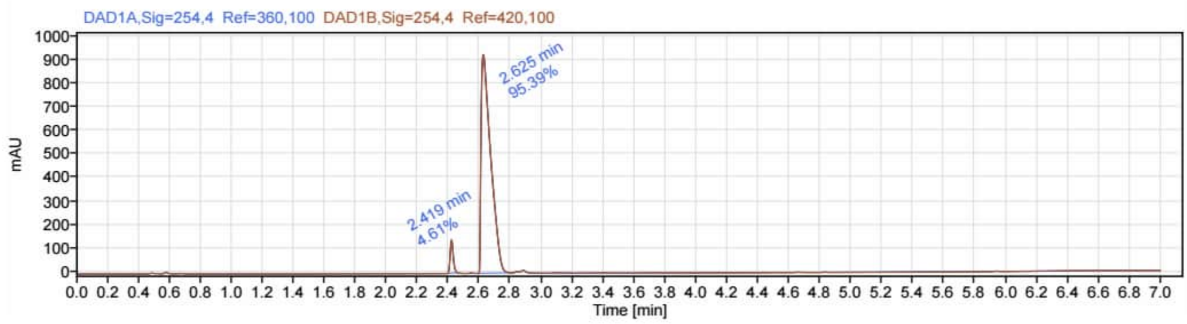

Peak Results

RT (min)	Area	Area%
2.419	194.3	4.61
2.625	4018.8	95.39

***Figure S29**. Compound **5h** kept in buffer for 1 month.*

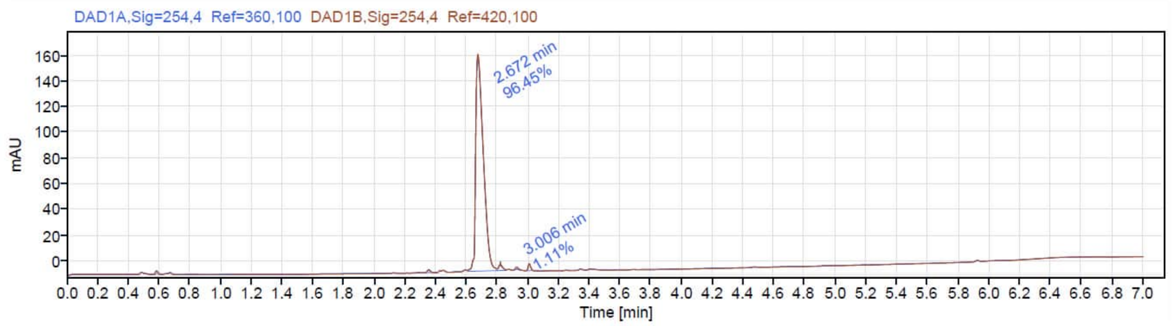

Peak Results

RT (min)	Area	Area%
2.672	576.9	96.45
3.006	6.7	1.11

***Figure S30**. Compound **5i** right after dissolving it in buffer.*

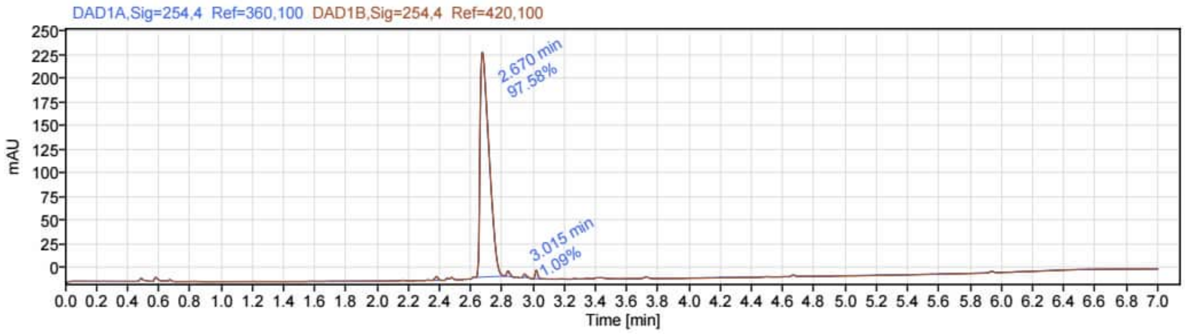

Peak Results

RT (min)	Area	Area%
2.670	942.4	97.58
3.015	10.5	1.09

Figure S31*. Compound* ***5i*** *kept in buffer for 1 month.*

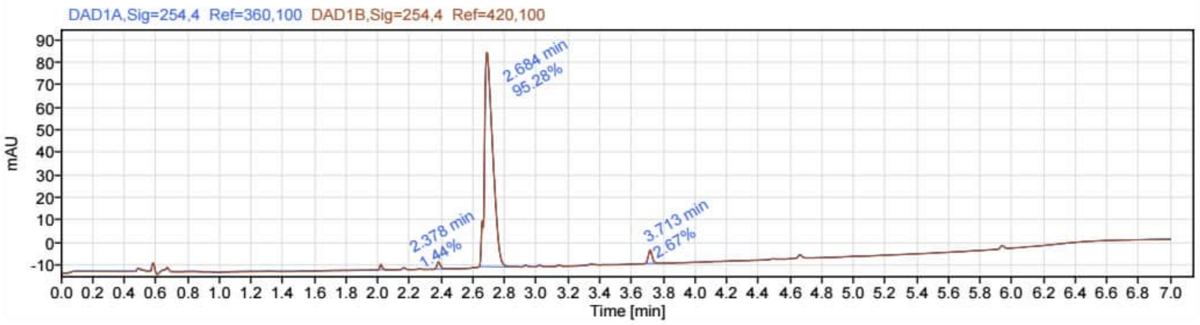

Peak Results

RT (min)	Area	Area%
2.378	5.2	1.44
2.684	346.0	95.28
3.713	9.7	2.67

Figure S32*. Compound* ***5j*** *right after dissolving it in buffer.*

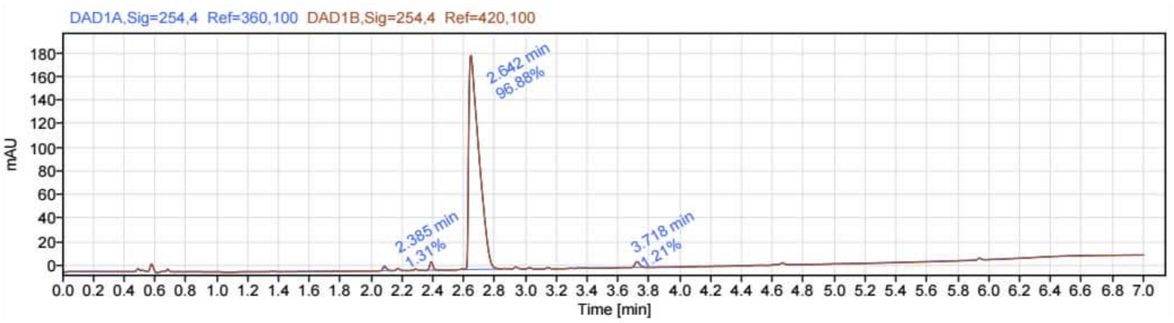

Peak Results

RT (min)	Area	Area%
2.385	11.3	1.31
2.642	832.0	96.88
3.718	10.4	1.21

***Figure S33**. Compound **5j** kept in buffer for 1 month.*

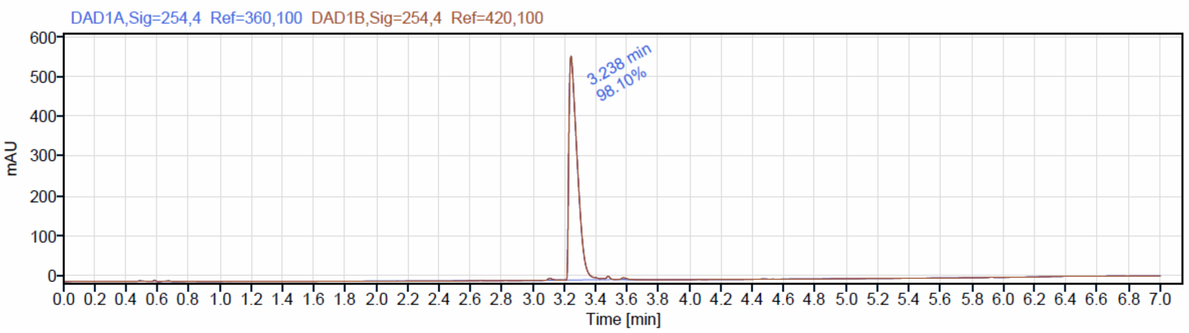

Peak Results

RT (min)	Area	Area%
3.238	2089.9	98.10

***Figure S34**. Compound **5k** right after dissolving it in buffer.*

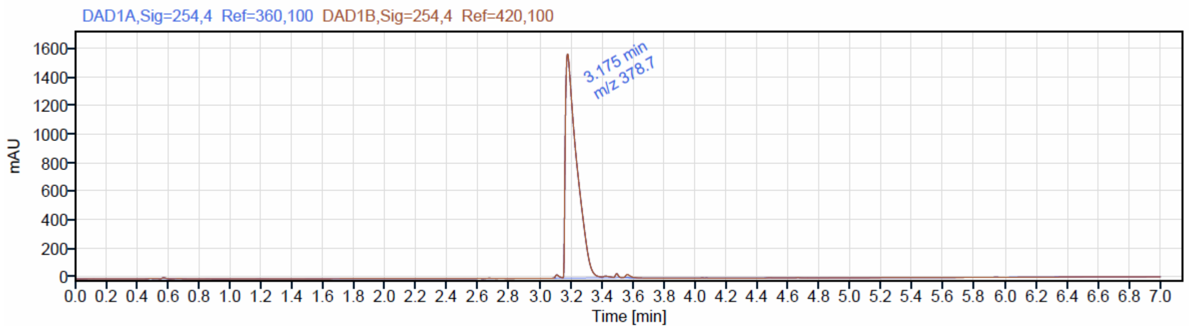

Peak Results

RT (min)	Area	Area%
3.175	8043.2	98.27

***Figure S35**. Compound **5k** kept in buffer for 1 month.*

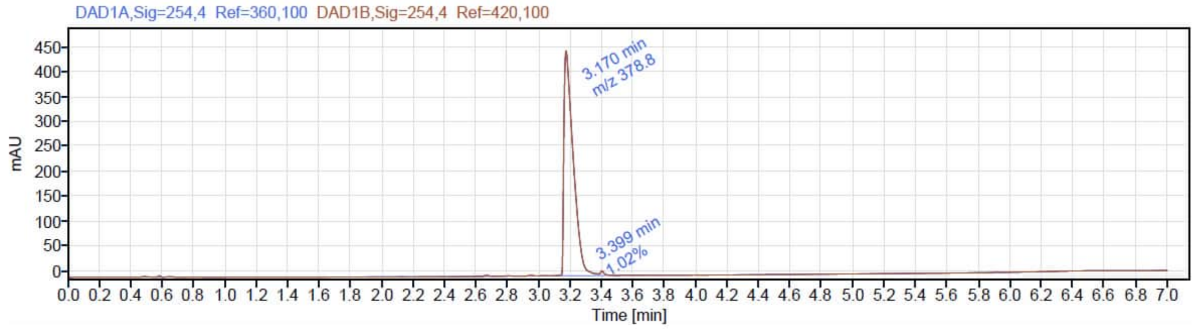

Peak Results

RT (min)	Area	Area%
3.170	1873.8	98.54
3.399	19.397	1.02

Figure S36. *Compound* **5l** *right after dissolving it in buffer.*

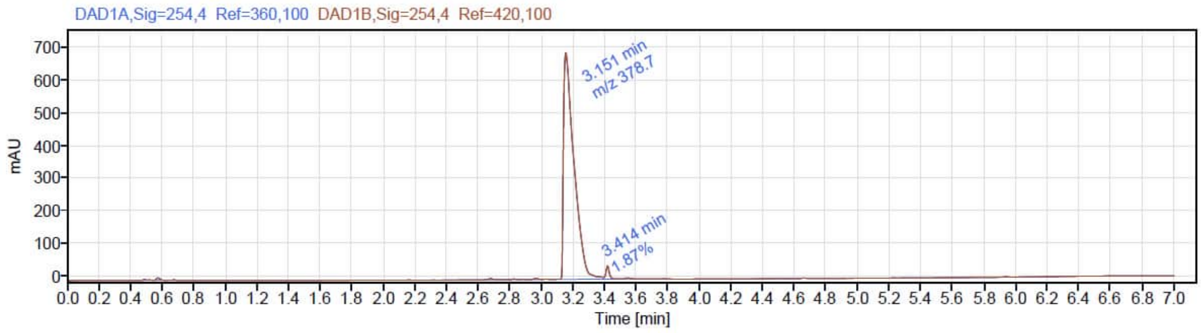

Peak Results

RT (min)	Area	Area%
3.151	3226.3	98.10
3.414	61.5	1.87

Figure S37. *Compound* **5l** *kept in buffer for 1 month.*

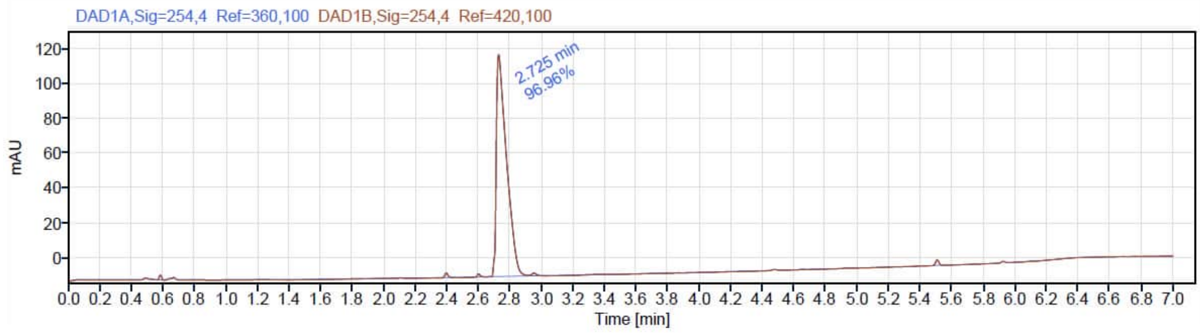

Peak Results

RT (min)	Area	Area%
2.725	575.1	96.96

Figure S38. *Compound* **5m** *right after dissolving it in buffer.*

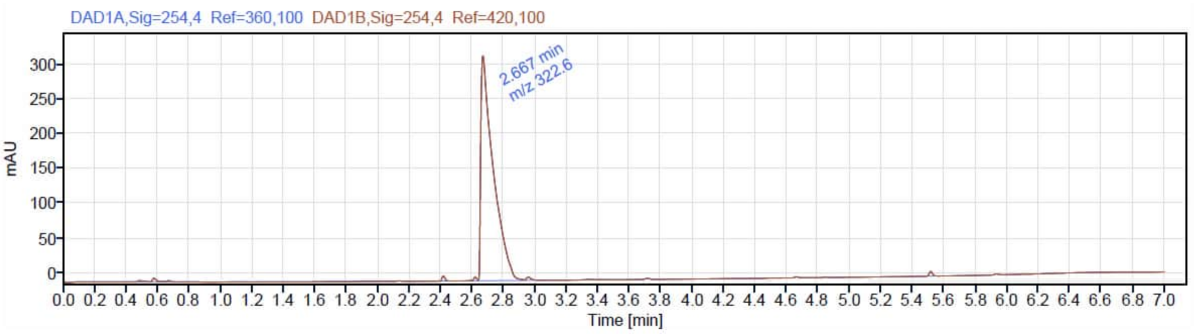

Peak Results

**RT (min)**	**Area**	**Area%**
2.667	1781.8	97.13

***Figure S39**. Compound **5m** kept in buffer for 1 month.*

5. Chiral HPLC

Samples of **5d** were dissolved to ~1 mg/mL in MeCN and ~1 mg/mL in IPA. These samples were then analysed by SFC and HPLC. Stationary phases such as IA, AmyC/AmyC Neo, A1, and SA were used during screening, but in all cases, no separation of the two enantiomers was observed. Normal phase HPLC separation was attempted using iso-hexane/ethanol & iso-hexane/isopropanol on the following columns: Lux Amylose 2, Lux i-Amylose 1, Lux Cellulose 1, Lux Cellulose 2 ,Lux Cellulose 3, Lux Cellulose 4, Lux i-Cellulose 5, YMC SA, Chiralpak IG, YMC Amy-C, Chiralpak IH, Chiralpak IA. Polar organic HPLC separation was attempted using methanol, ethanol, & MeCN/isopropanol on the following columns: Lux Amylose 1, Lux Cellulose 1, Lux Cellulose 2, Lux Cellulose 4, Lux i-Cellulose 5, Chiralpak IG and using MeCN/water on Amylose 1 column. SFC separation was attempted using methanol, ethanol and iso-propanol with basic modifier on the following columns: YMC AmyC-Neo, Lux Amylose 2, Lux Cellulose 1, Lux Cellulose 2, Lux Cellulose 3, Lux Cellulose 4, Lux i-Cellulose 5, Chiralpak IA, Chiralpak IG, Chiralpak IH, Chiralpak IJ. SFC separation was attempted using methanol, ethanol & acetonitrile with acidic modifier on the following columns: Lux Amylose 1, Lux Cellulose 1, Lux Cellulose 3, Lux Amylose 2, Lux i-Cellulose 5, Chiralpak IG.

The fact that the stereoisomers of **5d** could not be separated provides indirect evidence for it to be sterodynamic. X-ray diffraction has proven **5** to contain two enantiomers. We cannot exclude that the stereoisomers do not interconvert, although the lack of separation in the above extensive screening attempt suggest this be the case.

6. Metallo-β-lactamase inhibition (IC_{50}, K_i) on purified enzymes

Table S2. *Measured half-maximal inhibitory concentrations (IC_{50}) towards VIM-2, GIM-1 and NDM-1.*

**Inhibitors**	**IC_{50} (µM)**			***$logIC_{50}$* (µM)[1]**		
	**VIM-2**	**GIM-1**	**NDM-1**	**VIM-2**	**GIM-1**	**NDM-1**
**5a**	71	20	217	1.85 ± 0.07	1.30 ± 0.08	2.34 ± 0.09
**5b**	124	236	97	2.10 ± 0.08	2.37 ± 0.21	1.99 ± 0.13
**5c**	103	101	ND[2]	2.01 ± 0.05	2.01 ± 0.09	ND[2]
**5d**	21	10	ND[2]	1.33 ± 0.03	1.01 ± 0.19	ND[2]
**5e**	79	53	ND[2]	1.90 ± 0.11	1.73 ± 0.12	ND[2]
**5f**	48	52	30	1.68 ± 0.05	1.71 ± 0.10	1.48 ± 0.03
**5g**	12	21	ND[2]	1.06 ± 0.04	1.33 ± 0.11	ND[2]
**5h**	55	35	85	1.74 ± 0.05	1.54 ± 0.13	1.93 ± 0.09
**5i**	38	189	87	1.58 ± 0.08	2.28 ± 0.52	1.94 ± 0.08
**5j**	37	103	232	1.57 ± 0.03	2.01 ± 0.06	2.37 ± 0.07
**5k**	51	80	65	1.71 ± 0.07	1.90 ± 0.08	1.81 ± 0.11
**5l**	26	51	67	1.42 ± 0.18	1.71 ± 0.07	1.83 ± 0.05
**5m**	74	121	ND[2]	1.87 ± 0.35	2.08 ± 0.53	ND[2]

[1] *Errors are reported as the standard error of mean (SEM) of $logIC_{50}$*

[2] *ND = Not determined*

All data is calculated using GraphPad Prism v9.3.1

Table S3. *Estimated inhibition constants (K_i) for VIM-2, GIM-1 and NDM-1. The reported values are approximated from Dixon plots (1/v vs [I]) at one constant substrate concentration.*

**Inhibitors**	K_i (µM)[1]		
	**VIM-2**	**GIM-1**	**NDM-1**
**5a**	36 ± 6	144 ± 36	617 ± 95
**5b**	112 ± 11	162 ± 21	451 ± 57
**5c**	71 ± 6	102 ± 10	ND[2]
**5d**	31 ± 5	64 ± 13	ND
**5e**	72 ± 7	76 ± 5	ND
**5f**	40 ± 2	14 ± 84	128 ± 58
**5g**	43 ± 15	113 ± 66	ND
**5h**	85 ± 12	118 ± 20	363 ± 86
**5i**	76 ± 8	348 ± 28	1067 ± 360
**5j**	88 ± 16	ND	196 ± 13
**5k**	119 ± 13	189 ± 76	262 ± 41
**5l**	39 ± 5	193 ± 41	411 ± 160
**5m**	74 ± 7	213 ± 22	ND

[1] *Errors are reported as the standard error (SE)*

[2] *ND = Not determined*

All data is calculated using GraphPad Prism v9.3.1

Table S4. *The dose-response graphs for the enzymatic assay of the inhibitors* ***5a-m*** *against VIM-2 and GIM-1.*

Inhibitors	VIM-2	GIM-1
**5a**	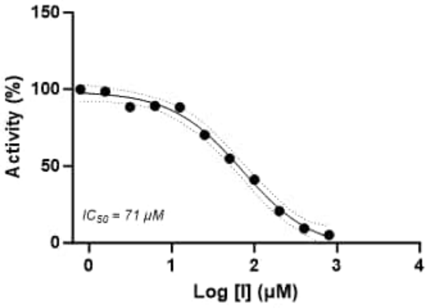	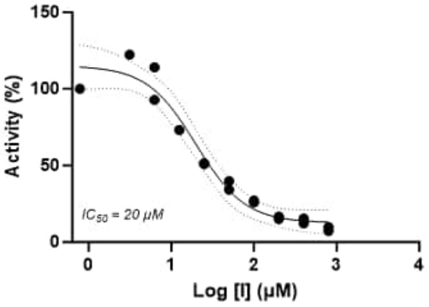
**5b**	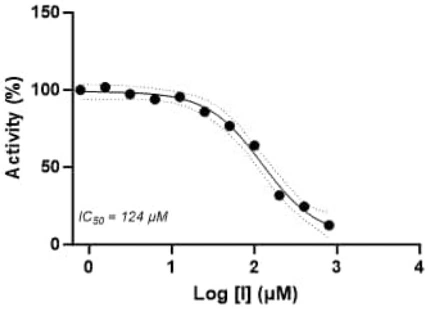	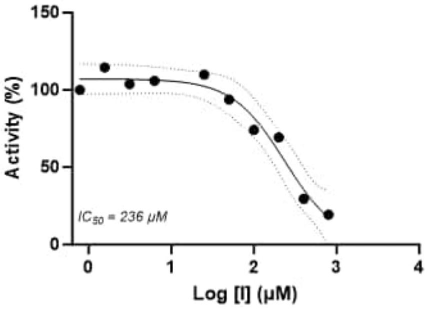
**5c**	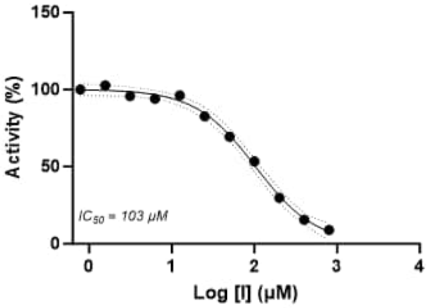	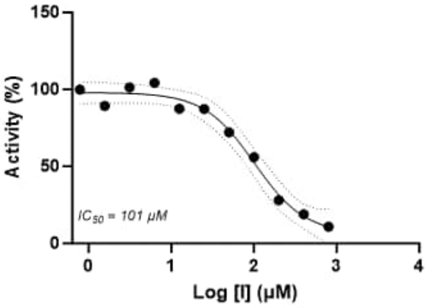
**5d**	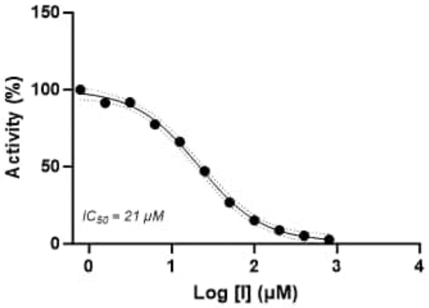	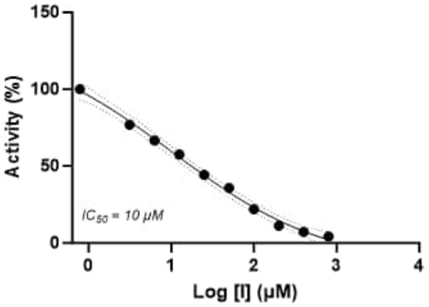
**5e**	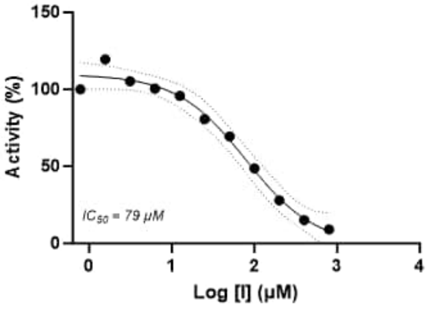	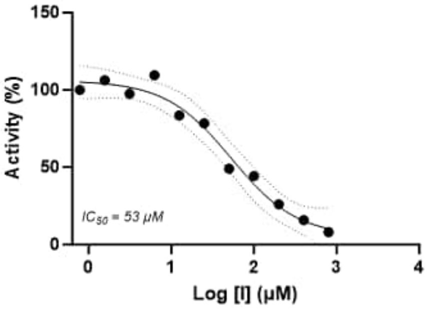

<table>
<tr><td>5f</td><td>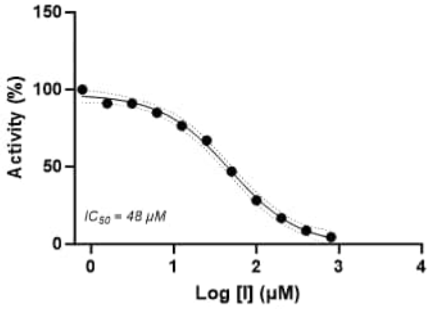
</td><td>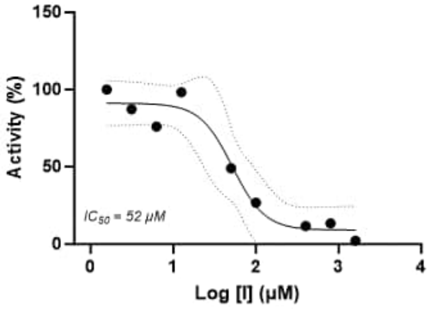
</td></tr>
<tr><td>5g</td><td>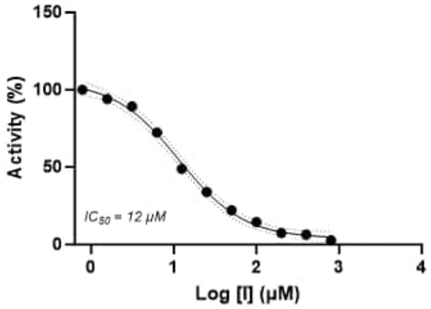
</td><td>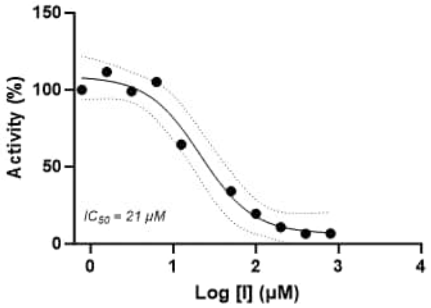
</td></tr>
<tr><td>5h</td><td>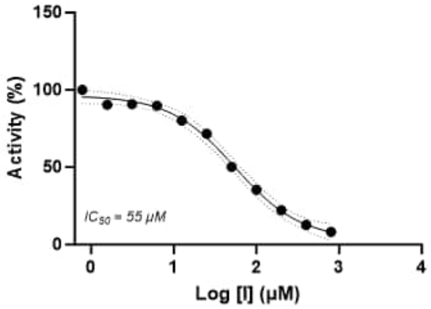
</td><td>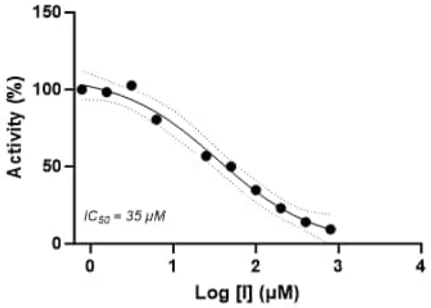
</td></tr>
<tr><td>5i</td><td>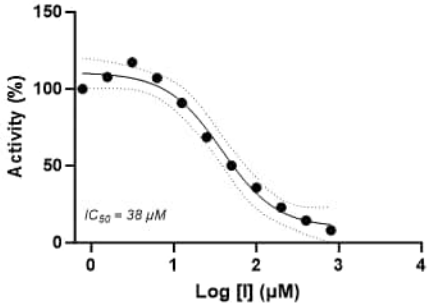
</td><td>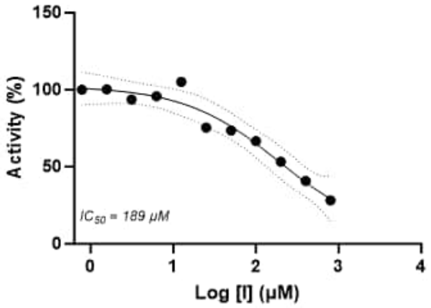
</td></tr>
<tr><td>5j</td><td>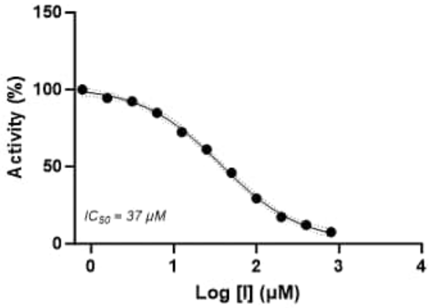
</td><td>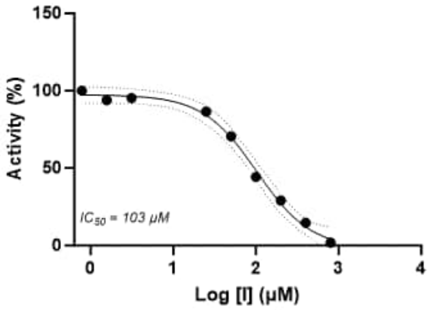
</td></tr>
</table>

**5k**	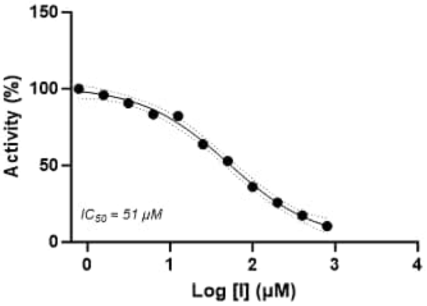 Activity (%) Log [I] (μM) IC_{50} = 51 μM	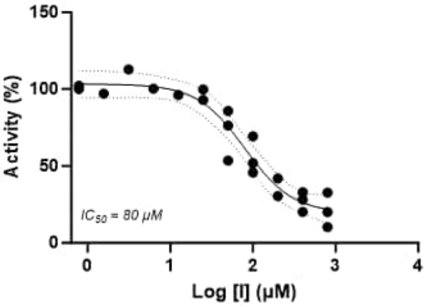 Activity (%) Log [I] (μM) IC_{50} = 80 μM
**5l**	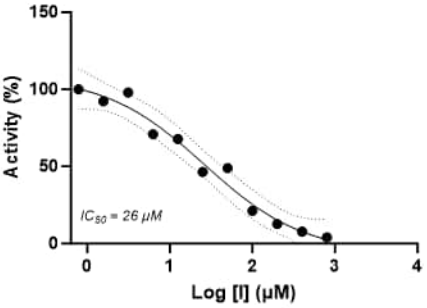 Activity (%) Log [I] (μM) IC_{50} = 26 μM	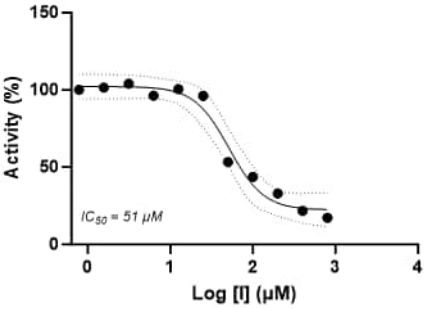 Activity (%) Log [I] (μM) IC_{50} = 51 μM
**5m**	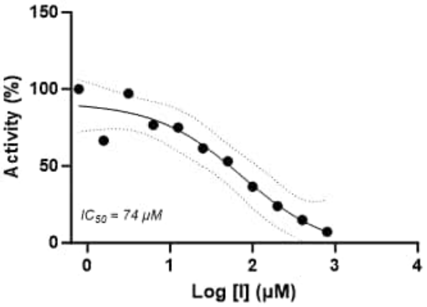 Activity (%) Log [I] (μM) IC_{50} = 74 μM	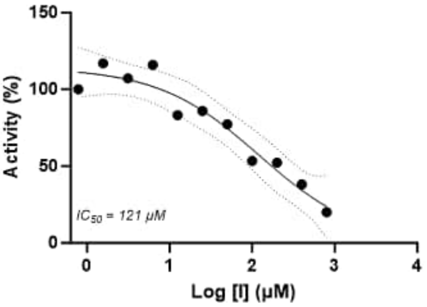 Activity (%) Log [I] (μM) IC_{50} = 121 μM

Table S5. *The dose-response graphs for the enzymatic assay of the inhibitors* ***5a-m*** *against NDM-1.*

<table>
<tr><th>Inhibitors</th><th>NDM-1</th></tr>
<tr><td>5a</td><td>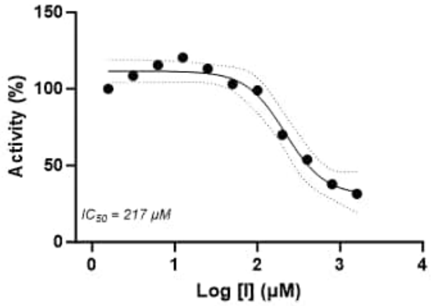
</td></tr>
<tr><td>5b</td><td>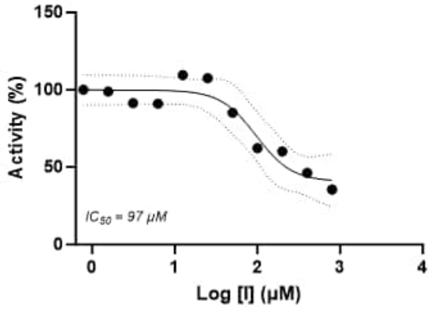
</td></tr>
<tr><td>5c</td><td>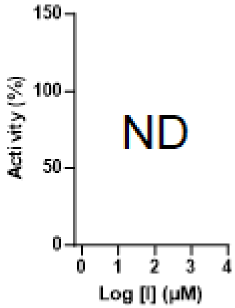
</td></tr>
<tr><td>5d</td><td>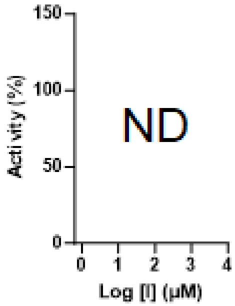
</td></tr>
<tr><td>5e</td><td>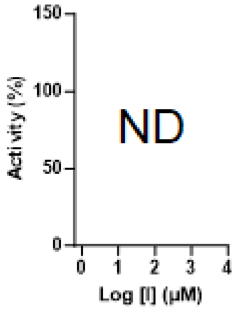
</td></tr>
</table>

**5f**	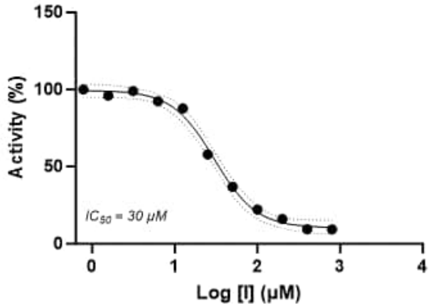Activity (%)<br>Log [I] (μM)<br>IC50 = 30 μM
**5g**	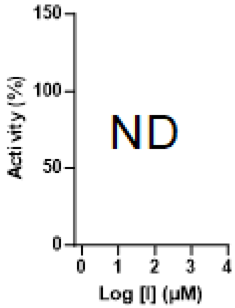Activity (%)<br>ND<br>Log [I] (μM)
**5h**	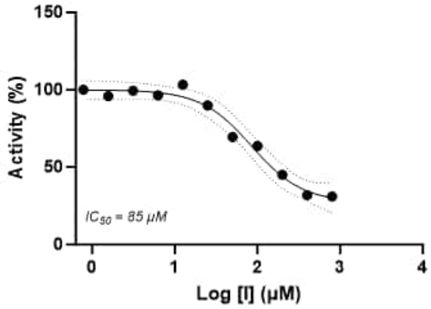Activity (%)<br>Log [I] (μM)<br>IC50 = 85 μM
**5i**	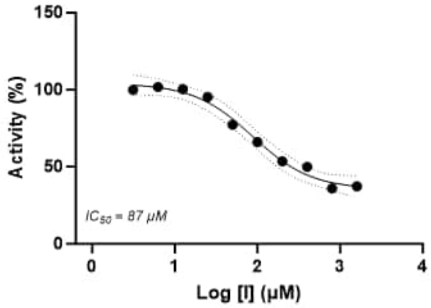Activity (%)<br>Log [I] (μM)<br>IC50 = 87 μM
**5j**	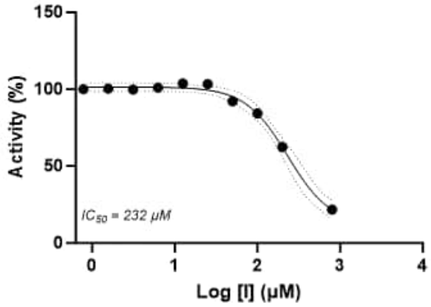Activity (%)<br>Log [I] (μM)<br>IC50 = 232 μM

**5k**	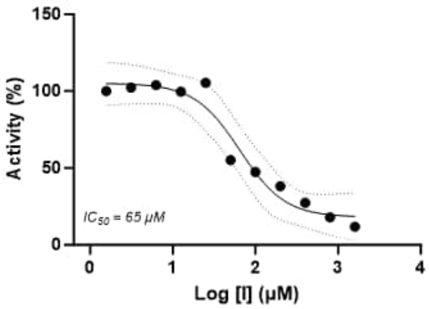 Activity (%) · Log [I] (μM) · IC50 = 65 μM
**5l**	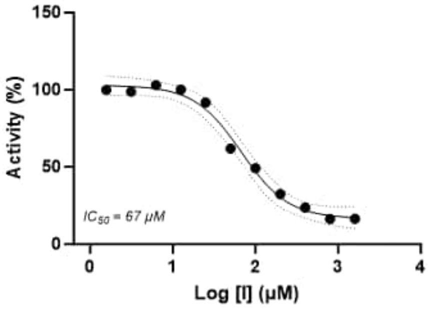 Activity (%) · Log [I] (μM) · IC50 = 67 μM
**5m**	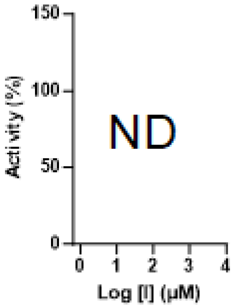 Activity (%) · ND · Log [I] (μM)

7. Enzyme kinetic studies

Enzyme steady state kinetic studies were performed to determine the mechanism of inhibition using inhibitor **5g** and VIM-2. K_m’, K_i and V_{max} values were determined (**Table S6**).

For data analysis, SimFit software was used to fit the initial reaction velocities (v_0) to a Michaelis-Menten (1:1) model (**Figure S40a**). The data were also analyzed using a Lineweaver-Burk plot, to assess the inhibition mode which both suggest competitive inhibition with a K_i value of 36.0 ± 3.3 µM (**Figure S40b**).

As the inhibitors **5a-m** are structurally closely related and VIM-2, NDM-1 and GIM-1 belong to the same enzyme family, these inhibitors can all be presumed to act with a competitive mechanism on the studied metallo-β-lactamases.

Table S6. *Kinetic parameters (K_m’, K_i and v_{max} values) of inhibitor* ***5g*** *against VIM-2 using nitrocefin as reporter substrate.*

**[I]**	V_{max}	K_m’	K_i
0 µM	49.4 ΔA/min	13.8 µM	-
36 µM	50.0 ΔA/min	29.6 µM	31.3 µM
72 µM	47.0 ΔA/min	38.1 µM	40.7 µM

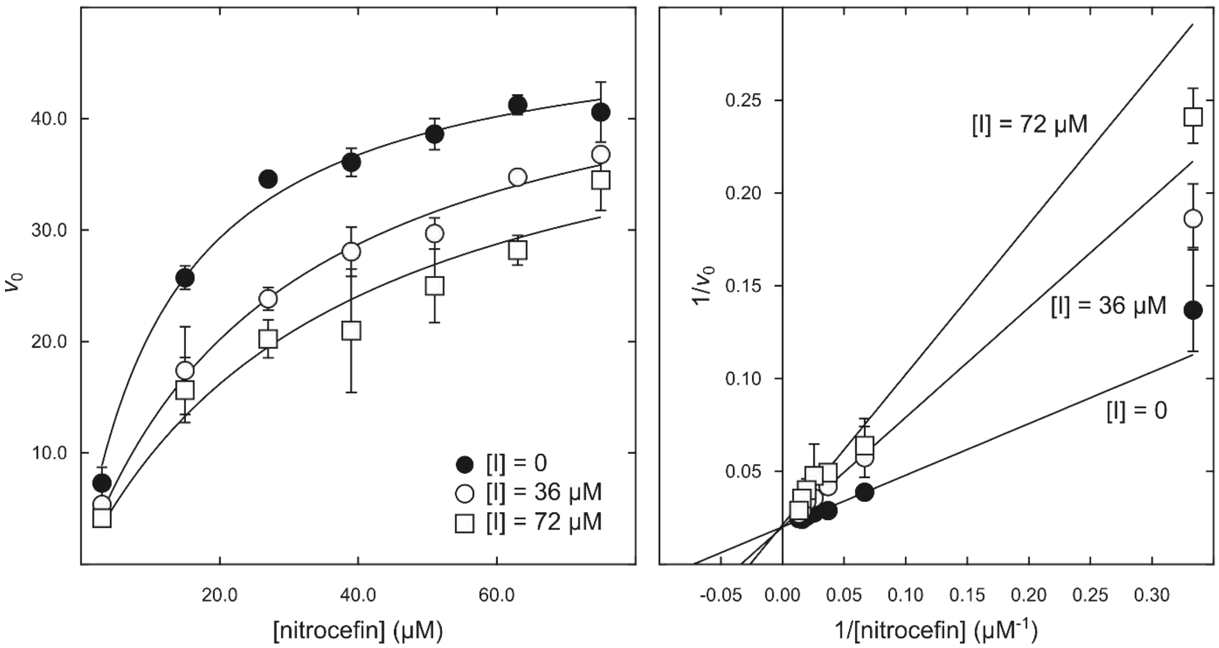

Figure S40. a, *Michaelis-Menten fitting of the substrate concentration dependence of initial reaction velocities.* ***b,*** *Double reciprocal* ***plot of the data in*** *a, for VIM-2 in the absence and presence of inhibitor* ***5g*** *at two concentrations (36 µM and 72 µM) using nitrocefin as substrate. [I] is the concentration of inhibitor* ***5g.***

8. Cytotoxocity against HepG2 cells

Table S7. *CC_{50} values and viability curves of metallo-β-lactamase inhibitors tested on HepG2 cells. CC_{50} values were measured with MTT assay using 0.0025 mM - 1mM concentration range. Data are shown as % viability values ± SEM (n=4). Calculations were done using GraphPad Prism software.*

Inhibitors	CC_{50} (µM)	HepG2
**5a**	4554	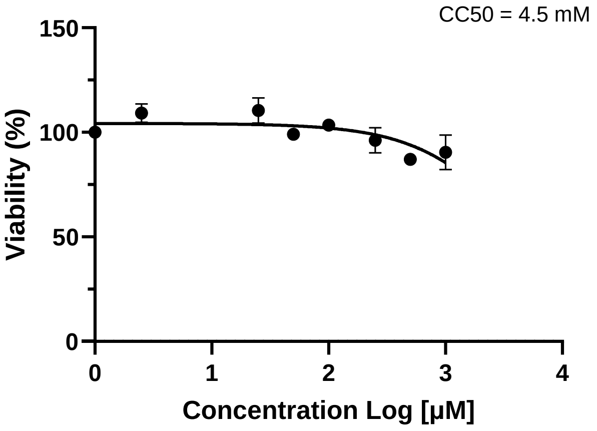
**5b**	1833	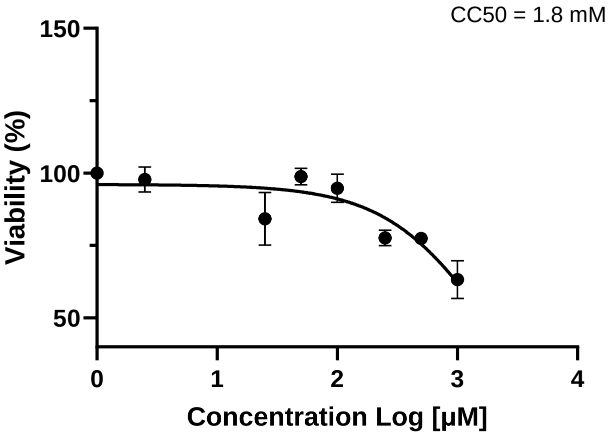
**5c**	2930	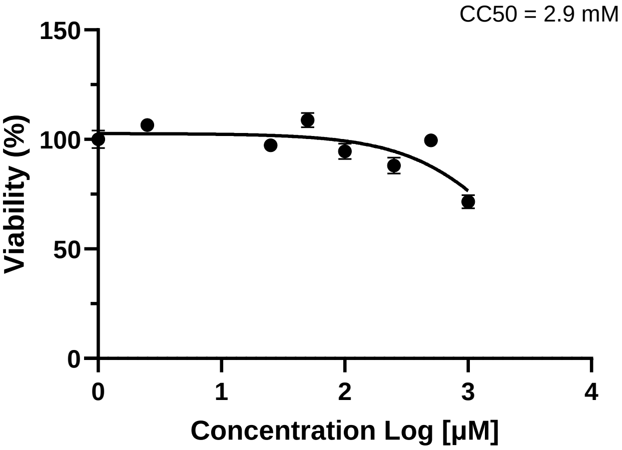

**5d**	4437	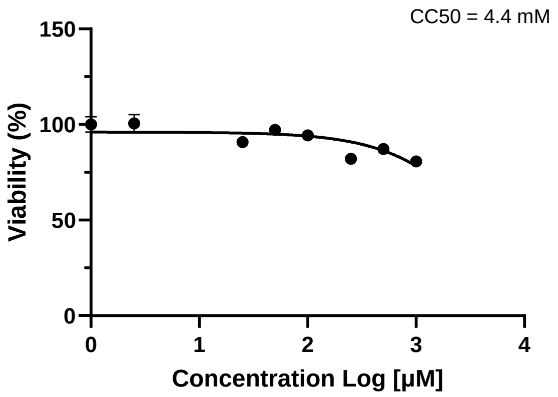
**5e**	(> 1000 µM)	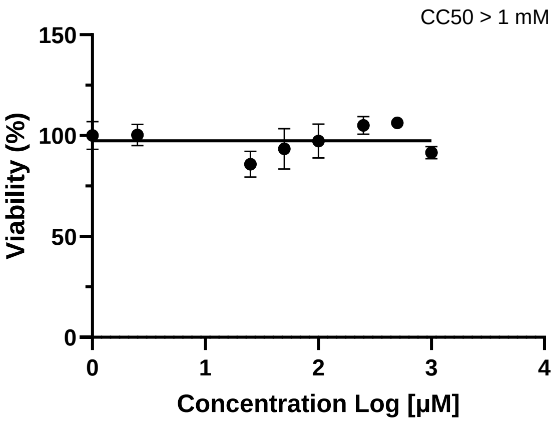
**5f**	4801	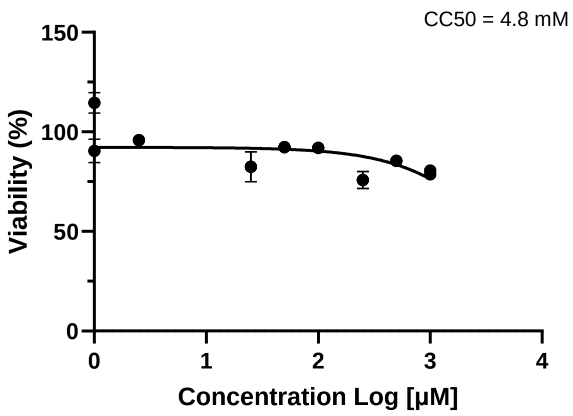
**5g**	1422	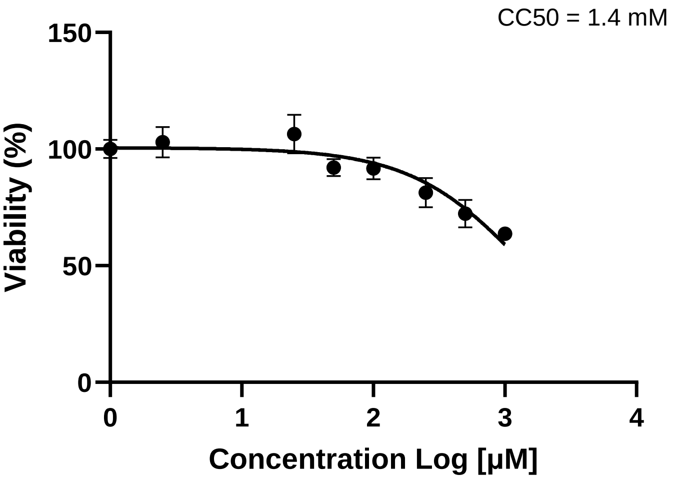

**5h**	(> 1000 µM)	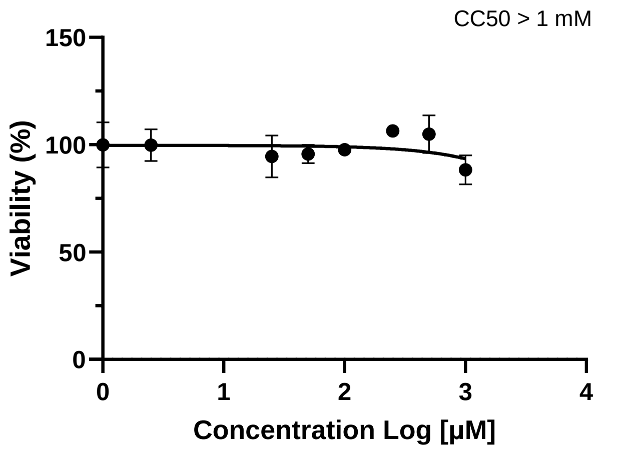
**5i**	1493	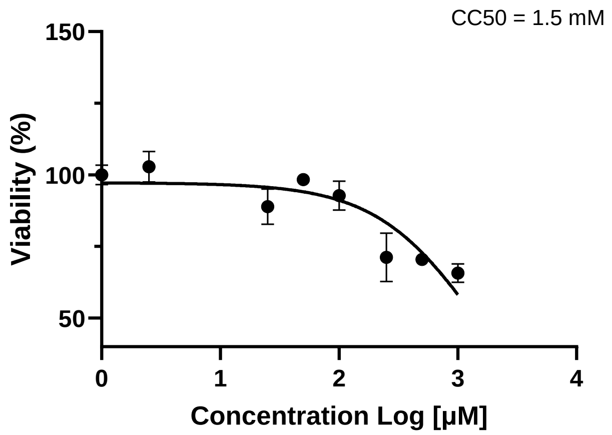
**5j**	3970	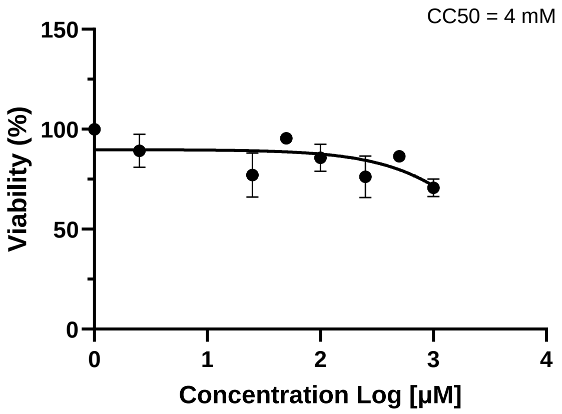
**5k**	5608	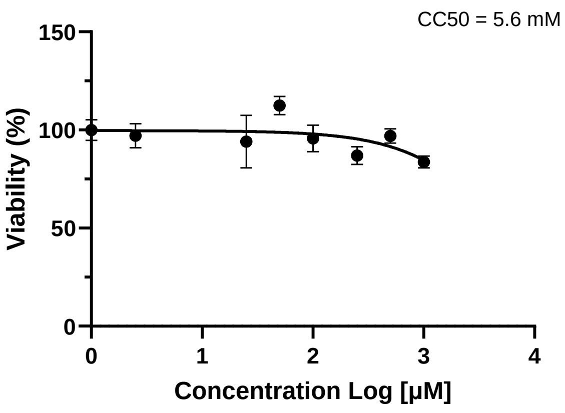

**5l**	3909	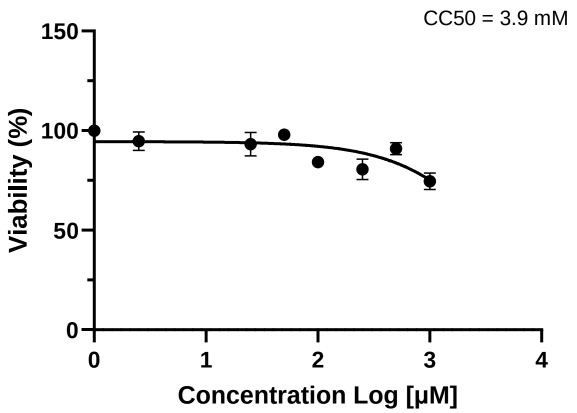
**5m**	(> 1000 μM)	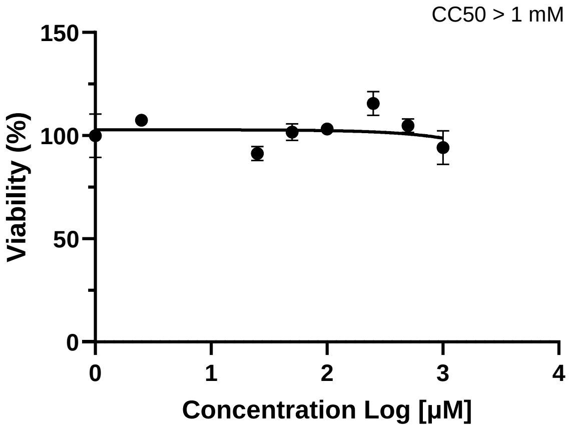

9. Inhibitor binding comparison to previous phosphorous-containing metallo-β-lactamase inhibitors

Previously developed, heteroaryl phosphonate inhibitor by Chen and Pemberton et *al*. was crystallized with VIM-2. In the crystal structure, the phosphonate group of their inhibitor is positioned exactly as for our inhibitors. However, the rest of the molecule being not structurally similar, shows no siginificant similarity with our inhibitors, except for the *(R)*-isomers of our inhibitors where the phenyl group is located similarly as the heteroaryl group of the literature compound (**Figure S41a**). Another heteroaryl phosphonate inhibitor 6-phosphonomethylpyridine-2-carboxylate, utilizing several zinc-binding motifs, was successfully crystallized with the B1 class metallo-β-lactamase IMP-1. However, in this crystal structure it was not the phosphonate group that coordinates to the zinc ions rather the pyridine nitrogen and the carboxylate group. Therefore, this binding mode does not resemble the binding of our inhibitors. Only the nitrogens in the *(S)*-isomers of our molecules are located similarly as the pyridine nitrogen of the literature compound (**Figure S41b**).

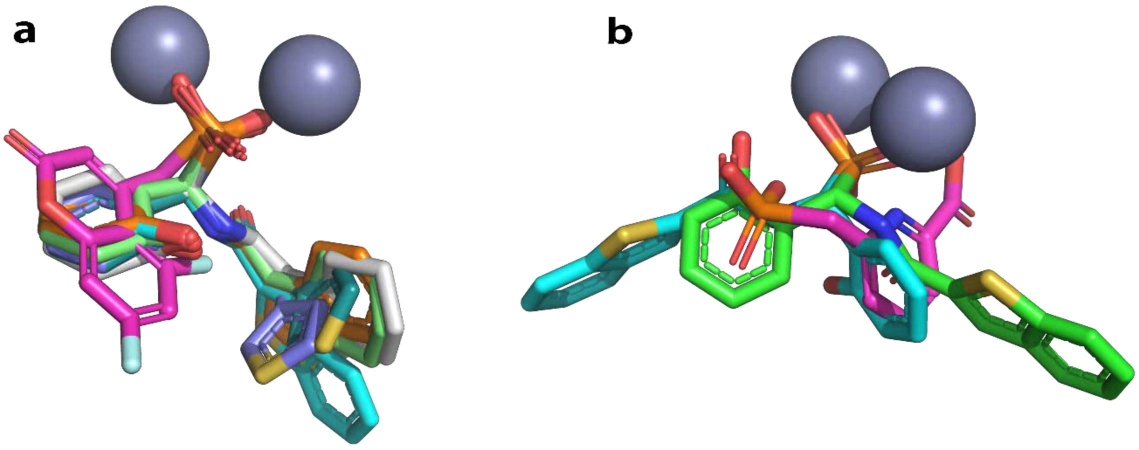

Figure S41. *Comparison of the binding mode of inhibitors* ***5c-j*** *with that of previously developed phosphonate-type metallo-β-lactamase inhibitors.* ***a,*** *Overlay of the heteroaryl phosphonate inhibitor previously developed by Chen et al. (magenta, PDB ID: 6DD0*[10]*) and the (R)-isomers of inhibitor* ***5c*** *(cyan, PDB ID: 9F0P),* ***5d*** *(orange, PDB ID: 9F0Q),* ***5g*** *(gray and light green, PDB ID: 9F0S) and* ***5j*** *(purple and deepteal, PDB ID: 9F0R) bound to VIM-2.* ***b,*** *Overlay of the 6-phosphonomethylpyridine-2-carboxylate inhibitor (magenta, PDB ID: 5HH4*[57]*) bound to IMP-1 and the two stereoisomers of our inhibitor* ***5c*** *(green (S)-isomer, cyan (R)-isomer) bound to VIM-2 as an example.*

Table S8. *Inhibitory activities and proposed binding modes of phosphorous-containing metallo-β-lactamase inhibitor candidates from the literature.*

**Inhibitor structure**	**VIM-2**	**NDM-1**	**GIM-1**	**Proposed binding**
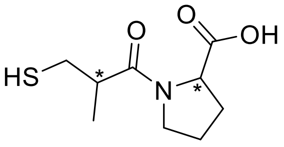 Captopril (mercaptocarboxylic acid)	D-Captopril 0.072 μM (IC_{50})[58] L-Captopril 4.4 μM (IC_{50})[6,58]	D-Captopril 7.9 μM[59], 20.1 μM[58] (IC_{50}) L-Captopril 202.0 μM[59], 157.4 μM (IC_{50})[58]	no data	Zn-binding with -S[58]
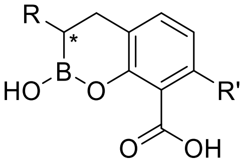 Bicyclic boronates	Taniborbactam 0.002 μM (IC_{50})[35]	Taniborbactam 0.004 μM (IC_{50})[35]	no data	Zn binding with $-B(OH)_2$ and -COOH
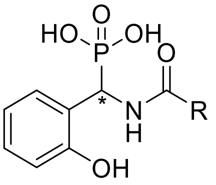 Our dynamically chiral hydroxyphenyl α-aminophosphonates	12 - 124 μM (IC_{50})	30 - 232 μM (IC_{50})	10 - 236 μM (IC_{50})	Zn-binding with $-P(O)(OH)_2$
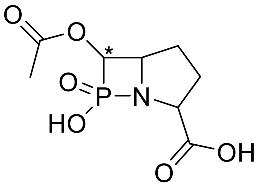 β-Phospholactams	no data	53% inhibition at an inhibitor concentration of 100 μM[60]	no data	Zn-binding with $-P(O)(OH)_2$ and -N[60]
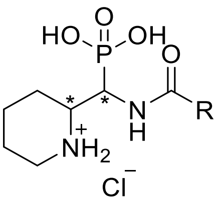 Piperidinyl α-aminophosphonates	4.1 - 328 μM (IC_{50})[4] 0.4 - 15 mM (K_d)[4]	7.9 - 506 μM (IC_{50})[4] 0.5 - 3.1mM (K_d)[4]	no data	Zn-binding with $-P(O)(OH)_2$[4]

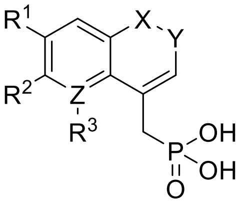 Heteroaryl phosphonates	0.3 - 30.3 μM (K_i)[10]	31.4 - 741.3 μM (K_i)[10]	no data	Zn-binding with -P(O)(OH)$_2$[10]
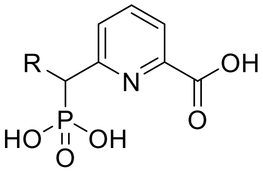 6-Phosphonomethylpyridine-2-carboxylates	0.464 - 1.90 μM (IC_{50})[57]<br>0.038 - 0.61 μM (K_i)[57]	0.306 - 0.374 μM (IC_{50})[57]<br>0.034 - 0.078 μM (K_i)[57]	no data	Zn-binding with -N and –COOH[57]
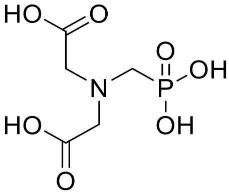 N-(phosphonomethyl)-iminodiacetic acid	0.68 μM (IC_{50})[61]	0.91 μM (IC_{50})[61]	no data	Zn-sequestering[61]
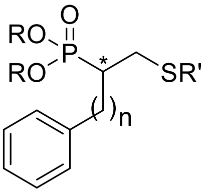 Mercaptophosphonates	0.38 - 133 μM (IC_{50})[62]	1.8 - 144 μM (IC_{50})[62]	0.18 - >5000 μM (IC_{50})[62]	Zn-binding with -S[62]

10. Membrane permeability on *in situ* and on permeabilized membrane

In order to evaluate whether the difference in enzyme inhibition of compounds **5a-m** and L-captopril in the outer membrane vesicles, as shown in **Figure 2b**, originate from membrane penetration or inhibition potency, we also recorded the enzyme activity for OMVs permeabilized with Triton X-100. Whereas diffusion of both the nitrocefin as well as of the inhibitors **5a-m** is limited by the membrane as they compete for the same pores, in the presence of Triton X-100 the vesicle membrane is solubilized and the metallo-β-lactamase becomes freely accessible (**Figure S42**). Since in presence of Triton X-100 the relative effects of the inhibitors are almost identical to the data with intact OMVs, this suggests that in first approximation compounds **5a-m** all penetrate the vesicles equally well.

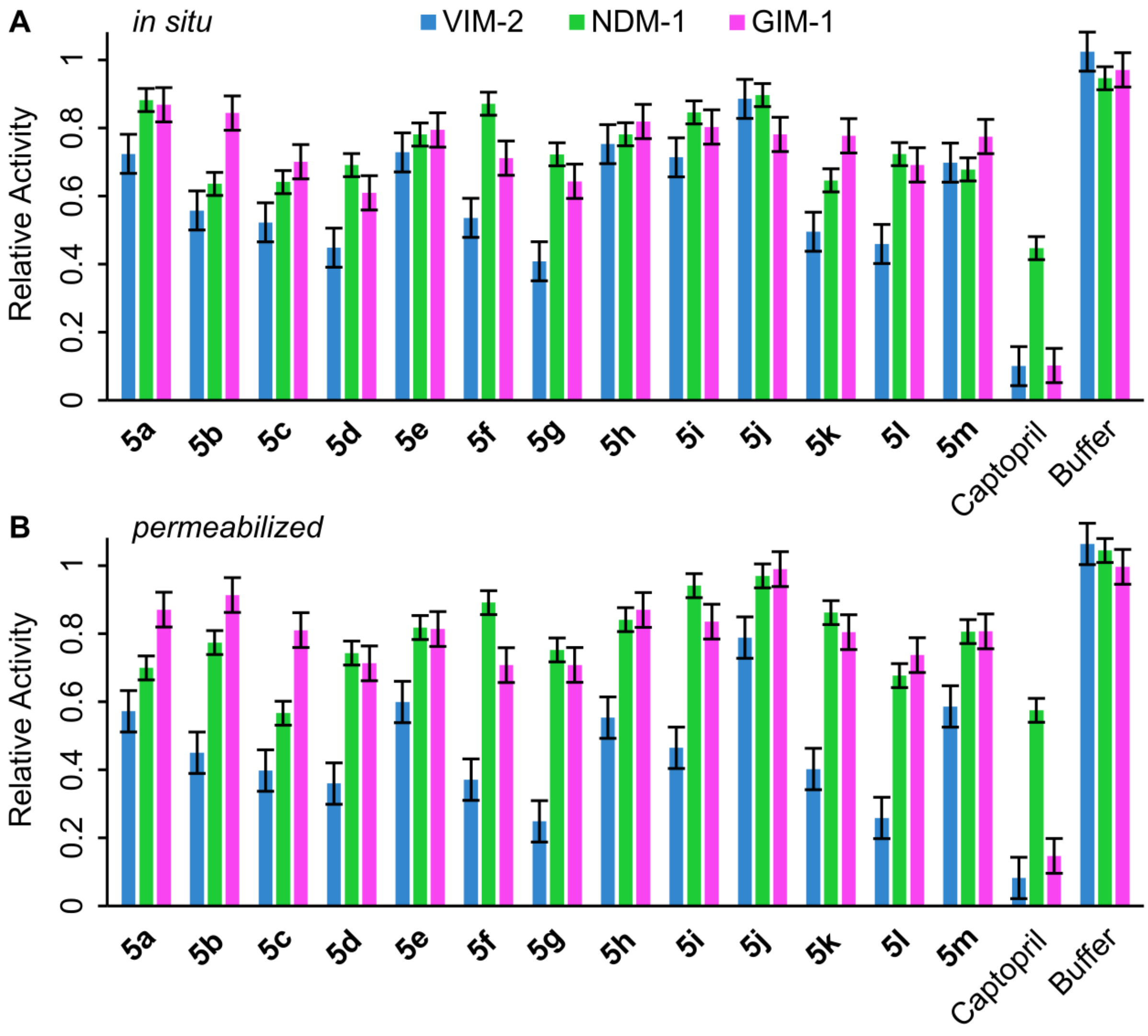

Figure S42. *Normalized relative activity of metallo-β-lactamases encapsulated in outer membrane vesicles in absence (buffer) and presence of inhibitors* ***a,*** *in situ, and* ***b,*** *following permeabilization of the membrane using Triton X-100. Error bars represent the standard deviation of four measurements. All compounds were used at a final concentration of 100 µM. Relative activities based on the slope of the measured data were calculated as described in Methods section.*

11. Prediction of ADME properties

Besides the promising inhibitory activity and lack of cytotoxicity of our inhibitor candidates, the pharmacokinetic properties are also important for drug discovery. To gain more understanding of the drug-like qualities and ADMET properties of the inhibitors, we predicted these using the PreADMET (https://preadmet.webservice.bmdrc.org/) and the SwissADME[36] webservers (**Tables S10-S15**).

The SwissADME webserver calculates LogS values with 3 different methods. Compounds **5a-m** were predicted to be water soluble or moderately soluble. The classification ranges can be found on the Swissadme webpage (http://www.swissadme.ch/). Following the classification from DrugBank database for the PreADMET predictions, out of the 13 compounds, 4-4 compounds fall into the „Freely soluble" (1000000 - 10000 mg/L) and the „Soluble" (10000 - 1000 mg/L) categories, while the rest are „Sparingly soluble" (1000 - 100 mg/L). In summary, the predicted values are in accordance with our experimental observations, which showed overall good aqueous solubility for our phosphonic acid-based compounds.

To describe the lipophilicity of the compounds, logP values were predicted, where a value between 1 and 3 is desired for an orally bioavailable drug candidate and indicates a good balance between permeability and aqueous solubility. From SwissADME predictions, we took the average value of 5 predicted LogPs using different methods. Most of the compounds'' average LogP falls within the criteria, with two exceptions (compounds **5a** and **5b**) having slightly lower values than 1. As seen from the predictions of PreADMET, almost all of our compounds fulfill the above criteria, with two exceptions being compounds **5e** and **5g** that have slightly higher predicted logP values than 3. In general, PreADMET predicted somewhat higher logP values. Both predictions indicate that compounds **5a-m** in general have favourable lipophilicity.

BBB penetration refers to the blood brain barrier penetration ability of a compound, which is not desirable for our metallo-β-lactamase inhibitors. SwissADME predicted no BBB penetration for any of the compounds. For PreADMET, the value is given as c_{brain}/c_{blood}. There are different classifications for CNS-inactive compounds, with preferable c_{brain}/c_{blood} value less than 1.0[37] or 0.4[38]. All of the compounds studied herein are considered CNS-inactive according to the predictions. Another classification[39] differentiates between „High-„ (value is higher than 2.0), „Middle-„ (2.0 - 0.1) and „Low absorption to CNS" (lower than 0.1). Whithin this classification, our compounds are in the Middle category. Overall, both webservers suggest that the coupounds are not likely to cross the blood brain barrier.

Pgp inhibition shows whether a compounds inhibits the P-glycoprotein, which is undesirable for drug candidates as in general no inhibition helps to avoid drug-drug interactions, maintain consistent pharmacokinetics, and reduce the risk of toxicity. According to the SwissADME predictions, 5 out of the 13 compounds might show Pgp inhibition, however PreADMET suggests that none of the compounds are likely to inhibit P-glycoprotein.

The CYP enzyme family plays a crucial role in drug metabolism. Out of the enzymes, CYP3A4 can be generally considered as one of the most important CYP enzyme for drug metabolism as for example it is estimated to be involved in the metabolism of approximately 50% of all marketed drugs.[40] Regarding the undesirable CYP inhibition, our compounds are predicted not

to inhibit CYP3A4, CYP2D6, CYP2C19, CYP1A2, whereas they might inhibit CYP2C9 (according to PreADMET calculations but not for the SwissADME predictions) which is involved in the metabolism of nonsteroidal anti-inflammatory drugs, anticoagulants and certain antidiabetic medications.

A few additional properties were predicted using the PreADMET webserver, such as the „Human Intestinal Absorption" (HIA) which is given as percentage value. It shows the sum of biovailability and absorption, and is evluated from excretion ratio.[41] A high value is desired, where 20 - 70 % means that the compounds are moderately absorbed, and 70 - 100 % refers to well absorbed compounds.[42]. Most of the compounds were predicted to be well absorbed, with only 4 exceptions (compounds **5a**, **5b**, **5i**, **5j**) being moderately absorbed (having values 60.0 and 63.7, for the four tiophene-containing compounds).

To address cell premeability, Caco-2 (human colon adenocarcinoma) and MDCK (Madin Darby Canine Kidney) cell permeqability models can be used. The values are given in nm/sec. In the case of Caco-2, a value lower than 4 nm/sec refers to low permeability[43,44], whereas this value for the MDCK model is 500 nm/sec. It can be concluded, that all of our compounds are predicted to have very low permeability according to both models. However, these compounds are designed to inhibit metallo-β-lactamase enzymes of bacteria, which are not located in human cells, thus, high cell permeability is not an essential requirement.

The last predicted value is plasma protein binding (PPB), which shows the percentage of a compound bound to plasma protein, and the percentage of the unbound drug available for action. The predictions show that our compounds have high plasma protein binding. However, literature data suggest that plasma protein binding has only a minor contribution to the *in vivo* efficacy of a drug candidate therefore it is not advisable in general to optimaze a drug candidate on plasma protein binding.[45,46]

Additionally, druglikeness predictions were also carried out, using the PreADMET and SwissADME webserves. All compounds fulfill the criteria that has been used to describe compounds being potentially good drug candidates, including the Lipinski's Rule of Five[47], the CMC-like rule[48] or the Muegge filter[49] (**Tables S9-S13**).

Table S9. *Criteria for the Lipinski's Rule of Five[47], CMC-like rule[48] and Muegge filter[49].*

	**Lipinski's Rule of Five**	**CMC-like rule**	**Muegge rule**
**MW [g/mol]**	≤ 500	160 ≤ MW ≤ 480	200 ≤ MW ≤ 600
**Log*P***	≤ 5	-0.4 ≤ Log*P* ≤ 5.6	-2 ≤ Log*P* ≤ 5
**Nr. of H-bond acceptors**	≤ 10	-	≤ 10
**Nr. of H-bond donors**	≤ 5	-	≤ 5
**Total nr. of atoms**	-	20 ≤ atoms ≤ 70	-
**Molar Refractivity**	-	40 ≤ MR ≤ 130	-

**TPSA [Å^2]**	-	-	≤ 150
**Nr. of rotatable bonds**	-	-	≤ 15
**Nr. of carbons**	-	-	> 4
**Nr. of heteroatoms**	-	-	> 1
**Nr. of rings**	-	-	≤ 7

Table S10. *Physicochemical properties of compound **5a-f**.*

	**5a**	**5b**	**5c**	**5d**	**5e**	**5f**
**Formula**	$C_{12}H_{12}NO_5PS$	$C_{12}H_{12}NO_5PS$	$C_{16}H_{14}NO_5PS$	$C_{16}H_{14}NO_5PS$	$C_{16}H_{18}NO_5PS$	$C_{16}H_{18}NO_5PS$
**MW [g/mol]**	313.27	313.27	363.32	363.32	367.36	367.36
**Nr. of heavy atoms**	20	20	24	24	24	24
**Nr. of aromatic heavy atoms**	11	11	15	15	11	11
**Nr. of H-bond acceptors**	5	5	5	5	5	5
**Nr. of H-bond donors**	4	4	4	4	4	4
**Molar Refractivity**	74.81	74.81	92.32	92.32	92.24	92.24
**Nr. of rotatable bonds**	5	5	5	5	5	5
**TPSA [Å^2]**[50]	144.91	144.91	144.91	144.91	144.91	144.91

Table S11. *Physicochemical properties of compounds **5g-m**.*

	**5g**	**5h**	**5i**	**5j**	**5k**	**5l**	**5m**
**Formula**	$C_{16}H_{18}NO_5PS$	$C_{14}H_{14}NO_5P$	$C_{13}H_{14}NO_5PS$	$C_{13}H_{14}NO_5PS$	$C_{17}H_{16}NO_5PS$	$C_{17}H_{16}NO_5PS$	$C_{15}H_{16}NO_5P$
**MW [g/mol]**	367.36	307.24	327.29	327.29	377.35	377.35	321.27
**Nr. of HA**	24	21	21	21	25	25	22
**Nr. of aromatic HA**	11	12	11	11	15	15	12
**Nr. of H-bond acceptors**	5	5	5	5	5	5	5
**Nr. of H-bond donors**	4	4	4	4	4	4	4
**Molar Refractivity**	92.24	76.94	79.40	79.40	96.90	96.90	81.52
**Nr. of rotatable bonds**	5	5	6	6	6	6	6
**TPSA [Å^2]**[50]	144.91	116.67	144.91	144.91	144.91	144.91	116.67

Table S12. *SwissADME predictions of compounds **5a-f**.*

	**5a**	**5b**	**5c**	**5d**	**5e**	**5f**
**Consensus Log $P_{o/w}$**[a]	0.89	0.84	1.91	1.89	1.94	1.97
**Water Solubility**[b]	Soluble	Soluble	Soluble/ Moderately soluble	Soluble/ Moderately soluble	Soluble/ Moderately soluble	Soluble/ Moderately soluble
**BBB permeant**	No	No	No	No	No	No
**P-gp substrate**	No	No	No	No	Yes	Yes

**CYP3A4 inhibitor**	No	No	No	No	No	No
**CYP2D6 inhibitor**	No	No	No	No	No	No
**CYP2C19 inhibitor**	No	No	No	No	No	No
**CYP2C9 inhibitor**	No	No	No	No	No	No
**CYP1A2 inhibitor**	No	No	No	No	No	No

a, Average of the 5 predicted Log $P_{o/w}$ values predicted by the SwissADME webserver (iLogP[51], XlogP3 [XLOGP program, version 3.2.2], WlogP[52], MlogP[47,53,54], SILICOS-IT [FILTER-IT program, version 1.0.2]). b, According to the three Log*S* values predicted by the SwissADME webserver (ESOL[55], Ali[56], SILICOS-IT [FILTER-IT program, version 1.0.2]).

Table S13. *SwissADME predictions of compounds* ***5g-m***.

	5g	5h	5i	5j	5k	5l	5m
**Consensus Log $P_{o/w}$[a]**	2.04	0.96	0.99	0.91	1.98	1.93	1.01
**Water Solubility[b]**	Soluble/ Moderately soluble	Soluble	Soluble	Soluble	Soluble/ Moderately soluble	Soluble/ Moderately soluble	Soluble
**BBB permeant**	No	No	No	No	No	No	No
**P-gp substrate**	Yes	No	No	No	Yes	Yes	No
**CYP3A4 inhibitor**	No	No	No	No	No	No	No
**CYP2D6 inhibitor**	No	No	No	No	No	No	No
**CYP2C19 inhibitor**	No	No	No	No	No	No	No
**CYP2C9 inhibitor**	No	No	No	No	No	No	No
**CYP1A2 inhibitor**	No	No	No	No	No	No	No

a, Average of the 5 predicted Log $P_{o/w}$ values predicted by the SwissADME webserver (iLogP[51], XlogP3 [XLOGP program, version 3.2.2], WlogP[52], MlogP[47,53,54], SILICOS-IT [FILTER-IT program, version 1.0.2]). b, According to the three Log*S* values predicted by the SwissADME webserver (ESOL[55], Ali[56], SILICOS-IT [FILTER-IT program, version 1.0.2]).

Table S14. *PreADME predictions of compounds* ***5a-f***.

	5a	5b	5c	5d	5e	5f
**Water solubility [mg/L]**	17552	9017	554	270	1173	799
**logP**	1.69	1.40	2.89	2.60	3.08	2.78
**BBB penetration**	0.147	0.124	0.267	0.215	0.333	0.269
**HIA [%]**	60.0	60.0	86.9	86.9	73.9	73.9

**Pgp inhibition**	Non	Non	Non	Non	Non	Non
**CYP3A4 inhibition**	Non	Non	Non	Non	Non	Non
**CYP2D6 inhibition**	Non	Non	Non	Non	Non	Non
**CYP2C19 inhibition**	Non	Non	Non	Non	Non	Non
**CYP2C9 inhibition**	Inhibitor	Inhibitor	Inhibitor	Inhibitor	Inhibitor	Inhibitor
**Caco-2 permeability**	0.371	0.390	0.410	0.383	0.416	0.384
**MDCK permeability**	4.4	2.4	68.7	52.0	85.1	67.7
**PPB [%]**	94.4	91.9	97.9	96.8	94.2	93. 4

Table S15. *PreADMET predictions of compounds* ***5g-m****.*

	**5g**	**5h**	**5i**	**5j**	**5k**	**5l**	**5m**
**Water solubility [mg/L]**	337	9438	40730	20924	1278	624	21918
**logP**	3.21	1.74	1.73	1.44	2.93	2.64	1.77
**BBB penetration**	0.373	0.299	0.150	0.126	0.228	0.197	0.309
**HIA [%]**	73.9	77.2	63.7	63.7	88.3	88.3	79.8
**Pgp inhibition**	Non	Non	Non	Non	Non	Non	Non
**CYP3A4 inhibition**	Non	Non	Non	Non	Non	Non	Non
**CYP2D6 inhibition**	Non	Non	Non	Non	Non	Non	Non
**CYP2C19 inhibition**	Non	Non	Non	Non	Non	Non	Non
**CYP2C9 inhibition**	Inhibitor	Inhibitor	Inhibitor	Inhibitor	Inhibitor	Inhibitor	Inhibitor

**Caco-2 permeability**	0.387	0.762	0.374	0.453	0.475	0.396	0.490
**MDCK permeability**	105.2	30.7	17.3	9.5	5.8	27.8	66.7
**PPB [%]**	94.0	99.6	94.8	95.8	98.1	100.0	99.9

12. ^{1}H, ^{15}N-HSQC titration experiments and chemical shift perturbations

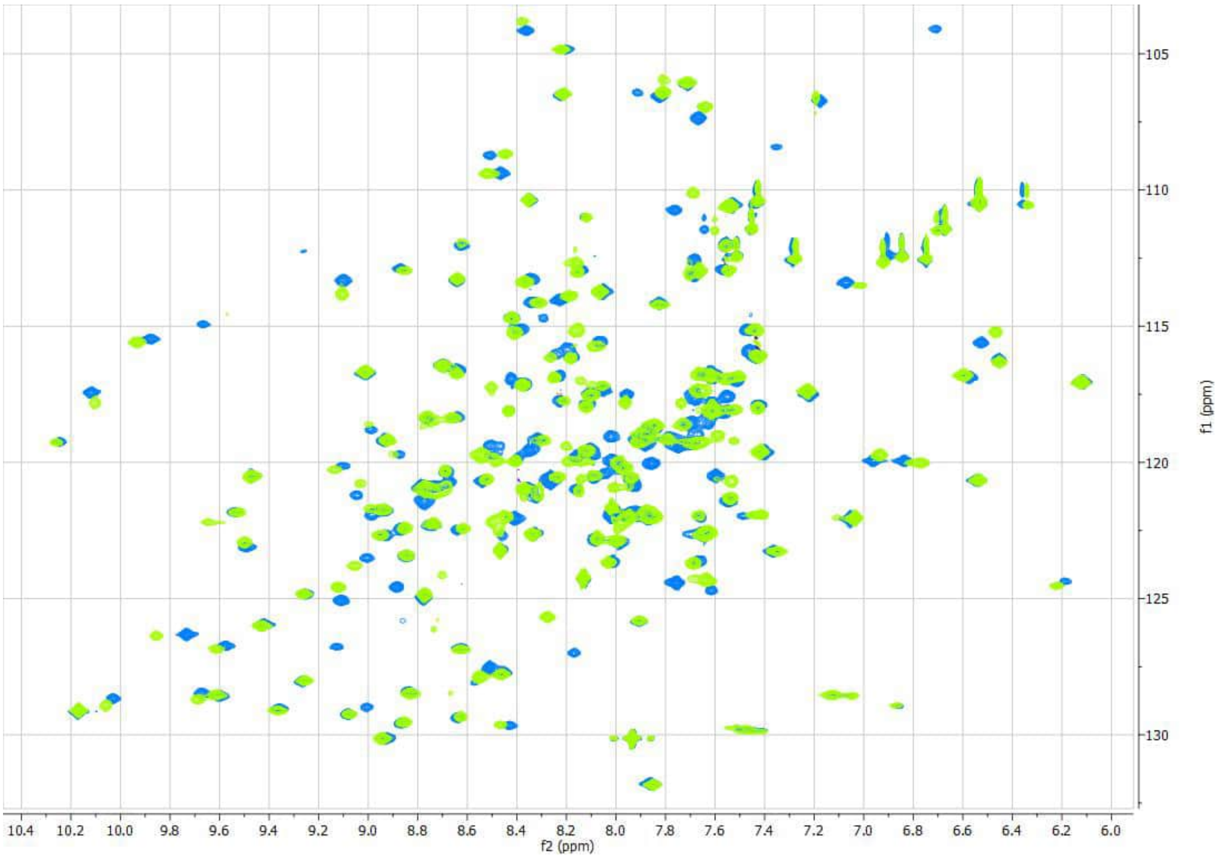

Figure S43. *Superimposition of the ^{1}H,^{15}N-HSQC spectrum (600 MHz, pH 7.0, 25 °C) of VIM-2 in the absence (blue) and in the presence (green) of 10 molar equivalent of compound* ***5c***. *Buffer: 20 mM KPO_4, 0.1 mM $ZnCl_2$, with 15% D_2O.*

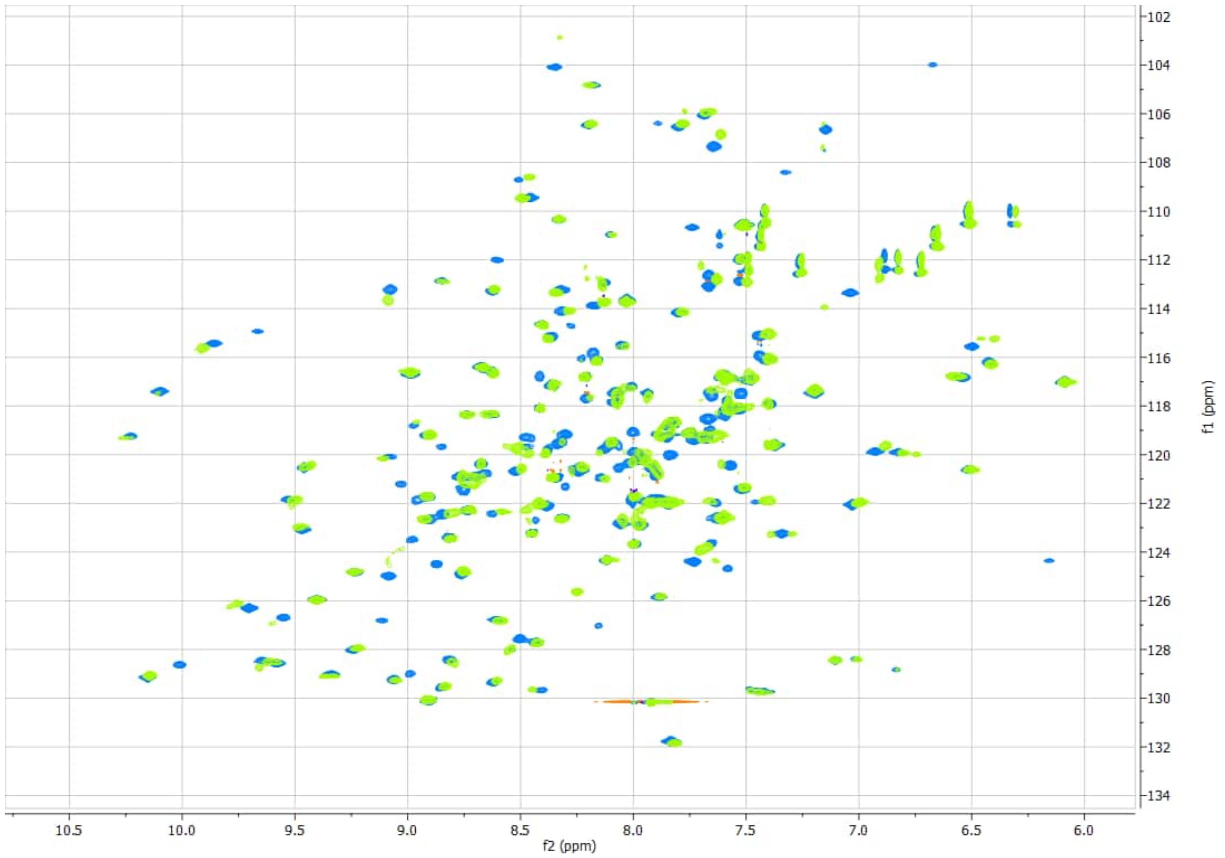

***Figure S44**. Superimposition of the $^{1}H,^{15}N$-HSQC spectrum (600 MHz, pH 7.0, 25 °C) of VIM-2 in the absence (blue) and in the presence (green) of 10 molar equivalent of compound **5d**. Buffer: 20 mM KPO_4, 0.1 mM $ZnCl_2$, with 15% D_2O.*

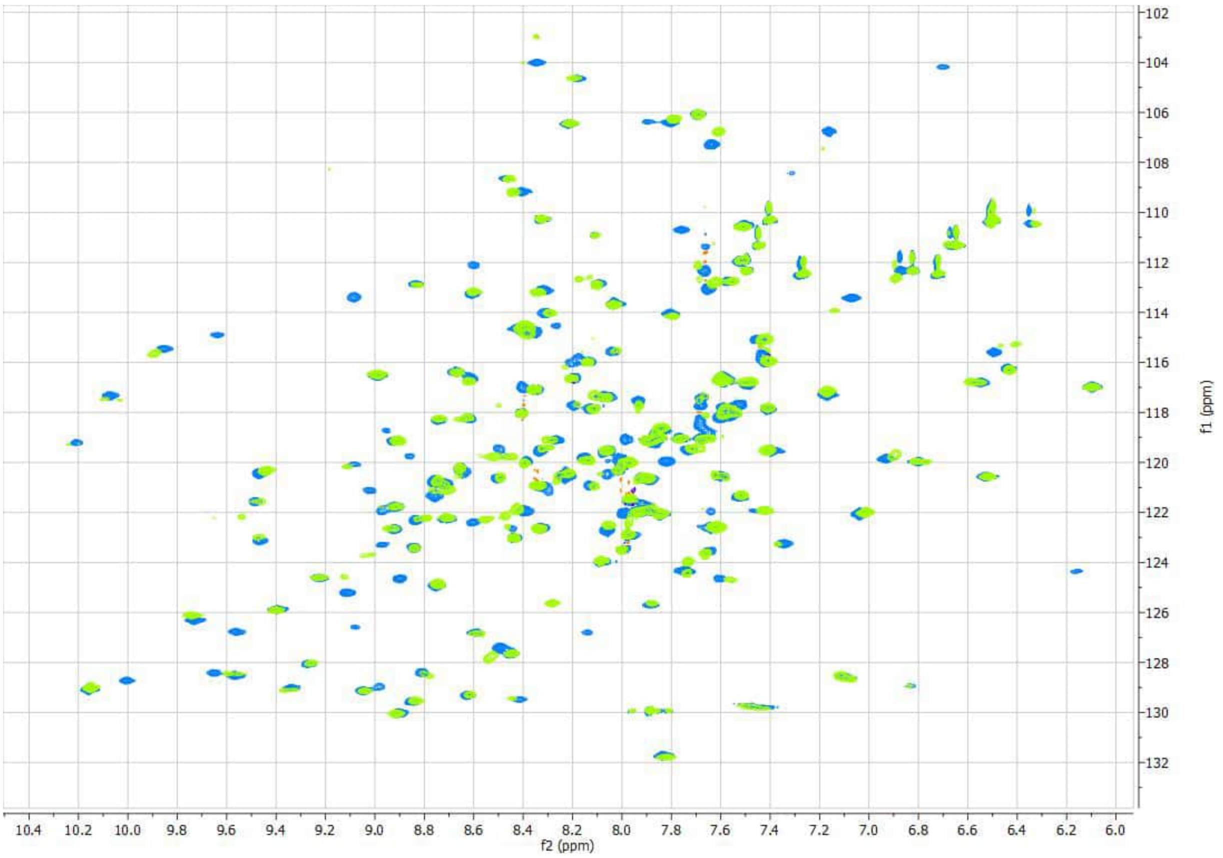

Figure S45. *Superimposition of the* $^{1}H,^{15}N$*-HSQC spectrum (600 MHz, pH 7.0, 25 °C) of VIM-2 in the absence (blue) and in the presence (green) of 10 molar equivalent of compound* ***5g***. *Buffer: 20 mM* KPO_4*, 0.1 mM* $ZnCl_2$*, with 15%* D_2O *and 5% EtOH.*

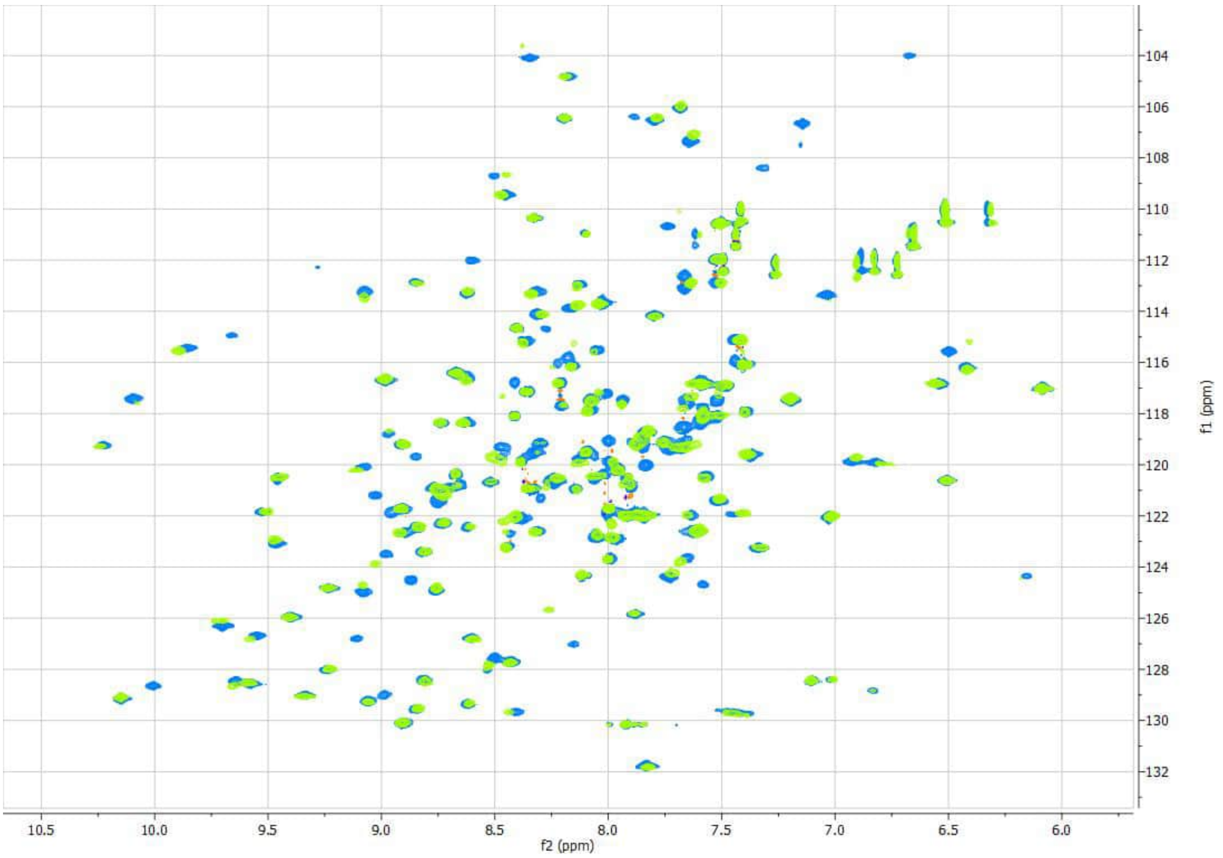

Figure S46. *Superimposition of the* $^{1}H,^{15}N$*-HSQC spectrum (600 MHz, pH 7.0, 25 °C) of VIM-2 in the absence (blue) and in the presence (green) of 11 molar equivalent of compound* ***5i***. *Buffer: 20 mM* KPO_4*, 0.1 mM* $ZnCl_2$*, with 15%* D_2O*.*

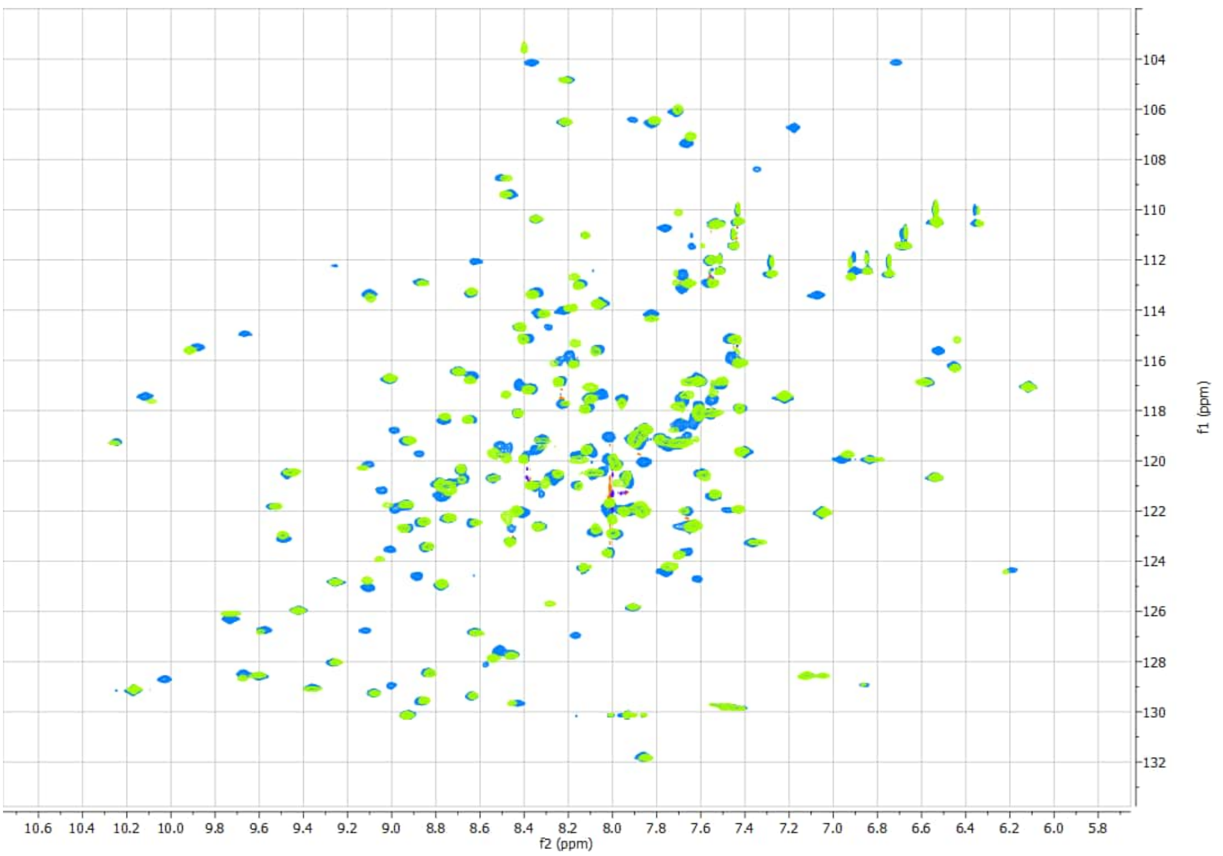

Figure S47. *Superimposition of the ^{1}H,^{15}N-HSQC spectrum (600 MHz, pH 7.0, 25 °C) of VIM-2 in the absence (blue) and in the presence (green) of 10 molar equivalent of compound* ***5j****. Buffer: 20 mM KPO_4, 0.1 mM $ZnCl_2$, with 15% D_2O.*

Chemical shift perturbation values (CSP, $\Delta\delta_{NH}$) were calculated as the weighted average of the chemical shift changes in f1 ($\Delta\delta_N$) and f2 ($\Delta\delta_H$) dimensions using the following equation[8]:

$$\Delta\delta_{NH} = \sqrt{\Delta\delta_H{}^2 + \left(\frac{\Delta\delta_N}{R_{scale}}\right)^2} \; ; \; R_{scale} = 6.5$$

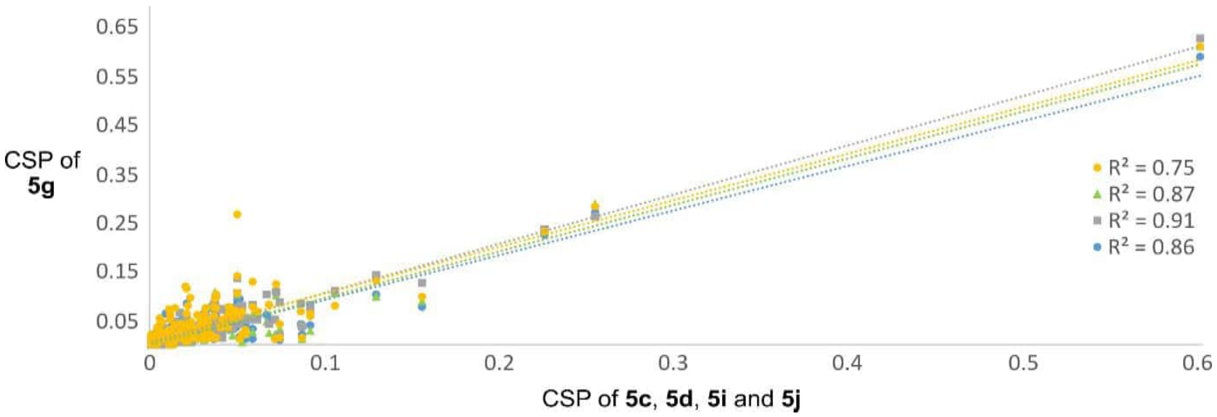

Figure S48. *Linear regression of the chemical shift perturbations of compound* ***5g*** *(IC_{50} = 12 µM) as a function of the chemical shift perturbations of compounds* ***5c*** *(yellow circle),* ***5d*** *(grey square)****,*** ***5i*** *(green triangle) and* ***5j*** *(blue circle) for VIM-2. The data of the studied*

*inhibitors is compared to that of **5g** as it is the most active compound. The graph indicates that all studied inhibitors bind the same active site in comparable binding modes.*

Table S16. *Chemical shift perturbations for VIM-2 in the presence of compound **5c**.*

**Compound 5c**	Free VIM-2		VIM-2 in the presence of 10 equiv. of ligand		Difference		**CSP (ppm)**
	**^{1}H (ppm)**	**^{15}N (ppm)**	**^{1}H (ppm)**	**^{15}N (ppm)**	**Δ^{1}H (ppm)**	**$\Delta\,^{15}$N (ppm)**	
28D	8.46	123.2	8.47	123.3	-0.01	0.0	0.01
29S	8.17	116.1	8.18	116.1	-0.01	0.0	0.01
30S	8.43	118.1	8.43	118.1	0.00	-0.1	0.01
31G	8.35	110.4	8.35	110.4	0.00	0.0	0.00
32E	8.35	121.0	8.37	121.0	-0.02	0.0	0.02
33Y	8.51	127.6	8.55	127.9	-0.04	-0.3	0.07
35T	7.76	110.7	7.68	110.1	0.08	0.6	0.12
36V	8.42	116.9	8.50	117.2	-0.08	-0.3	0.09
37S	8.13	111.0	8.12	111.0	0.01	0.0	0.01
38E	7.62	118.3	7.61	118.1	0.01	0.1	0.02
39I	6.96	119.9	6.94	119.7	0.02	0.2	0.03
43E	7.92	119.2	7.90	119.0	0.02	0.2	0.04
44V	8.2	115.8	8.16	115.2	0.04	0.6	0.10
45R	8.99	121.9	9.00	121.7	-0.01	0.2	0.04
46L	8.83	119.0	8.72	119.5	0.12	-0.4	0.13
47Y	9.01	123.5	9.05	123.8	-0.04	-0.3	0.06
48Q	8.64	129.4	8.63	129.4	0.01	0.0	0.01
49I	8.43	129.7	8.47	129.6	-0.04	0.0	0.04
50A	7.88	119.3	7.91	119.3	-0.03	0.0	0.03
51D	8.39	119.9	8.41	120.0	-0.02	-0.1	0.02
52G	8.14	112.9	8.16	113.0	-0.02	-0.1	0.02
53V	7.22	117.5	7.23	117.4	-0.01	0.1	0.02
54W	9.67	128.5	9.68	128.7	-0.01	-0.2	0.04
55S	9.88	115.5	9.93	115.6	-0.05	-0.1	0.05
56H	7.69	118.6	7.73	118.6	-0.04	0.0	0.04
57I	9.04	121.2	9.03	120.8	0.01	0.4	0.07
58A	8.57	128.1					
64G	8.36	104.2	8.38	103.8	-0.02	0.3	0.06
65A	7.75	124.4	7.68	124.3	0.07	0.2	0.08
66V	7.86	120.1	8.01	120.9	-0.15	-0.9	0.20
67Y	8.86	125.8	8.72	125.8	0.14	0.0	0.14
69S	9.01	115.9	9.19	117.2	-0.18	-1.3	0.27
70N	8.49	123.7					
71G	7.91	106.4	7.81	105.9	0.10	0.5	0.13
73I	9.49	123.1	9.50	123.0	-0.01	0.2	0.03
74V	9.42	125.9	9.43	126.0	-0.01	-0.1	0.02
75R	9.26	128.0	9.26	128.0	0.00	0.0	0.00
76D	9.08	129.2	9.07	129.3	0.01	0.0	0.01
77G	8.64	113.3	8.64	113.3	0.00	0.1	0.01

78D	8.86	129.6	8.85	129.5	0.01	0.1	0.01
79E	7.61	116.8	7.66	116.8	-0.05	0.0	0.05
80L	8.7	116.5	8.70	116.5	0.00	0.0	0.00
81L	8.84	123.4	8.84	123.4	0.00	0.0	0.00
82L	8.84	128.4	8.83	128.5	0.01	0.0	0.01
83I	9.58	126.7	9.61	126.9	-0.03	-0.1	0.04
84D	8.99	118.8	8.99	118.6	0.00	0.2	0.03
85T	6.71	104.1	6.80	104.5	-0.09	-0.4	0.11
97A	7.48	122.0	7.42	121.9	0.06	0.0	0.06
98E	8.41	122.1	8.45	122.0	-0.04	0.0	0.04
99I	8.5	119.7	8.48	120.0	0.02	-0.3	0.04
100E	7.64	122.6	7.63	122.6	0.01	0.0	0.01
101K	7.43	116.1	7.42	116.1	0.01	0.0	0.01
102Q	8.34	113.3	8.37	113.4	-0.03	0.0	0.03
103I	8.05	117.3	8.06	117.2	-0.01	0.1	0.02
104G	8.2	104.8	8.22	104.8	-0.02	0.0	0.02
105L	6.12	117.1	6.12	117.1	0.00	0.0	0.00
107V	8.74	122.3	8.74	122.3	0.00	0.0	0.00
108T	8.33	114.1	8.31	114.1	0.02	0.0	0.02
109R	7.54	121.4	7.54	121.3	0.00	0.1	0.01
110A	8.94	121.8	8.94	121.8	0.00	0.0	0.01
111V	8.73	120.9	8.73	121.2	0.00	-0.2	0.03
112S	7.66	122.0	7.67	122.0	-0.01	0.0	0.01
124D	7.68	117.5	7.65	117.4	0.03	0.1	0.04
125V	7.66	123.6	7.69	123.7	-0.03	-0.1	0.04
127R	8.77	121.4	8.73	121.2	0.04	0.3	0.06
128A	7.98	122.9	8.00	122.9	-0.02	0.0	0.02
129A	7.4	119.7	7.42	119.6	-0.02	0.0	0.02
130G	7.82	106.6	7.81	106.4	0.01	0.1	0.02
131V	7.92	121.9	7.87	121.9	0.05	0.0	0.05
132A	7.86	131.8	7.85	131.9	0.01	-0.1	0.02
133T	8.25	116.1	8.26	116.1	-0.01	-0.1	0.02
134Y	9.1	120.1	9.14	120.3	-0.04	-0.1	0.04
135A	8.64	118.4	8.66	118.4	-0.02	0.0	0.02
136S	10.25	119.3	10.26	119.3	-0.01	0.0	0.01
138S	7.57	112.9	7.54	112.9	0.03	0.0	0.03
139T	8.16	121.0	8.15	121.1	0.01	-0.1	0.02
140R	8.23	117.7	8.21	117.8	0.02	-0.1	0.02
141R	7.67	119.0	7.65	119.3	0.02	-0.3	0.04
142L	8.02	119.9	7.99	120.0	0.03	-0.1	0.03
143A	8.54	120.7	8.52	120.6	0.02	0.0	0.02
144E	7.56	118.1	7.52	118.1	0.04	-0.1	0.04
145V	7.99	120.2	7.97	120.2	0.02	0.0	0.02
146E	7.68	117.6	7.74	117.8	-0.06	-0.2	0.07
147G	7.71	106.1	7.71	106.1	0.00	0.0	0.01
148N	8.16	119.8	8.19	120.0	-0.03	-0.2	0.04
149E	8.1	119.6	8.14	120.0	-0.04	-0.3	0.07
150I	8.06	115.6	8.08	115.8	-0.02	-0.2	0.03
152T	8.46	122.7	8.47	122.5	-0.01	0.2	0.03
153H	8.76	118.4	8.76	118.3	0.00	0.0	0.01

154S	8.64	116.6	8.64	116.8	0.00	-0.1	0.02
155L	7.91	125.9	7.91	125.8	0.00	0.0	0.01
156E	8.33	122.6	8.34	122.6	-0.01	0.0	0.01
157G	8.22	106.5	8.21	106.5	0.01	0.0	0.01
158L	8.11	119.6	8.12	119.6	-0.01	0.0	0.01
159S	8.22	114.0	8.19	113.9	0.03	0.1	0.04
161S	8.69	120.4	8.69	120.3	0.00	0.0	0.01
162G	8.87	112.9	8.85	113.0	0.02	-0.1	0.02
163D	8.01	123.7	8.03	123.7	-0.02	0.0	0.02
164A	7.94	120.6	7.93	120.6	0.01	0.0	0.01
165V	9.01	116.8	9.01	116.7	0.00	0.1	0.01
166R	8.78	125.0	8.77	124.8	0.01	0.1	0.02
167F	8.93	130.2	8.94	130.2	-0.01	0.0	0.01
168G	8.42	114.7	8.42	114.7	0.00	0.0	0.01
169S	8.93	119.2	8.92	119.2	0.01	0.0	0.01
170V	7.53	110.6	7.54	110.6	-0.01	0.0	0.01
171E	9.54	121.8	9.53	121.8	0.01	0.0	0.01
172L	9.25	124.8	9.26	124.8	-0.01	0.0	0.01
173F	8.93	122.7	8.95	122.7	-0.02	0.0	0.02
174Y	8.45	127.7	8.46	127.8	-0.01	0.0	0.01
177A	7.96	117.5	7.96	117.8	0.00	-0.3	0.05
178A	7.36	123.3	7.35	123.3	0.01	0.0	0.01
179H	6.19	124.4	6.22	124.5	-0.03	-0.2	0.04
180S	7.67	107.4	7.64	107.0	0.03	0.4	0.07
181T	9.67	114.9	9.57	114.6	0.10	0.3	0.11
182D	10.12	117.4	10.10	117.8	0.02	-0.4	0.06
183N	6.45	116.2	6.45	116.3	0.00	-0.1	0.01
184L	8.88	119.7	8.89	119.7	-0.01	0.0	0.01
185V	9.1	113.3	9.11	113.8	-0.01	-0.5	0.08
186V	8.5	119.4	8.54	119.7	-0.04	-0.3	0.07
187Y	9.6	128.6	9.61	128.6	-0.01	0.0	0.01
188V	8.63	126.8	8.63	126.9	0.00	0.0	0.01
190S	7.68	113.1	7.66	113.0	0.02	0.1	0.03
191A	6.54	120.7	6.54	120.7	0.00	0.0	0.00
192S	7.55	112.1	7.56	112.1	-0.01	0.0	0.01
193V	6.57	116.9	6.60	116.8	-0.03	0.1	0.03
194L	9.36	129.1	9.36	129.1	0.00	-0.1	0.01
195Y	10.03	128.7	10.05	128.9	-0.02	-0.3	0.05
196G	8.62	112.1	8.62	112.0	0.00	0.1	0.01
198C	7.66	123.6	7.69	123.7	-0.03	-0.1	0.04
199A	6.52	115.6	6.47	115.2	0.05	0.4	0.08
200I	7.07	113.4	7.01	113.5	0.06	-0.1	0.06
201Y	9.01	129.0					
202E	8.53	116.4	8.32	118.1	0.21	-1.8	0.34
203L	8.33	121.3	8.31	121.0	0.02	0.3	0.05
204S	8.51	108.7	8.45	108.7	0.06	0.0	0.06
205R	7.61	124.7	7.63	124.4	-0.02	0.4	0.06
206T	8.29	114.7	8.18	115.8	0.11	-1.0	0.19
207S	7.46	115.9	7.11	113.6	0.35	2.4	0.51
208A	8.62	124.5	8.74	126.2	-0.12	-1.7	0.28

209G	7.48	106.0					
211V	8.46	119.4	8.54	119.7	-0.08	-0.3	0.10
212A	8.17	127.0	8.28	125.7	-0.11	1.3	0.23
213D	7.91	114.3					
215D.	7.88	118.9	7.87	119.0	0.01	0.0	0.01
216L	8.64	122.5	8.62	122.4	0.02	0.0	0.02
217A	7.86	118.8	7.85	118.7	0.01	0.1	0.02
218E	7.61	117.9	7.61	118.1	0.00	-0.3	0.04
221T	7.42	117.9	7.43	118.0	-0.01	-0.1	0.02
222S	8.87	122.5	8.85	122.4	0.02	0.0	0.02
223I	8.26	120.7	8.31	121.2	-0.05	-0.5	0.10
224E	7.87	122.0	7.84	122.0	0.03	0.0	0.03
225R	8.11	117.9	8.12	118.0	-0.01	-0.1	0.02
226I	7.78	119.1	7.79	119.2	-0.01	0.0	0.01
227Q	8.32	119.2	8.29	119.2	0.03	0.0	0.03
228Q	8.05	113.7	8.07	113.7	-0.02	0.0	0.02
229H	7.51	117.0	7.50	116.9	0.01	0.1	0.02
230Y	7.47	115.2	7.45	115.2	0.02	0.0	0.02
232E	8.37	117.2	8.37	117.2	0.00	0.0	0.00
233A	7.05	122.1	7.04	122.1	0.01	0.0	0.01
234Q	8.67	120.7	8.69	120.9	-0.02	-0.2	0.04
235F	7.55	117.6	7.55	117.0	0.00	0.6	0.09
236V	9.11	125.1	9.12	124.6	-0.01	0.5	0.07
237I	9.12	126.8	9.20	126.9	-0.08	-0.1	0.08
239G	7.35	108.4	7.40	108.1	-0.05	0.4	0.07
240H	7.45	114.6	7.11	113.6	0.34	1.0	0.37
241G	9.26	112.2	9.24	108.3	0.02	4.0	0.61
242L	7.7	119.3	7.59	119.0	0.11	0.3	0.12
244G	8.47	109.4	8.52	109.4	-0.05	0.0	0.05
245G	8.38	115.1	8.41	115.2	-0.03	-0.1	0.03
246L	8.79	120.9	8.77	121.0	0.02	0.0	0.02
247D	9.47	120.5	9.48	120.5	-0.01	0.0	0.01
248L	8.26	120.7	8.23	120.5	0.03	0.1	0.04
249L	8.08	122.8	8.07	122.8	0.01	0.0	0.01
250K	6.84	120.0	6.77	120.0	0.07	-0.1	0.07
251H	8.1	117.5	8.10	117.6	0.00	-0.1	0.01
252T	7.82	114.2	7.83	114.2	-0.01	0.0	0.01
253T	7.61	116.8	7.62	116.9	-0.01	0.0	0.01
254N	7.75	119.4	7.68	119.3	0.07	0.1	0.07
256V	8.02	119.1	8.20	119.4	-0.18	-0.4	0.19
257K	8.35	119.6	8.30	119.4	0.05	0.2	0.06
258A	7.6	120.5	7.53	120.7	0.07	-0.2	0.08
259H	7.63	118.5	7.73	118.6	-0.10	-0.1	0.10
260T	7.68	112.6	7.70	113.1	-0.02	-0.5	0.08
261N	8.04	120.4	8.09	120.5	-0.05	-0.1	0.05
262R	7.93	120.9	7.94	120.9	-0.01	-0.1	0.01
263S	8.23	116.8	8.25	116.9	-0.02	-0.1	0.03
264V	8.02	121.7	8.02	121.7	0.00	0.0	0.01
265V	8.13	124.3	8.13	124.2	0.00	0.0	0.01
266E	7.93	130.2	7.94	130.2	-0.01	0.0	0.01

Table S17. *Mean and standard deviation of the chemical shift perturbations for VIM-2 in the presence of 10 equivalents of compound* ***5c****.*

Compound	Mean (ppm)	Standard deviation (ppm)
**5c**	0.05	0.08

Table S18. *Chemical shift perturbations for VIM-2 in the presence of compound* ***5d****.*

Compound 5d	Free VIM-2		VIM-2 in the presence of 10 equiv. of ligand		Difference		CSP (ppm)
	^{1}H (ppm)	^{15}N (ppm)	^{1}H (ppm)	^{15}N (ppm)	Δ^{1}H (ppm)	Δ ^{15}N (ppm)	
28D	8.45	123.2	8.44	123.2	0.01	0.0	0.01
29S	8.16	116.1	8.17	116.2	-0.01	-0.1	0.01
30S	8.41	118.1	8.41	118.1	0.00	-0.1	0.01
31G	8.33	110.4	8.33	110.4	0.00	0.0	0.00
32E	8.34	120.9	8.36	120.9	-0.02	0.0	0.02
33Y	8.50	127.6	8.54	128.0	-0.04	-0.4	0.07
35T	7.74	110.7	7.67	110.1	0.07	0.5	0.11
36V	8.41	116.9	8.46	117.2	-0.05	-0.3	0.07
37S	8.10	110.9	8.09	111.0	0.01	0.0	0.01
38E	7.59	118.3	7.58	118.1	0.01	0.2	0.03
39I	6.93	119.9	6.88	119.7	0.05	0.2	0.06
43E	7.89	119.2	7.88	119.2	0.01	0.0	0.01
44V	8.17	115.8	8.13	115.2	0.04	0.6	0.10
45R	8.96	121.9	8.98	121.7	-0.02	0.2	0.04
46L	8.84	118.9	8.84	119.2	0.00	-0.3	0.05
47Y	8.98	123.5	9.02	123.9	-0.04	-0.4	0.07
48Q	8.62	129.4	8.61	129.3	0.01	0.1	0.01
49I	8.41	129.7	8.44	129.7	-0.03	0.0	0.03
50A	7.85	119.3	7.87	119.1	-0.02	0.2	0.03
51D	8.38	119.8	8.39	119.9	-0.01	-0.1	0.02
52G	8.13	113.0	8.14	113.0	-0.01	0.0	0.01
53V	7.19	117.5	7.20	117.4	-0.01	0.1	0.02
54W	9.64	128.5	9.66	128.7	-0.02	-0.3	0.04
55S	9.85	115.4	9.91	115.7	-0.06	-0.2	0.07
56H	7.67	118.6	7.63	118.3	0.04	0.3	0.06
57I	9.03	121.2	9.01	120.8	0.02	0.5	0.07
58A	8.54	128.0					
64G	8.35	104.1	8.37	103.9	-0.02	0.2	0.04
65A	7.73	124.4	7.70	123.9	0.03	0.5	0.08
66V	7.83	120.0	7.95	120.8	-0.12	-0.8	0.18
67Y	8.83	125.8	8.87	125.4	-0.04	0.4	0.07
69S	8.97	115.9	9.06	116.5	-0.09	-0.7	0.14
70N	8.45	123.6					
71G	7.89	106.4	7.77	105.9	0.12	0.5	0.14
73I	9.47	123.1	9.48	123.0	-0.01	0.1	0.02
74V	9.40	126.0	9.40	126.0	0.00	0.0	0.00
75R	9.24	128.0	9.22	128.0	0.02	0.1	0.02

76D	9.06	129.3	9.06	129.3	0.00	0.0	0.00
77G	8.62	113.3	8.61	113.2	0.01	0.1	0.02
78D	8.85	129.6	8.83	129.5	0.02	0.0	0.02
79E	7.59	116.8	7.60	116.8	-0.01	0.0	0.01
80L	8.67	116.4	8.67	116.4	0.00	0.0	0.00
81L	8.81	123.4	8.81	123.4	0.00	0.0	0.01
82L	8.81	128.4	8.80	128.5	0.01	-0.1	0.02
83L	9.55	126.7	9.61	126.9	-0.06	-0.2	0.07
84D	8.97	118.8	8.96	118.7	0.01	0.1	0.03
85T	6.68	104.0					
97A	7.46	121.9	7.40	121.9	0.06	0.0	0.06
98E	8.38	122.1	8.42	122.0	-0.04	0.1	0.04
99I	8.47	119.7	8.47	120.0	0.00	-0.3	0.04
100E	7.62	122.6	7.60	122.6	0.02	0.0	0.02
101K	7.41	116.1	7.39	116.1	0.02	0.0	0.02
102Q	8.32	113.3	8.34	113.3	-0.02	-0.1	0.02
103I	8.01	117.2	8.00	117.2	0.01	0.0	0.01
104G	8.17	104.8	8.19	104.8	-0.02	0.0	0.02
105L	6.09	117.0	6.09	117.0	0.00	0.0	0.00
107V	8.73	122.3	8.72	122.3	0.01	0.0	0.01
108T	8.32	114.2	8.28	114.1	0.04	0.1	0.04
109R	7.52	121.4	7.51	121.3	0.01	0.1	0.02
110A	8.92	121.8	8.92	121.7	0.00	0.0	0.01
111V	8.70	120.9	8.71	121.1	-0.01	-0.2	0.04
112S	7.63	122.0	7.66	121.9	-0.03	0.1	0.03
124D	7.66	117.5	7.66	117.3	0.00	0.2	0.02
125V	7.65	123.6	7.68	123.8	-0.03	-0.2	0.04
126L	7.66	118.9	7.57	119.2	0.09	-0.3	0.10
127R	8.75	121.4	8.74	121.2	0.01	0.3	0.04
128A	7.96	122.9	7.97	122.8	-0.01	0.1	0.01
129A	7.37	119.6	7.39	119.6	-0.02	0.0	0.02
130G	7.80	106.5	7.78	106.4	0.02	0.1	0.03
131V	7.89	121.9	7.88	121.9	0.01	0.0	0.01
132A	7.83	131.8	7.82	131.9	0.01	-0.1	0.02
133T	8.23	116.1	8.23	116.2	0.00	-0.1	0.02
134Y	9.08	120.1	9.11	120.2	-0.03	-0.1	0.03
135A	8.62	118.4	8.64	118.3	-0.02	0.0	0.02
136S	10.23	119.2	10.25	119.4	-0.02	-0.1	0.03
138S	7.53	112.8	7.50	112.9	0.03	-0.1	0.03
139T	8.15	121.0	8.13	121.0	0.02	0.0	0.02
140R	8.21	117.7	8.19	117.6	0.02	0.0	0.02
141R	7.68	119.3	7.64	119.3	0.04	0.0	0.04
142L	8.00	119.9	7.98	120.0	0.02	-0.1	0.02
143A	8.52	120.7	8.50	120.6	0.02	0.1	0.03
144E	7.53	118.1	7.52	118.1	0.01	0.1	0.01
145V	7.96	120.2	7.95	120.3	0.01	-0.1	0.01
146E	7.66	117.6	7.70	117.8	-0.04	-0.2	0.05
147G	7.68	106.1	7.67	105.9	0.01	0.1	0.02
148N	8.13	119.8	8.15	119.9	-0.02	-0.2	0.03
149E	8.08	119.7	8.04	119.9	0.04	-0.2	0.05

150I	8.05	115.5	8.04	115.5	0.01	0.0	0.01
152T	8.43	122.7	8.46	122.7	-0.03	0.0	0.03
153H	8.74	118.4	8.74	118.3	0.00	0.0	0.00
154S	8.62	116.6	8.62	116.7	0.00	0.0	0.01
155L	7.88	125.9	7.88	125.9	0.00	0.0	0.00
156E	8.31	122.6	8.32	122.6	-0.01	0.0	0.01
157G	8.20	106.5	8.19	106.4	0.01	0.0	0.01
158L	8.09	119.5	8.10	119.5	-0.01	0.0	0.01
159S	8.17	113.9	8.14	113.7	0.03	0.1	0.04
160S	8.91	119.2	8.90	119.2	0.01	0.0	0.01
161S	8.67	120.4	8.67	120.4	0.00	0.0	0.00
162G	8.85	112.9	8.85	112.9	0.00	0.0	0.00
163D	8.00	123.7	8.00	123.7	0.00	0.0	0.00
164A	7.92	120.6	7.91	120.5	0.01	0.1	0.01
165V	8.99	116.7	8.98	116.6	0.01	0.1	0.01
166R	8.76	124.9	8.75	124.8	0.01	0.1	0.02
167F	8.91	130.1	8.91	130.1	0.00	0.0	0.00
168G	8.41	114.7	8.40	114.7	0.01	0.0	0.01
170V	7.51	110.6	7.51	110.6	0.00	0.0	0.01
171E	9.53	121.8	9.50	121.9	0.03	0.0	0.03
172L	9.24	124.8	9.24	124.8	0.00	0.0	0.00
173F	8.90	122.7	8.93	122.6	-0.03	0.0	0.03
174Y	8.43	127.7	8.42	127.7	0.01	0.0	0.01
177A	7.94	117.5	7.93	117.6	0.01	-0.2	0.03
178A	7.34	123.3	7.29	123.3	0.05	0.0	0.05
179H	6.16	124.3	6.21	124.8	-0.05	-0.5	0.09
180S	7.64	107.3	7.61	106.8	0.03	0.5	0.08
181T	9.67	114.9	9.72	115.0	-0.05	-0.1	0.05
182D	10.10	117.4	10.11	117.5	-0.01	-0.1	0.02
183N	6.42	116.2	6.41	116.3	0.01	-0.1	0.01
184L	8.85	119.7	8.92	119.7	-0.07	-0.1	0.07
185V	9.07	113.2	9.09	113.7	-0.02	-0.5	0.07
186V	8.47	119.3	8.51	119.7	-0.04	-0.4	0.08
187Y	9.58	128.6	9.60	128.5	-0.02	0.0	0.02
188V	8.61	126.8	8.58	126.9	0.03	-0.1	0.03
190S	7.66	113.1	7.63	112.8	0.03	0.3	0.05
191A	6.51	120.6	6.50	120.6	0.01	0.0	0.01
192S	7.53	112.0	7.53	112.0	0.00	0.0	0.00
193V	6.54	116.9	6.58	116.8	-0.04	0.0	0.04
194L	9.34	129.0	9.34	129.1	0.00	-0.1	0.01
195Y	10.01	128.6	10.03	128.7	-0.02	-0.1	0.03
196G	8.60	112.0	8.58	111.8	0.02	0.2	0.04
198C	7.66	123.6	7.68	123.8	-0.02	-0.2	0.04
199A	6.50	115.6	6.40	115.3	0.10	0.3	0.11
200I	7.04	113.4	6.96	113.4	0.08	-0.1	0.08
201Y	8.99	129.0					
202E	8.52	116.4	8.46	117.2	0.06	-0.8	0.14
203L	8.30	121.3	8.27	121.3	0.03	0.0	0.03
204S	8.51	108.7	8.46	108.6	0.05	0.1	0.05
205R	7.58	124.7	7.63	124.4	-0.05	0.3	0.06

206T	8.27	114.7	8.10	114.9	0.17	-0.2	0.17
207S	7.44	115.9	7.15	114.0	0.29	2.0	0.42
208A	8.61	124.4	8.93	126.6	-0.32	-2.1	0.46
211V	8.43	119.3	8.53	119.8	-0.10	-0.5	0.13
212A	8.15	127.0	8.25	125.6	-0.10	1.4	0.23
213D	7.89	114.3					
215D	7.85	118.8	7.84	118.8	0.01	0.1	0.01
216L	8.63	122.4	8.58	122.3	0.05	0.1	0.05
217A	7.83	118.7	7.81	118.7	0.02	0.0	0.02
218E	7.58	117.8	7.57	117.6	0.01	0.2	0.04
221T	7.39	117.9	7.40	117.9	-0.01	0.0	0.01
222S	8.85	122.5	8.80	122.4	0.05	0.1	0.05
223I	8.26	120.7	8.28	120.7	-0.02	0.0	0.02
224E	7.84	122.0	7.84	121.9	0.00	0.1	0.01
225R	8.08	117.8	8.07	117.8	0.01	0.1	0.01
226I	7.75	119.2	7.76	119.1	-0.01	0.0	0.01
227Q	8.30	119.2	8.26	119.2	0.04	0.0	0.04
228Q	8.03	113.7	8.03	113.7	0.00	0.0	0.01
229H	7.48	116.9	7.46	116.9	0.02	0.0	0.02
230Y	7.44	115.2	7.41	115.1	0.03	0.1	0.03
232E	8.36	117.2	8.36	117.2	0.00	0.0	0.00
233A	7.03	122.1	7.00	121.9	0.03	0.1	0.03
234Q	8.65	120.8	8.66	120.9	-0.01	-0.2	0.03
235F	7.52	117.5	7.52	117.0	0.00	0.5	0.07
236V	9.08	125.0	9.08	124.6	0.00	0.4	0.06
237I	9.11	126.8	9.15	126.8	-0.04	0.0	0.04
239G	7.31	108.4	7.34	108.1	-0.03	0.3	0.06
240H	7.43	114.6	7.15	114.0	0.28	0.6	0.30
241G	9.29	112.3	9.20	108.3	0.09	4.0	0.63
242L	7.66	119.2	7.62	119.3	0.04	-0.1	0.04
244G	8.46	109.5	8.49	109.5	-0.03	-0.1	0.03
245G	8.37	115.2	8.38	115.3	-0.01	-0.1	0.02
246L	8.77	120.9	8.75	120.9	0.02	0.1	0.02
247D	9.46	120.5	9.44	120.5	0.02	0.0	0.02
248L	8.24	120.6	8.22	120.5	0.02	0.1	0.03
249L	8.06	122.8	8.05	122.7	0.01	0.1	0.02
250K	6.82	119.9	6.81	119.9	0.01	0.0	0.01
251H	8.08	117.5	8.07	117.6	0.01	-0.1	0.02
252T	7.80	114.2	7.78	114.1	0.02	0.0	0.02
253T	7.60	116.8	7.59	116.9	0.01	0.0	0.01
254N	7.73	119.4	7.69	119.3	0.04	0.1	0.04
256V	8.00	119.1					
257K	8.33	119.5	8.31	119.5	0.02	0.0	0.02
258A	7.57	120.5	7.61	120.4	-0.04	0.0	0.04
259H	7.66	118.5	7.69	118.7	-0.03	-0.2	0.04
260T	7.66	112.7	7.70	112.2	-0.04	0.5	0.08
261N	8.02	120.3	8.00	120.3	0.02	0.0	0.02
262R	7.90	120.9	7.89	120.8	0.01	0.0	0.01
263S	8.21	116.8	8.21	116.9	0.00	-0.1	0.01
264V	8.00	121.8	8.00	121.7	0.00	0.1	0.01

265V	8.12	124.3	8.11	124.3	0.01	0.0	0.01
266E	7.92	130.2	7.92	130.2	0.00	0.0	0.00

Table S19. *Mean and standard deviation of the chemical shift perturbations for VIM-2 in the presence of 10 equivalents of compound* ***5d****.*

Compound	Mean (ppm)	Standard deviation (ppm)
**5d**	0.04	0.07

Table S20. *Chemical shift perturbations for VIM-2 in the presence of compound* ***5g****.*

**Compound 5g**	Free VIM-2		VIM-2 in the presence of 10 equiv. of ligand		Difference		**CSP (ppm)**
	**^{1}H (ppm)**	**^{15}N (ppm)**	**^{1}H (ppm)**	**^{15}N (ppm)**	**Δ^{1}H (ppm)**	**Δ ^{15}N (ppm)**	
28D	8.44	123.0	8.44	123.0	0.00	-0.1	0.01
29S	8.13	116.0	8.14	116.0	-0.01	0.0	0.01
30S	8.41	118.0	8.41	118.1	0.00	0.0	0.00
31G	8.33	110.3	8.33	110.3	0.00	0.0	0.00
32E	8.33	120.9	8.35	121.0	-0.02	0.0	0.02
33Y	8.50	127.4	8.52	127.7	-0.02	-0.3	0.05
35T	7.76	110.7	7.71	110.4	0.05	0.3	0.07
36V	8.41	117.0	8.44	117.1	-0.03	-0.1	0.04
37S	8.11	110.9	8.11	110.9	0.00	0.0	0.01
38E	7.60	118.2	7.58	118.0	0.02	0.2	0.03
39I	6.93	119.8	6.89	119.7	0.04	0.1	0.04
43E	7.89	119.1	7.91	119.1	-0.02	0.0	0.02
44V	8.18	115.9	8.16	115.6	0.02	0.3	0.05
45R	8.97	121.9	8.97	121.8	0.00	0.1	0.02
46L	8.81	119.0	8.80	119.4	0.01	-0.4	0.06
47Y	8.97	123.3	9.03	123.7	-0.06	-0.4	0.09
48Q	8.63	129.3	8.62	129.3	0.01	0.0	0.01
49I	8.41	129.5	8.45	129.5	-0.04	0.0	0.04
50A	7.87	119.2	7.89	119.2	-0.02	0.0	0.02
51D	8.39	119.9	8.39	120.0	0.00	-0.1	0.02
52G	8.10	112.9	8.10	112.9	0.00	0.0	0.01
53V	7.17	117.3	7.17	117.2	0.00	0.1	0.02
54W	9.66	128.4	9.66	128.7	0.00	-0.2	0.03
55S	9.85	115.4	9.89	115.6	-0.04	-0.2	0.05
56H	7.69	118.5	7.68	118.6	0.01	-0.1	0.02
57I	9.02	121.1	9.00	120.8	0.02	0.3	0.05
58A	8.57	128.1					
64G	8.35	104.0	8.40	104.0	-0.05	0.0	0.05
65A	7.74	124.4	7.73	124.0	0.01	0.4	0.06
66V	7.82	120.0	7.94	120.7	-0.12	-0.7	0.16
67Y	8.85	125.7	8.86	125.4	-0.01	0.3	0.05
69S	9.00	115.9	9.02	115.6	-0.02	0.3	0.05
70N	8.48	123.6					

71G	7.90	106.4	7.80	105.8	0.10	0.5	0.13
73I	9.47	123.1	9.48	123.0	-0.01	0.1	0.02
74V	9.40	125.9	9.40	125.9	0.00	0.0	0.00
75R	9.27	128.0	9.26	128.0	0.01	0.0	0.01
76D	9.05	129.1	9.04	129.1	0.01	0.0	0.01
77G	8.61	113.3	8.60	113.2	0.01	0.1	0.01
78D	8.85	129.6	8.83	129.5	0.02	0.1	0.02
79E	7.59	116.7	7.59	116.7	0.00	-0.1	0.01
80L	8.67	116.4	8.67	116.4	0.00	0.0	0.00
81L	8.84	123.4	8.83	123.5	0.01	0.0	0.01
82L	8.82	128.4	8.80	128.5	0.02	-0.1	0.02
83L	9.56	126.8	9.58	127.0	-0.02	-0.2	0.04
84D	8.95	118.7	8.95	118.7	0.00	0.0	0.00
85T	6.70	104.2	6.95	105.3	-0.25	-1.1	0.30
97A	7.45	121.9	7.42	121.9	0.03	0.0	0.03
98E	8.39	121.9	8.43	121.9	-0.04	0.1	0.04
99I	8.47	119.7	8.44	119.8	0.03	-0.1	0.03
100E	7.64	122.6	7.62	122.6	0.02	0.0	0.02
101K	7.42	116.0	7.41	115.9	0.01	0.0	0.01
102Q	8.32	113.1	8.34	113.2	-0.02	-0.1	0.02
103I	8.07	117.4	8.07	117.4	0.00	0.0	0.01
104G	8.18	104.6	8.21	104.6	-0.03	0.0	0.03
105L	6.10	117.0	6.10	117.0	0.00	0.0	0.00
107V	8.71	122.3	8.70	122.2	0.01	0.0	0.01
108T	8.31	114.0	8.29	114.0	0.02	0.0	0.02
109R	7.52	121.4	7.52	121.3	0.00	0.1	0.01
110A	8.92	121.8	8.91	121.8	0.01	0.0	0.01
111V	8.72	120.9	8.70	121.1	0.02	-0.2	0.04
112S	7.64	121.9	7.67	121.9	-0.03	0.1	0.03
124D	7.67	117.4	7.67	117.4	0.00	0.0	0.00
125V	7.65	123.5	7.66	123.6	-0.01	-0.1	0.02
126L	7.65	118.8	7.59	119.0	0.06	-0.2	0.07
127R	8.76	121.3	8.77	121.1	-0.01	0.2	0.04
128A	7.97	122.9	7.97	122.9	0.00	0.0	0.00
129A	7.39	119.5	7.41	119.5	-0.02	0.0	0.02
130G	7.80	106.4	7.79	106.3	0.01	0.1	0.02
131V	7.92	121.9	7.88	121.9	0.04	0.0	0.04
132A	7.83	131.7	7.83	131.8	0.00	-0.1	0.01
133T	8.21	116.0	8.23	116.2	-0.02	-0.2	0.03
134Y	9.09	120.1	9.11	120.2	-0.02	-0.1	0.02
135A	8.62	118.3	8.63	118.2	-0.01	0.1	0.01
136S	10.21	119.2	10.21	119.2	0.00	-0.1	0.01
138S	7.57	112.8	7.55	112.8	0.02	0.0	0.02
139T	8.13	120.9	8.11	121.0	0.02	-0.1	0.02
140R	8.20	117.7	8.18	117.7	0.02	0.1	0.02
141R	7.67	119.0	7.67	119.1	0.00	0.0	0.01
142L	8.01	119.8	7.98	120.0	0.03	-0.2	0.04
143A	8.50	120.6	8.49	120.6	0.01	0.0	0.01
144E	7.56	118.1	7.55	118.0	0.01	0.1	0.02
145V	7.99	120.0	7.96	120.0	0.03	0.0	0.03

146E	7.69	117.8	7.69	117.5	0.00	0.3	0.04
147G	7.69	106.1	7.69	106.1	0.00	0.0	0.00
148N	8.15	119.8	8.14	119.9	0.01	-0.1	0.02
149E	8.06	119.9	8.00	120.0	0.06	0.0	0.06
150I	8.04	115.6	8.02	115.5	0.02	0.0	0.02
152T	8.44	122.6	8.46	122.6	-0.02	0.1	0.02
153H	8.75	118.3	8.74	118.2	0.01	0.1	0.02
154S	8.61	116.6	8.62	116.8	-0.01	-0.1	0.02
155L	7.88	125.7	7.88	125.6	0.00	0.0	0.01
156E	8.33	122.6	8.34	122.6	-0.01	0.0	0.01
157G	8.22	106.4	8.21	106.4	0.01	0.0	0.01
158L	8.06	119.5	8.06	119.5	0.00	0.0	0.00
159S	8.26	114.1	8.23	114.3	0.03	-0.2	0.04
160S	8.92	119.1	8.91	119.1	0.01	0.0	0.01
161S	8.66	120.3	8.66	120.2	0.00	0.1	0.01
162G	8.83	112.9	8.83	112.9	0.00	0.0	0.01
163D	8.00	123.5	8.00	123.5	0.00	0.0	0.00
164A	7.91	120.6	7.93	120.7	-0.02	0.0	0.02
165V	8.99	116.5	8.99	116.5	0.00	0.1	0.01
166R	8.75	124.9	8.75	124.9	0.00	0.1	0.01
167F	8.91	130.0	8.91	130.0	0.00	0.0	0.00
168G	8.41	114.7	8.40	114.6	0.01	0.0	0.01
170V	7.50	110.5	7.51	110.6	-0.01	-0.1	0.01
171E	9.48	121.5	9.46	121.6	0.02	0.0	0.02
172L	9.23	124.6	9.23	124.6	0.00	0.0	0.00
173F	8.93	122.7	8.92	122.6	0.01	0.0	0.01
174Y	8.45	127.7	8.44	127.7	0.01	0.0	0.01
177A	7.94	117.6	7.93	117.7	0.01	-0.2	0.03
178A	7.34	123.3	7.27	123.2	0.07	0.1	0.07
179H	6.16	124.4	6.21	124.7	-0.05	-0.4	0.07
180S	7.64	107.3	7.61	106.8	0.03	0.5	0.09
181T	9.64	114.9	9.66	114.9	-0.02	0.0	0.02
182D	10.07	117.3	10.07	117.5	0.00	-0.1	0.02
183N	6.44	116.2	6.43	116.3	0.01	-0.1	0.02
184L	8.86	119.7	8.91	119.8	-0.05	-0.1	0.05
185V	9.09	113.4	9.08	113.7	0.01	-0.3	0.04
186V	8.50	119.5	8.52	119.8	-0.02	-0.3	0.05
187Y	9.57	128.5	9.55	128.4	0.02	0.1	0.02
188V	8.60	126.8	8.58	126.9	0.02	0.0	0.02
190S	7.65	113.1	7.62	112.8	0.03	0.3	0.05
191A	6.52	120.6	6.52	120.6	0.00	0.0	0.00
192S	7.52	111.9	7.52	111.9	0.00	0.0	0.00
193V	6.55	116.8	6.58	116.8	-0.03	0.0	0.03
194L	9.34	129.0	9.34	129.1	0.00	-0.1	0.02
195Y	10.01	128.7	10.00	128.5	0.01	0.2	0.03
196G	8.60	112.1	8.66	112.5	-0.06	-0.4	0.09
198C	7.66	123.5	7.66	123.6	0.00	-0.2	0.03
199A	6.50	115.6	6.41	115.3	0.09	0.3	0.10
200I	7.07	113.4	6.98	113.5	0.09	-0.1	0.09
201Y	9.00	129.0					

203L	8.30	121.2	8.30	121.5	0.00	-0.3	0.05
204S	8.47	108.6	8.46	108.7	0.01	0.0	0.01
205R	7.60	124.6	7.56	124.7	0.04	-0.1	0.04
206T	8.27	114.5	8.11	115.0	0.16	-0.5	0.17
207S	7.43	115.7	7.32	115.8	0.11	-0.1	0.11
208A	8.58	124.5	8.71	125.9	-0.13	-1.4	0.25
211V	8.43	119.3	8.57	119.7	-0.14	-0.4	0.15
212A	8.14	126.8	8.28	125.6	-0.14	1.1	0.22
213D	7.91	114.4					
215D	7.86	118.8	7.85	119.0	0.01	-0.2	0.04
216L	8.61	122.4	8.56	122.3	0.05	0.1	0.05
217A	7.84	118.7	7.84	118.7	0.00	0.0	0.01
218E	7.59	117.8	7.58	117.9	0.01	-0.1	0.02
221T	7.41	117.9	7.41	117.8	0.00	0.0	0.01
222S	8.84	122.3	8.79	122.2	0.05	0.1	0.05
223I	8.25	120.5	8.26	120.7	-0.01	-0.1	0.02
224E	7.85	122.1	7.84	122.1	0.01	0.0	0.01
225R	8.13	117.9	8.11	117.9	0.02	0.0	0.02
226I	7.77	119.1	7.77	119.1	0.00	0.0	0.00
227Q	8.27	119.1	8.30	119.1	-0.03	0.0	0.03
228Q	8.03	113.6	8.04	113.7	-0.01	-0.1	0.01
229H	7.49	116.9	7.48	116.8	0.01	0.1	0.02
230Y	7.45	115.1	7.42	115.1	0.03	0.0	0.03
232E	8.36	117.1	8.35	117.1	0.01	0.0	0.01
233A	7.04	122.0	7.01	122.0	0.03	0.0	0.03
234Q	8.65	120.4	8.66	120.5	-0.01	-0.1	0.01
235F	7.52	117.7	7.53	117.5	-0.01	0.2	0.04
236V	9.11	125.2	9.11	125.0	0.00	0.2	0.03
237I	9.08	126.6	9.14	126.8	-0.06	-0.2	0.07
239G	7.31	108.4	7.33	108.2	-0.02	0.2	0.04
240H	7.44	114.4					
241G	9.20	112.2	9.19	108.3	0.01	3.9	0.60
242L	7.66	118.9	7.65	119.0	0.01	-0.1	0.02
244G	8.41	109.2	8.44	109.2	-0.03	0.0	0.03
245G	8.36	114.8	8.38	114.8	-0.02	0.0	0.02
246L	8.76	120.8	8.75	120.8	0.01	0.0	0.01
247D	9.47	120.5	9.44	120.3	0.03	0.1	0.04
248L	8.23	120.5	8.22	120.5	0.01	0.1	0.02
249L	8.06	122.7	8.06	122.5	0.00	0.2	0.03
250K	6.80	120.0	6.81	120.0	-0.01	0.0	0.01
251H	8.07	117.8	8.11	117.4	-0.04	0.4	0.07
252T	7.81	114.0	7.79	114.2	0.02	-0.1	0.03
253T	7.60	116.7	7.61	116.7	-0.01	0.0	0.01
254N	7.73	119.5	7.72	119.5	0.01	0.1	0.01
256V	7.99	119.1					
257K	8.33	119.5	8.31	119.4	0.02	0.1	0.03
258A	7.60	120.5	7.62	120.5	-0.02	0.0	0.02
259H	7.68	118.3	7.66	118.1	0.02	0.2	0.04
260T	7.67	112.3	7.69	112.1	-0.02	0.2	0.04
261N	8.01	120.3	8.01	120.3	0.00	0.0	0.00

262R	7.89	120.7	7.90	120.6	-0.01	0.0	0.01
263S	8.20	116.6	8.21	116.6	-0.01	0.0	0.01
264V	7.98	121.5	7.97	121.5	0.01	0.0	0.01
265V	8.08	124.0	8.09	123.9	-0.01	0.0	0.01
266E	7.89	129.9	7.88	129.9	0.01	0.0	0.01

Table S21. *Mean and standard deviation of the chemical shift perturbations for VIM-2 in the presence of 10 equivalents of compound* ***5g****.*

**Compound**	**Mean (ppm)**	**Standard deviation (ppm)**
**5g**	0.04	0.06

Table S22. *Chemical shift perturbations for VIM-2 in the presence of compound* ***5i****.*

**Compound 5i**	Free VIM-2		VIM-2 in the presence of 10 equiv. of ligand		Difference		**CSP (ppm)**
	**^{1}H (ppm)**	**^{15}N (ppm)**	**^{1}H (ppm)**	**^{15}N (ppm)**	**Δ^{1}H (ppm)**	**Δ ^{15}N (ppm)**	
28D	8.45	123.2	8.45	123.2	0.00	0.0	0.00
29S	8.15	116.1	8.16	116.2	-0.01	0.0	0.01
30S	8.41	118.1	8.41	118.1	0.00	0.0	0.00
31G	8.33	110.4	8.33	110.4	0.00	0.0	0.00
32E	8.34	120.9	8.35	121.0	-0.01	0.0	0.01
33Y	8.50	127.6	8.53	127.9	-0.03	-0.3	0.05
35T	7.73	110.7	7.69	110.1	0.04	0.6	0.10
36V	8.41	116.8	8.47	117.3	-0.06	-0.6	0.11
37S	8.11	110.9	8.10	111.0	0.01	0.0	0.01
38E	7.59	118.3	7.52	118.1	0.07	0.2	0.08
39I	6.93	119.9	6.91	119.7	0.02	0.2	0.03
43E	7.89	119.1	7.87	119.3	0.02	-0.1	0.03
44V	8.17	115.8	8.15	115.3	0.02	0.5	0.08
45R	8.95	121.9	8.99	121.7	-0.04	0.1	0.04
46L	8.80	118.8	8.83	119.2	-0.03	-0.3	0.06
47Y	8.98	123.5	9.01	123.9	-0.03	-0.4	0.06
48Q	8.62	129.4	8.61	129.3	0.01	0.0	0.01
49I	8.40	129.7	8.44	129.7	-0.04	0.0	0.04
50A	7.86	119.3	7.87	119.0	-0.01	0.2	0.04
51D	8.38	119.8	8.39	119.9	-0.01	-0.1	0.02
52G	8.13	112.9	8.14	113.0	-0.01	-0.1	0.02
53V	7.19	117.5	7.20	117.4	-0.01	0.1	0.01
54W	9.64	128.5	9.65	128.7	-0.01	-0.2	0.03
55S	9.85	115.4	9.90	115.5	-0.05	-0.1	0.05
56H	7.67	118.6	7.65	118.4	0.02	0.2	0.04
57I	9.03	121.2	9.02	120.8	0.01	0.4	0.06
58A	8.53	128.0					
64G	8.34	104.1	8.38	103.6	-0.04	0.5	0.08
65A	7.73	124.4	7.72	124.3	0.01	0.1	0.02
66V	7.83	120.0	7.94	120.8	-0.11	-0.8	0.16

67Y	8.84	125.7	8.80	125.5	0.04	0.3	0.06
69S	8.98	115.9	9.04	116.4	-0.06	-0.5	0.10
70N	8.46	123.7					
71G	7.89	106.4	7.81	106.1	0.08	0.4	0.10
73I	9.46	123.1	9.47	122.9	-0.01	0.2	0.03
74V	9.40	126.0	9.40	126.0	0.00	0.0	0.00
75R	9.24	128.0	9.23	128.0	0.01	0.0	0.01
76D	9.06	129.3	9.06	129.3	0.00	0.0	0.00
77G	8.62	113.3	8.62	113.3	0.00	0.1	0.01
78D	8.85	129.6	8.84	129.5	0.01	0.0	0.01
79E	7.59	116.8	7.59	116.9	0.00	0.0	0.00
80L	8.67	116.4	8.67	116.4	0.00	0.0	0.00
81L	8.81	123.4	8.80	123.4	0.01	0.0	0.01
82L	8.82	128.4	8.81	128.5	0.01	-0.1	0.02
83L	9.54	126.7	9.58	126.8	-0.04	-0.1	0.04
84D	8.97	118.8	8.96	118.7	0.01	0.1	0.02
85T	6.67	104.0					
97A	7.46	121.9	7.41	121.9	0.05	0.0	0.05
98E	8.38	122.1	8.41	122.1	-0.03	0.1	0.03
99I	8.48	119.7	8.51	119.7	-0.03	0.0	0.03
100E	7.62	122.6	7.60	122.6	0.02	0.0	0.02
101K	7.40	116.1	7.40	116.1	0.00	0.0	0.00
102Q	8.31	113.3	8.34	113.3	-0.03	0.0	0.03
103I	8.01	117.2	8.04	117.2	-0.03	0.1	0.03
104G	8.17	104.8	8.19	104.8	-0.02	0.0	0.02
105L	6.08	117.0	6.09	117.0	-0.01	0.0	0.01
107V	8.73	122.3	8.72	122.3	0.01	0.0	0.01
108T	8.31	114.1	8.29	114.1	0.02	0.0	0.02
109R	7.51	121.4	7.51	121.3	0.00	0.1	0.01
110A	8.91	121.8	8.91	121.7	0.00	0.0	0.01
111V	8.70	120.9	8.71	121.1	-0.01	-0.2	0.03
112S	7.63	122.0	7.65	122.0	-0.02	0.0	0.02
124D	7.65	117.5	7.63	117.3	0.02	0.2	0.04
125V	7.64	123.7	7.68	123.8	-0.04	-0.1	0.05
126L	7.65	119.0	7.61	119.1	0.04	-0.1	0.04
127R	8.75	121.4	8.73	121.2	0.02	0.3	0.05
128A	7.96	122.9	7.98	122.9	-0.02	0.0	0.02
129A	7.37	119.6	7.39	119.6	-0.02	0.0	0.02
130G	7.80	106.5	7.78	106.4	0.02	0.1	0.02
131V	7.89	121.9	7.85	122.0	0.04	-0.2	0.05
132A	7.83	131.8	7.82	131.8	0.01	0.0	0.01
133T	8.23	116.1	8.25	116.2	-0.02	-0.1	0.03
134Y	9.08	120.1	9.11	120.2	-0.03	-0.1	0.04
135A	8.62	118.4	8.64	118.3	-0.02	0.0	0.02
136S	10.23	119.3	10.25	119.3	-0.02	0.0	0.02
138S	7.53	112.8	7.51	112.9	0.02	0.0	0.02
139T	8.14	120.9	8.14	121.0	0.00	0.0	0.00
140R	8.21	117.6	8.19	117.7	0.02	0.0	0.02
141R	7.68	119.3	7.69	119.3	-0.01	0.0	0.01
142L	8.00	119.9	7.98	119.9	0.02	0.0	0.02

143A	8.52	120.7	8.52	120.7	0.00	0.0	0.00
144E	7.52	118.1	7.50	118.1	0.02	0.1	0.02
145V	7.96	120.2	7.96	120.3	0.00	0.0	0.01
146E	7.65	117.6	7.67	117.8	-0.02	-0.2	0.04
147G	7.68	106.1	7.68	105.9	0.00	0.1	0.02
148N	8.13	119.8	8.14	119.9	-0.01	-0.2	0.03
149E	8.06	119.7	8.11	119.9	-0.05	-0.2	0.06
150I	8.05	115.5	8.07	115.6	-0.02	-0.1	0.02
152T	8.43	122.7	8.45	122.6	-0.02	0.1	0.02
153H	8.74	118.4	8.74	118.3	0.00	0.0	0.01
154S	8.62	116.6	8.62	116.7	0.00	-0.1	0.01
155L	7.88	125.9	7.88	125.8	0.00	0.0	0.00
156E	8.31	122.6	8.32	122.6	-0.01	0.0	0.01
157G	8.20	106.5	8.19	106.4	0.01	0.0	0.01
158L	8.09	119.5	8.10	119.5	-0.01	0.0	0.01
159S	8.17	113.9	8.14	113.8	0.03	0.1	0.03
160S	8.91	119.2	8.90	119.2	0.01	0.0	0.01
161S	8.67	120.4	8.67	120.4	0.00	0.0	0.00
162G	8.85	112.9	8.85	112.9	0.00	0.0	0.01
163D	7.99	123.7	8.00	123.7	-0.01	0.0	0.01
164A	7.92	120.6	7.91	120.5	0.01	0.1	0.01
165V	8.98	116.7	8.98	116.7	0.00	0.0	0.01
166R	8.76	124.9	8.76	124.8	0.00	0.1	0.01
167F	8.90	130.1	8.91	130.1	-0.01	0.0	0.01
168G	8.40	114.7	8.40	114.7	0.00	0.0	0.0
170V	7.50	110.6	7.51	110.6	-0.01	0.0	0.01
171E	9.52	121.8	9.51	121.8	0.01	0.0	0.01
172L	9.23	124.8	9.24	124.8	-0.01	0.0	0.01
173F	8.91	122.7	8.92	122.7	-0.01	0.0	0.01
174Y	8.43	127.7	8.43	127.8	0.00	0.0	0.01
177A	7.94	117.5	7.94	117.7	0.00	-0.2	0.03
178A	7.34	123.3	7.32	123.2	0.02	0.0	0.02
179H	6.15	124.4	6.18	124.4	-0.03	-0.1	0.03
180S	7.64	107.3	7.62	107.1	0.02	0.2	0.04
181T	9.66	114.9	9.67	114.8	-0.01	0.2	0.03
182D	10.09	117.4	10.08	117.6	0.01	-0.2	0.03
183N	6.42	116.2	6.42	116.3	0.00	-0.1	0.01
184L	8.84	119.7	8.85	119.7	-0.01	0.0	0.01
185V	9.07	113.2	9.08	113.5	-0.01	-0.2	0.04
186V	8.47	119.3	8.50	119.8	-0.03	-0.5	0.08
187Y	9.58	128.6	9.59	128.5	-0.01	0.0	0.01
188V	8.60	126.8	8.60	126.8	0.00	0.0	0.01
190S	7.66	113.1	7.64	112.9	0.02	0.1	0.03
191A	6.51	120.6	6.51	120.6	0.00	0.0	0.00
192S	7.53	112.0	7.53	112.0	0.00	0.0	0.00
193V	6.54	116.8	6.56	116.8	-0.02	0.0	0.02
194L	9.34	129.0	9.34	129.1	0.00	0.0	0.00
195Y	10.01	128.7	10.02	128.8	-0.01	-0.2	0.03
196G	8.60	112.0	8.59	112.0	0.01	0.0	0.01
198C	7.65	123.7	7.68	123.8	-0.03	-0.1	0.04

199A	6.50	115.6	6.41	115.2	0.09	0.3	0.10
200I	7.04	113.4	7.04	113.5	0.00	-0.2	0.03
201Y	8.99	129.0					
202E	8.52	116.5					
203L	8.30	121.3	8.30	121.4	0.00	-0.1	0.02
204S	8.51	108.7	8.44	108.7	0.07	0.0	0.07
205R	7.58	124.7	7.58	124.5	0.00	0.2	0.03
206T	8.27	114.7	8.16	112.2	0.11	2.5	0.40
207S	7.43	116.0					
208A	8.61	124.4	8.70	126.2	-0.09	-1.8	0.29
211V	8.45	119.4	8.47	119.9	-0.02	-0.5	0.09
212A	8.15	127.0	8.26	125.7	-0.11	1.3	0.23
213D	7.89	114.3	8.15	112.7	-0.26	1.7	0.37
215D	7.84	118.8	7.83	118.7	0.01	0.1	0.02
216L	8.63	122.5	8.61	122.4	0.02	0.0	0.02
217A	7.83	118.7	7.82	118.7	0.01	0.0	0.01
218E	7.58	117.8	7.58	118.1	0.00	-0.2	0.04
221T	7.39	117.9	7.40	117.9	-0.01	0.0	0.01
222S	8.84	122.5	8.84	122.4	0.00	0.0	0.00
223I	8.24	120.7	8.27	120.9	-0.03	-0.2	0.04
224E	7.84	122.0	7.84	122.0	0.00	0.0	0.00
225R	8.08	117.9	8.09	117.9	-0.01	-0.1	0.01
226I	7.76	119.1	7.75	119.1	0.01	0.0	0.01
227Q	8.30	119.2	8.30	119.1	0.00	0.1	0.01
228Q	8.03	113.7	8.04	113.7	-0.01	0.0	0.01
229H	7.48	117.0	7.48	116.9	0.00	0.0	0.01
230Y	7.43	115.2	7.42	115.1	0.01	0.0	0.01
232E	8.36	117.2	8.36	117.2	0.00	0.0	0.00
233A	7.03	122.1	7.02	122.0	0.01	0.0	0.01
234Q	8.65	120.8	8.66	120.8	-0.01	0.0	0.01
235F	7.52	117.5	7.52	117.3	0.00	0.2	0.03
236V	9.08	125.0	9.08	124.7	0.00	0.2	0.04
237I	9.11	126.8	9.13	126.8	-0.02	0.1	0.02
239G	7.33	108.4	7.35	108.1	-0.02	0.3	0.05
240H	7.41	114.5					
241G	9.28	112.3	9.21	108.3	0.07	4.0	0.61
242L	7.67	119.2	7.66	119.3	0.01	-0.1	0.02
244G	8.45	109.5	8.47	109.5	-0.02	0.0	0.02
245G	8.37	115.2	8.38	115.2	-0.01	0.0	0.01
246L	8.77	120.9	8.76	120.9	0.01	0.0	0.01
247D	9.46	120.5	9.45	120.5	0.01	0.0	0.01
248L	8.24	120.6	8.23	120.5	0.01	0.1	0.02
249L	8.05	122.8	8.05	122.7	0.00	0.1	0.02
250K	6.82	119.9	6.80	119.9	0.02	0.0	0.02
251H	8.07	117.5	8.08	117.5	-0.01	0.0	0.01
252T	7.79	114.2	7.80	114.2	-0.01	0.0	0.01
253T	7.60	116.8	7.61	116.8	-0.01	0.0	0.01
254N	7.73	119.4	7.70	119.3	0.03	0.1	0.03
256V	8.00	119.1					
257K	8.33	119.6	8.31	119.5	0.02	0.0	0.02

258A	7.57	120.5	7.57	120.5	0.00	-0.1	0.01
259H	7.66	118.5	7.69	118.7	-0.03	-0.1	0.04
260T	7.66	112.7	7.70	112.5	-0.04	0.2	0.05
261N	8.02	120.4	8.03	120.5	-0.01	-0.1	0.02
262R	7.90	120.9	7.90	120.8	0.00	0.1	0.01
263S	8.21	116.8	8.22	116.9	-0.01	0.0	0.01
264V	8.00	121.8	8.00	121.7	0.00	0.1	0.01
265V	8.12	124.3	8.12	124.3	0.00	0.0	0.00
266E	7.92	130.2	7.92	130.2	0.00	0.0	0.00

Table S23. *Mean and standard deviation of the chemical shift perturbations for VIM-2 in the presence of 10 equivalents of compound* ***5i****.*

Compound	Mean (ppm)	Standard deviation (ppm)
**5i**	0.04	0.07

Table S24. *Chemical shift perturbations for VIM-2 in the presence of compound* ***5j****.*

Compound 5j	Free VIM-2		VIM-2 in the presence of 10 equiv. of ligand		Difference		CSP (ppm)
	^{1}H (ppm)	^{15}N (ppm)	^{1}H (ppm)	^{15}N (ppm)	Δ^{1}H (ppm)	$\Delta\ ^{15}$N (ppm)	
28D	8.46	123.2	8.46	123.2	0.00	0.0	0.01
29S	8.17	116.1	8.17	116.1	0.00	0.0	0.00
30S	8.43	118.1	8.43	118.1	0.00	0.0	0.00
31G	8.35	110.4	8.35	110.4	0.00	0.0	0.00
32E	8.36	121.0	8.37	121.0	-0.01	0.0	0.01
33Y	8.51	127.6	8.54	127.9	-0.03	-0.3	0.06
35T	7.76	110.7	7.70	110.1	0.06	0.6	0.11
36V	8.42	117.0	8.48	117.4	-0.06	-0.4	0.08
37S	8.13	111.0	8.12	111.0	0.01	0.0	0.01
38E	7.62	118.3	7.55	118.1	0.07	0.2	0.07
39I	6.96	119.9	6.93	119.7	0.03	0.2	0.04
43E	7.91	119.2	7.89	119.1	0.02	0.1	0.03
44V	8.20	115.8	8.17	115.3	0.03	0.5	0.08
45R	8.98	121.9	9.02	121.8	-0.04	0.2	0.05
46L	8.83	119.0	8.82	119.0	0.01	0.0	0.01
47Y	9.01	123.5	9.06	123.9	-0.05	-0.4	0.08
48Q	8.64	129.4	8.63	129.4	0.01	0.0	0.01
49I	8.43	129.7	8.45	129.7	-0.02	0.0	0.02
50A	7.88	119.3	7.90	119.3	-0.02	0.0	0.02
51D	8.40	119.9	8.40	120.0	0.00	-0.1	0.01
52G	8.14	113.0	8.15	113.0	-0.01	-0.1	0.01
53V	7.22	117.5	7.22	117.4	0.00	0.1	0.01
54W	9.67	128.5	9.67	128.7	0.00	-0.2	0.02
55S	9.88	115.5	9.91	115.6	-0.03	-0.1	0.03
56H	7.69	118.7	7.73	118.8	-0.04	-0.1	0.04
57I	9.04	121.2	9.02	120.9	0.02	0.3	0.05

58A	8.57	128.1					
64G	8.36	104.1	8.40	103.6	-0.04	0.5	0.09
65A	7.75	124.4	7.75	124.2	0.00	0.2	0.03
66V	7.86	120.1	7.99	120.9	-0.13	-0.9	0.19
67Y	8.86	125.9	8.83	125.4	0.03	0.5	0.09
69S	9.01	116.0	9.07	116.5	-0.06	-0.5	0.10
70N	8.50	123.7					
71G	7.91	106.4	7.83	106.0	0.08	0.4	0.10
73I	9.49	123.1	9.49	123.0	0.00	0.2	0.02
74V	9.42	126.0	9.42	126.0	0.00	0.0	0.00
75R	9.26	128.0	9.25	128.0	0.01	0.0	0.01
76D	9.08	129.3	9.08	129.3	0.00	0.0	0.00
77G	8.64	113.3	8.64	113.3	0.00	0.1	0.01
78D	8.86	129.6	8.85	129.5	0.01	0.1	0.01
79E	7.61	116.8	7.61	116.9	0.00	0.0	0.00
80L	8.70	116.5	8.70	116.5	0.00	0.0	0.00
81L	8.84	123.4	8.83	123.4	0.01	0.0	0.01
82L	8.83	128.4	8.83	128.5	0.00	0.0	0.01
83L	9.58	126.8	9.60	126.8	-0.02	0.0	0.02
84D	8.99	118.8	8.98	118.7	0.01	0.1	0.02
85T	6.71	104.1					
97A	7.48	122.0	7.43	121.9	0.05	0.0	0.05
98E	8.41	122.1	8.44	122.0	-0.03	0.1	0.03
99I	8.51	119.7	8.54	119.8	-0.03	0.0	0.03
100E	7.64	122.6	7.63	122.6	0.01	0.0	0.01
101K	7.43	116.1	7.42	116.1	0.01	0.0	0.01
102Q	8.34	113.3	8.36	113.4	-0.02	0.0	0.02
103I	8.05	117.3	8.10	117.1	-0.05	0.2	0.06
104G	8.21	104.8	8.22	104.8	-0.01	0.0	0.01
105L	6.11	117.0	6.12	117.1	-0.01	0.0	0.01
107V	8.74	122.3	8.74	122.3	0.00	0.0	0.00
108T	8.33	114.2	8.31	114.2	0.02	0.0	0.02
109R	7.54	121.4	7.54	121.3	0.00	0.1	0.01
110A	8.93	121.8	8.93	121.8	0.00	0.0	0.00
111V	8.73	120.9	8.75	121.0	-0.02	-0.1	0.02
112S	7.66	122.1	7.68	122.0	-0.02	0.0	0.02
124D	7.68	117.5	7.66	117.4	0.02	0.1	0.03
125V	7.66	123.6	7.70	123.7	-0.04	-0.1	0.04
126L	7.67	119.0	7.62	119.2	0.05	-0.2	0.06
127R	8.77	121.4	8.74	121.1	0.03	0.3	0.05
128A	7.98	122.9	8.00	122.9	-0.02	0.0	0.02
129A	7.40	119.6	7.42	119.6	-0.02	0.0	0.02
130G	7.82	106.6	7.81	106.4	0.01	0.1	0.02
131V	7.92	121.8	7.88	121.9	0.04	-0.1	0.04
132A	7.86	131.8	7.85	131.8	0.01	0.0	0.01
133T	8.24	116.1	8.27	116.1	-0.03	-0.1	0.03
134Y	9.11	120.1	9.13	120.3	-0.02	-0.2	0.03
135A	8.64	118.4	8.66	118.4	-0.02	0.0	0.02
136S	10.25	119.3	10.25	119.3	0.00	0.0	0.01
138S	7.57	112.9	7.54	112.9	0.03	0.0	0.03

139T	8.16	121.0	8.16	121.0	0.00	-0.1	0.01
140R	8.23	117.7	8.21	117.7	0.02	0.0	0.02
141R	7.70	119.3	7.70	119.3	0.00	0.0	0.00
142L	8.02	119.9	8.00	119.9	0.02	0.0	0.02
143A	8.54	120.7	8.54	120.7	0.00	0.0	0.00
144E	7.55	118.1	7.53	118.1	0.02	0.0	0.02
145V	7.99	120.2	7.98	120.2	0.01	0.0	0.01
146E	7.68	117.6	7.70	117.8	-0.02	-0.2	0.04
147G	7.71	106.1	7.70	106.0	0.01	0.1	0.02
148N	8.16	119.8	8.17	120.0	-0.01	-0.2	0.03
149E	8.10	119.7	8.13	120.0	-0.03	-0.3	0.06
150I	8.06	115.6	8.08	115.6	-0.02	-0.1	0.02
152T	8.46	122.7	8.47	122.4	-0.01	0.2	0.04
153H	8.76	118.4	8.76	118.3	0.00	0.2	0.02
154S	8.64	116.7	8.65	116.8	-0.01	-0.1	0.03
155L	7.91	125.9	7.91	125.8	0.00	0.0	0.00
156E	8.33	122.6	8.34	122.6	-0.01	0.0	0.01
157G	8.22	106.5	8.21	106.5	0.01	0.0	0.01
158L	8.11	119.6	8.12	119.6	-0.01	0.0	0.01
159S	8.22	114.0	8.19	113.9	0.03	0.1	0.03
160S	8.93	119.2	8.92	119.2	0.01	0.0	0.01
161S	8.69	120.3	8.69	120.3	0.00	0.0	0.00
162G	8.87	112.9	8.86	112.9	0.01	0.0	0.01
163D	8.02	123.7	8.02	123.7	0.00	0.0	0.00
164A	7.94	120.6	7.93	120.6	0.01	0.0	0.01
165V	9.01	116.7	9.01	116.7	0.00	0.0	0.01
166R	8.78	125.0	8.77	124.9	0.01	0.1	0.02
167F	8.93	130.2	8.93	130.2	0.00	0.0	0.00
168G	8.42	114.7	8.42	114.7	0.00	0.0	0.00
170V	7.53	110.6	7.53	110.6	0.00	0.0	0.00
171E	9.54	121.8	9.53	121.8	0.01	0.0	0.01
172L	9.26	124.8	9.25	124.8	0.01	0.0	0.01
173F	8.94	122.7	8.95	122.7	-0.01	0.0	0.01
174Y	8.46	127.7	8.46	127.8	0.00	0.0	0.01
177A	7.96	117.6	7.96	117.7	0.00	-0.1	0.02
178A	7.36	123.3	7.35	123.3	0.01	0.0	0.01
179H	6.19	124.4	6.21	124.4	-0.02	0.0	0.02
180S	7.66	107.3	7.65	107.1	0.01	0.3	0.04
181T	9.67	114.9	9.59	114.8	0.08	0.1	0.08
182D	10.12	117.4	10.09	117.6	0.03	-0.2	0.04
183N	6.45	116.2	6.45	116.3	0.00	-0.1	0.01
184L	8.88	119.7	8.89	119.8	-0.01	-0.1	0.01
185V	9.10	113.4	9.09	113.5	0.01	-0.2	0.03
186V	8.51	119.4	8.54	119.7	-0.03	-0.3	0.05
187Y	9.60	128.6	9.60	128.6	0.00	0.0	0.01
188V	8.63	126.8	8.63	126.9	0.00	0.0	0.01
190S	7.68	113.1	7.65	112.9	0.03	0.2	0.04
191A	6.54	120.7	6.53	120.7	0.01	0.0	0.01
192S	7.55	112.0	7.56	112.0	-0.01	0.0	0.01
193V	6.58	116.9	6.59	116.9	-0.01	0.0	0.01

194L	9.36	129.1	9.37	129.1	-0.01	0.0	0.01
195Y	10.03	128.7	10.04	128.8	-0.01	-0.1	0.02
196G	8.62	112.1	8.60	112.0	0.02	0.0	0.02
198C	7.69	123.6	7.70	123.7	-0.01	-0.1	0.02
199A	6.52	115.6	6.44	115.2	0.08	0.4	0.10
200I	7.07	113.4	7.04	113.6	0.03	-0.1	0.04
201Y							
202E	8.52	116.4					
203L	8.33	121.3	8.37	121.4	-0.04	-0.1	0.04
204S	8.51	108.7	8.48	108.8	0.03	0.0	0.03
205R	7.61	124.8	7.62	124.6	-0.01	0.2	0.03
206T	8.29	114.7	8.18	112.2	0.11	2.5	0.39
207S	7.46	115.9					
208A	8.63	124.6	8.73	126.2	-0.10	-1.6	0.27
209G	7.50	106.0					
211V	8.46	119.5	8.48	120.0	-0.02	-0.5	0.08
212A	8.17	127.0	8.28	125.7	-0.11	1.3	0.22
213D	7.93	114.3					
215D	7.87	118.9	7.88	118.9	-0.01	0.0	0.01
216L	8.63	122.5	8.62	122.5	0.01	0.0	0.01
217A	7.85	118.8	7.85	118.8	0.00	0.0	0.00
218E	7.61	117.9	7.61	118.1	0.00	-0.3	0.04
221T	7.42	117.9	7.42	117.9	0.00	0.0	0.00
222S	8.87	122.5	8.86	122.4	0.01	0.0	0.01
223I	8.28	120.6	8.30	120.9	-0.02	-0.2	0.04
224E	7.87	122.0	7.86	122.0	0.01	0.0	0.01
225R	8.12	117.9	8.13	118.0	-0.01	0.0	0.01
226I	7.78	119.1	7.78	119.2	0.00	-0.1	0.01
227Q	8.32	119.2	8.31	119.2	0.01	0.0	0.01
228Q	8.05	113.7	8.07	113.8	-0.02	0.0	0.02
229H	7.51	117.0	7.50	116.9	0.01	0.1	0.02
230Y	7.46	115.2	7.44	115.2	0.02	0.0	0.02
232E	8.37	117.2	8.38	117.2	-0.01	0.0	0.01
233A	7.05	122.1	7.04	122.1	0.01	0.0	0.01
234Q	8.67	120.7	8.68	120.7	-0.01	0.0	0.01
235F	7.55	117.6	7.55	117.3	0.00	0.3	0.05
236V	9.11	125.1	9.11	124.8	0.00	0.3	0.04
237I	9.12	126.8	9.17	126.8	-0.05	0.0	0.05
239G	7.34	108.4	7.35	108.1	-0.01	0.3	0.05
240H	7.45	114.5					
241G	9.26	112.2	9.23	108.4	0.03	3.8	0.59
242L	7.70	119.2	7.68	119.3	0.02	-0.1	0.03
244G	8.46	109.4	8.49	109.4	-0.03	0.0	0.03
245G	8.39	115.1	8.40	115.1	-0.01	0.0	0.01
246L	8.79	121.0	8.78	121.0	0.01	0.0	0.01
247D	9.47	120.5	9.45	120.5	0.02	0.0	0.02
248L	8.27	120.6	8.25	120.5	0.02	0.1	0.03
249L	8.08	122.8	8.08	122.7	0.00	0.1	0.02
250K	6.83	119.9	6.84	119.9	-0.01	0.0	0.01
251H	8.09	117.5	8.09	117.5	0.00	0.0	0.01

252T	7.82	114.2	7.83	114.3	-0.01	-0.2	0.03
253T	7.62	116.8	7.65	116.9	-0.03	-0.1	0.03
254N	7.75	119.4	7.72	119.3	0.03	0.1	0.03
256V	8.02	119.1					
257K	8.35	119.6	8.32	119.5	0.03	0.1	0.03
258A	7.59	120.5	7.58	120.6	0.01	-0.1	0.02
259H	7.69	118.6	7.68	118.3	0.01	0.2	0.04
260T	7.68	112.5	7.71	112.5	-0.03	0.0	0.03
261N	8.04	120.4	8.07	120.5	-0.03	-0.1	0.03
262R	7.92	120.9	7.95	120.9	-0.03	0.0	0.03
263S	8.23	116.8	8.24	116.9	-0.01	-0.1	0.01
264V	8.02	121.7	8.01	121.7	0.01	0.1	0.02
265V	8.13	124.3	8.13	124.2	0.00	0.1	0.01
266E	7.93	130.2	7.93	130.1	0.00	0.0	0.00

Table S25. *Mean and standard deviation of the chemical shift perturbations for VIM-2 in the presence of 10 equivalents of compound* ***5j****.*

Compound	Mean (ppm)	Standard deviation (ppm)
**5j**	0.04	0.08

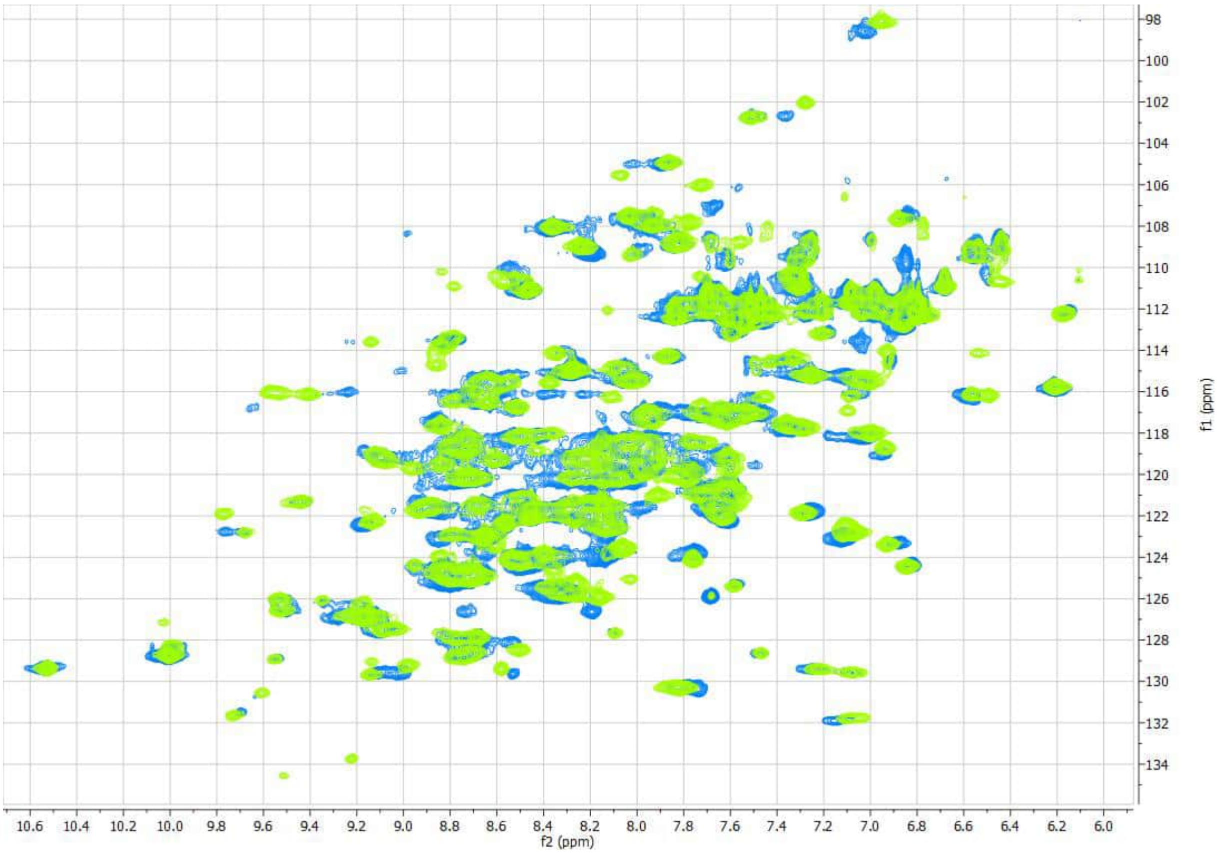

Figure S49*. Superimposition of the $^{1}H,^{15}N$-HSQC spectrum (600 MHz, pH 7.0, 25 °C) of NDM-1 in the absence (blue) and in the presence (green) of 10 molar equivalent of compound* ***5f****. Buffer: 20 mM HEPES, 0.1 mM $ZnCl_2$, with 15% D_2O.*

Table S26. *Chemical shift perturbations for NDM-1 in the presence of compound* ***5f***.

**Compound 5f**	Free NDM-1		NDM-1 in the presence of 10 equiv. of ligand		Difference		**CSP (ppm)**
	**^{1}H (ppm)**	**^{15}N (ppm)**	**^{1}H (ppm)**	**^{15}N (ppm)**	**Δ^{1}H (ppm)**	**Δ ^{15}N (ppm)**	
39M	8.46	121.7	8.46	121.6	0	0.1	0.01
40E	8.65	123.0	8.65	123.0	0	-0.1	0.01
41T	8.28	115.1	8.28	115.0	0	0.0	0.01
42G	8.47	111.1	8.47	111.1	0	0.0	0.00
43D	8.15	121.5	8.15	121.5	0	0.0	0.00
44Q	9.18	122.4	9.13	122.3	0.05	0.1	0.05
45R	8.82	125.2	8.83	124.7	-0.01	0.5	0.07
46F	8.68	128.0	8.69	127.9	-0.01	0.2	0.03
47G	8.64	116.5	8.64	116.5	0	0.0	0.00
48D	9.28	126.9	9.26	126.9	0.02	0.0	0.02
49L	8.45	124.3	8.40	123.9	0.05	0.4	0.09
50V	8.14	119.2					
51F	8.84	123.1	8.79	122.9	0.05	0.2	0.06
52R	8.53	121.0	8.50	121.1	0.03	-0.1	0.04
53Q	9.64	130.7	9.62	130.6	0.02	0.1	0.03
54L	8.85	127.6	8.84	127.7	0.01	0.0	0.01
55A	8.31	121.7	8.29	121.7	0.02	0.0	0.02
57N	7.99	109.2	8.03	109.4	-0.04	-0.3	0.06
58V	7.05	116.1	7.09	116.2	-0.04	-0.1	0.04
59W	10.01	128.8	10.00	128.7	0.01	0.0	0.01
60Q	9.68	122.8	9.68	122.8	0	0.0	0.01
61H	8.11	127.6	8.09	127.7	0.02	-0.1	0.03
62T	8.54	118.3	8.45	118.1	0.09	0.1	0.09
63S	8.50	118.8	8.42	118.8	0.08	0.0	0.08
64Y	9.17	118.8	9.12	119.0	0.05	-0.2	0.06
65L	7.84	124.0	7.76	123.7	0.08	0.3	0.09
66D	8.19	126.6	8.16	125.9	0.03	0.7	0.11
67M	8.33	125.8	8.26	125.3	0.07	0.5	0.11
70F	8.14	118.7					
71G	8.19	109.3	8.24	109.0	-0.05	0.3	0.07
72A	8.14	123.9	8.06	123.6	0.08	0.3	0.10
73V	8.74	126.6	8.85	126.6	-0.11	0.1	0.11
74A	8.53	129.6	8.58	129.4	-0.05	0.2	0.06
75S	9.02	115.0	8.86	114.7	0.16	0.3	0.17
76N	9.05	129.6	9.14	129.0	-0.09	0.6	0.13
77G	7.95	108.0	7.93	107.9	0.02	0.0	0.02
78L	8.81	116.3	8.79	116.5	0.02	-0.2	0.03
79I	9.42	121.3	9.44	121.3	-0.02	0.0	0.02
80V	9.51	126.5	9.53	126.6	-0.02	-0.1	0.02
81R	9.54	128.9	9.55	128.9	-0.01	0.0	0.01
82D	9.02	129.6	8.97	129.2	0.05	0.4	0.07
83G	8.82	113.6	8.83	113.8	-0.01	-0.2	0.03
84G	8.64	116.5	8.64	116.5	0	0.0	0.00

85R	7.65	117.2	7.72	116.7	-0.07	0.5	0.11
86V	8.81	118.9	8.78	118.6	0.03	0.2	0.05
87L	9.52	126.1	9.53	126.0	-0.01	0.1	0.02
88V	8.13	123.0	8.13	122.5	0	0.5	0.07
89V	9.16	126.9	9.17	126.9	-0.01	0.0	0.01
90D	9.10	119.2	9.09	119.3	0.01	-0.1	0.02
91T	7.57	106.2	7.72	106.0	-0.15	0.2	0.15
92A	6.57	113.7	6.54	114.2	0.03	-0.5	0.08
93W	7.05	113.6	6.93	114.1	0.12	-0.5	0.14
94T	7.03	98.5	6.95	98.1	0.08	0.4	0.10
95D	9.35	126.2	9.35	126.1	0	0.1	0.01
96D	8.71	120.2	8.72	120.2	-0.01	0.0	0.01
97Q	7.80	117.0	7.71	117.1	0.09	0.0	0.09
98T	7.98	117.2	7.94	117.3	0.04	-0.1	0.04
99A	8.83	124.6	8.84	124.0	-0.01	0.6	0.10
100Q	7.56	117.2	7.61	117.5	-0.05	-0.3	0.07
101I	7.72	120.8	7.72	121.0	0	-0.2	0.03
102L	7.50	116.3	7.45	116.2	0.05	0.1	0.05
103N	8.15	120.3	8.12	120.2	0.03	0.1	0.03
104W	8.41	125.5	8.35	125.4	0.06	0.1	0.06
105I	8.42	118.1	8.36	118.0	0.06	0.0	0.06
106K	7.62	120.8	7.59	121.1	0.03	-0.3	0.06
107Q	7.26	115.2	7.25	115.2	0.01	0.0	0.01
108E	8.04	115.4	8.03	115.5	0.01	-0.1	0.02
109I	7.34	117.8	7.28	117.8	0.06	0.0	0.06
110N	8.67	115.4	8.65	115.5	0.02	-0.1	0.02
111L	6.22	115.8	6.20	115.8	0.02	0.0	0.02
113V	8.45	122.0	8.45	122.1	0	0.0	0.01
114A	9.09	129.6	9.14	129.7	-0.05	-0.1	0.05
115L	7.31	110.6	7.33	110.4	-0.02	0.2	0.04
116A	8.77	120.3	8.74	120.2	0.03	0.1	0.04
117V	8.85	121.8	8.88	121.5	-0.03	0.2	0.05
118V	8.15	119.3					
119T	10.41	112.7	10.35	113.1	0.06	-0.4	0.08
120H	6.84	107.4	6.88	107.7	-0.04	-0.2	0.05
121A	8.69	121.6	8.68	121.5	0.01	0.0	0.01
122H	7.15	118.2	7.10	116.9	0.05	1.2	0.20
123Q	9.51	123.3	9.44	122.9	0.07	0.4	0.09
124D	9.00	108.4	8.78	110.9	0.22	-2.6	0.45
127G	8.08	112.4	8.12	112.1	-0.04	0.3	0.06
128G	8.02	105.0	8.07	105.5	-0.05	-0.5	0.10
129M	6.60	116.2	6.56	116.1	0.04	0.1	0.04
130D	8.87	117.5	8.84	117.7	0.03	-0.2	0.04
131A	7.64	120.9	7.64	121.0	0	0.0	0.01
132L	7.10	115.3	7.03	115.4	0.07	-0.1	0.07
133H	8.30	120.3	8.28	120.2	0.02	0.1	0.02
134A	8.96	124.4	8.95	124.4	0.01	0.0	0.01
135A	7.02	118.1	7.00	118.0	0.02	0.1	0.02
136G	7.96	107.8	7.93	107.5	0.03	0.3	0.06
137I	7.61	122.3	7.64	122.1	-0.03	0.1	0.04

138A	7.79	130.3	7.82	130.3	-0.03	0.0	0.03
139T	7.31	109.4	7.28	109.7	0.03	-0.3	0.05
140Y	8.89	119.8	8.96	119.7	-0.07	0.1	0.07
141A	8.21	119.3					
142N	10.31	120.4	10.41	120.3	-0.1	0.1	0.10
143A	9.43	134.2	9.51	134.6	-0.08	-0.4	0.10
144L	7.36	114.6	7.33	114.3	0.03	0.2	0.05
145S	7.52	114.5	7.50	114.6	0.02	-0.1	0.02
146N	7.31	117.8	7.31	117.8	0	0.0	0.00
147Q	7.49	119.5	7.50	119.7	-0.01	-0.2	0.03
148L	7.77	118.6	7.70	118.5	0.07	0.1	0.07
149A	8.01	120.4	8.07	120.2	-0.06	0.2	0.07
151Q	7.48	117.1	7.53	117.1	-0.05	0.0	0.05
152E	7.56	117.2	7.65	117.2	-0.09	0.0	0.09
153G	7.67	107.1	7.78	107.9	-0.11	-0.8	0.16
154M	7.98	118.8					
155V	8.33	124.0	8.36	124.6	-0.03	-0.6	0.10
156A	7.75	123.7	7.76	124.2	-0.01	-0.4	0.07
157A	7.63	119.1	7.61	119.2	0.02	-0.1	0.03
158Q	8.57	118.3	8.50	118.2	0.07	0.1	0.07
159H	7.85	114.3	7.87	114.3	-0.02	0.0	0.02
160S	9.10	119.0	9.09	119.1	0.01	-0.1	0.02
161L	9.20	126.4	9.17	126.3	0.03	0.0	0.03
162T	7.56	112.9	7.54	112.8	0.02	0.1	0.03
163F	8.73	118.3	8.73	118.3	0	0.0	0.01
164A	8.76	125.1	8.79	124.9	-0.03	0.3	0.05
166N	7.66	111.7	7.64	111.7	0.02	0.0	0.02
167G	8.04	107.6	8.03	107.5	0.01	0.1	0.02
168W	7.66	121.6	7.66	121.6	0	0.0	0.01
169V	8.15	122.2	8.14	122.5	0.01	-0.3	0.04
170E	8.71	128.7	8.70	128.7	0.01	0.0	0.01
172A	8.73	118.8	8.73	118.8	0	0.0	0.00
173T	7.89	105.0	7.86	105.0	0.03	0.0	0.03
174A	6.83	124.4	6.84	124.5	-0.01	-0.1	0.02
176N	8.70	116.1	8.68	116.0	0.02	0.2	0.03
177F	8.12	122.7	8.15	122.3	-0.03	0.4	0.07
178G	8.37	108.1	8.36	108.1	0.01	0.0	0.01
180L	7.76	119.7	7.79	120.1	-0.03	-0.4	0.07
181K	9.09	127.5	9.08	127.5	0.01	0.0	0.01
182V	8.48	124.4	8.54	124.1	-0.06	0.3	0.07
183F	8.79	128.0	8.77	127.9	0.02	0.1	0.03
184Y	9.25	134.0	9.22	133.7	0.03	0.3	0.05
188G	7.52	108.0	7.55	108.8	-0.03	-0.7	0.12
189H	7.14	131.9	7.07	131.8	0.07	0.1	0.07
190T	7.36	102.7	7.28	102.1	0.08	0.6	0.13
191S	9.37	116.2	9.54	116.1	-0.17	0.1	0.17
192D	9.21	113.7	9.15	113.7	0.06	0.0	0.06
193N	6.55	116.3	6.49	116.2	0.06	0.1	0.06
194I	8.76	113.5	8.79	113.4	-0.03	0.1	0.04
195T	8.86	110.8	8.84	110.2	0.02	0.6	0.09

196V	8.38	116.1	8.38	115.6	0	0.5	0.08
197G	9.64	116.7	9.58	116.1	0.06	0.7	0.12
198I	6.94	119.0	6.94	118.7	0	0.3	0.04
199D	9.71	131.5	9.73	131.7	-0.02	-0.2	0.03
201T	7.84	109.0	7.82	108.9	0.02	0.1	0.03
202D	8.85	119.4	8.83	119.5	0.02	0.0	0.02
203I	8.29	119.4	8.24	119.3	0.05	0.1	0.05
204A	8.54	128.1	8.50	128.5	0.04	-0.4	0.07
205F	9.77	122.8	9.77	122.0	0	0.8	0.12
206G	8.89	112.4					
209L	6.87	116.0					
211K	8.14	124.8	8.03	125.1	0.11	-0.3	0.12
212D	6.85	112.8	6.85	112.8	0	0.1	0.01
213S	8.68	110.1	8.60	110.4	0.08	-0.3	0.09
214K	8.09	119.4					
215A	7.68	125.9	7.68	125.9	0	0.0	0.01
217S	7.43	111.8	7.43	111.9	0	-0.2	0.03
222G	8.55	110.0	8.57	110.7	-0.02	-0.6	0.10
223D	8.14	118.4					
224A	7.25	121.8	7.29	121.9	-0.04	-0.1	0.04
225D	8.33	120.3	8.28	120.2	0.05	0.1	0.05
226T	8.31	114.0	8.35	114.2	-0.04	-0.2	0.05
227E	7.98	121.6	7.91	121.0	0.07	0.7	0.12
228H	7.60	113.0	7.59	113.3	0.01	-0.3	0.05
229Y	7.32	120.8	7.37	120.8	-0.05	0.0	0.05
230A	8.94	121.4	8.94	121.8	0	-0.4	0.06
231A	7.97	119.4					
232S	8.58	119.4	8.61	119.3	-0.03	0.1	0.03
233A	8.66	123.8	8.61	123.5	0.05	0.3	0.07
234R	8.06	114.9	8.10	114.9	-0.04	0.0	0.04
235A	8.68	124.8	8.71	124.9	-0.03	-0.1	0.04
236F	7.96	118.7					
237G	7.50	102.7	7.51	102.8	-0.01	-0.1	0.02
238A	7.62	120.8	7.61	120.7	0.01	0.1	0.01
239A	7.11	122.9	7.11	122.4	0	0.5	0.07
240F	6.16	112.1	6.17	112.3	-0.01	-0.2	0.03
242K	8.52	116.7	8.52	116.8	0	-0.1	0.01
243A	6.88	123.3	6.93	123.4	-0.05	-0.2	0.06
244S	8.20	116.1	8.10	116.2	0.1	-0.1	0.10
245M	8.33	125.8	8.30	125.8	0.03	0.0	0.03
246I	9.13	127.6	9.04	127.5	0.09	0.1	0.09
247V	8.77	128.3	8.75	128.3	0.02	0.0	0.02
248M	10.00	128.3	10.00	128.2	0	0.0	0.01
249S	7.44	112.3	7.43	112.2	0.01	0.1	0.02
251S	6.97	112.1	7.04	112.2	-0.07	-0.1	0.07
252A	8.27	125.7	8.30	125.8	-0.03	0.0	0.03
254D	8.71	123.0	8.75	123.0	-0.04	0.0	0.04
255S	9.24	116.0	9.41	116.1	-0.17	-0.1	0.17
256R	8.59	115.9	8.61	115.7	-0.02	0.2	0.03
258A	7.99	119.5					

259I	7.81	118.7	7.77	118.4	0.04	0.3	0.06
260T	7.19	113.1	7.21	113.2	-0.02	-0.1	0.03
261H	8.65	121.5	8.68	121.5	-0.03	0.0	0.03
262T	8.00	115.4	7.99	115.4	0.01	0.0	0.01
263A	8.24	121.7	8.21	121.5	0.03	0.2	0.04
264R	7.90	116.9	7.96	117.1	-0.06	-0.2	0.07
265M	7.94	118.6					
266A	8.29	121.7	8.27	122.1	0.02	-0.4	0.06
267D	8.48	123.9	8.50	124.2	-0.02	-0.4	0.06
268K	7.04	115.6	7.00	115.5	0.04	0.0	0.04
269L	7.36	117.8	7.36	117.6	0	0.2	0.03
270R	7.12	123.1	7.07	122.9	0.05	0.2	0.06

Table S27. *Mean and standard deviation of the chemical shift perturbations for NDM-1 in the presence of 10 equivalents of compound* ***5f****.*

Compound	Mean (ppm)	Standard deviation (ppm)
**5f**	0.05	0.05

13. X-ray diffraction of VIM-2 inhibitor complexes

Protein expression and purification

For expression and purification of VIM-2 the pDEST14_VIM-2 plasmid described in Christopeit et *al*. 2016[24] was used. It consists of a His_6-tag and a Tobacco etch virus (TEV) protease cleavage site at the N-terminus of the VIM-2 gene sequence coding for residues Val25-Glu300. The plasmid was transformed into chemically competent *Escherichia coli* BL21 (DE3). Bacteria were grown at 37 °C in LB medium supplemented with 100 µg/ml ampicillin. Protein expression was induced at an OD600 of 0.7 with 0.4 M isopropyl β-D-1-thiogalactopyranoside (IPTG) and cells were harvested after overnight expression at 20 °C by centrifugation at 5.500 x g for 10 min. Cells were resuspended in IMAC buffer (50 mM Tris/HCl, pH 7.2, 150 mM NaCl, 100 µM $ZnCl_2$) and sonicated for lysis. The cleared lysate was applied onto a 5 ml HisTrap FF column (Cytiva). The column was washed with washing buffer (50 mM Tris/HCl, pH 7.2, 150 mM NaCl, 100 µM $ZnCl_2$, 25 mM imidazole) and VIM-2 was eluted via a linear imidazole gradient (50 mM Tris/HCl, pH 7.2, 150 mM NaCl, 100 µM $ZnCl_2$, 25 - 500 mM imidazole). The His_6-tag was proteolytically removed by TEV digestion (added in a 1:15 molar ratio) and followed by overnight dialysis against IMAC buffer using a 6-8 kDa cutoff membrane. Subsequently, the cleaved His_6-tag, uncleaved protein and the His-tagged TEV protease were removed by a reverse Nickel-affinity chromatography. The VIM-2 containing flow-through was collected and the buffer was exchanged to 50 mM Tris/HCl, pH 7.2, 100 µM $ZnCl_2$ using a 10 kDa cutoff ultra centrifugal filter (Amicon, Merck KGaA) preparing the sample for anion exchange chromatography. Anion exchange chromatography was performed using HiTrap Q FF 5 mL column (Cytiva) with buffer A2 (50 mM Tris/HCl, pH 7.2, 100 µM $ZnCl_2$) and the protein was eluted using a linear gradient of 0-100% buffer B2 (50 mM Tris/HCl, pH 7.2, 1 M NaCl, 100 µM $ZnCl_2$). The purified protein was concentrated to 10.3 mg/mL using a 10 kDa cutoff ultra centrifugal filter (Amicon, Merck KGaA) concentrator. Purified protein was flash-frozen in liquid nitrogen and stored at -80°C until further use.

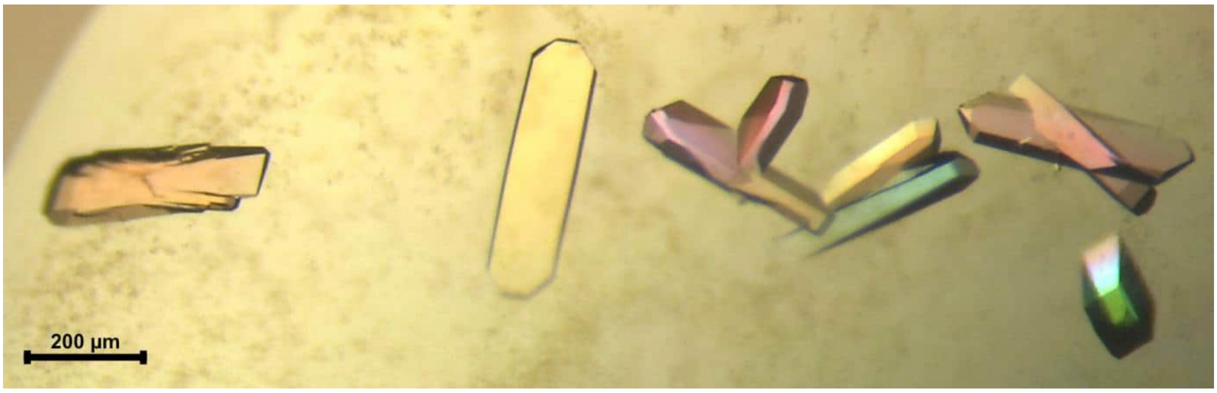

Figure S50. *Microscope image of VIM-2, co-crystallized with compound **5c** after 14 days. The scalebar is 200 µm.*

X-ray data collection and processing

X-ray diffraction data was collected at beamline P14 (EMBL-Hamburg, Germany) at 100 K on an Eiger2 CdTe 16 M detector (Dectris, Switzerland). Diffraction data were processed using XDS[25-27] and AutoPROC, using StarAniso[28,29] for the **5j** and **5g** structures, respectively. Phasing was performed by molecular replacement using *phaser*[30] with an apo VIM-2 structure in the same spacegroup as a search model (PDB ID: 5YD7). Geometry restraints for the inhibitors were generated via *phenix.elbow*, structures were iteratively refined in *phenix.refine* and manual model correction in *coot*[31,32]. POLDER-OMIT maps were generated in *phenix.polder*. Molecular images were generated in PyMol (Schrödinger LLC), 2D-representations of the protein ligand interactions were generated in LigPlot+[33]. Dose calculations were conducted using RADDOSE3D[34]. Refined coordinates and structure factors of the VIM-2 inhibitor complexes were deposited in the RCSB structure data-base under the coordinates 9F0Q (**5d**); 9F0P (**5c**); 9F0S (**5j**); 9F0R (**5g**). Data-collection and refinement statistics are shown in **Table S28**.

Table S28. *Data-collection and refinement statistics. Values in parenthesis indicate parameters in the highest resolution shell.*

Ligand	**5d**	**5c**	**5j**	**5g**
PDB ID	9F0Q	9F0P	9F0S	9F0R
Data collection				
Beam size (FWHM, µm)	50x201	50x52	60x248	58x358
Flux (ph/s)	7.94E+11	2.04E+11	2.33E+11	2.61E+11
Energy (keV)	15.5	15.5	15.5	15.5
Exposure time (ms)	8			
Diffraction weighted dose (MGy)	0.280603	0.108507	0.073586	0.082638
Number of images	2400			
SpaceGroup	C2			
Unit cell constants	101.80, 79.02, 67.82	102.15, 79.02, 67.89	101.82, 79.23, 67.78	101.26, 79.15, 67.70
Unit cell angles	90.0, 130.75, 90.0	90.0, 130.73, 90.0	90.0, 130.44, 90.0	90.0, 130.37, 90.0
with / without StarAniso	w/o	w/o	with	w/o
Resolution	55.19 - 1.92 (1.95 - 1.92)	55.29 - 1.34 (1.36 - 1.34)	55.40 - 1.15 (1.44 - 1.4)	55.24 - 1.21 (1.33 - 1.28)
R_{merge}	0.14 (0.98)	0.07 (0.61)	0.065 (0.507)	0.061 (0.605)
R_{meas}	0.154 (1.066)	0.07 (0.67)	0.07 (0.546)	0.065 (0.662)
R_{pim}	0.058 (0.397)	0.027 (0.280)	0.026 (0.201)	0.034 (0.265)
Total number of observations	216573 (10940)	623492 (22057)	603069 (30716)	777721 (63523)
Unique number of observations	30937 (1551)	90818 (4037)	85514 (4276)	113751 (10251)
Mean I/sd(I)	9.8 (2.2)	13.3 (2.2)	12.8 (3.48)	15.0 (3.3)
Completeness (ellipsoidal)	99.3 (99.4)	98.3 (87.0)	78.3 (67.7)	92.5 (88.8)
Multiplicity	7.0 (7.1)	6.9 (5.5)	7.1 (7.18)	6.8 (6.2)
CC(1/2)	0.997 (0.802)	0.999 (0.865)	0.999 (0.897)	0.99 (0.84)
Refinement				
Resolution range	55.19 - 1.92	39.51 - 1.34	55.34 - 1.40	31.39 - 1.30
Number of reflections	30907	90789	77533	98583
R_{work}	0.1712	0.1727	0.173	0.1366
R_{free}	0.2134	0.1884	0.1846	0.1693
Test set size (%)	4.97	4.76	4.96	5.01
Wilson B (Å^2)	21.69	15.12	13.24	12.59
Deviations from ideal values				
Bonds	0.007	0.006	0.007	0.088
Angles	0.889	0.901	1.109	2.75
Ramachandran Plot				
Outliers (%)	0.44	0.44	0.44	0.22

Allowed (%)	1.97	1.53	1.31	1.53
Favoured (%)	97.6	98.03	98.25	98.25
mean B-factors				
Overall	26.12	21.96	19.04	19.25
Protein	25.09	20.39	17.21	16.98
Water	33.68	32.13	30.03	23.32
Other	27.38	24.82	23.33	31.33

VIM-2 inhibitor complex crystal structures

Crystals of VIM-2 cocrystallized with compounds **5d**, **5c**, **5j** and **5g** belong to spacegroup C2 with 2 molecules in the asymmetric unit, packed in a face to backside orientation. The two subunits can be superimposed with a root mean square deviation (r.m.s.d) of ~0.15 - ~0.2 Å, indicating only minor intramolecular structural differences within the crystals (**Table S29**). To isolate structural differences induced by ligand binding, the VIM-2 inhibitor complexes were compared to an apo VIM-2 structure in the same space-group (PDB ID: 5YD7). Globally the inhibitor structures display an overall r.m.s.d. of ~0.2 - ~0.3 Å compared to the apo structure and can therefore be considered to be highly similar. However, some backbone variations can be determined in the inhibitor binding pocket. With respect to the apo structure, a slight opening of the protein backbone (up to ~2.5 Å) can be observed to accommodate the inhibitors. Compounds **5d** and **5c** generally induce larger shifts than **5j** and **5g** (**Table S29**).

Table S29. *VIM-2 inhibitor complex differences to apo VIM-2.*

			C_{alpha} shift compared to apo VIM-2 (Å)							
	r.m.s.d. (Å)		Chain A				Chain B			
VIM-2 complex	intramolecular	apo (5YD7)	Phe 62	Asp 63	Gly 209	Asn 210	Phe 62	Asp 63	Gly 209	Asn 210
**5d**	0.20	0.33	1.5	1.5	2.2	1.3	0.8	0.6	2.3	1.6
**5c**	0.16	0.27	2.0	1.6	2.6	1.9	0.7	0.8	2.6	1.9
**5j**	0.15	0.27	0.6	0.3	0.4	0.5	0.3	0.3	0.5	0.8
**5g**	0.17	0.23	1.0	0.6	0.3	0.7	0.2	0.3	0.4	1.2

Protein inhibitor interaction

Unsurprisingly, the four different inhibitors were found to bind in the active site of VIM-2 as evident from strong difference electron density after molecular replacement. Although not all residual difference density could be explained during refinement, the quality of the electron density maps permitted placement of the inhibitors, and in two cases (compounds **5d**, **5c**) unambiguous assignment of their absolute configuration, which was further supported by difference density omit maps (**Figure S51, Table S30**).

Table S30. *Absolute configuration of the inhibitors bound to VIM-2 with RSCC values in brackets.*

VIM-2 complex	enantiomer		
	subunit A	subunit B	interface
**5d**	S (0.91)	R (0.93)	S (0.87)
**5c**	S (0.91/0.93)	R (0.96)	S (0.91/0.93)
**5j**	R (0.93)	R (0.93)	n/a
**5g**	R (0.88)	R (0.96)	n/a

Commonly, the inhibitors interact with VIM-2 via their phosphonic acid group that bridges the two zinc ions. In addition, the inhibitors form interactions to His116 (except in compound **5d**, in subunit A) and to Asp118. In subunit B of compound **5d** and **5c**, respectively, the inhibitors also form a hydrogen bond to the amide in the side-chain of Asn210, which likely contributes to the slight shift in the backbone in comparison to the apo structure. In addition, to the inhibitors described above we were also able to solve a VIM-2 structure in complex with **5l** (*data not shown*). However, while this structure clearly showed an analogous binding mode via interaction of the phosphonic acid group with the zinc ions, the side-chain could unfortunately not be unambiguously assigned, underlining the dynamic binding mode of these inhibitors, and corroborating the results discussed below. Interestingly, in the structures of **5d** and **5c** the interface between subunit B and a symmetry related subunit A is occupied by an additional inhibitor molecule. This forms a hydrogen bond to the backbone amide of Asn210 and may thereby also contribute to the opening of the backbone in comparison to apo VIM-2. The phosphonic acid group of this additional inhibitor molecule faces the active site of the symmetry

related subunit A. Since this molecule is located at the interface between two symmetry related molecules, we assume that the crystal packing supports this binding mode and does not reflect a physiologically relevant interaction with VIM-2. A detailed overview of the different interactions can be found in **Figure S53**.

Stereodynamic inhibitors show versatile VIM-2 binding modes

Surprisingly, in the VIM-2 inhibitor structures of **5d** and **5c**, the (*R*)- and (*S*)-enantiomers, respectively, bind in either subunit A or subunit B (**Table S30**). We speculate that this 'crystallographic stereoselectivity' might be promoted by the binding of the additional inhibitor molecule at the crystal packing interface. The latter is corroborated by the absolute configuration of **5d** and **5c**, with the opposite enantiomer of the same inhibitor being bound rotated by ~180°. Steric hindrance of the interface-bound molecule prevents binding of the (*S)*-enantiomer in the active site of subunit B. In subunit A, however, inhibitor binding does not interfere with an additional interface bound ligand and the (*S*)-enantiomer can be bound.

By contrast, in the VIM-2 inhibitor structures of **5j** and **5g** only the (*R*)-enantiomer could be faithfully modelled into the active site. We note that clearly the active sites of these inhibitor complexes display further substantial difference density, which likely corresponds to additional bound inhibitor molecules. However, due to the dynamic nature of the binding mode the quality of the electron density did not permit unambiguous modelling or assignment of the absolute configuration, of these additional electron density patches. While we anticipate a binding pattern similar to **5d** and **5c**, where the active sites show binding competence to both enantiomers, the electron density in **5j** and **5g** was conservatively better explained by only placing the (*R*)-enantiomer in both subunits.

In summary, VIM-2 displays binding competence to these highly stereodynamic inhibitors with a conserved primary interaction of the phosphonic acid with the zinc ions in the active site. Our data support highly versatile binding modes of the inhibitors as VIM-2 can not only bind both enantiomers but is tolerant to alternate rotations in the active site. Moreover, in spite of highly variable substituents, all inhibitors were tightly bound in the active site. We anticipate that these properties will prevent sudden resistance formation as alternate binding modes could compensate for this or probably lead to a loss of function within VIM-2.

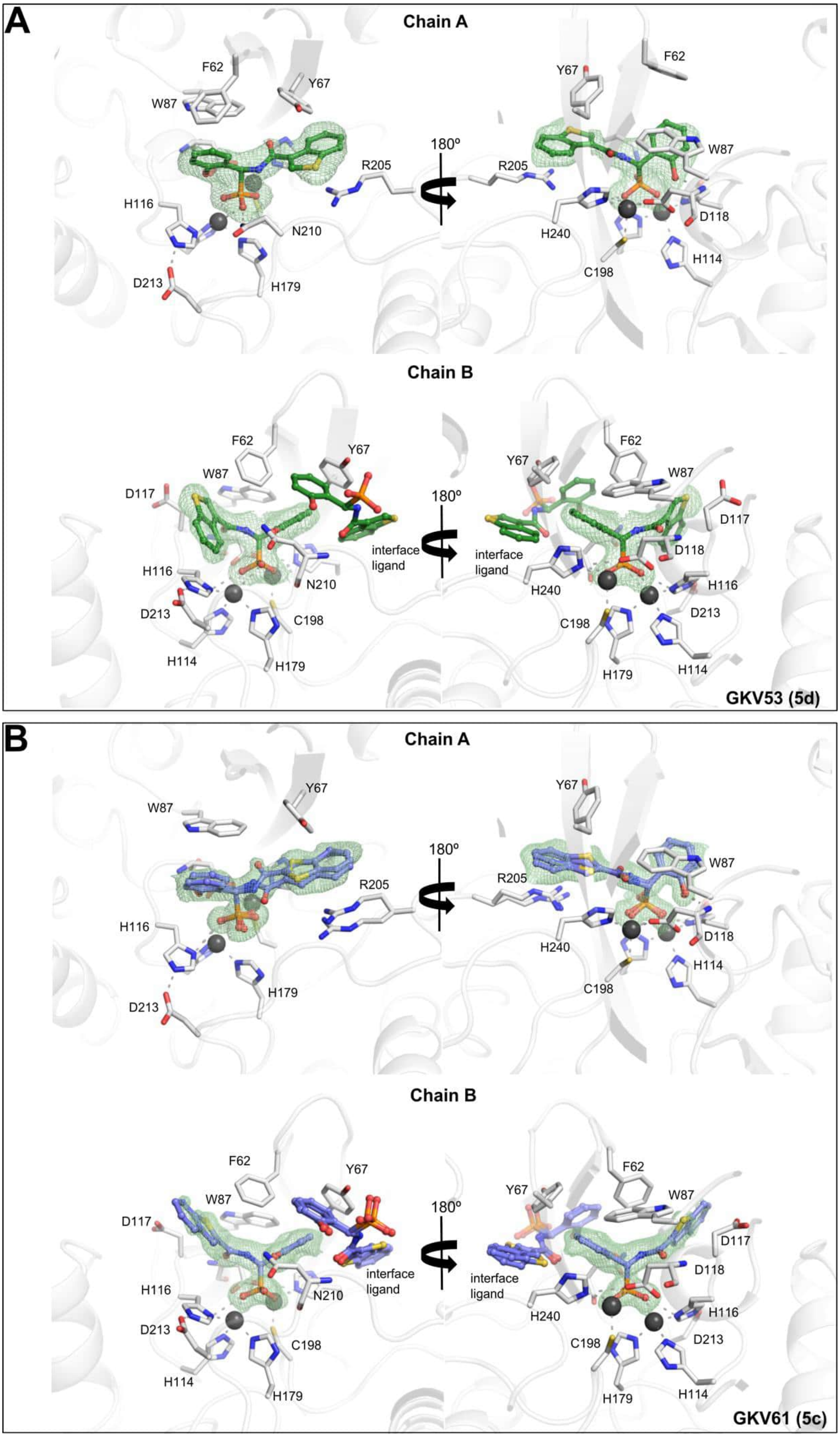

A
Chain A
F62
Y67
W87
R205
180º
H116
N210
D213
H179
D118
H240
C198
H114
Chain B
D117
interface ligand
N210
C198
H114
H179
GKV53 (5d)
B
Chain A
Chain B
GKV61 (5c)

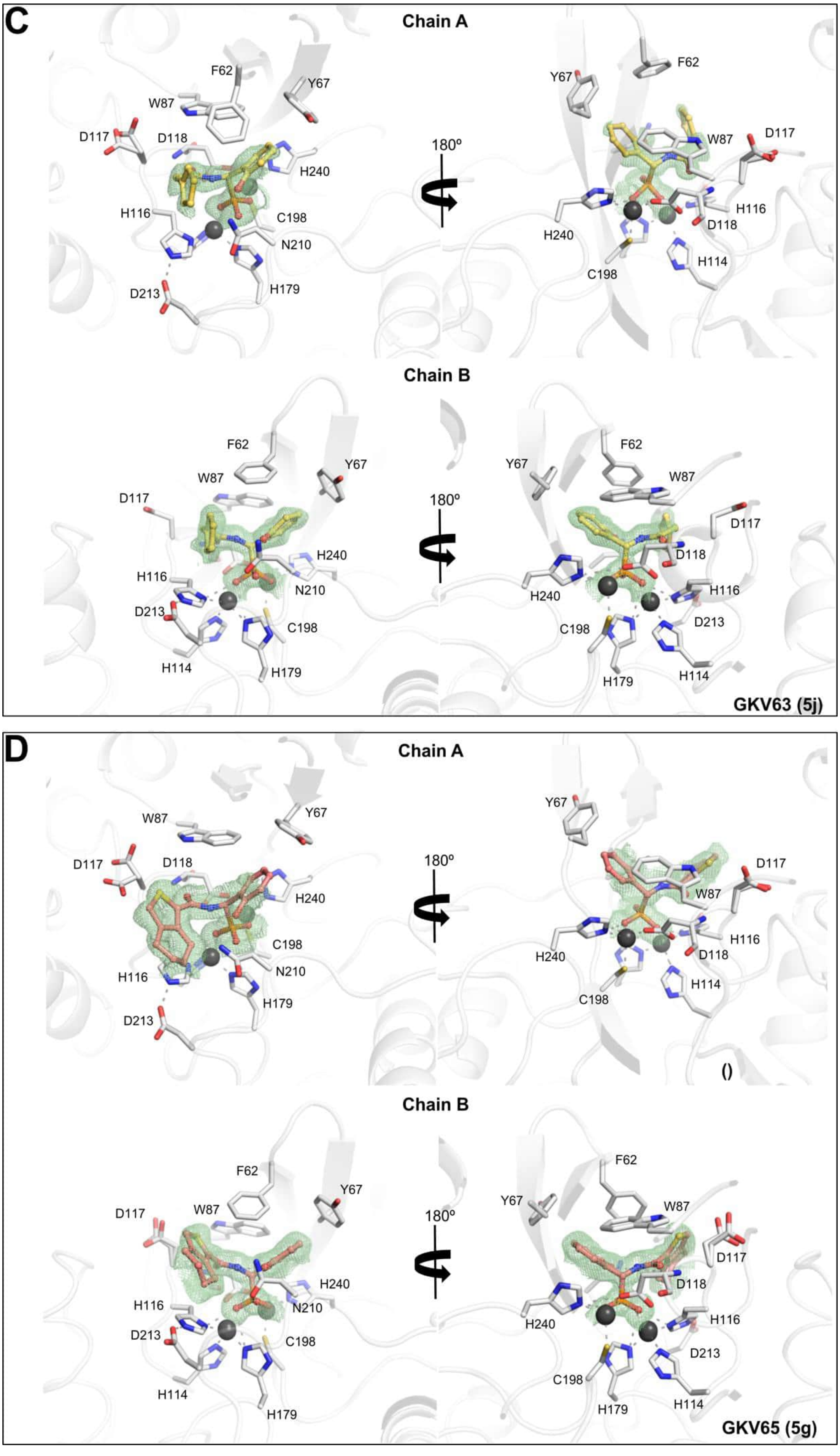
C
Chain A
F62
Y67
W87
D117
D118
H240
H116
C198
N210
D213
H179
180º
H114
Chain B
GKV63 (5j)
D
GKV65 (5g)

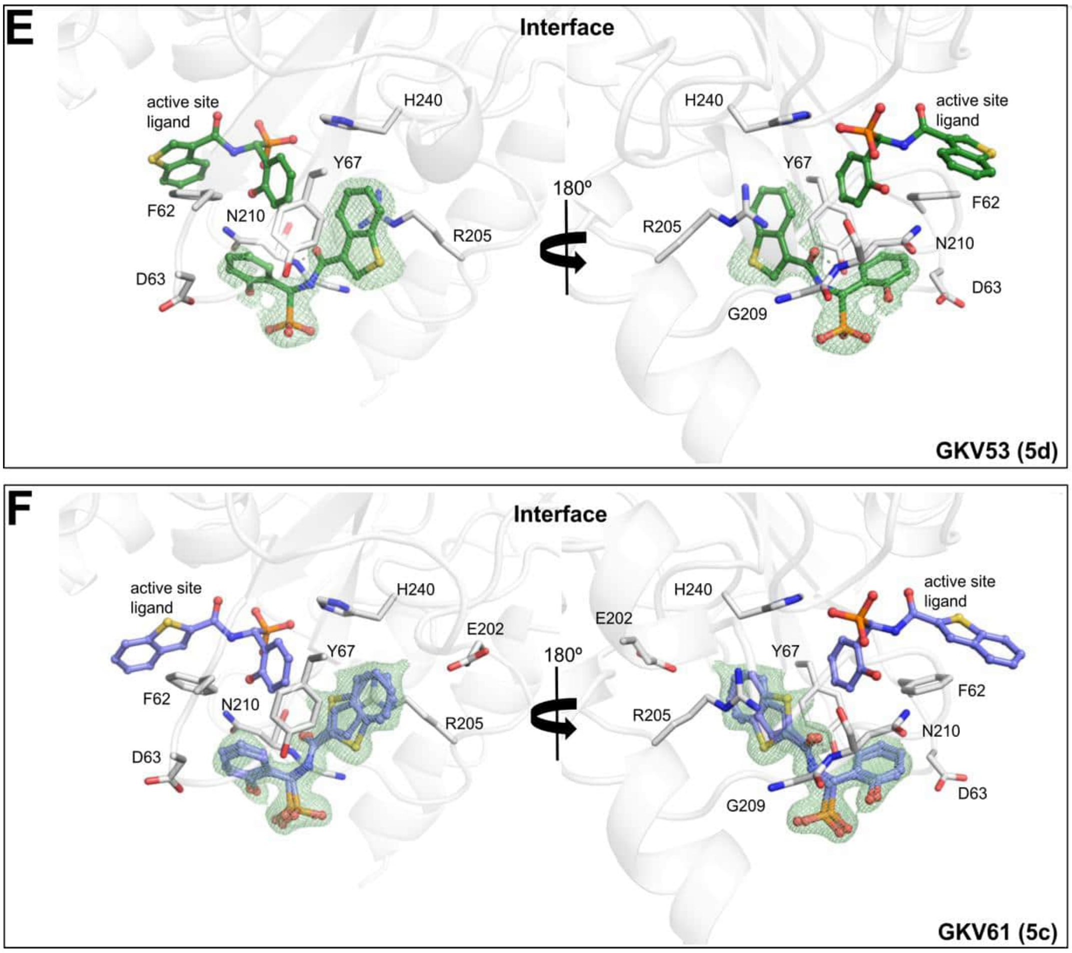

Figure S51. VIM-2 inhibitor complexes. *Difference electron density polder-OMIT maps support the orientation of the inhibitor molecules within the active site: A,E) compound **5d**; B, F) compound **5c**; C) compound **5j**; D) compound **5g** – 180° rotations of each panel demonstrate the goodness of fit of the inhibitors to the electron density. VIM-2 is shown as a grey cartoon, relevant side-chains in stick representation. Inhibitors are shown as ball-and-stick model in different colours. The polder-OMIT difference electron density maps are shown as a green mesh at an r.m.s.d.-level of 3*

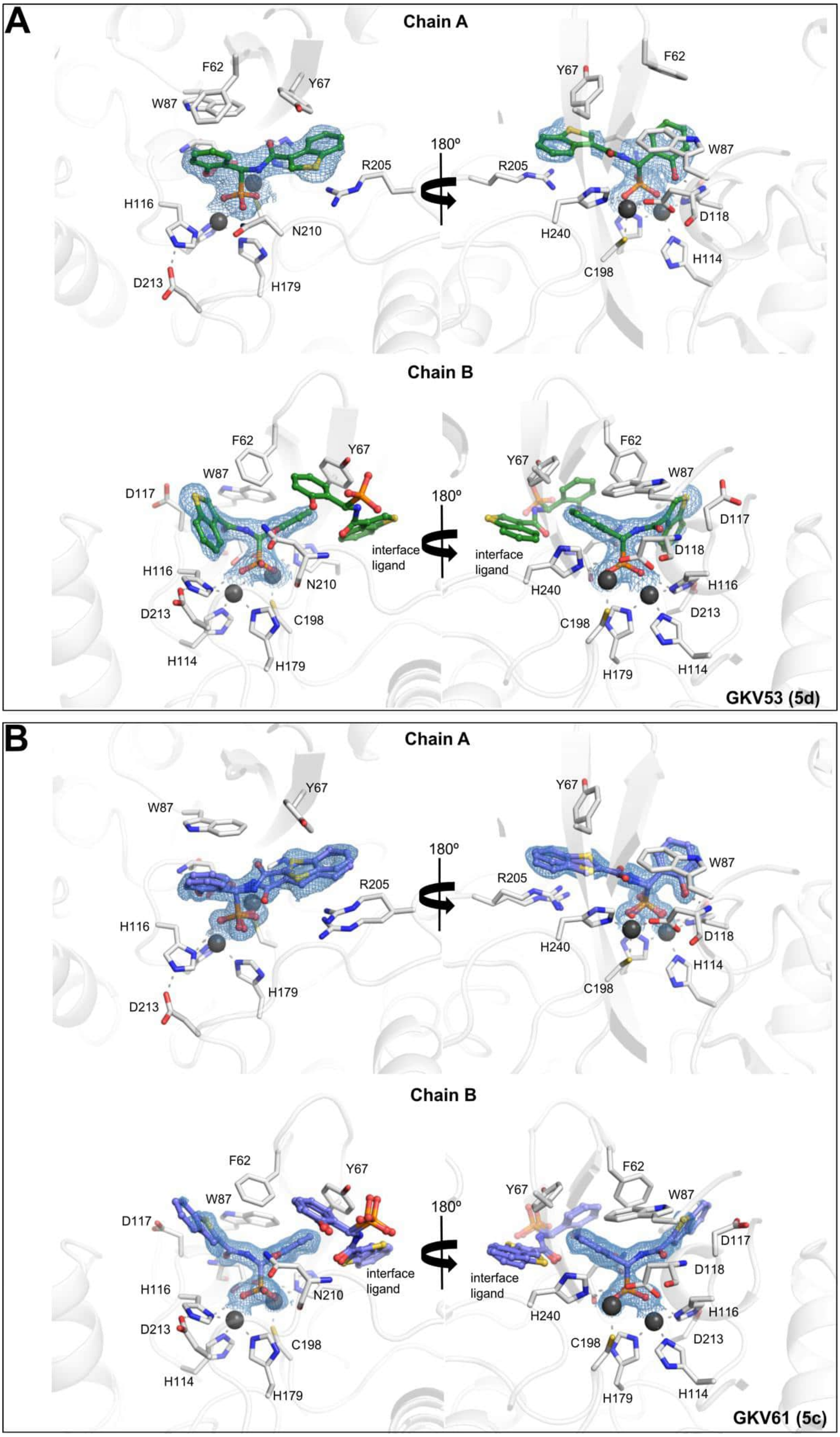
A
Chain A
F62
Y67
W87
180º
R205
H116
N210
D213
H179
W87
D118
H240
C198
H114
Chain B
F62
Y67
W87
D117
interface ligand
H116
N210
D213
C198
H114
H179
H240
D118
GKV53 (5d)
B
Chain A
Y67
W87
R205
H116
H179
D213
D118
H240
H114
C198
Chain B
F62
Y67
W87
D117
interface ligand
H116
N210
D213
C198
H114
H179
D118
H240
GKV61 (5c)

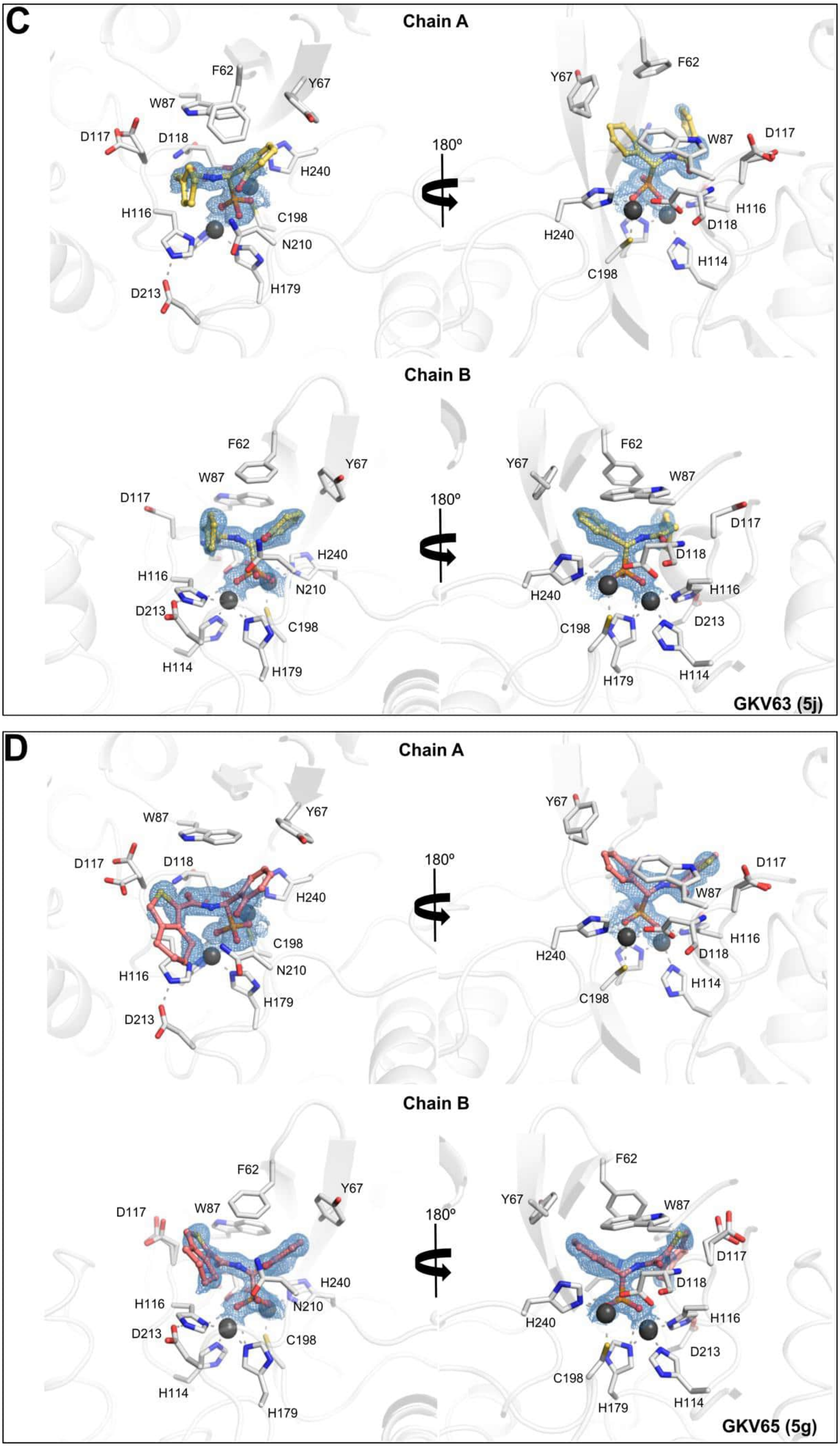

C
Chain A
F62
Y67
W87
D117
D118
H240
H116
C198
N210
D213
H179
180º
Y67
F62
W87
D117
H116
D118
H240
C198
H114
Chain B
F62
Y67
W87
D117
H240
H116
N210
D213
C198
H114
H179
180º
Y67
F62
W87
D117
D118
H240
H116
D213
C198
H179
H114
GKV63 (5j)
D
Chain A
W87
Y67
D117
D118
H240
C198
H116
N210
H179
D213
180º
Y67
D117
W87
H240
H116
D118
H114
C198
Chain B
F62
Y67
D117
W87
H240
H116
N210
D213
C198
H114
H179
180º
F62
Y67
W87
D117
D118
H240
H116
D213
C198
H179
H114
GKV65 (5g)

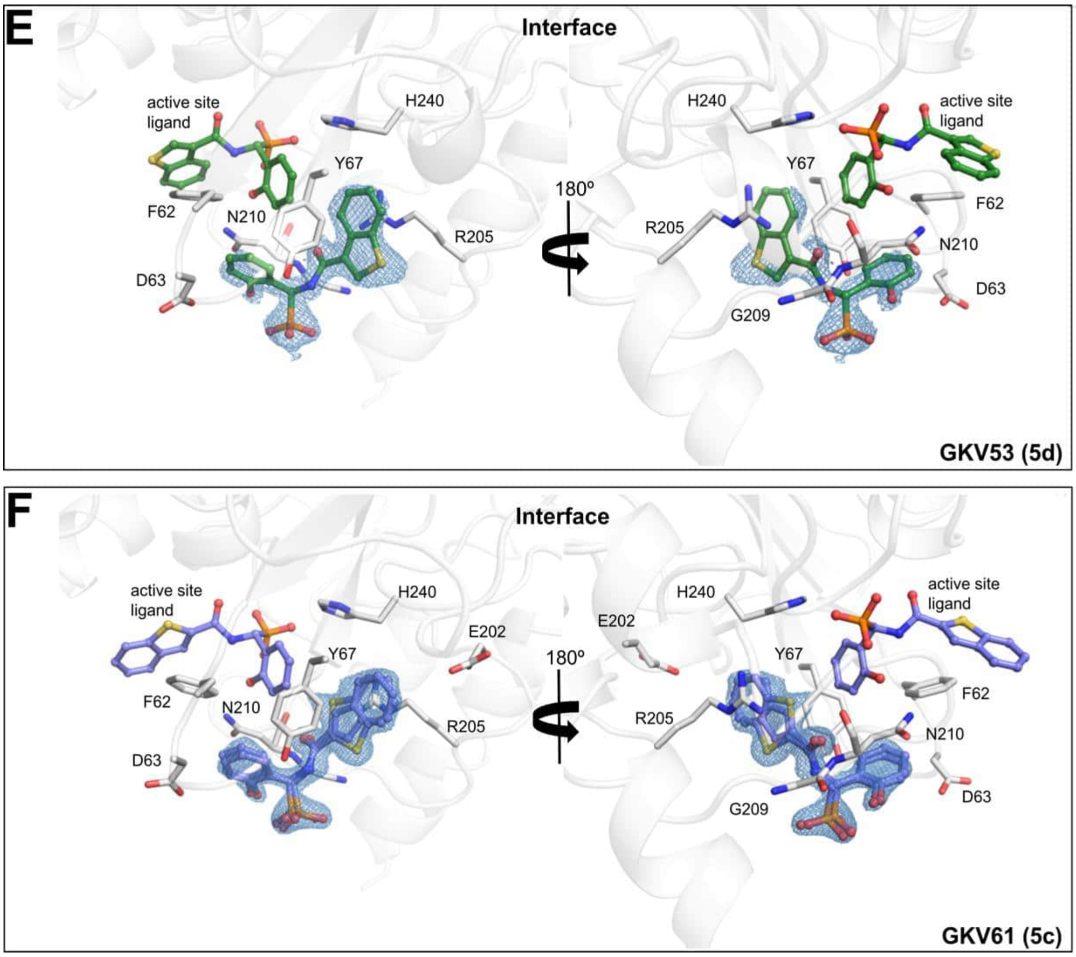

Figure S52. VIM-2 inhibitor complexes. *As above but as 2mFo-Fc map shown as a blue mesh at an r.m.s.d.-level of 1.*

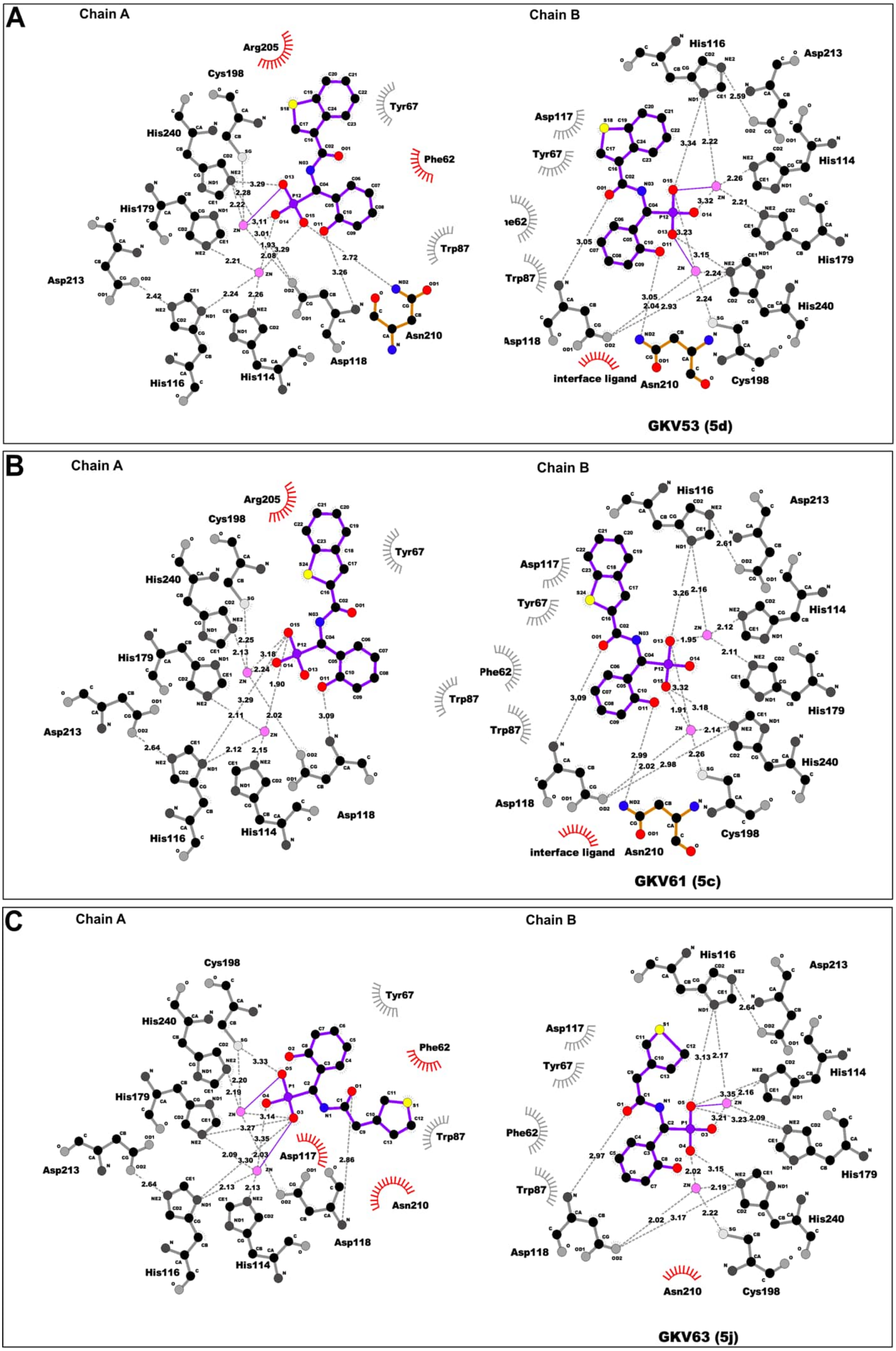
A
Chain A
Chain B
Arg205
Cys198
Tyr67
Phe62
Trp87
His240
His179
Asp213
Asn210
Asp118
His116
His114
His116
Asp213
Asp117
Tyr67
Trp87
His114
His179
His240
Asp118
interface ligand
Asn210
Cys198
GKV53 (5d)
B
Chain A
Chain B
Arg205
Cys198
Tyr67
His240
His179
Asp213
Trp87
Asp118
His116
His114
His116
Asp213
Asp117
Tyr67
Phe62
Trp87
His114
His179
His240
Asp118
interface ligand
Asn210
Cys198
GKV61 (5c)
C
Chain A
Chain B
Cys198
Tyr67
His240
Phe62
His179
Trp87
Asp117
Asp213
Asn210
Asp118
His116
His114
His116
Asp213
Asp117
Tyr67
Phe62
Trp87
His114
His179
His240
Asp118
Asn210
Cys198
GKV63 (5j)

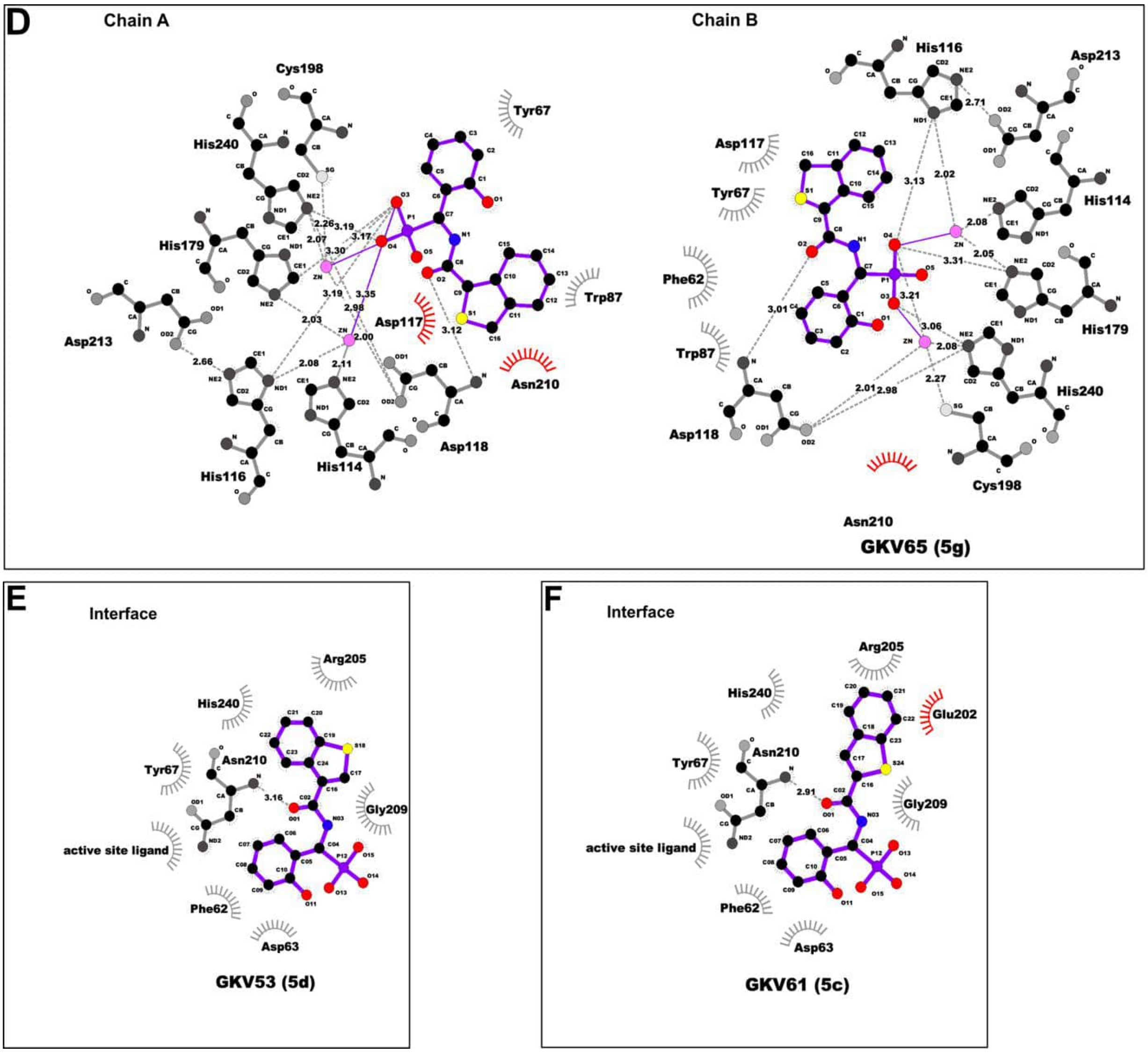

Figure S53. LigPlot diagram of the inhibitor VIM-2 interactions. *A-C) Interactions with compound* ***5d****, as indicated; D-F) interactions with* ***5c****. G/H)* ***5j*** *I/J)* ***5g****. Protein residues are shown in grey shades, the inhibitor molecules are depicted in purple with atom-specific color coding (carbon – black, oxygen – red, nitrogen – blue, sulfur – yellow), the zinc ions are shown as pink spheres.*

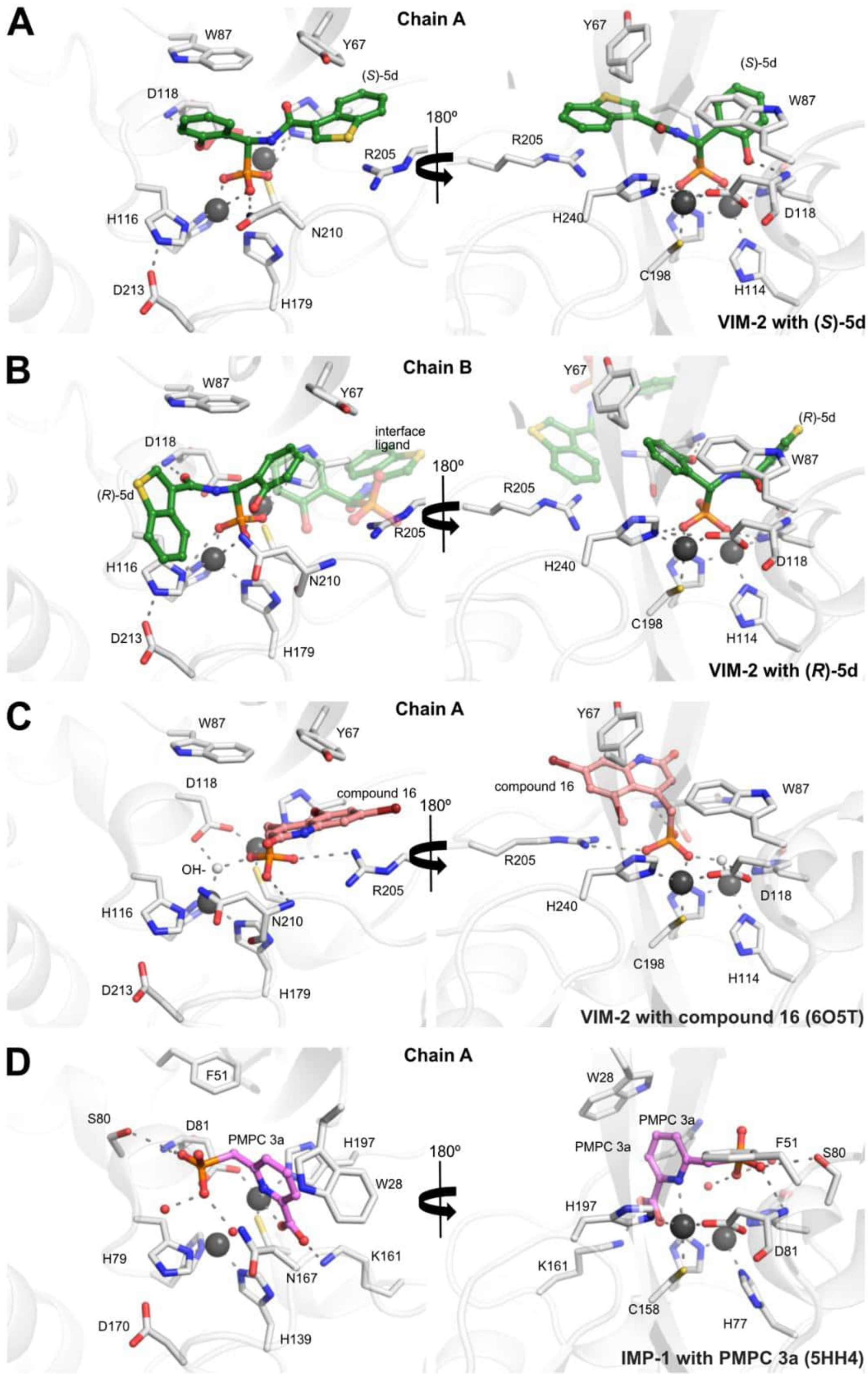

Figure S54. *Comparison of our dynamically chiral phosphonic acid inhibitor binding with previous phosphonate based inhibitors in class B1 metallo-beta-lactamases. Binding modes of A) compound 5d (S)-enantiomer in subunit A, B) 5d (R)-enantiomer in subunit B and C) compound 16 ([(5,7-dibromo-2-oxo-1,2-dihydroquinolin-4-yl)methyl]phosphonic acid; PDB 6O5T) in VIM-2 and D) PMPC 3a in IMP-1 (6-(phosphonomethyl)pyridine-2-carboxylic acid; PDB 5HH4).*

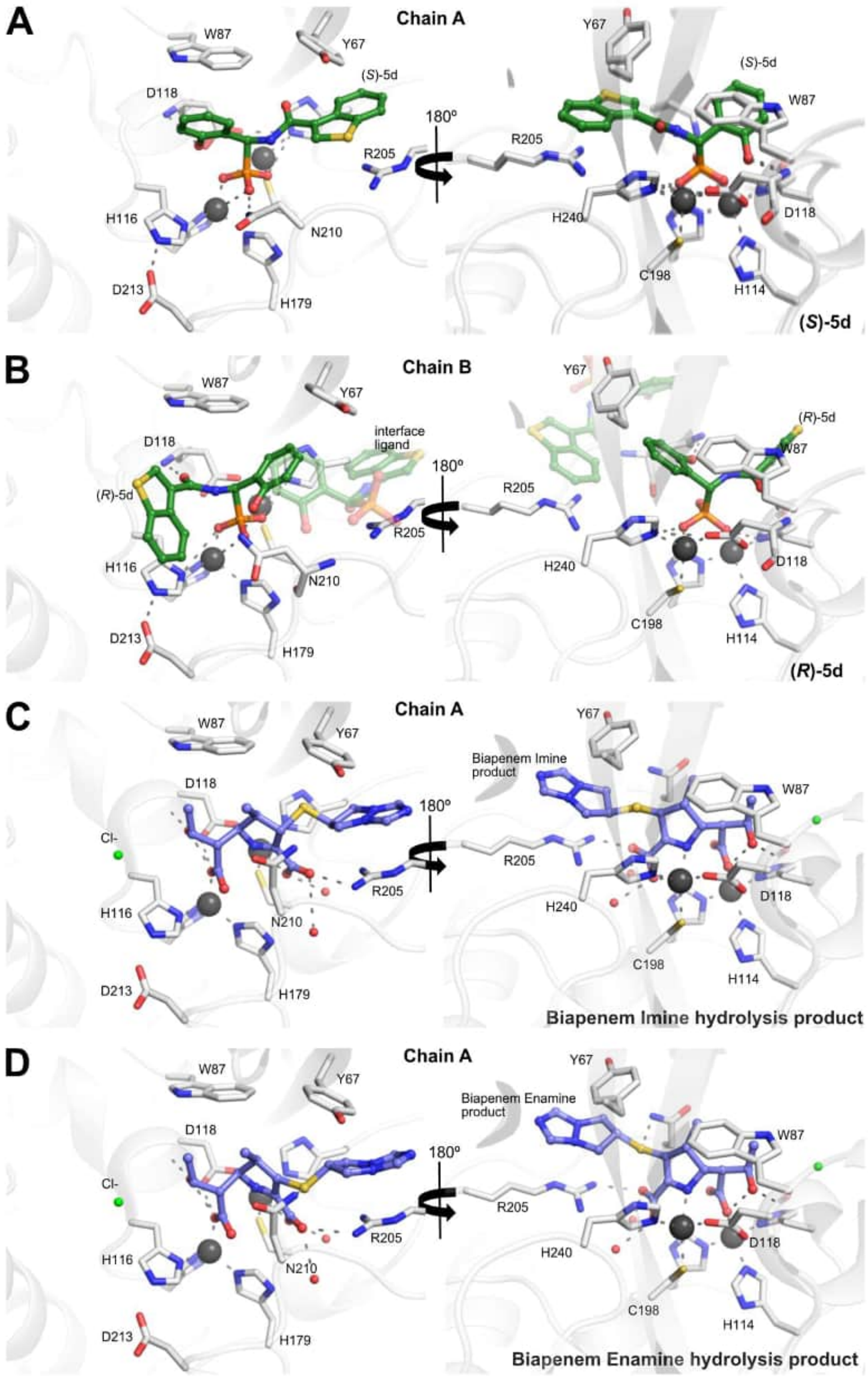

Figure S55. *Comparison of phosphonic acid inhibitor binding with hydrolyzed antibiotic product in VIM–2. Binding modes of a) compound* ***5d*** *(S)-enantiomer in subunit A, b)* ***5d*** *(R)-enantiomer in subunit B, c) biapenem imine hydrolysis product (PDB 6Y6J) and d) biapenem enamine hydrolysis product (PDB 6Y6J).*

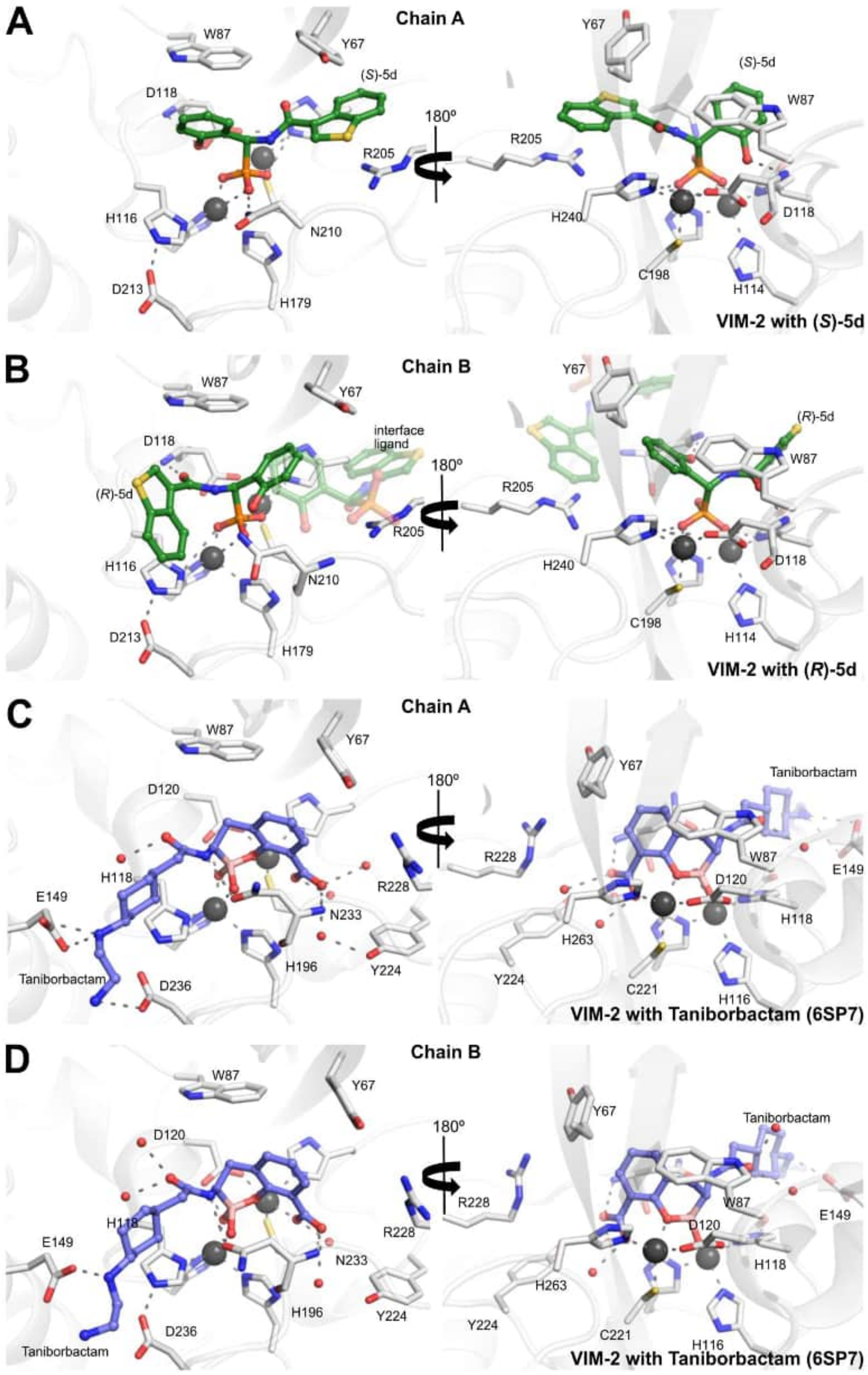

Figure S56. *Binding modes of compound* ***5d*** *(S)-enantiomer in subunit A and* ***5d*** *(R)-enantiomer in subunit B compared to taniborbactam binding (PDB:6SP7; for naming scheme, see Table S1).*

Tetrahedral orientation of the phosphonate moiety

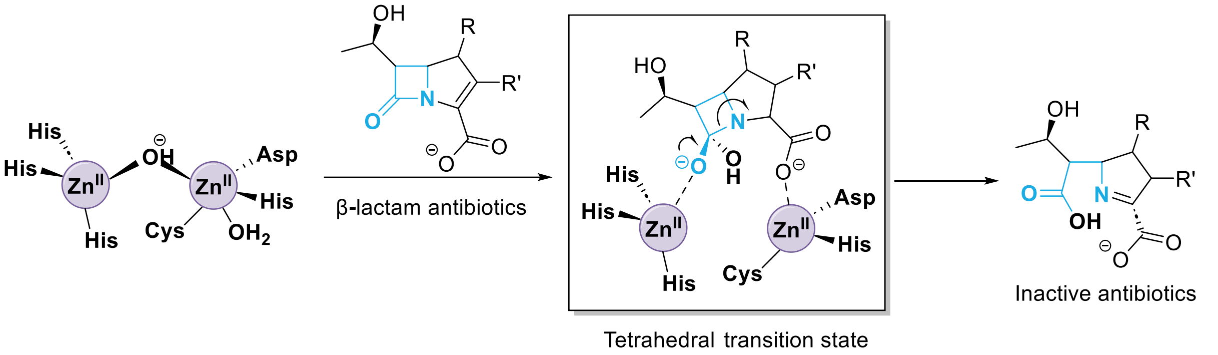

Figure S57. *The proposed general mechanism for β-lactam antibiotic hydrolysis by metallo-β-lactamases, shown on the example of carbapenems. The hydrolysis is proposed to proceed via a tetrahedral transition state.*[35]

The bond angles observed in the crystal structures of compounds **5c**, **5d**, **5g**, and **5j** in complex with VIM-2 indicate an sp^3 hybridized phosphorous that possesses tetrahedral geometry when binding to the zinc ions in the VIM-2 active site. β-Lactam hydrolysis is proposed to proceed via a tetrahedral transition state, involving a tetrahedral sp^3 carbon atom next to the nitrogen of the β-lactam ring (**Figure S57**). The hydrolysed β-lactam biapenem by VIM-2 (PDB ID: 6Y6J) is shown in **Figure S58**. Biapenem complexes the β-lactam nitrogen and the dihydropyrrol ring connected carboxylate oxygen coordinates Zn2, and the carboxylate that was originally part of the β-lactam ring coordinates Zn1 with both of its oxygens. The (*S*)-isomers of compounds **5c** and **5d** closely resemble the hydrolysed biapenem structure (**Figure S58**). The heterocycles of **5c-d** and of biapenem overlap as well as the phenolic hydroxy group of **5c-d** with the aliphatic hydroxy group of biapenem. The phosphonate group of **5c-d** coordinates both zinc ions whereas the carboxylate of biapenem coordinates to one of the zinc ions only, to which also the β-lactam nitrogen coordinates. The carboxylate formed by biapenem hydrolysis coordinates to the second zinc ion of the active site.

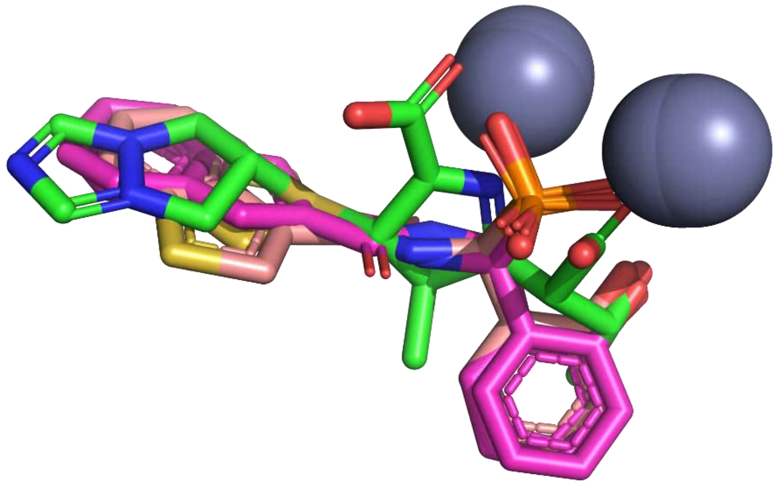

Figure S58. *Overlay of the structures of hydrolysed biapenem (green, PDB ID: 6Y6J), and of the (S)-isomers of inhibitor* **5c** *(magenta, PDB ID: 9F0P) and* **5d** *(salmon, PDB ID: 9F0Q) in complex with VIM-2.*

While the position of the phosphonate group in the (*R*)-isomers of **5** is not changed as compared to the (*S*)-isomers, the structures are flipped by 180°, so they don't overlap with the hydrolysed biapenem structure. However, the amide carboxylic oxygen of **5**-***(R)*** is positioned similar to the biapenem aliphatic hydroxy group (**Figure S59**).

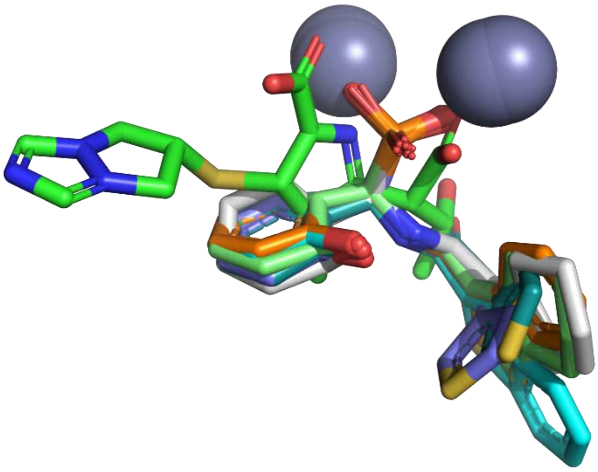

Figure S59. *Overlay of the structures of hydrolysed biapenem (green, PDB ID: 6Y6J), and of the (R)-isomers of inhibitor* ***5c*** *(cyan, PDB ID: 9F0P),* ***5d*** *(orange, PDB ID: 9F0Q),* ***5g*** *(gray and light green, PDB ID: 9F0S) and* ***5j*** *(purple and deepteal, PDB ID: 9F0R) in their complex with VIM-2.*

While the (*R*)-isomers don't closely resemble the orientation of the hydrolysed β-lactam biapenem in the binding site, they resemble the structure of VIM-2 bound taniborbactam. The tetrahedral boron atom of taniborbactam and the tetrahedral phosphorous atom of **5** are similarly positioned between the two zinc ions, while the amide bond of both and the moieties connected to them are pointing to the same direction and are located similarly. The carboxylate of the aromatic ring of taniborbactam is coordinated to Zn2, while the phenyl group of our inhibitors **5**-(*R*) is ~90° flipped as compared to the aromatic ring of taniborbactam (**Figure S60**). The binding mode of the (*S*)-isomers of our compounds don't show similarity to that of taniborbactam, other than the position of the phosphonate group which is conserved regardless of the stereochemistry (**Figure S61**). This indicates the adaptability of our inhibitors to the binding mode of both the hydrolysed β-lactam antibiotics and to the bicyclic boronate metallo-β-lactamase inhibitors.

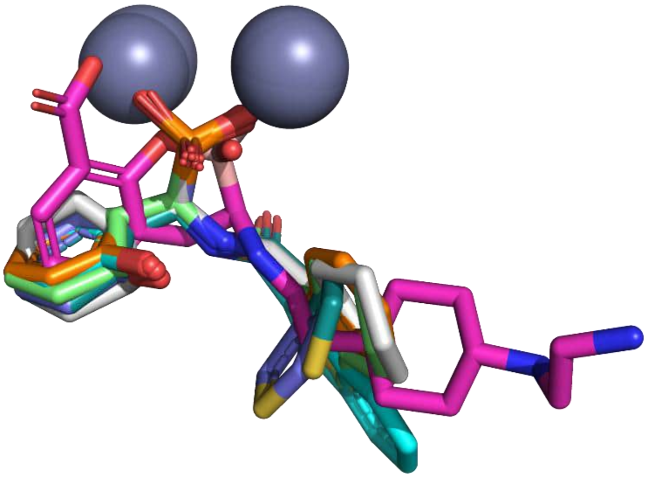

Figure S60. *Overlay of the structures of taniborbactam (magenta, PDB ID: 6SP7), and of the (R)-isomers of inhibitor* ***5c*** *(cyan, PDB ID: 9F0P),* ***5d*** *(orange, PDB ID: 9F0Q),* ***5g*** *(gray and light green, PDB ID: 9F0S) and* ***5j*** *(purple and deepteal, PDB ID: 9F0R) in their complex with VIM-2.*

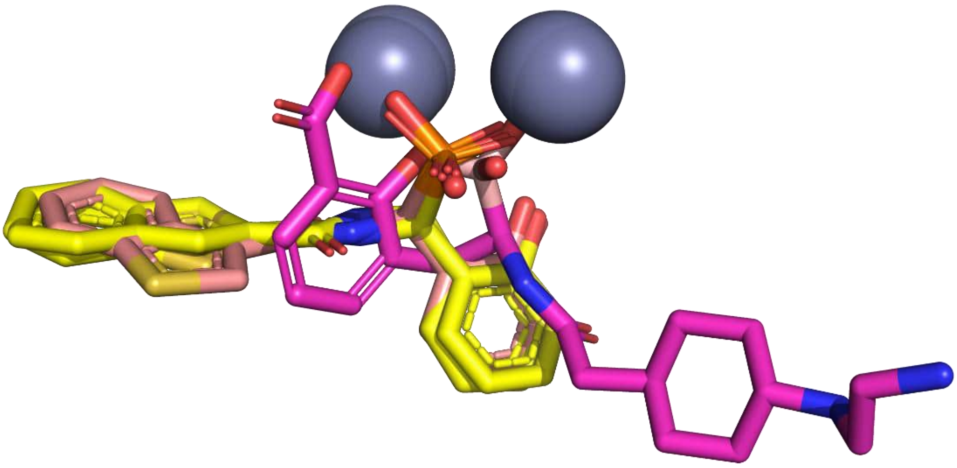

Figure 61. *Overlay of the structures of taniborbactam (magenta, PDB ID: 6SP7), and of the (S)-isomers of inhibitor* ***5c*** *(yellow, PDB ID: 9F0P) and* ***5d*** *(salmon, PDB ID: 9F0Q) in their complex with VIM-2.*

14. Computational simulations

Molecular docking

The structure of VIM-2 was derived from the crystal structure of VIM2-**5j**R, while the structures of GIM-1 (PDB ID: 2YNT[9]) and NDM-1 (PDB ID: 6D1A[10]) were obtained from the Protein Data Bank (PDB) for subsequent molecular docking studies. To prepare the protein structures at pH 7.35, the Protein Preparation Wizard[11] from the Schrödinger Release 2020 was employed. The docking site grid was generated using the Receptor Grid Generation application within the Glide module. For the ligands, three-dimensional structures of all compounds were generated using the LigPrep module (LigPrep, Schrödinger, LLC, New York, NY, 2020) at pH 7.35. Molecular docking was then carried out utilizing the Glide Standard Precision (SP) module[12,13] within the Schrödinger suite 2020 to investigate the binding patterns.

Computational docking of compounds **5c**, **5d**, **5g**, and **5j** to the binding site of VIM-2 confirmed to have similar binding poses, suggesting that zinc binding of the phosphonate group of the inhibitors plays a key role in inhibitor binding. Importantly, both stereoisomers were predicted to bind VIM-2, with comparable affinity (**Table S31**). The orientation of the two stereoisomers are opposite in the binding pocket while still keeping the strongest interaction between the phosphonic acid core and the Zn ions. The initial docked orientations for the (*R*)- and (*S*)-isomers of compound 5**g** were similar, but throughout the MD simulation the (*S*)-isomer turned around reaching a binding pose analogous to the (*S*)-isomer of compounds **5c** and **5d**, and **5j**, in good agreement with the X-ray data of the (*S*)-isomer of compound **5g** (**Figure S69**).

To assess the impact of amino acid mutations on compound binding, saturation mutagenesis was made to amino acids within 5 Å of the compound binding site. The mutagenesis of amino acids was performed using the wizard mutagenesis module in PyMOL (Schrödinger, LLC). The protein mutants were docked with compounds **5c**, **5d**, **5g**, and **5j** using the extra precision (XP) mode of Glide.[12,13]

Table S31. *Docking scores of the ligands and enzymes in kcal mol^{-1}.*

	**VIM-2**	**NDM-1**	**GIM-1**
**5a-R**	-8.472	-8.685	-7.562
**5a-S**	-8.240	-8.270	-8.178
**5b-R**	-8.486	-8.517	-7.652
**5b-S**	-8.433	-8.672	-8.224
**5c-R**	-8.332	-8.134	-7.317
**5c-S**	-8.125	-8.572	-8.614
**5d-R**	-8.413	-8.202	-7.397
**5d-S**	-7.845	-8.398	-8.553
**5e-R**	-8.311	-8.242	-7.403
**5e-S**	-8.147	-9.059	-8.540
**5f-R**	-8.426	-8.287	-7.272
**5f-S**	-8.092	-9.398	-8.597
**5g-R**	-8.419	-7.839	-7.058
**5g-S**	-6.633	-8.729	-8.497
**5h-R**	-8.407	-8.592	-7.464
**5h-S**	-8.339	-8.335	-8.343
**5i-R**	-8.898	-8.445	-7.240
**5i-S**	-8.556	-8.828	-8.313
**5j-R**	-8.489	-8.249	-7.207
**5j-S**	-8.489	-8.803	-8.337
**5k-R**	-8.285	-7.928	-7.616
**5k-S**	-8.229	-9.853	-8.342
**5l-R**	-8.465	-7.983	-7.212
**5l-S**	-8.252	-10.062	-8.402
**5m-R**	-8.785	-8.066	-7.579
**5m-S**	-8.496	-9.612	-8.182

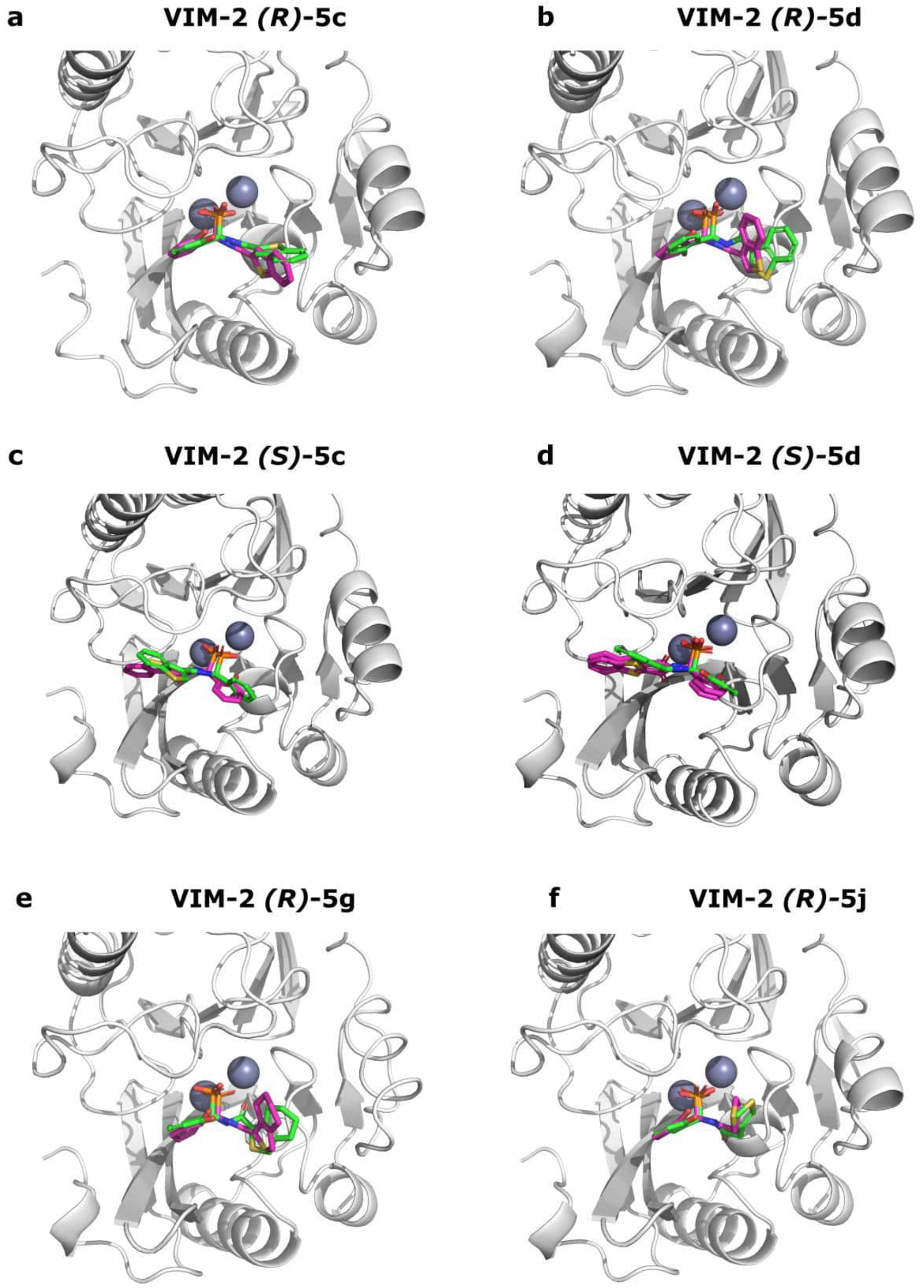

Figure S62. *Superimposition of the predicted binding modes from molecular docking and experimental binding modes for compounds* ***5c****,* ***5d****,* ***5g*** *and* ***5j*** *to VIM-2. The binding poses in crystal structures are shown in magenta and the predicted ones are shown as green.* ***a,*** *(R)-isomer of compound* ***5c****.* ***a,*** *(R)-isomer of compound* ***5d****.* ***c,*** *(S)-isomer of compound* ***5c****.* ***d,*** *(S)-isomer of compound* ***5d****.* ***e,*** *(R)-isomer of compound* ***5g****.* ***f,*** *(R)-isomer of compound* ***5j****.*

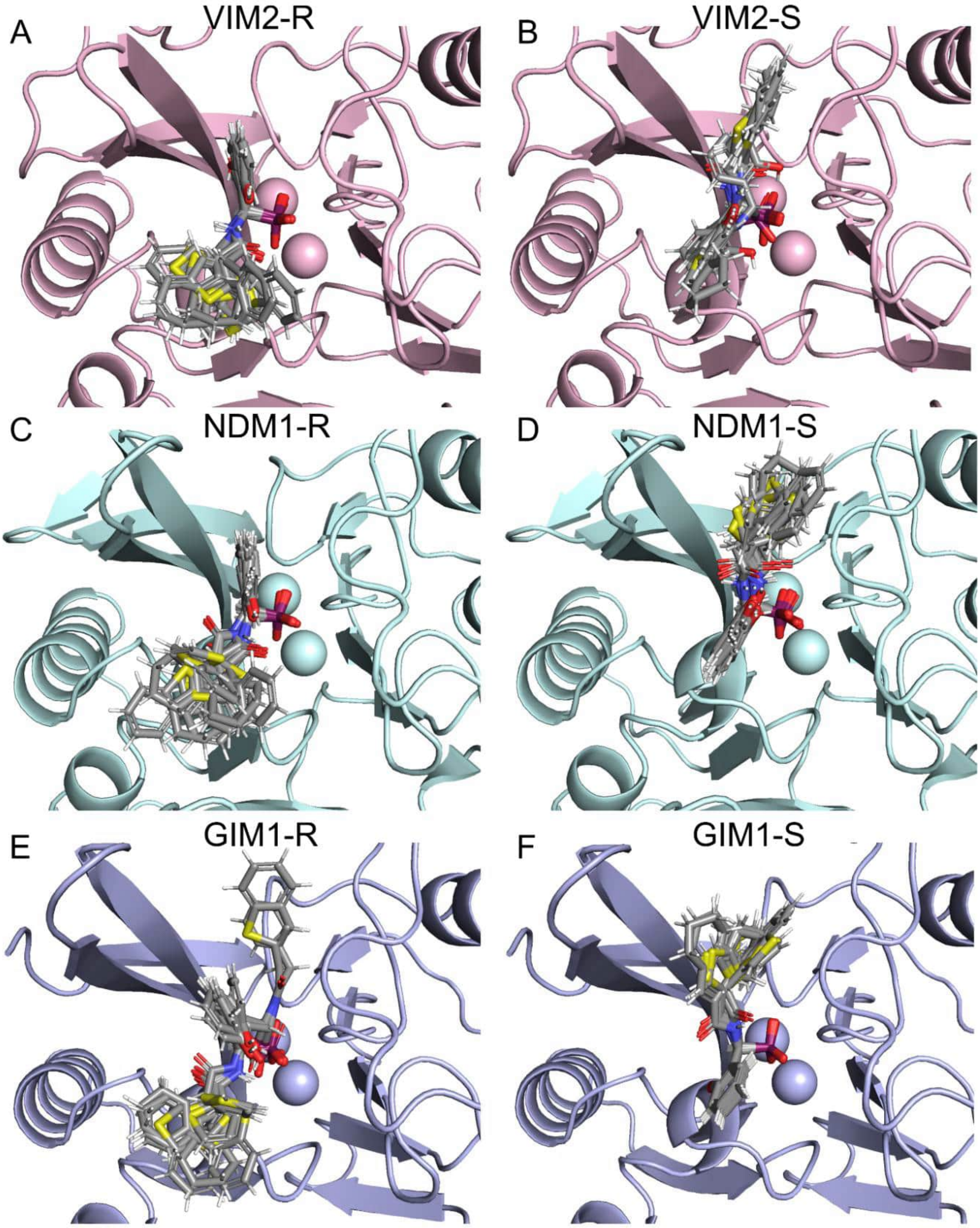

Figure S63. *The binding modes for (R)/(S)-isomers of 13 ligands (**5a-m**) against VIM2, NDM-1, GIM-1. The proteins, zinc ions and ligands are shown as cartoon, sphere and sticks.*

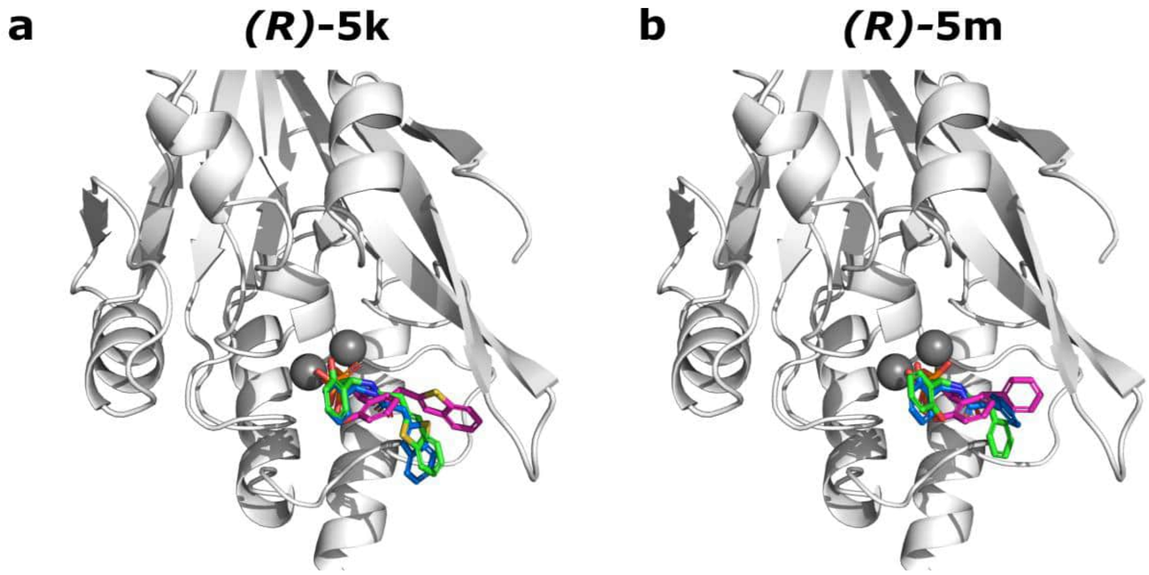

*Figure S64. The docked binding modes of **a,** (R)-**5k** against GIM-1 (magenta) superimposed with the binding modes of (S)-**5k** against VIM-2 (green) and against NDM-1 (blue) **b,** (R)-**5m** against GIM-1 (magenta) superimposed with the binding modes of (S)-**5m** against VIM-2 (green) and against NDM-1 (blue). GIM-1 (white) and its zinc ions (gray) and the ligands (coloured) are shown as cartoon, sphere and sticks.*

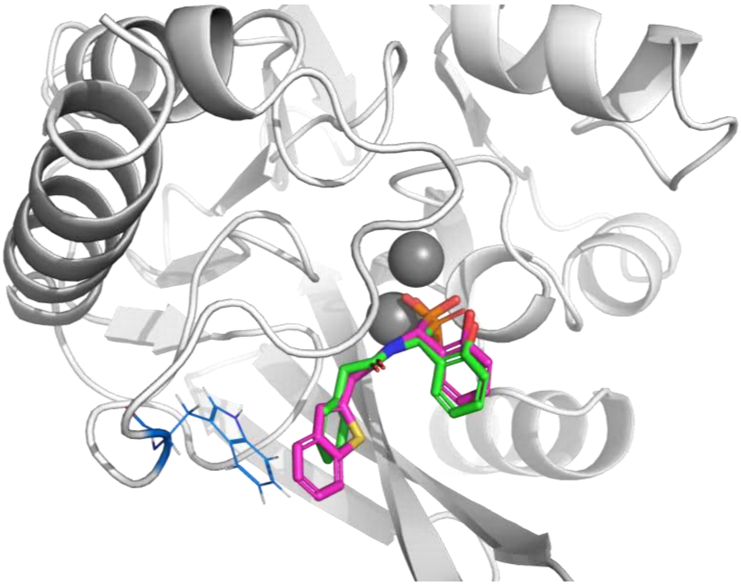

*Figure S65. The docked binding modes of (R)-**5k** (magenta) and (R)-**5m** (green) against GIM-1 with reside Trp228 highligted in blue. GIM-1 (white) and its zinc ions (gray) and the ligands (coloured) are shown as cartoon, sphere and sticks while residue Trp228 (blue) is shown as lines.*

Molecular dynamics simulation

Following the docking results, further molecular dynamics (MD) simulations were conducted for VIM2-**5g**, **5j**, **5c** and **5d** in three repetitions, each lasting 500 ns. The complex models were

solvated using a water model and parameterized employing the CHARMM36 all-atom additive force fields[14] for the protein. Additionally, sodium ions were introduced to neutralize charges in the system. The parameter files were obtained through Solution Builder module available within CHARMM-GUI[15,16]. During the energy minimization phase, a total of 8000 minimization steps were performed, consisting of 6000 steps of steepest descent minimization followed by 2000 steps of conjugate gradient minimization to eliminate unfavorable contacts and optimize the system's energy. Subsequently, a gradual temperature increase from 0 to 300 K was carried out over 200 ps with 1 fs/step. The equilibration phase was conducted in the NPT ensemble at 1.0 bar and 300 K, lasting for 50000 steps with 2 fs/step. The sander program, implemented in the AMBER18 software[17] package, was employed to execute the minimization, heating and NPT equilibration simulations. During these steps, position constraints (20 kcal/mol/$Å^2$) were applied to both the protein and ligand. The positional constraints were applied during the initial equilibration stages only to ensure system stability and to avoid any structural distortion. In the production phase of the molecular dynamics simulation all constraints were removed. As such, the production simulation results reflect the intrinsic interactions between the protein and ligand, free from external positional restraints.

In the production phase, the pmemd.cuda module in AMBER18 was used for 500 ns simulation. The simulations were conducted at 300 K and 1 bar for all complexes, and they were controlled using the Langevin thermostat[18] and the Nosé-Hoover Langevin barostat[19,20], respectively. To maintain the stability of the system, bonds involving hydrogen atoms were constrained using the SHAKE algorithm[21]. The cutoff distance applied for van der Waals interactions was 10 Å. All simulations were performed using particle-mesh Ewald (PME)[22] for long-range electrostatic interactions. Cpptraj module implemented in Amber18 was used for trajectory postprocessing and analyses. The Python library ProLif 1.1.0[23] was applied to generate interaction fingerprints for all conformations using default parameters.

As illustrated in **Figure S68**, besides the stable interaction between Zn and the phosphonate group, hydrophobic interactions between ligands and VIM-2 residues, such as Phe62, Trp87, Tyr67, dominate the binding process, which also elucidates the robust activity of compounds in this series. Additionally, π-stacking between His116 and the sulfur-containing moiety in **5c**, **5d**, **5g**, and **5j** is observed. Despite their low frequency, residues Asp118, Asp117, and Asn210 interact with these ligands through hydrogen bonds. In all cases, these four ligands show conserved binding patterns to VIM-2.

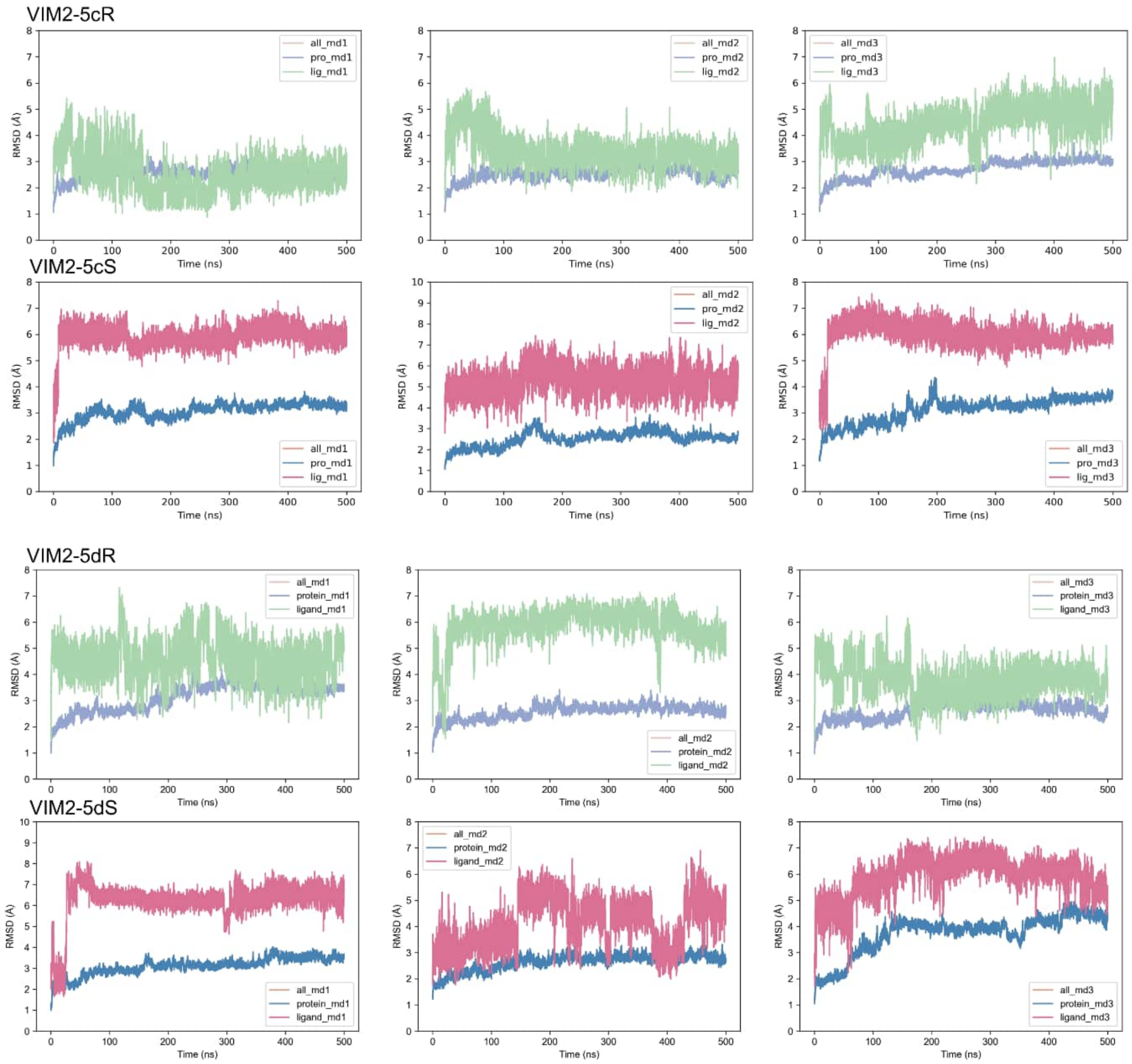

Figure S66. *RMSD of ligands, proteins and all heavy atoms for VIM2-**5c** and **5d** over 500 ns MD simulation in three repetitions.*

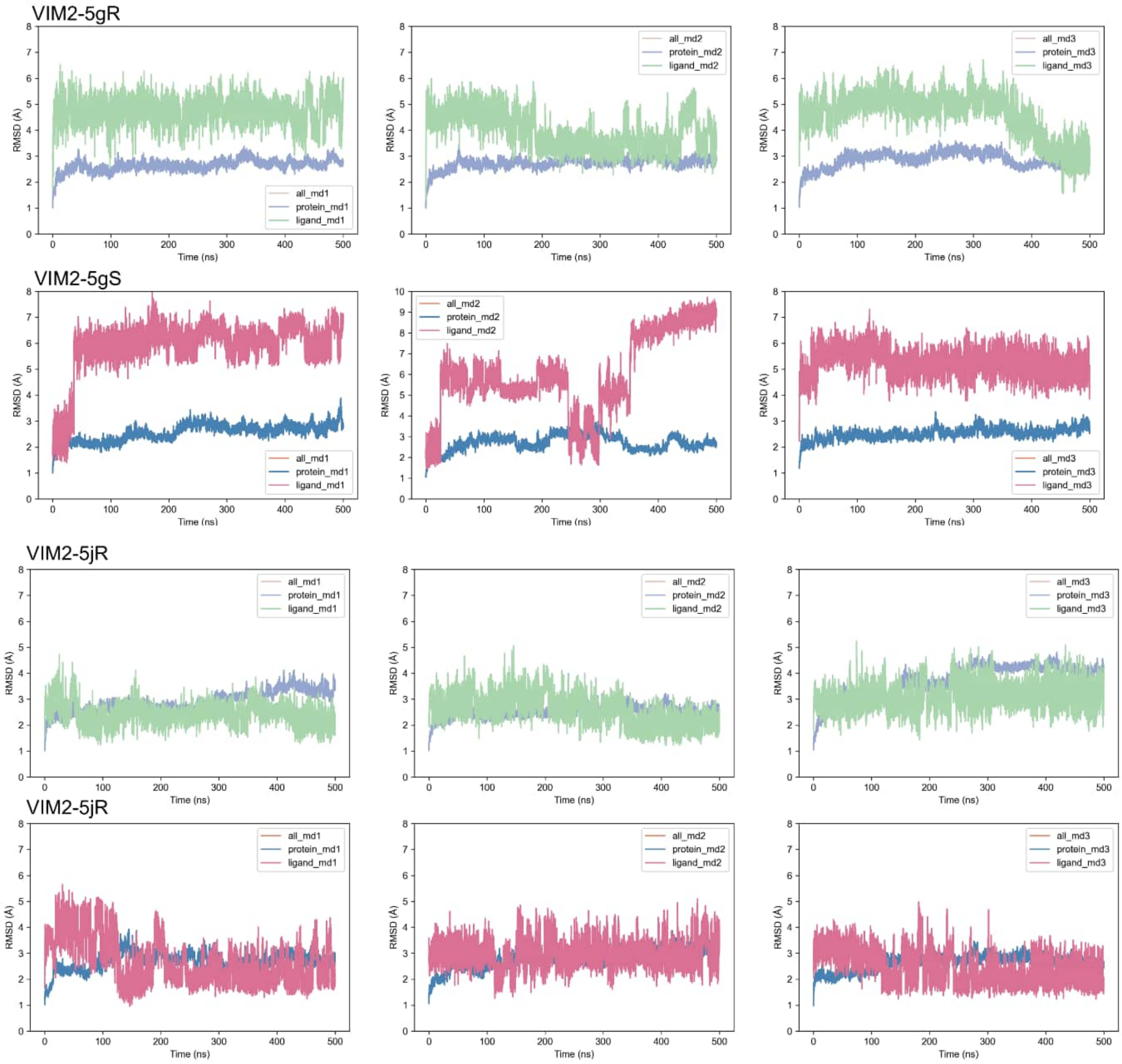

Figure S67. *RMSD of ligands, proteins and all heavy atoms for VIM2-**5g** and **5j** over 500 ns MD simulation in three repetitions.*

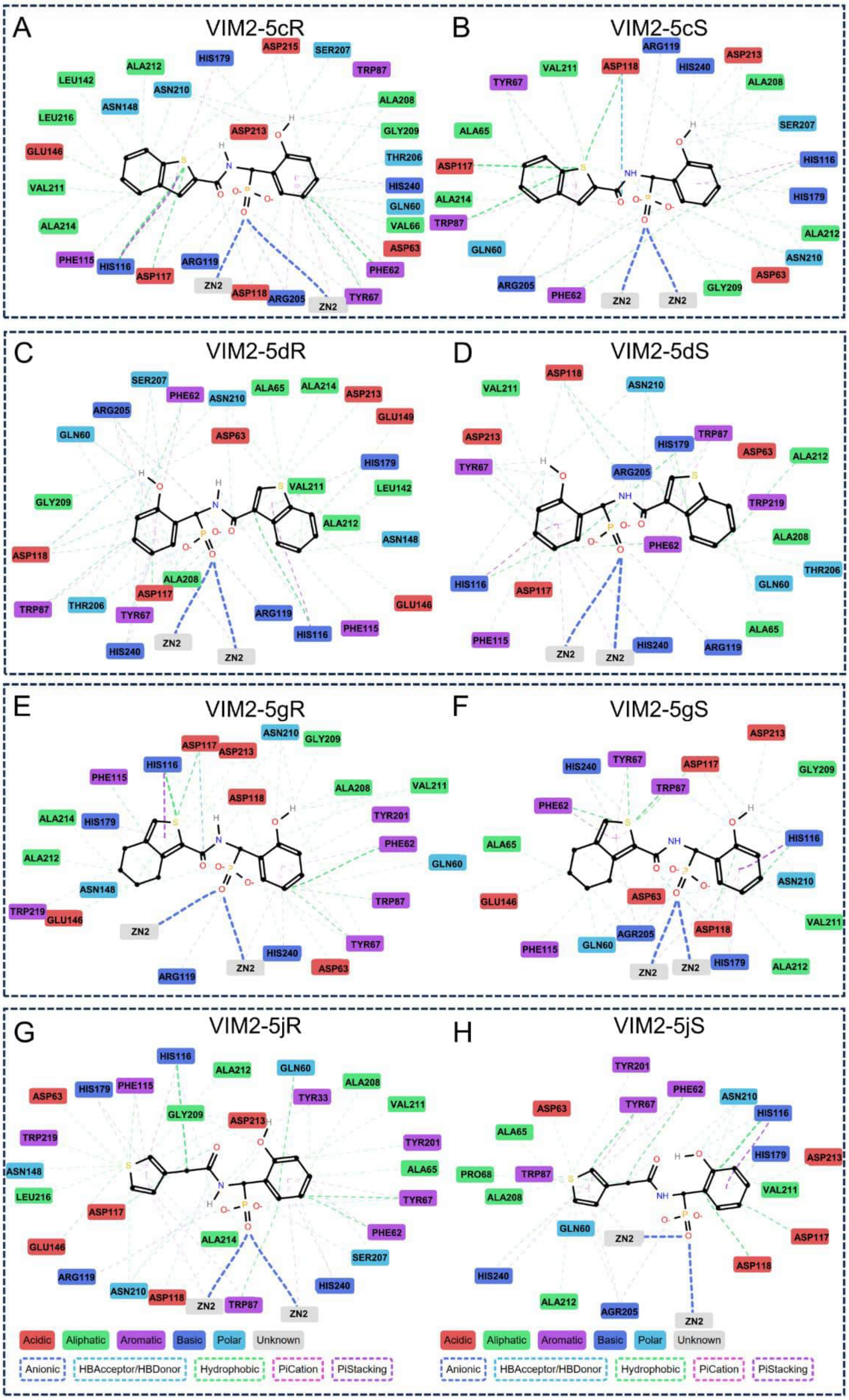

Figure S68. *The interaction patterns between VIM-2 and the ligands* ***5c****,* ***5d****,* ***5g*** *and* ***5j****. The colour of lines represents different types of interaction and the width of the lines depends on the interaction frequency over the whole simulation time.*

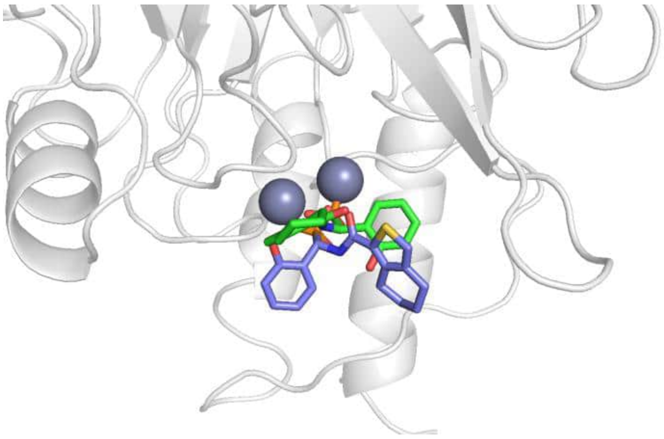

Figure S69. *The binding pose for compound* ***5g*** *(S)-isomer against VIM-2 before (green) and after (purple) MD simulation.*

Saturation mutagenesis followed by molecular docking experiments

Table S32. *Amino acids located in the VIM-2 binding pocket.*

F62	D63	Y67	P68	S69	W87	F115	D117	R119	E146
N148	Y201	R205	S207	A208	G209	N210	V211	A212	D213

Since H240, C198, D118, H179, H116, and H114 on VIM-2 are key amino acids for Zn ion binding, mutations in these amino acids were not considered.

Table S33. *Amino acids located in the NDM-1 binding pocket.*

L65	M67	F70	V73	W93	A121	Q123	K125	Q151
E152	G153	M154	K211	S217	L218	G219	N220	D223

Since H120, H122, H189, D124, C208, and H250 on NDM-1 are key amino acids for Zn ion binding, mutations in these amino acids were not considered.

Table S34. *Amino acids located in the GIM-1 binding pocket.*

K59	I61	Y64	V67	D68	S69	W87	E119	R121
E149	K165	T197	R224	W228	L231	G232	Y233	D236

Since H116, H118, H196, D120, C221, and H263 on GIM-1 are key amino acids for Zn ion binding, mutations in these amino acids were not considered.

Table S35. *The number of amino acid mutation types that reduce inhibitor binding affinity.*

	VIM-2[a]	NDM-1[b]	GIM-1[c]
(*R*)-**5c**	37	106	44
(*S*)-**5c**	105	46	4
(*R*+*S*)-**5c**[d]	21	34	1
(*R*)-**5d**	26	80	79
(*S*)-**5d**	232	42	5
(*R*+*S*)-**5d**	19	26	2
(*R*)-**5g**	22	92	40
(*S*)-**5g**	138	33	11
(*R*+*S*)-**5g**	19	26	1
(*R*)-**5j**	17	71	42
(*S*)-**5j**	22	31	14
(*R*+*S*)-**5j**	10	20	3

[a] There are 380 amino acid mutation combinations in the VIM-2 pocket.

[b] There are 342 amino acid mutation combinations in the NDM-1 pocket.

[c] There are 342 amino acid mutation combinations in the GIM-1 pocket.

[d] The number of amino acid mutation combinations that simultaneously weakened the binding of R and S enantiomers.

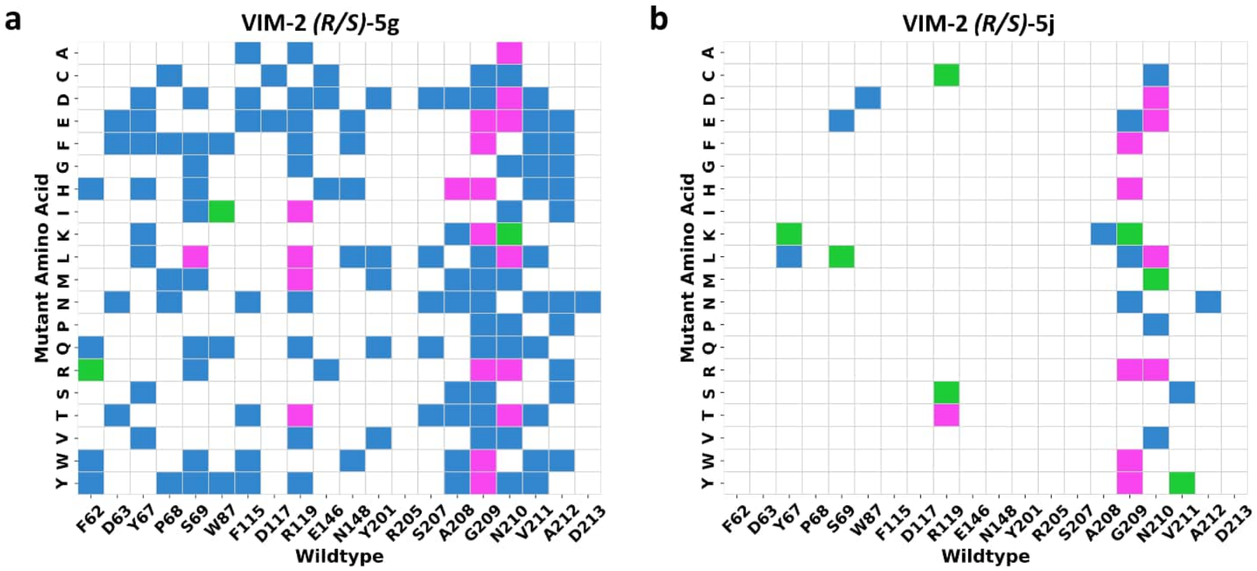

Figure S70. *Heatmaps showing amino acid mutations on VIM-2 that reduce the binding affinity of compounds* ***5g*** *(a) and* ***5j*** *(b). The x-axis represents the wildtype amino acids, while the y-axis shows the mutant amino acids. Blue cells indicate mutations that reduce the binding of the (S)-enantiomer, green cells represent those that weaken the binding of the (R)-enantiomer, and red-highlighted cells denote mutations that reduce the binding of both (S)- and (R)-enantiomers.*

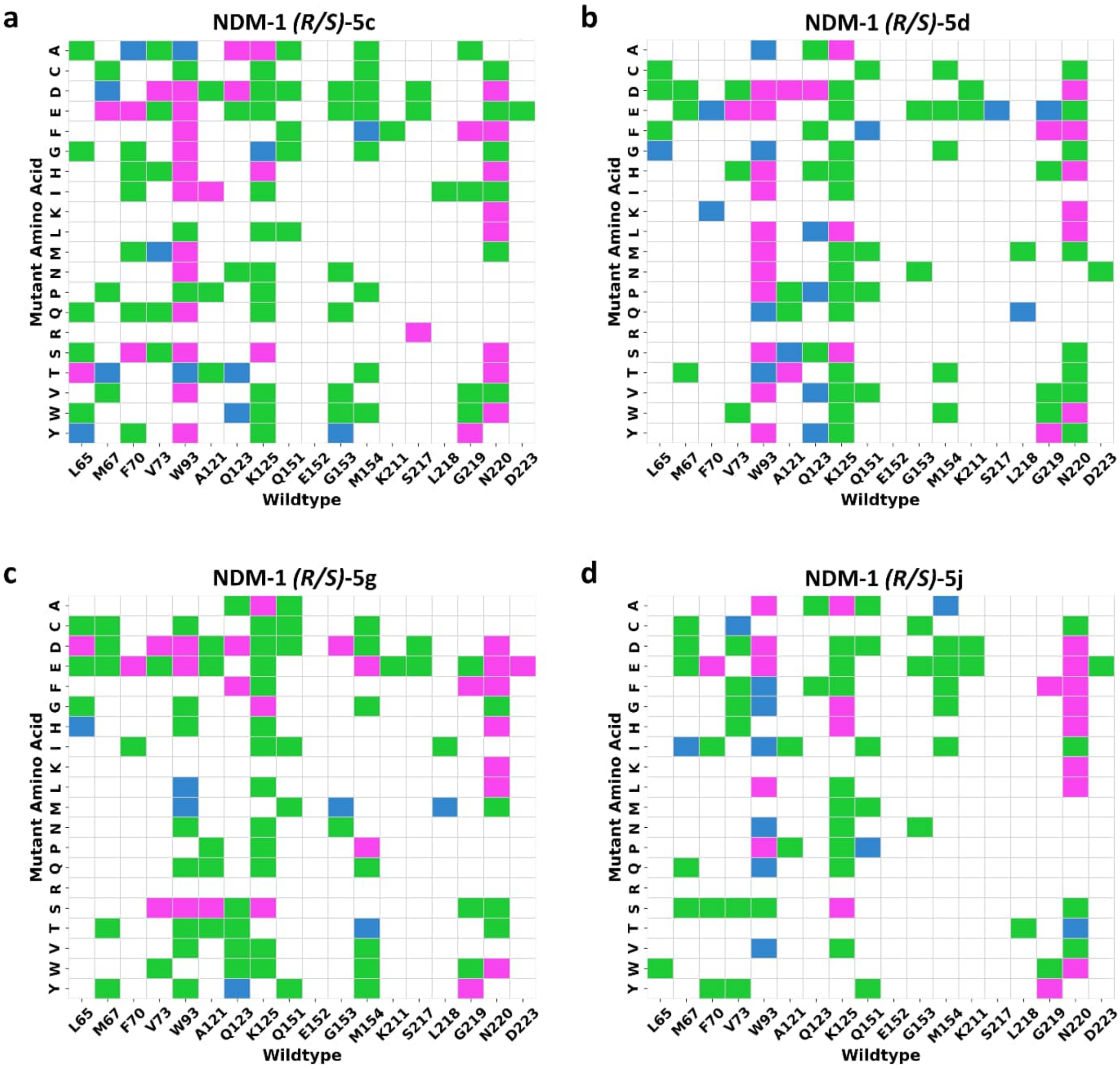

Figure S71. *Heatmaps showing amino acid mutations on NDM-1 that reduce the binding affinity of compounds* **5c** *(a),* **5d** *(b),* **5g** *(c) and* **5j** *(d). The x-axis represents the wildtype amino acids, while the y-axis shows the mutant amino acids. Blue cells indicate mutations that reduce the binding of the (S)-enantiomer, green cells represent those that weaken the binding of the (R)-enantiomer, and red-highlighted cells denote mutations that reduce the binding of both (S)- and (R)-enantiomers.*

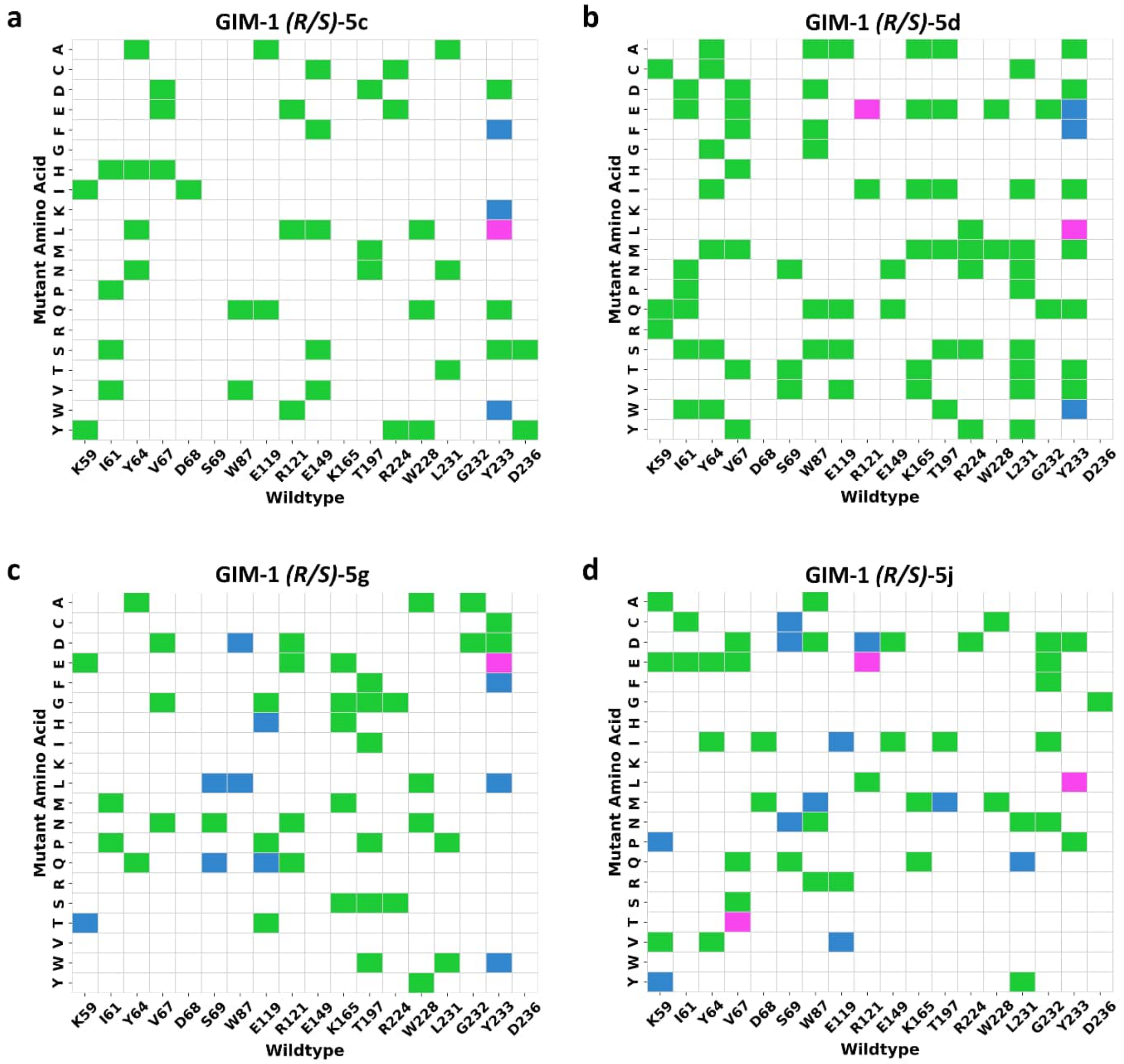

Figure S72. *Heatmaps showing amino acid mutations on GIM-1 that reduce the binding affinity of compounds **5c** (a), **5d** (b), **5g** (c) and **5j** (d). The x-axis represents the wildtype amino acids, while the y-axis shows the mutant amino acids. Blue cells indicate mutations that reduce the binding of the (S)-enantiomer, green cells represent those that weaken the binding of the (R)-enantiomer, and red-highlighted cells denote mutations that reduce the binding of both (S)- and (R)-enantiomers.*

15. Protein expression and purification

A previously described method was used for the expression and purification of uniformly ^{15}N-labeled VIM-2[6] and NDM-1[7].

The expression and purification yielded 23 mg of U-[^{15}N]-labeled VIM-2 batch 10 and 4 mg of U-[^{15}N]-labeled NDM-1 batch 19 per 1 L culture. The final concentration of ^{15}N-VIM-2 was 13.0 mg/ml and of ^{15}N-NDM-1 was 12.3 mg/ml. The final purity was estimated to be > 95% using SDS-PAGE analysis for both ^{15}N-VIM-2 batch 10 (**Figure S73**) and ^{15}N-NDM-1 batch 19 (**Figure S74**).

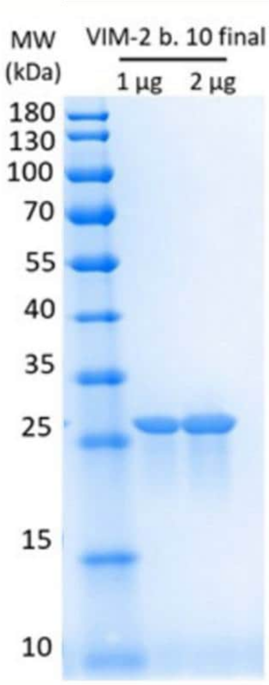

***Figure S73**. SDS-PAGE gel analysis of 1 and 2 µg of U-[^{15}N]-labeled VIM-1 batch 10 final product.*

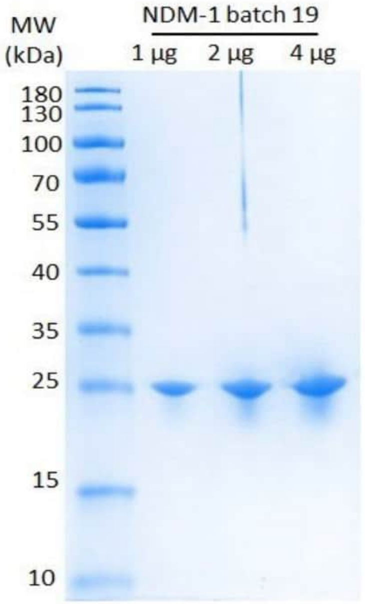

Figure S74. *SDS-PAGE gel analysis of 1, 2 and 4 µg of U-[^{15}N]-labeled NDM-1 batch 19 final product.*

16. NMR spectra

2-Methoxybenzaldehyde (1)

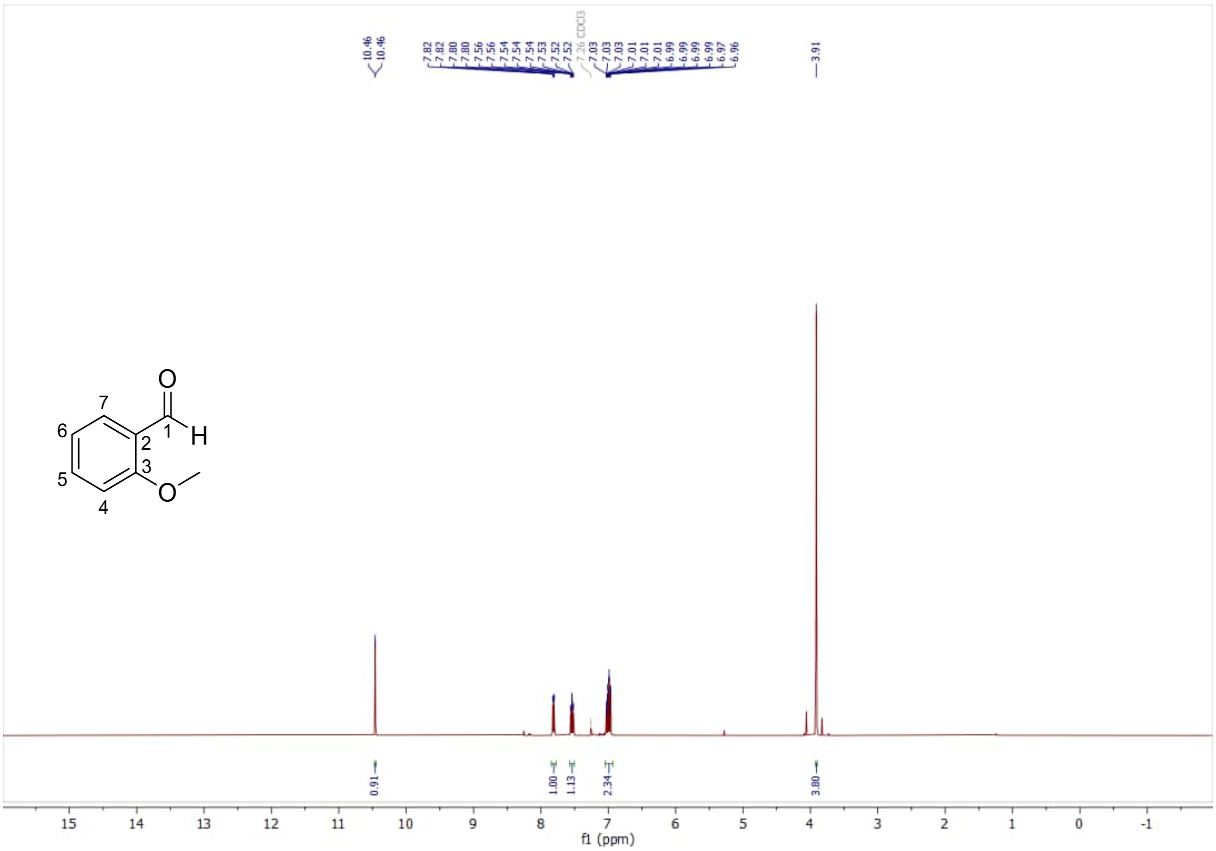

Figure S75. ^{1}*H NMR spectrum of* ***1*** *(400 MHz, CDCl$_3$).*

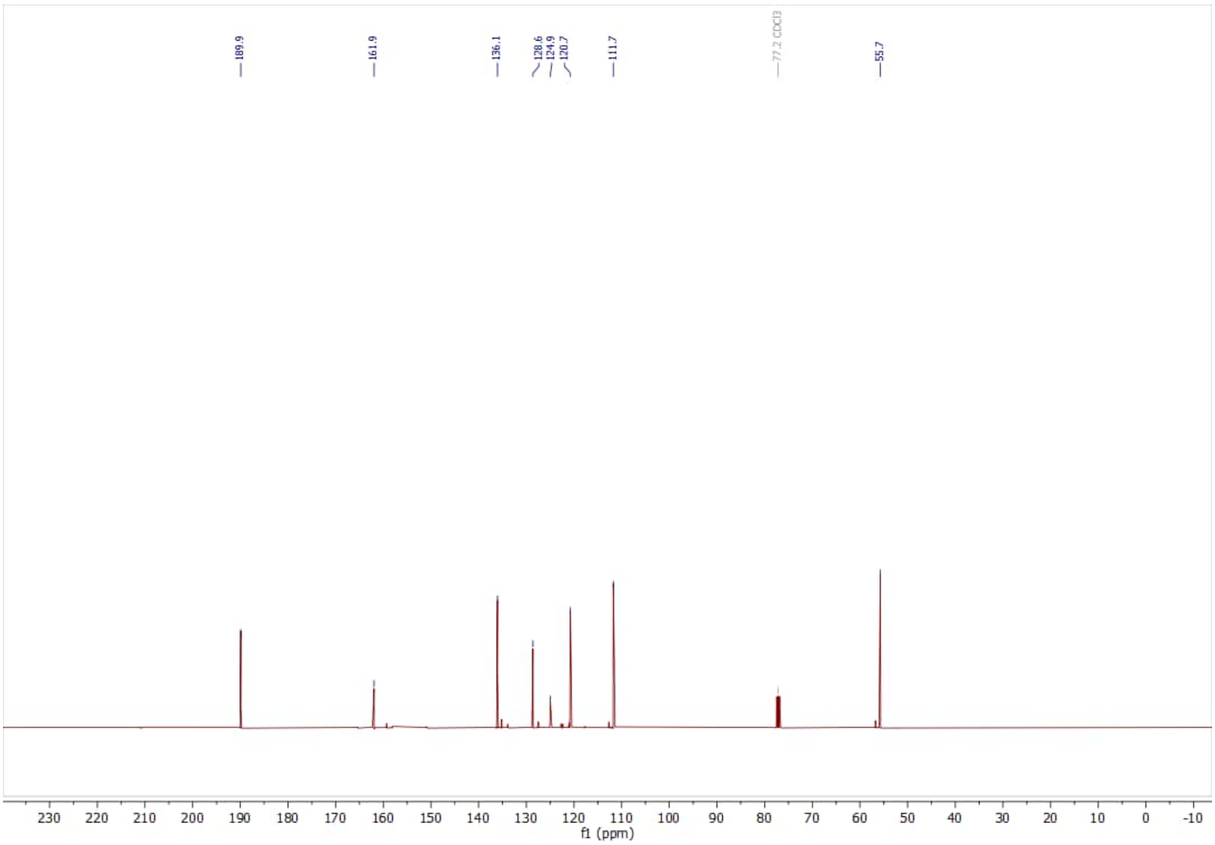

Figure S76. *^{13}C NMR spectrum of* ***1*** *(400 MHz, $CDCl_3$).*

Diethyl ((benzylamino)(2-methoxyphenyl)methyl)phosphonate (2)

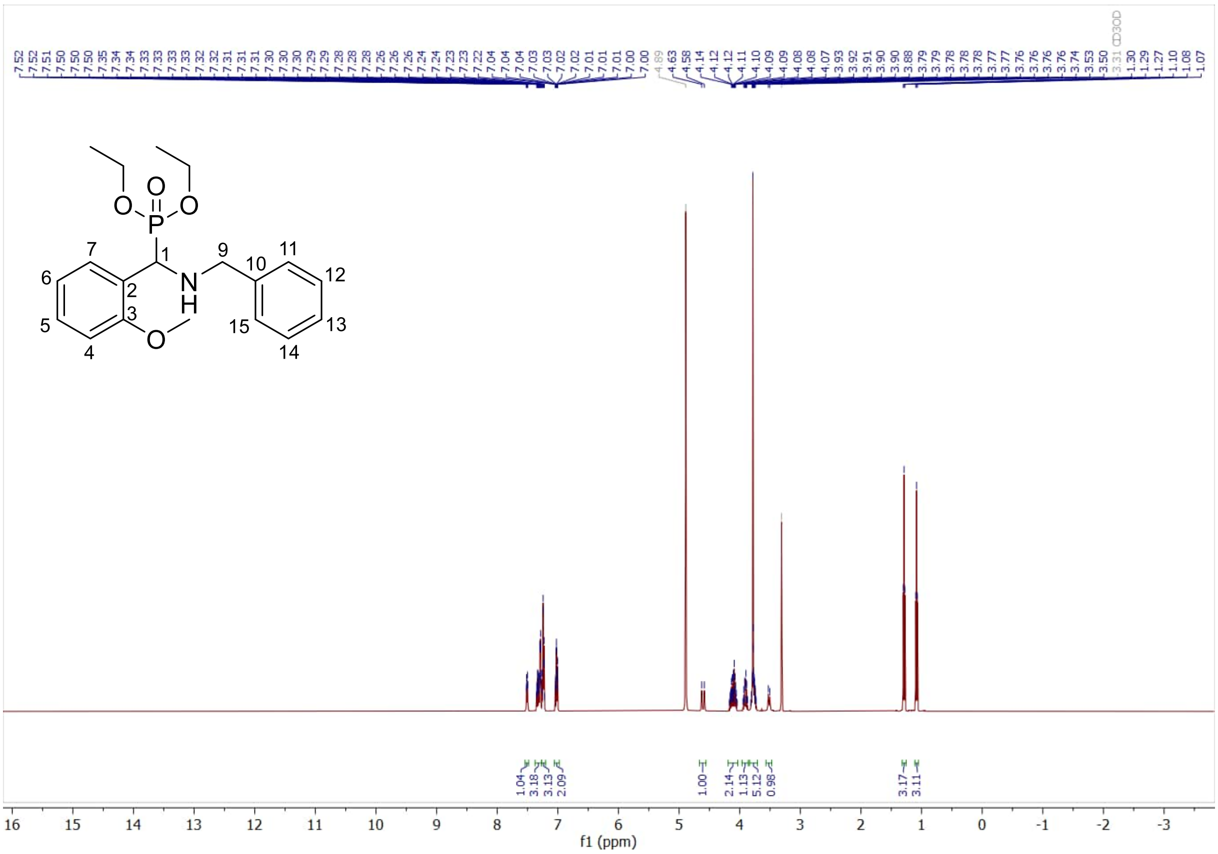

Figure S77. *^{1}H NMR spectrum of* ***2*** *(500 MHz, CD_3OD).*

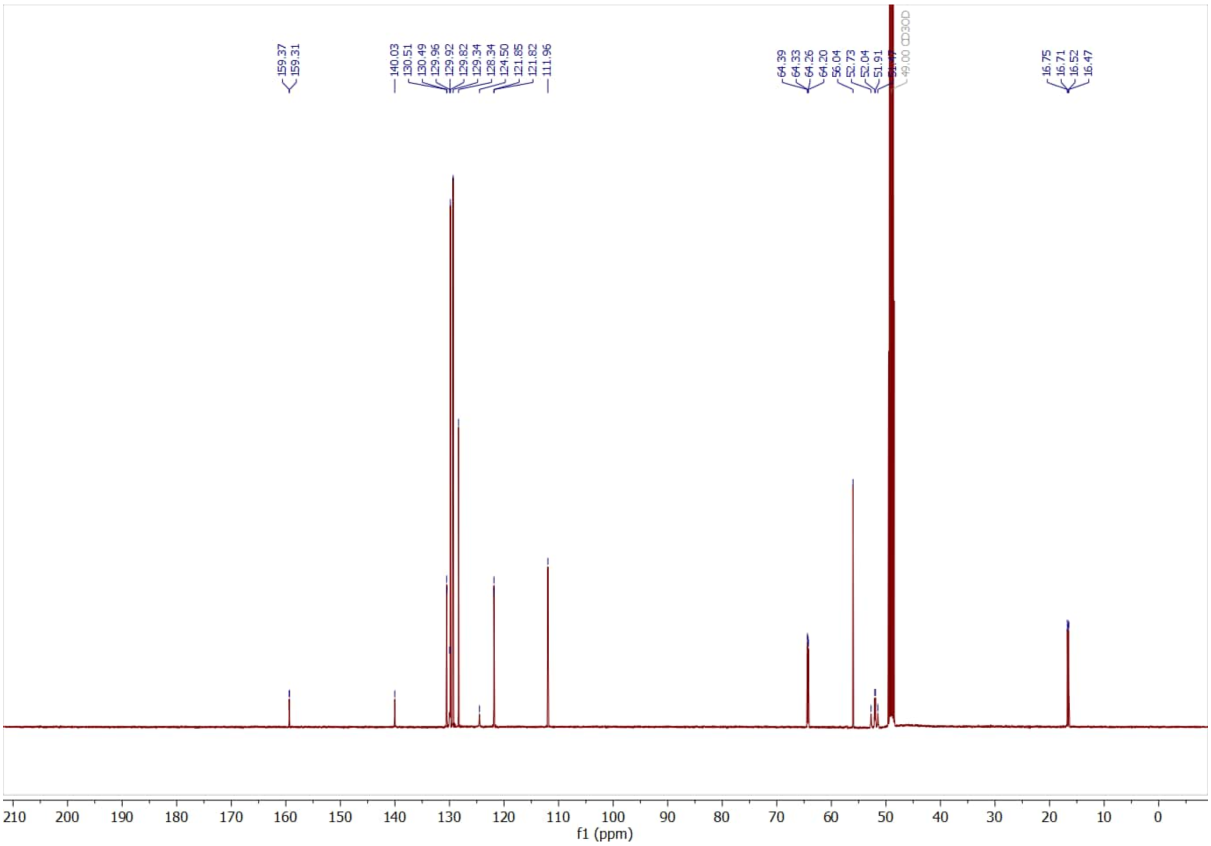

Figure S78. *^{13}C NMR spectrum of **2** (500 MHz, CD_3OD).*

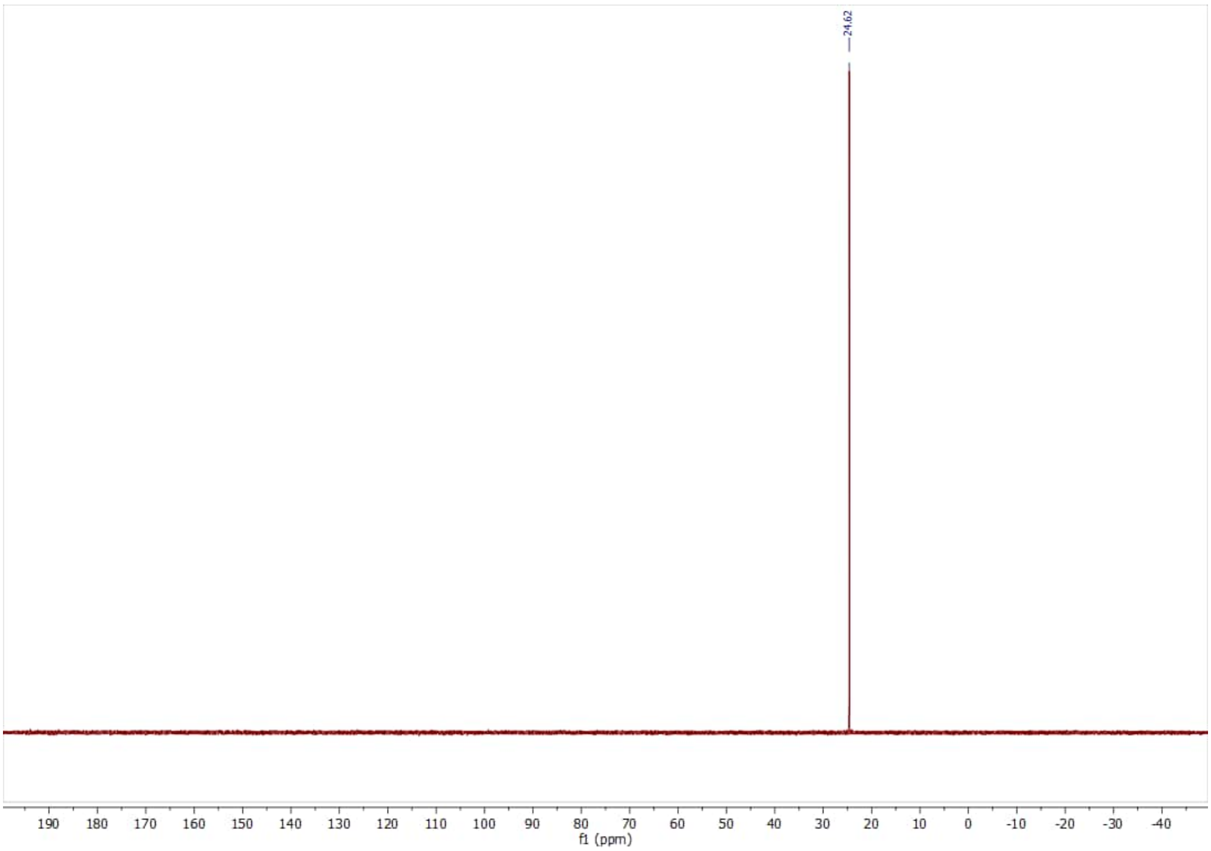

Figure S79. *^{31}P NMR spectrum of **2** (400 MHz, CD_3OD).*

Diethyl (amino(2-methoxyphenyl)methyl)phosphonate (3)

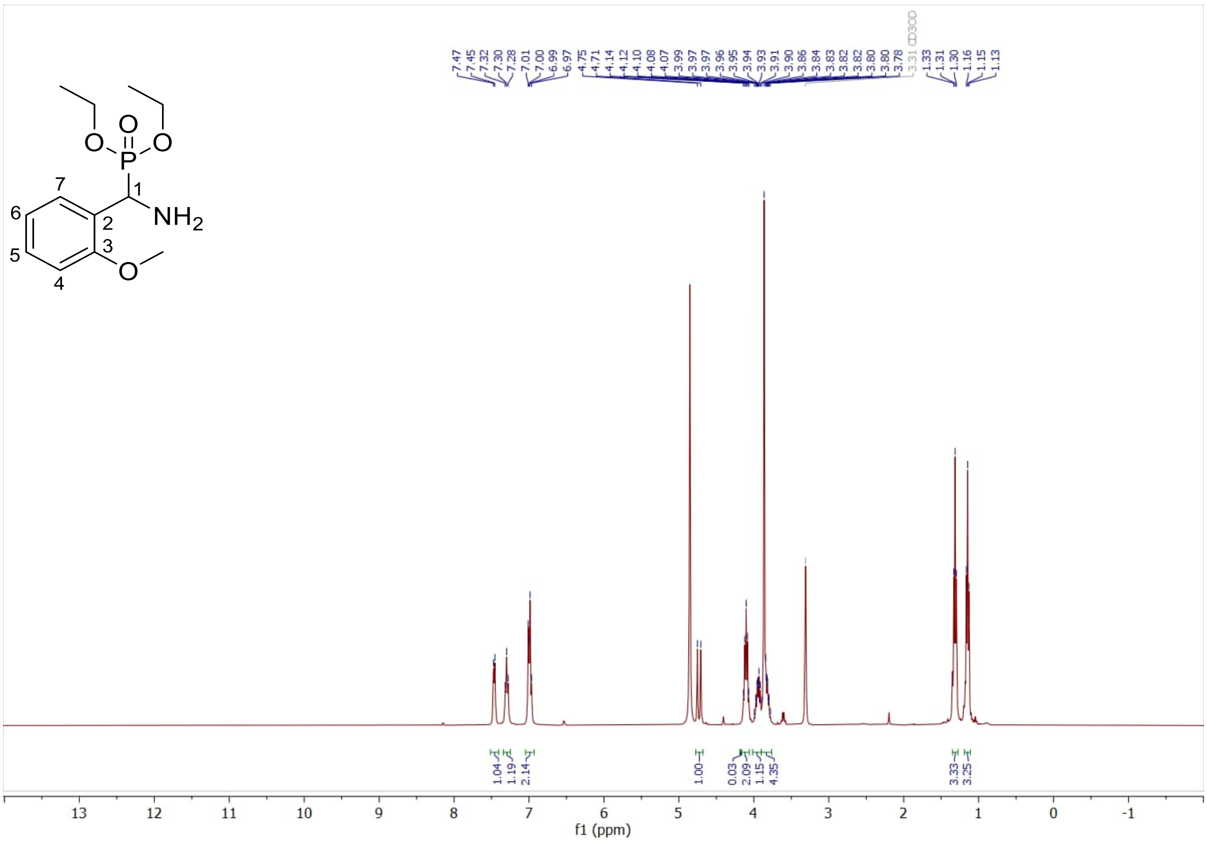

Figure S80. *^{1}H NMR spectrum of* ***3*** *(400 MHz, CD_3OD).*

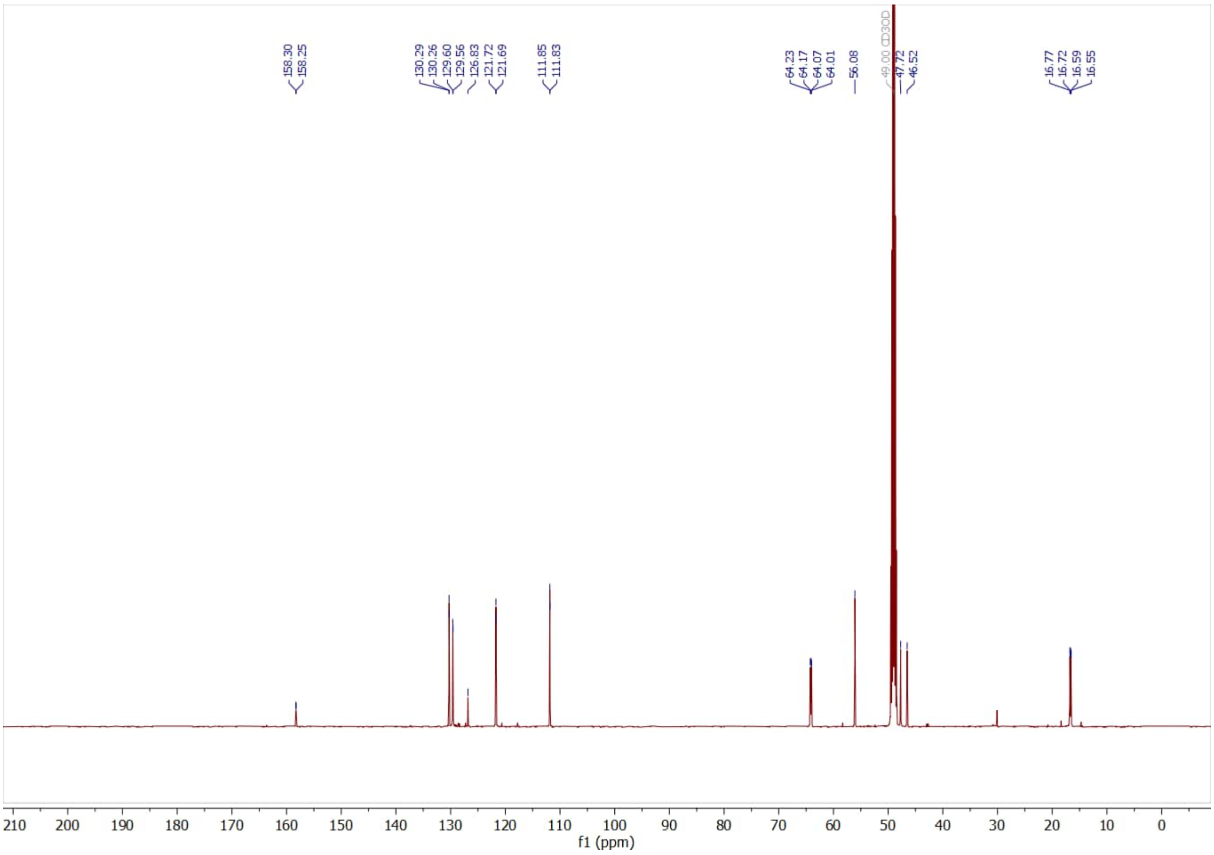

Figure S81. *^{13}C NMR spectrum of **3** (500 MHz, CD_3OD).*

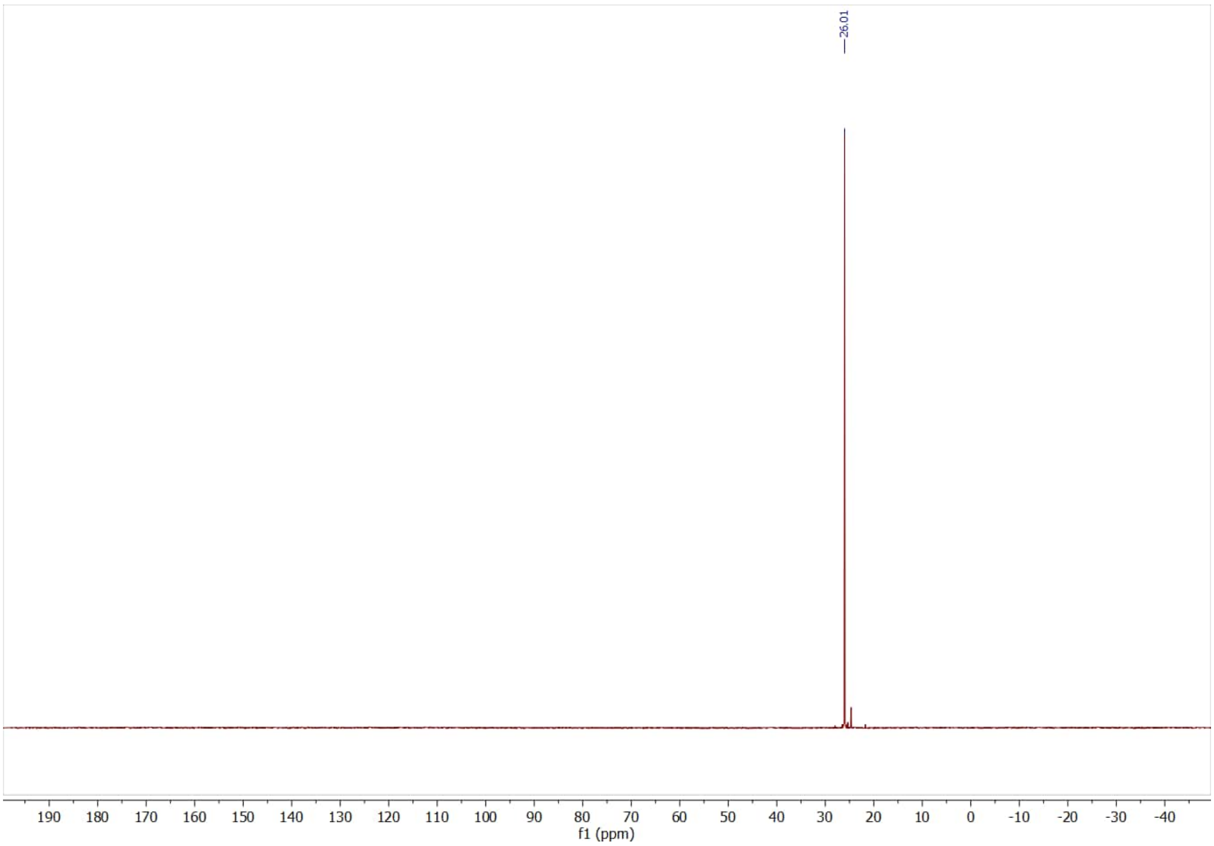

Figure S82. *^{31}P NMR spectrum of **3** (400 MHz, CD_3OD).*

Diethyl ((2-methoxyphenyl)(thiophene-2-carboxamido)methyl)phosphonate (4a)

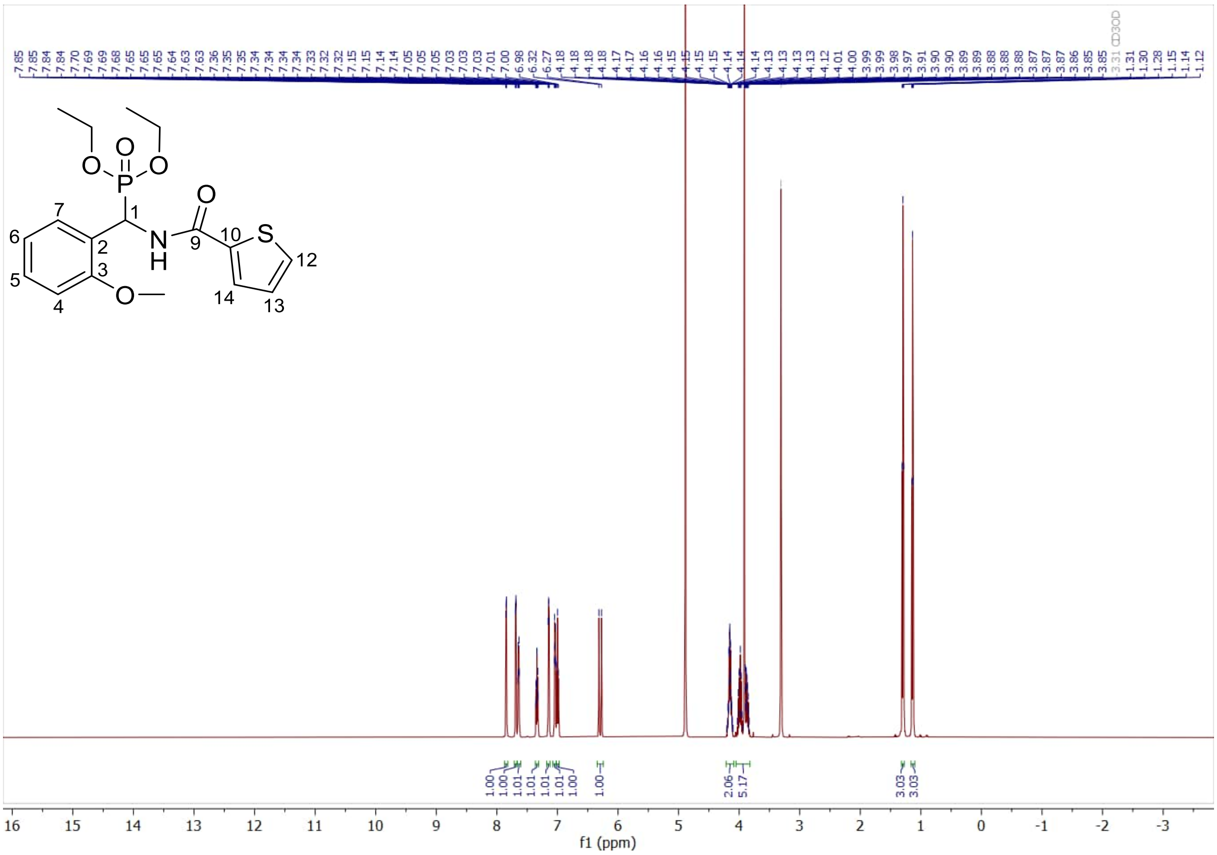

Figure S83. *^{1}H NMR spectrum of **4a** (500 MHz, CD_3OD).*

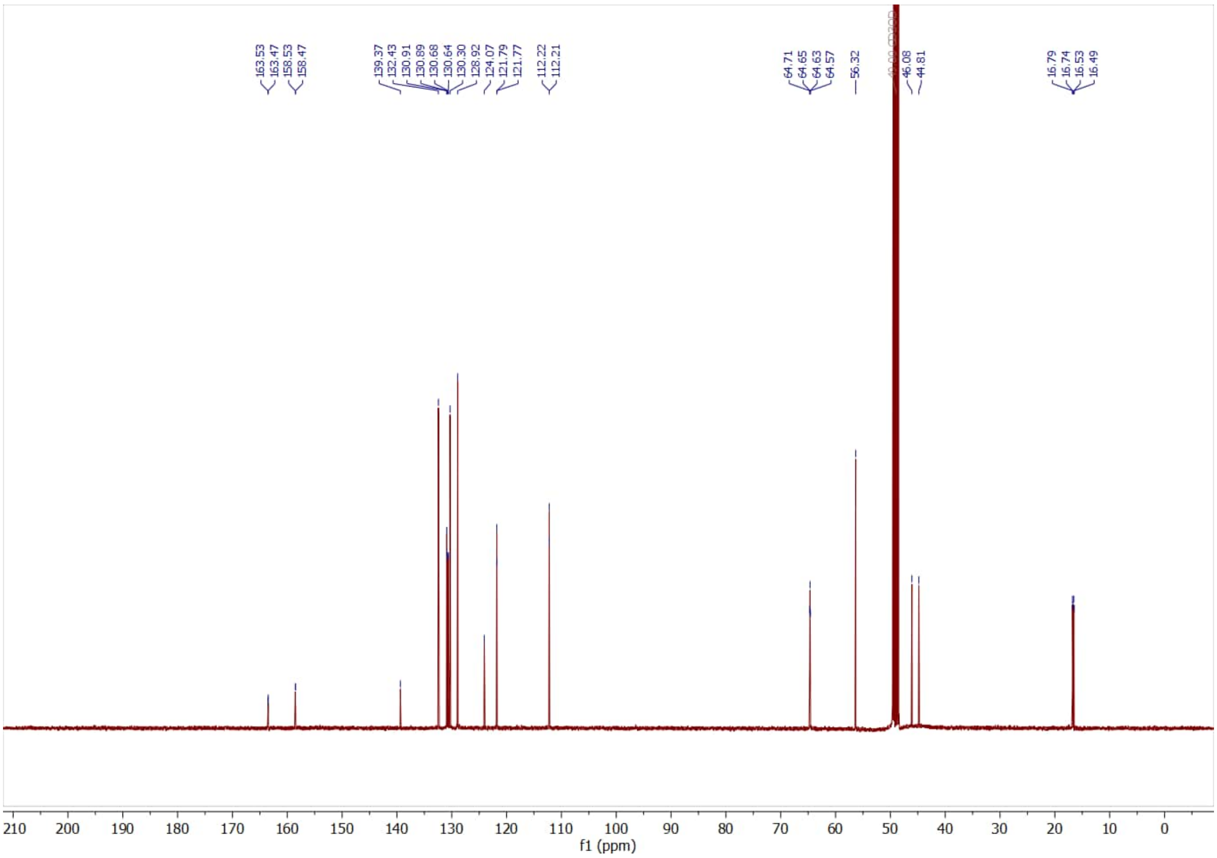

Figure S84. *^{13}C NMR spectrum of* ***4a*** *(500 MHz, CD_3OD).*

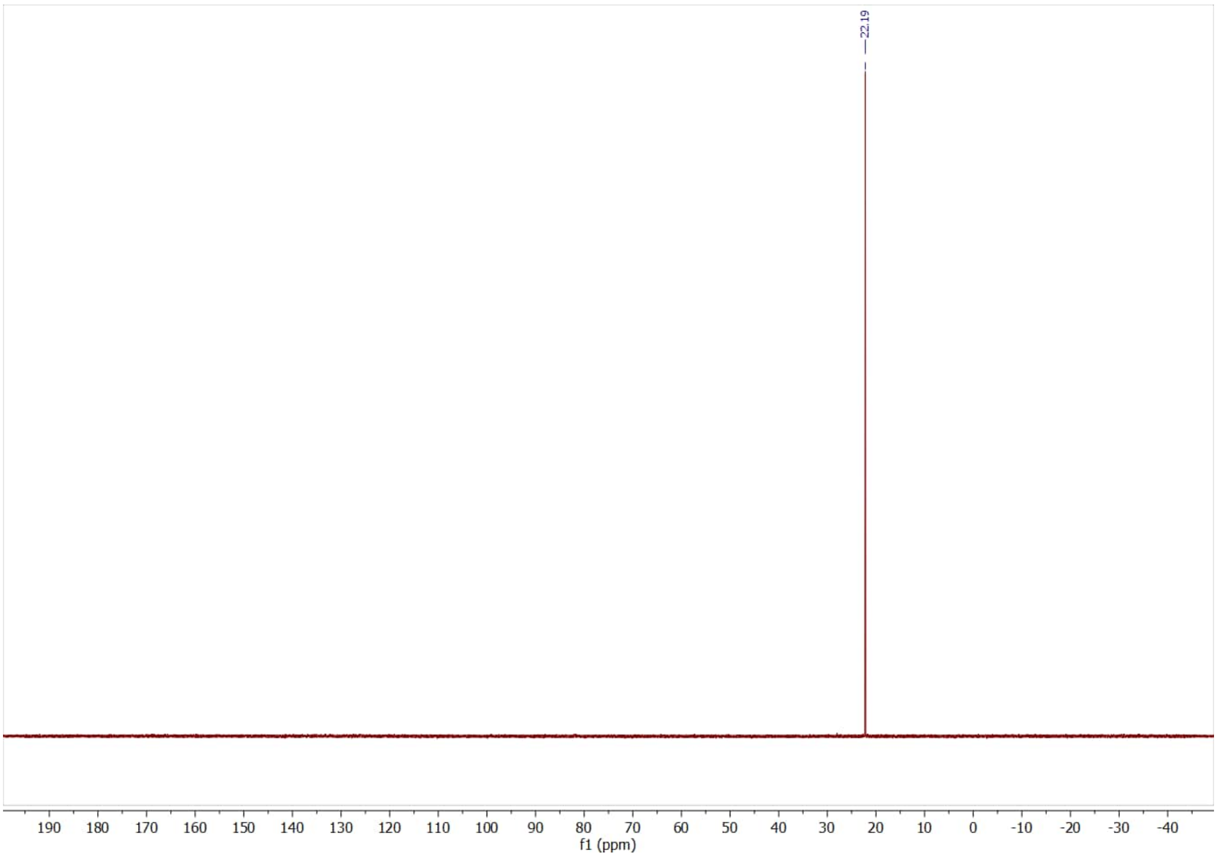

Figure S85. *^{31}P NMR spectrum of* ***4a*** *(400 MHz, CD_3OD).*

Diethyl ((2-methoxyphenyl)(thiophene-3-carboxamido)methyl)phosphonate (4b)

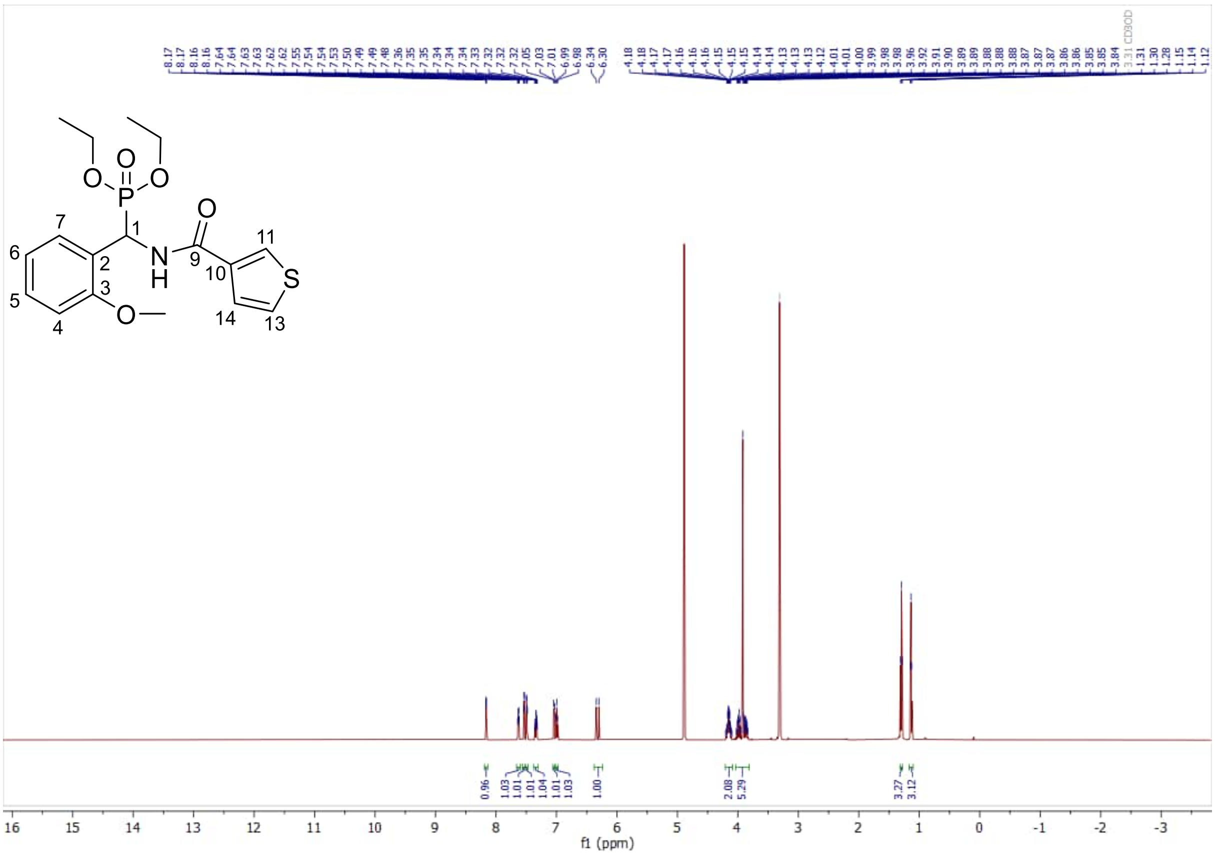

Figure S86. *^{1}H NMR spectrum of* ***4b*** *(500 MHz, CD_3OD).*

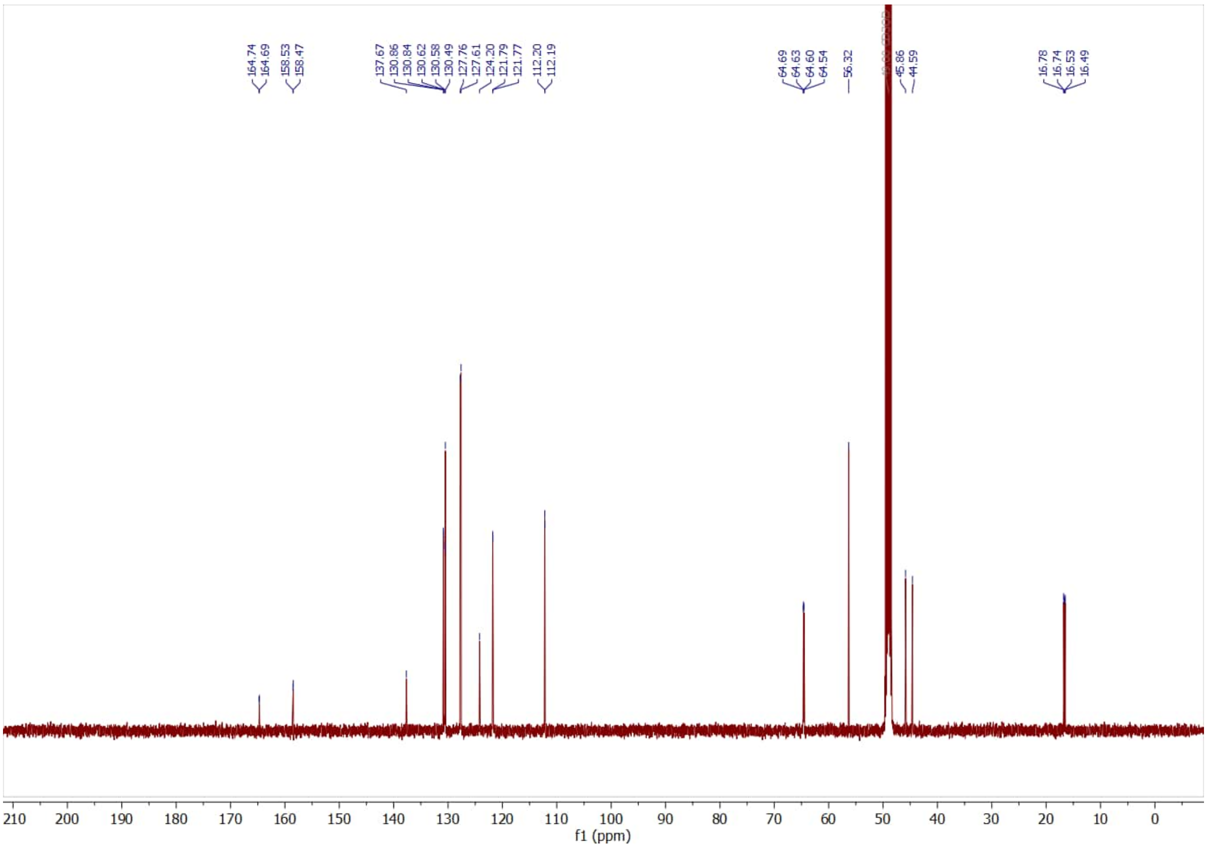

Figure S87. *^{13}C NMR spectrum of* ***4b*** *(500 MHz, CD_3OD).*

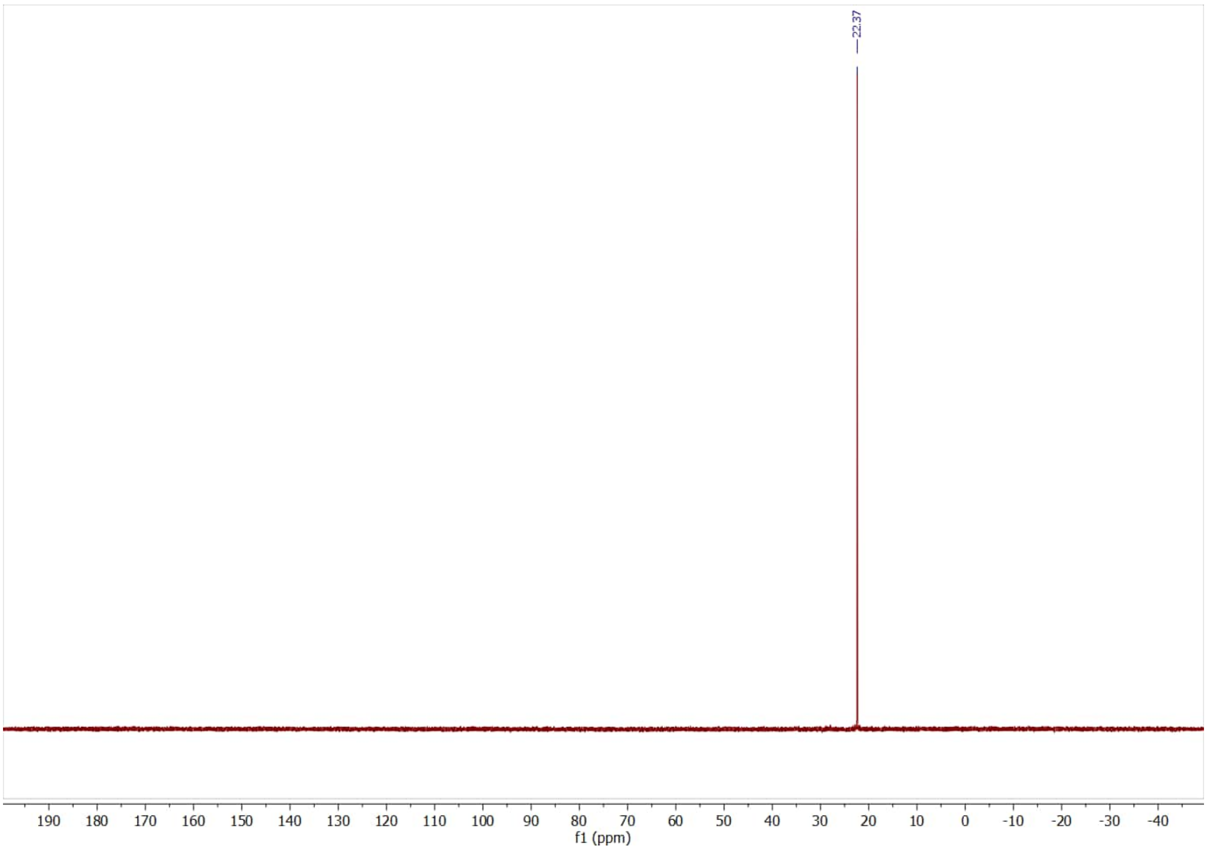

Figure S88. *^{31}P NMR spectrum of* ***4b*** *(400 MHz, CD_3OD).*

Diethyl ((benzo[*b*]thiophene-2-carboxamido)(2-methoxyphenyl)methyl)phosphonate (4c)

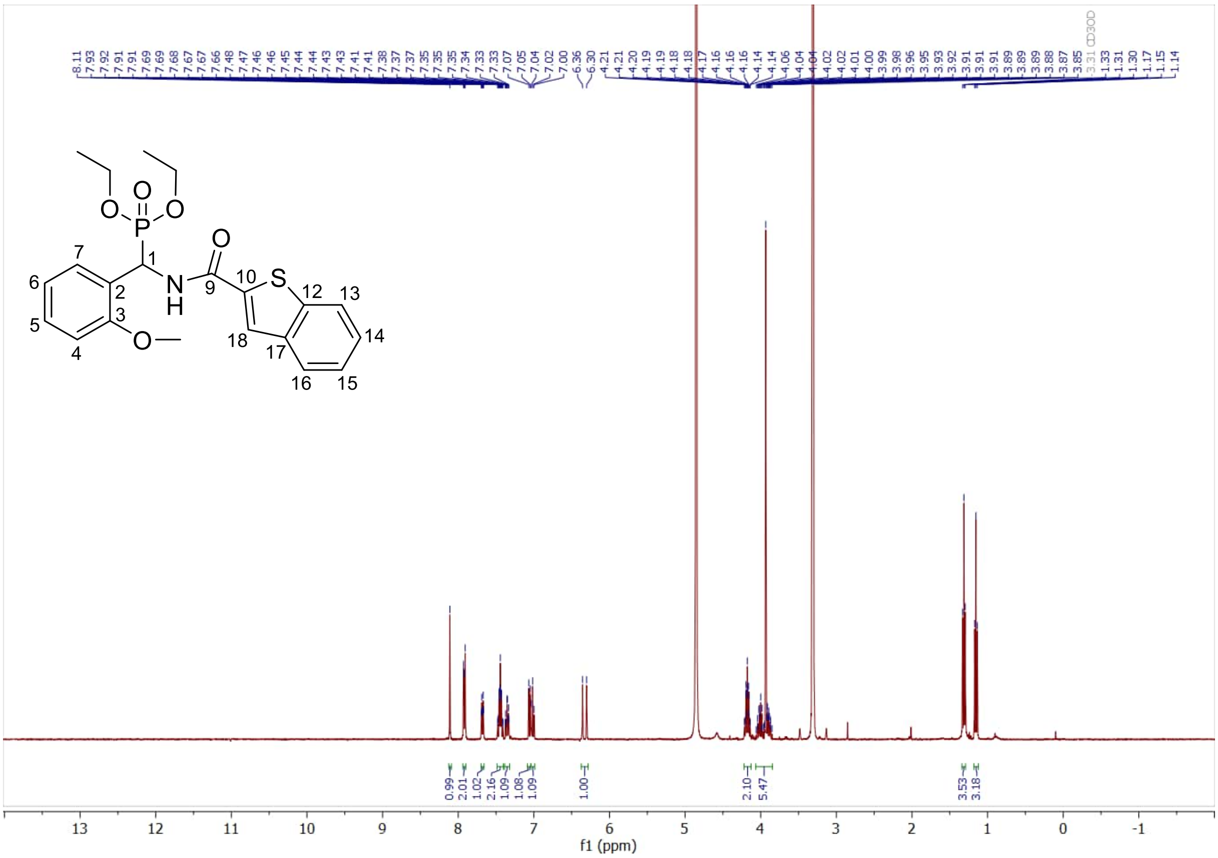

Figure S89. *^{1}H NMR spectrum of **4c** (400 MHz, CD_3OD).*

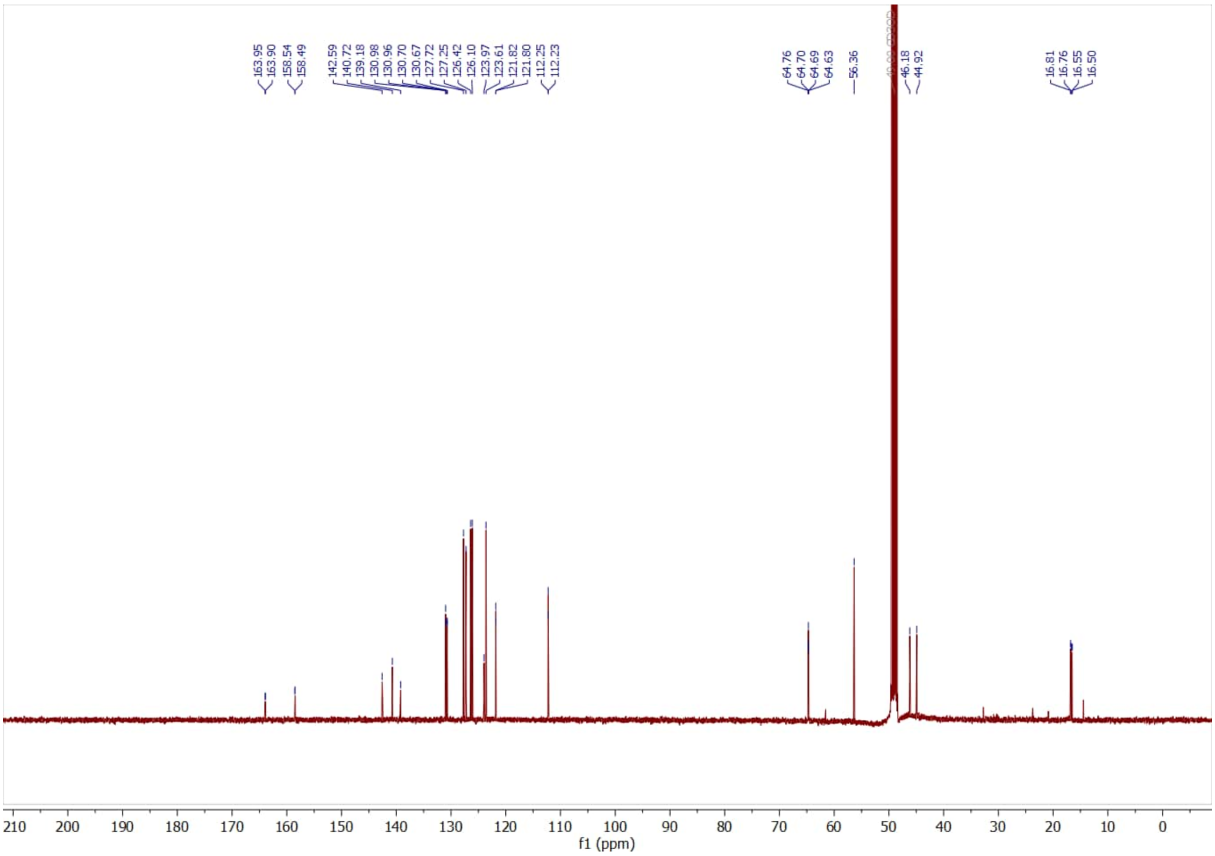

Figure S90. *^{13}C NMR spectrum of* ***4c*** *(500 MHz, CD_3OD).*

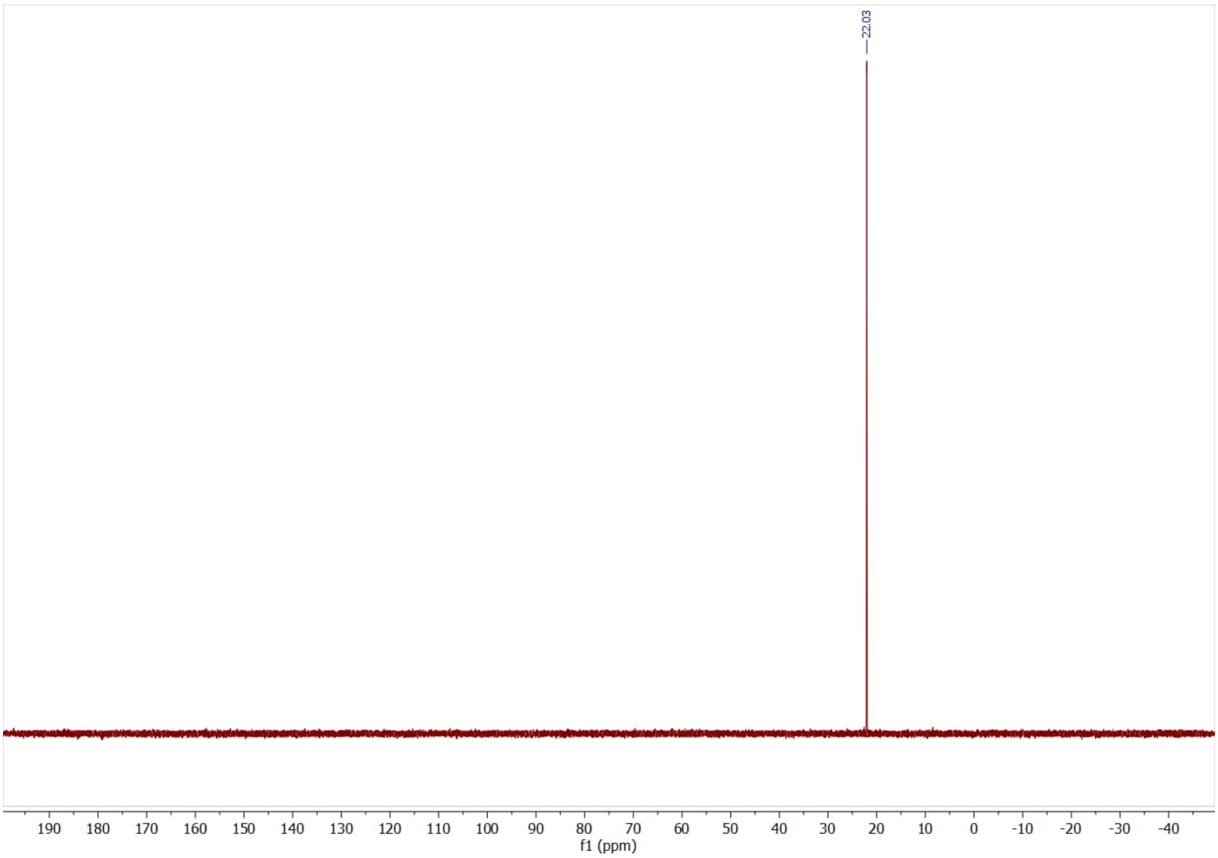

Figure S91. *^{31}P NMR spectrum of* ***4c*** *(400 MHz, CD_3OD).*

Diethyl ((benzo[*b*]thiophene-3-carboxamido)(2-methoxyphenyl)methyl)phosphonate (4d)

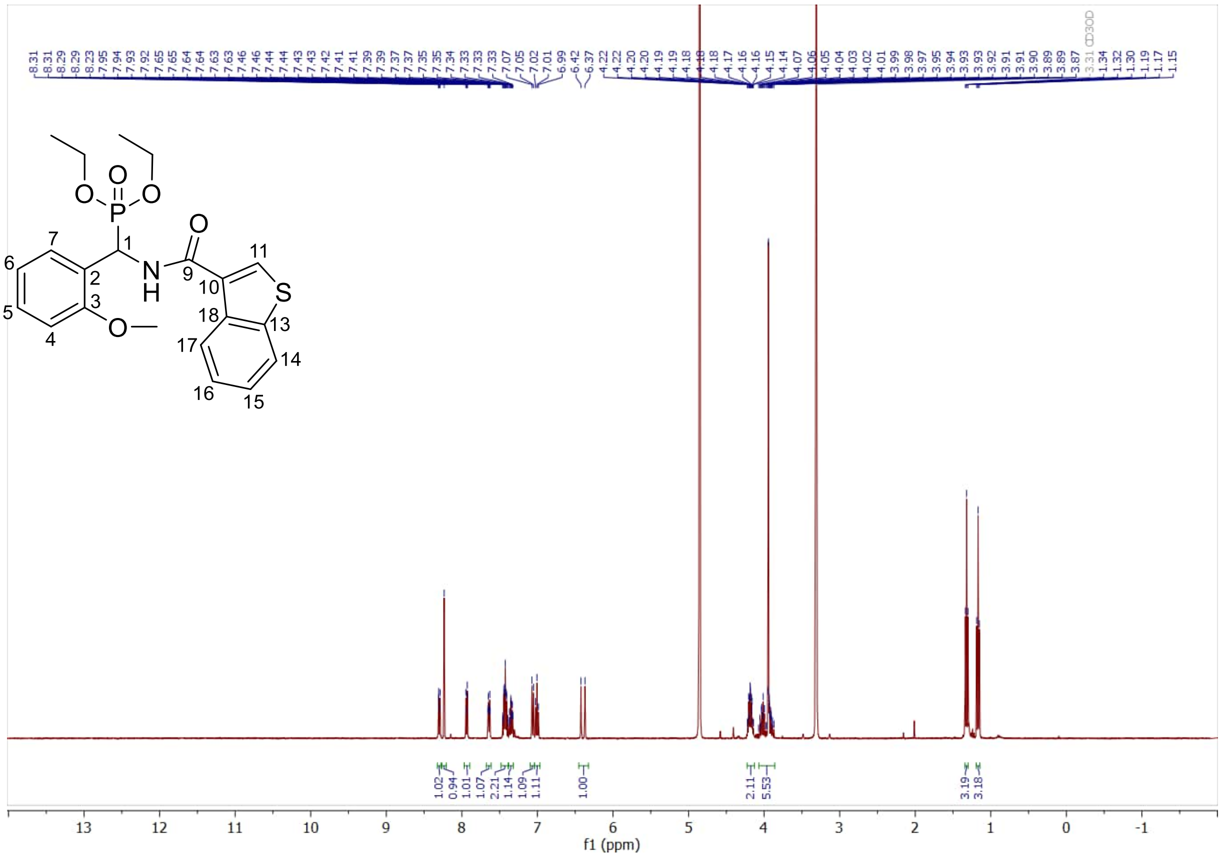

Figure S92. *^{1}H NMR spectrum of* ***4d*** *(400 MHz, CD_3OD).*

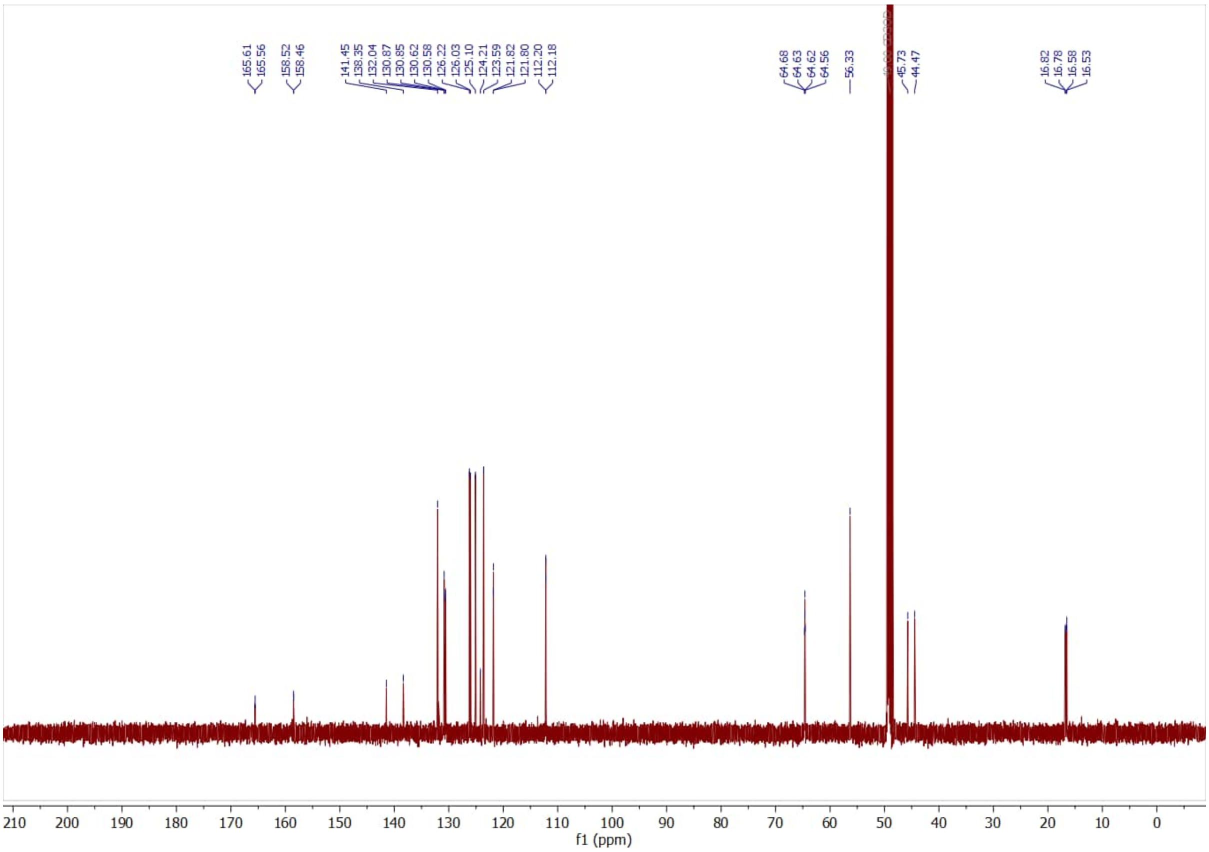

Figure S93. *^{13}C NMR spectrum of **4d** (500 MHz, CD_3OD).*

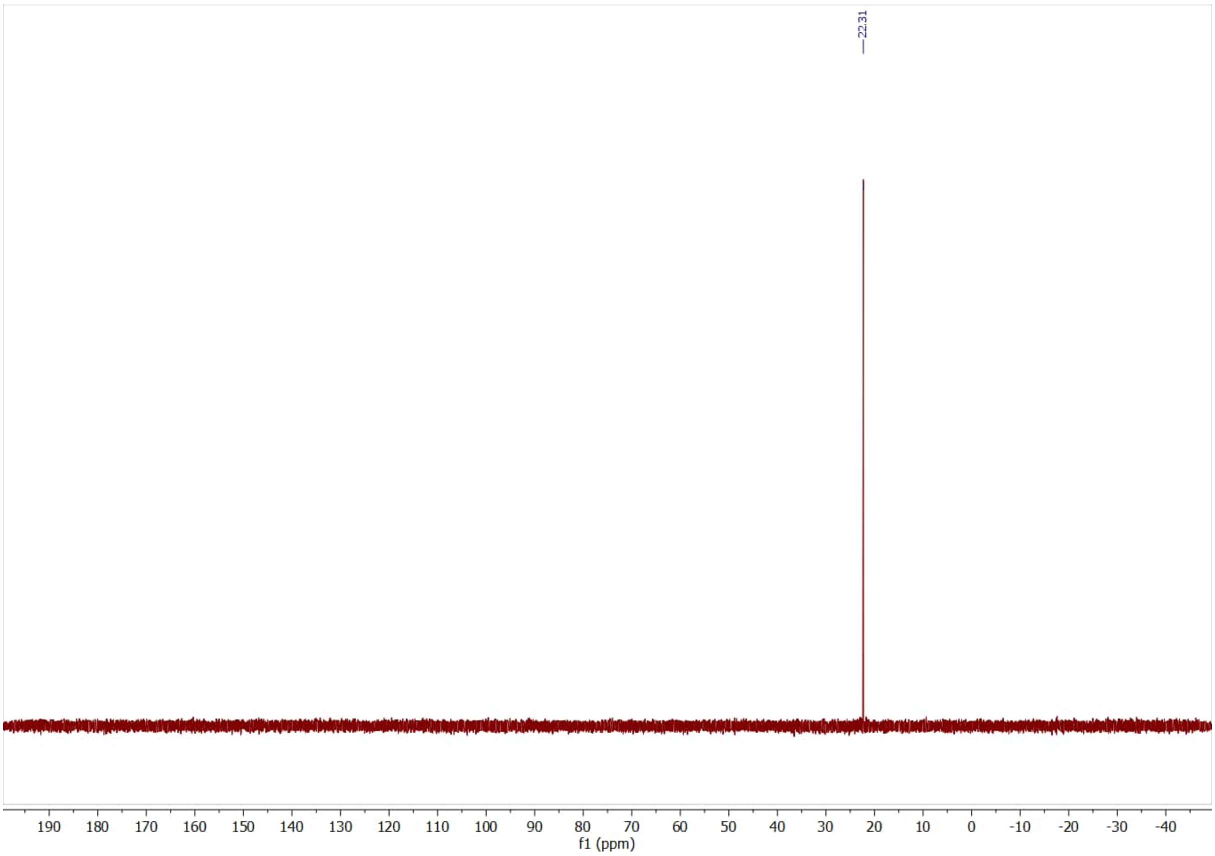

Figure S94. *^{31}P NMR spectrum of **4d** (400 MHz, CD_3OD).*

Diethyl ((2-methoxyphenyl)(4,5,6,7-tetrahydrobenzo[*b*]thiophene-2-carboxamido)methyl)phosphonate (4e)

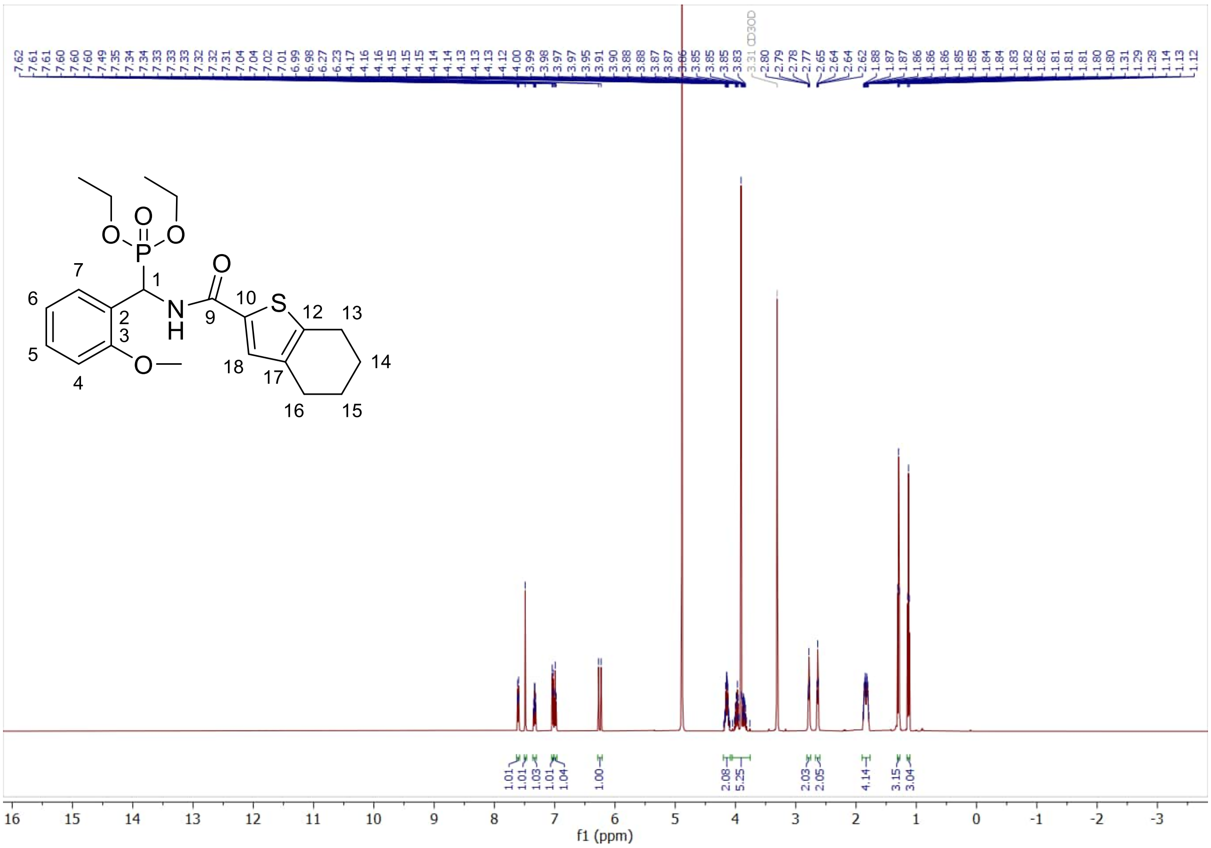

***Figure S95**. ¹H NMR spectrum of **4e** (500 MHz, CD_3OD).*

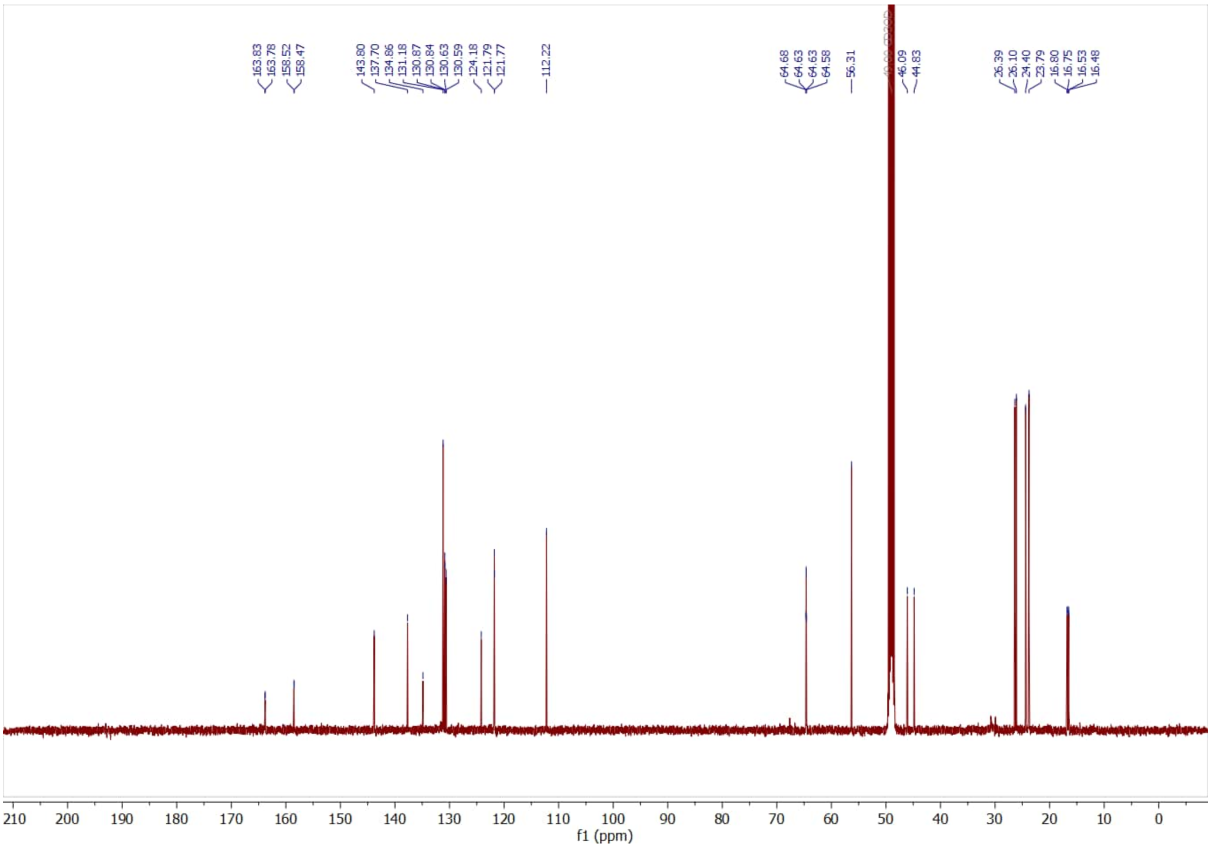

Figure S96. *^{13}C NMR spectrum of **4e** (500 MHz, CD_3OD).*

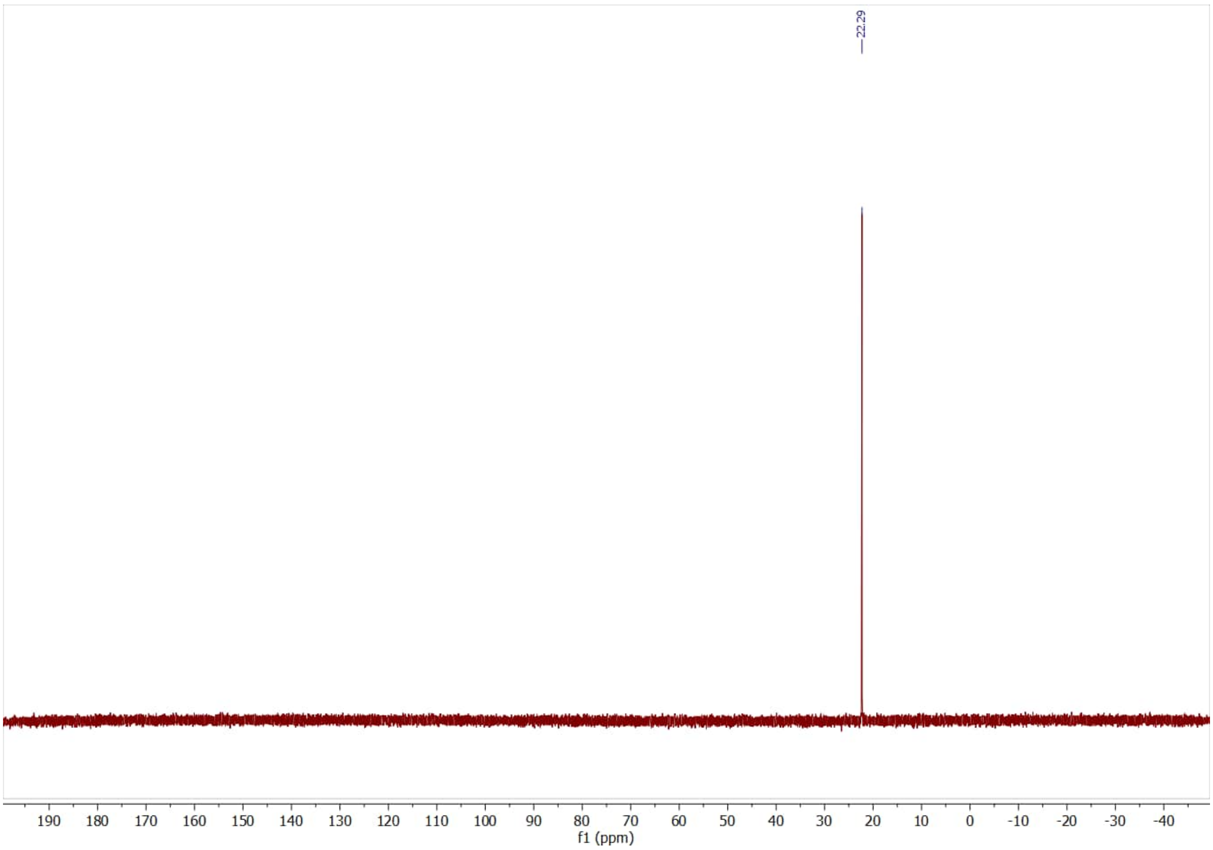

Figure S97. *^{31}P NMR spectrum of **4e** (400 MHz, CD_3OD).*

Diethyl ((2-methoxyphenyl)(4,5,6,7-tetrahydrobenzo[*b*]thiophene-3-carboxamido)methyl)phosphonate (4f)

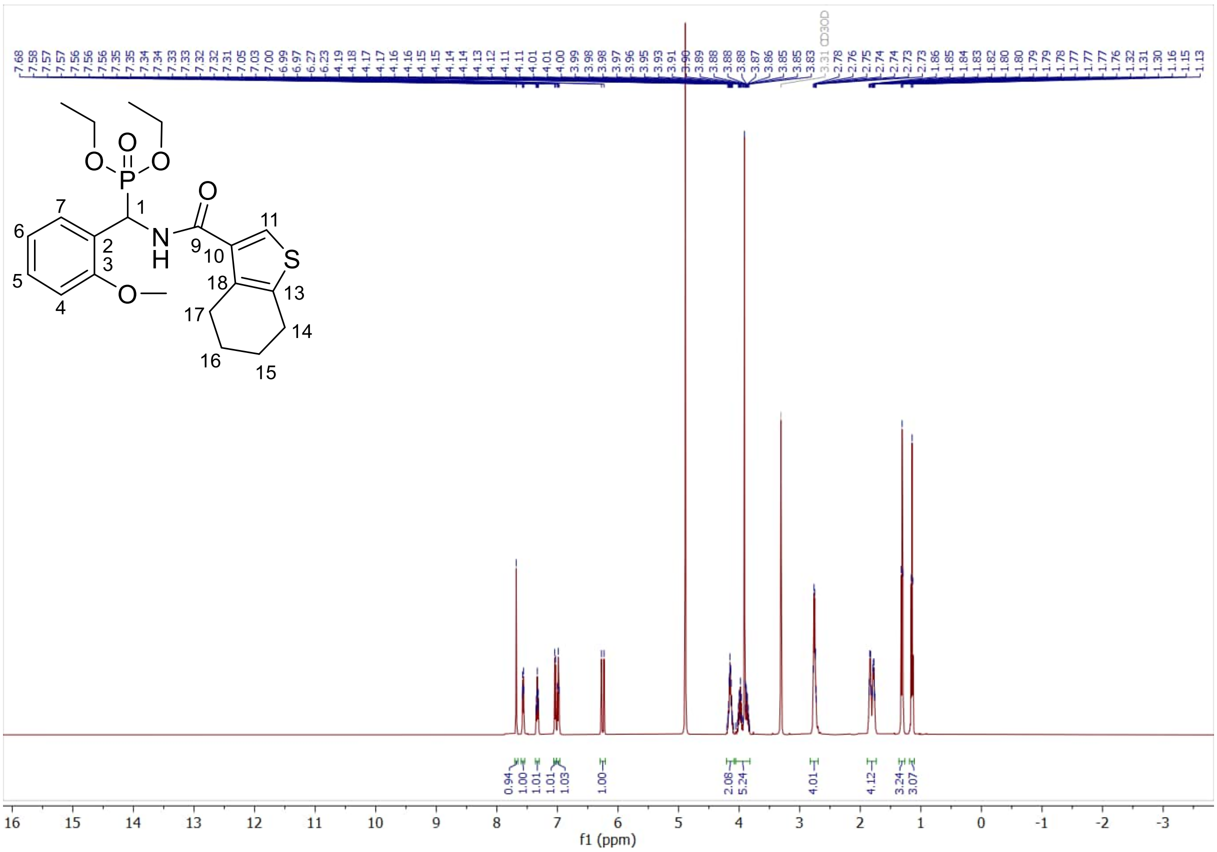

Figure S98. *^{1}H NMR spectrum of **4f** (500 MHz, CD_3OD).*

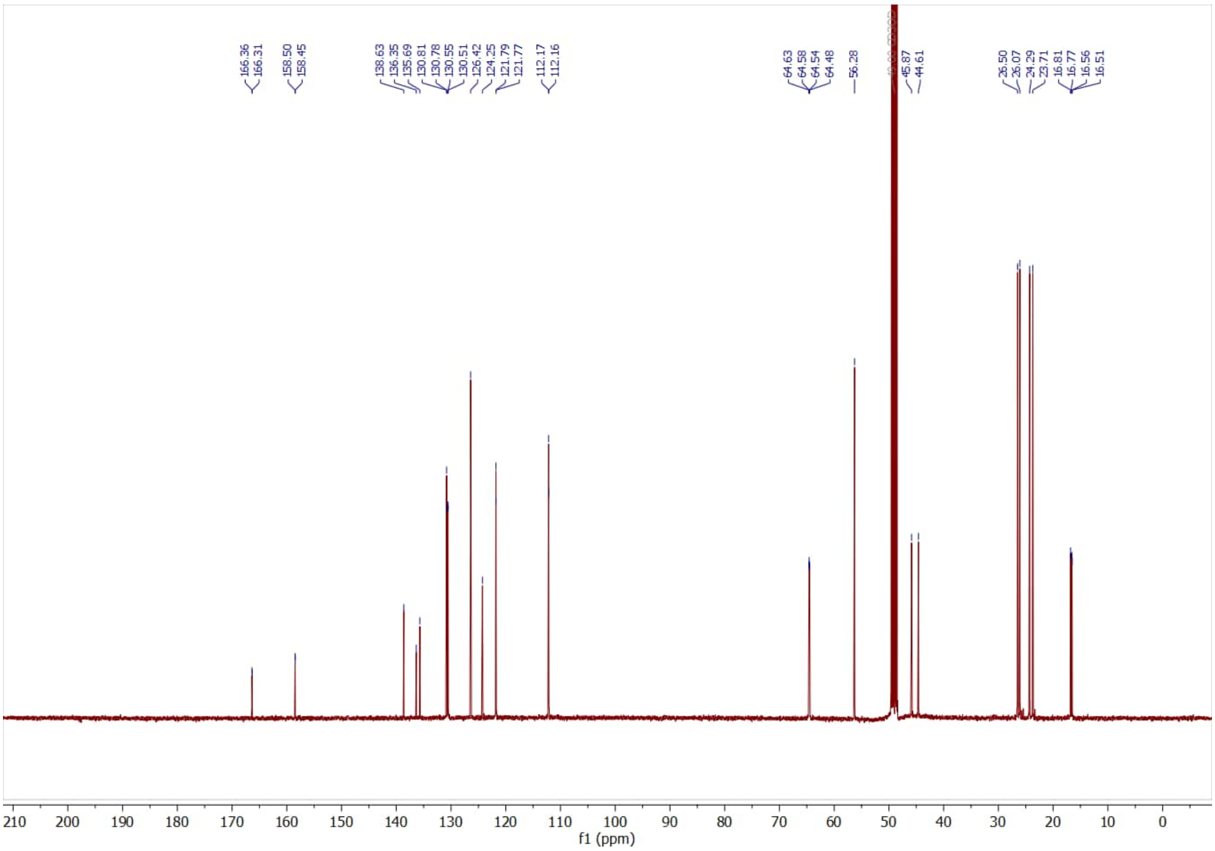

Figure S99. *^{13}C NMR spectrum of **4f** (500 MHz, CD_3OD).*

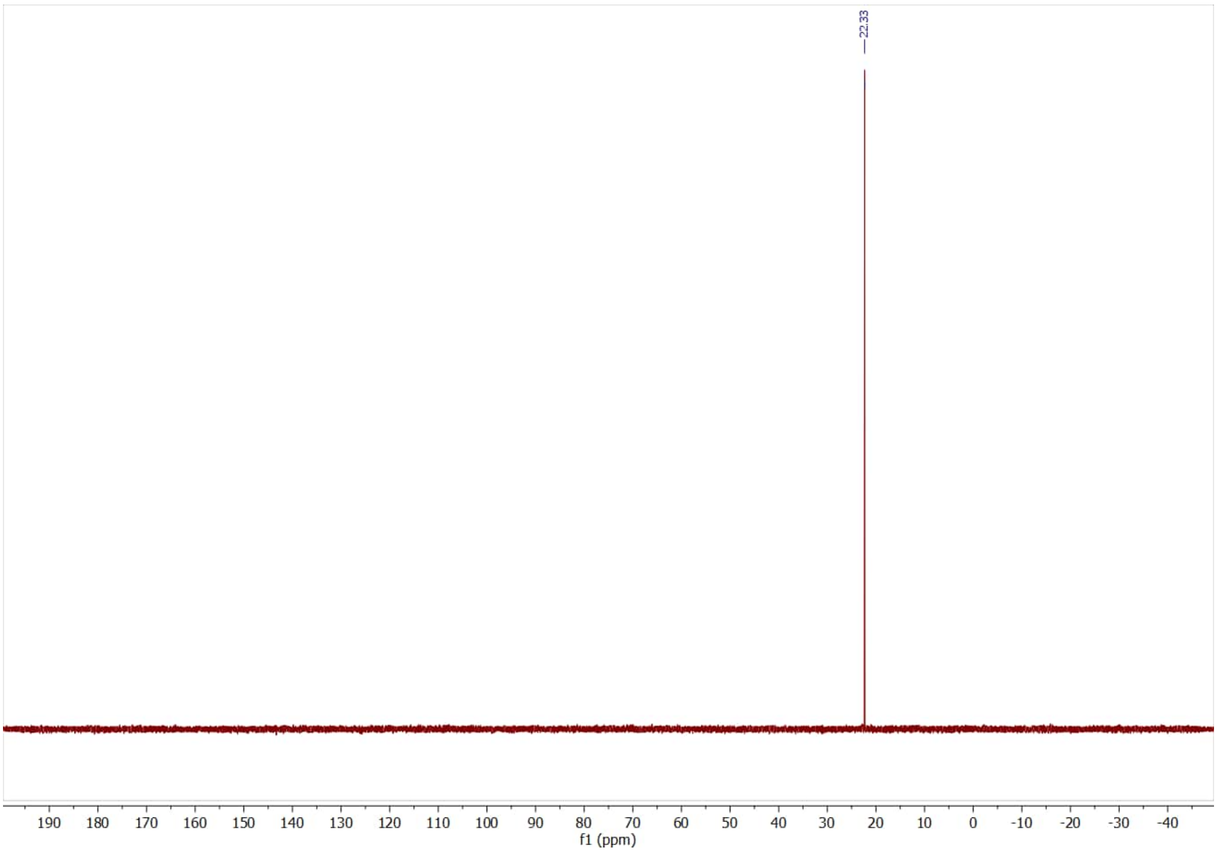

Figure S100. *^{31}P NMR spectrum of **4f** (400 MHz, CD_3OD).*

Diethyl ((2-methoxyphenyl)(4,5,6,7-tetrahydrobenzo[*c*]thiophene-1-carboxamido)-methyl)phosphonate (4g)

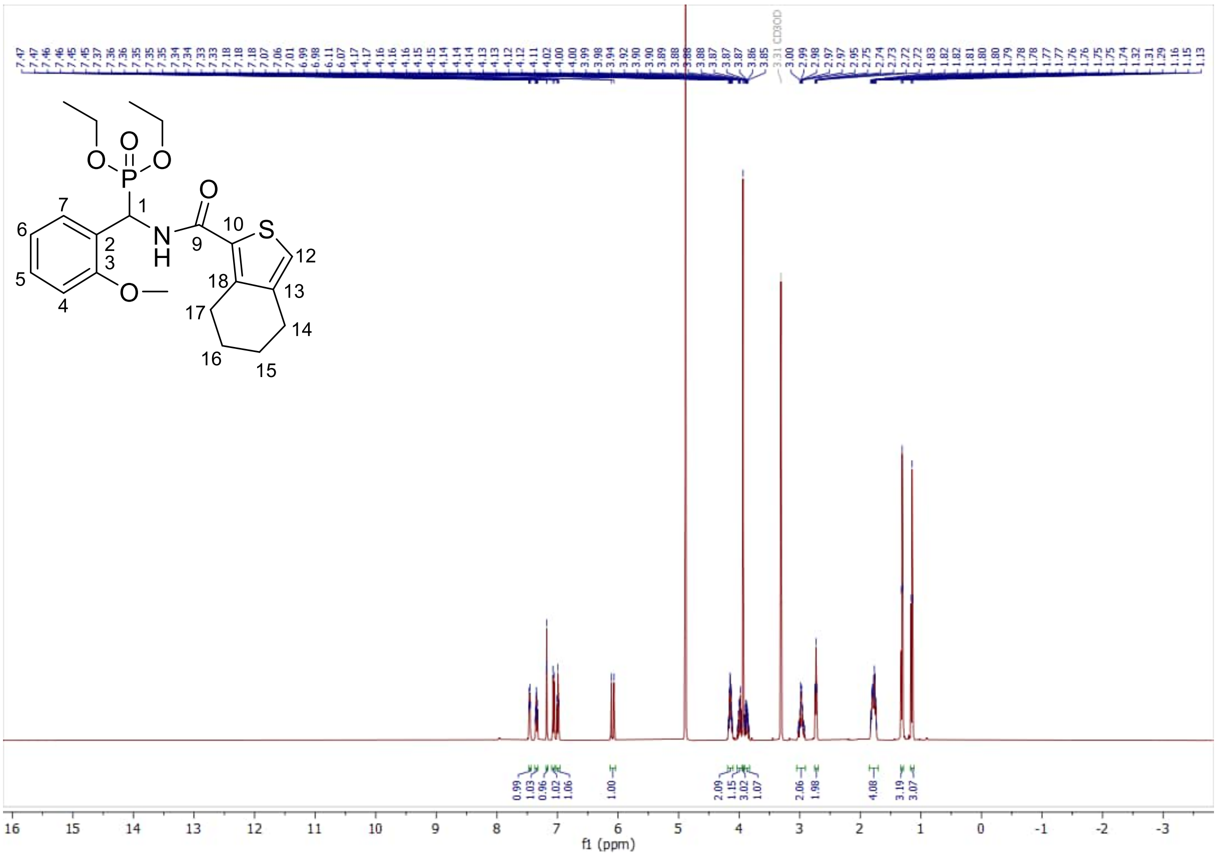

Figure S101. *^{1}H NMR spectrum of* ***4g*** *(500 MHz, CD_3OD).*

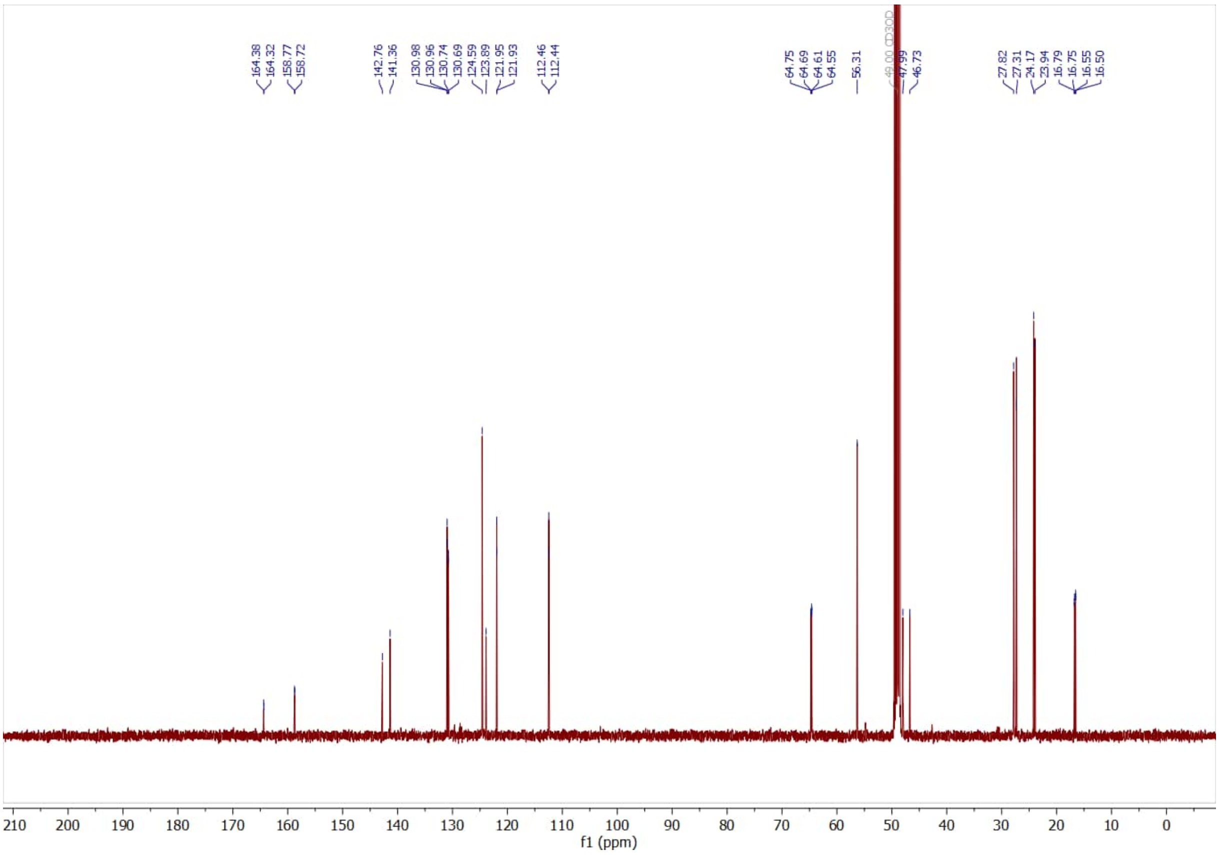

Figure S102. *^{13}C NMR spectrum of **4g** (500 MHz, CD_3OD).*

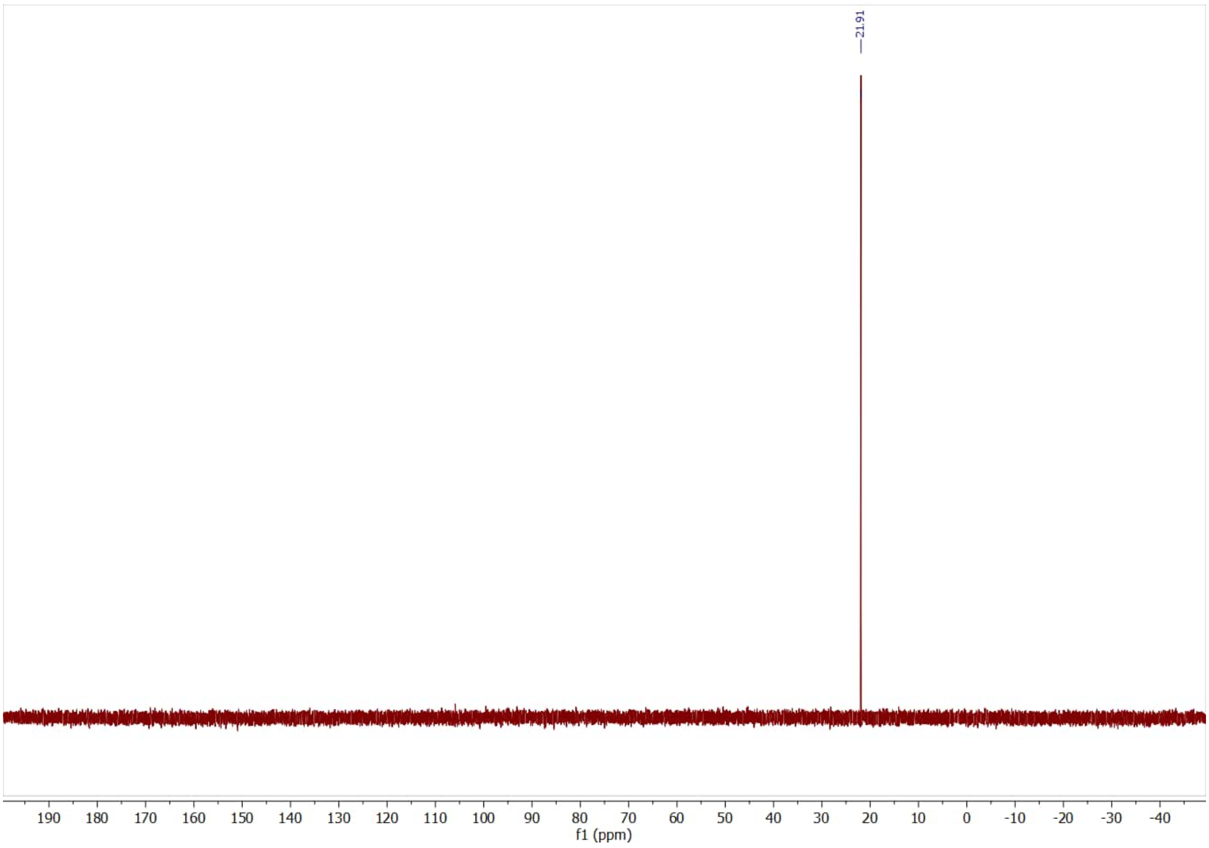

Figure S103. *^{31}P NMR spectrum of **4g** (400 MHz, CD_3OD).*

Diethyl (benzamido(2-methoxyphenyl)methyl)phosphonate (4h)

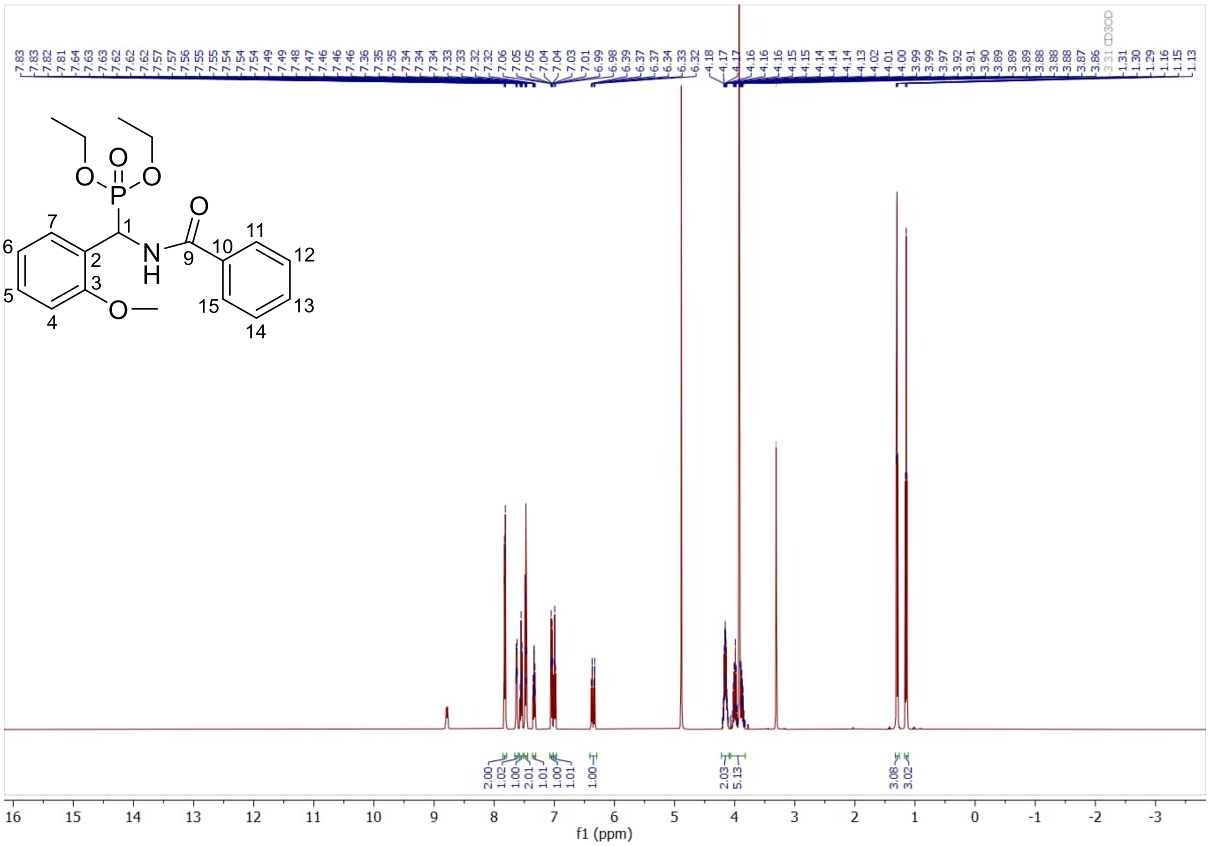

Figure S104. *¹H NMR spectrum of* ***4h*** *(500 MHz, CD_3OD).*

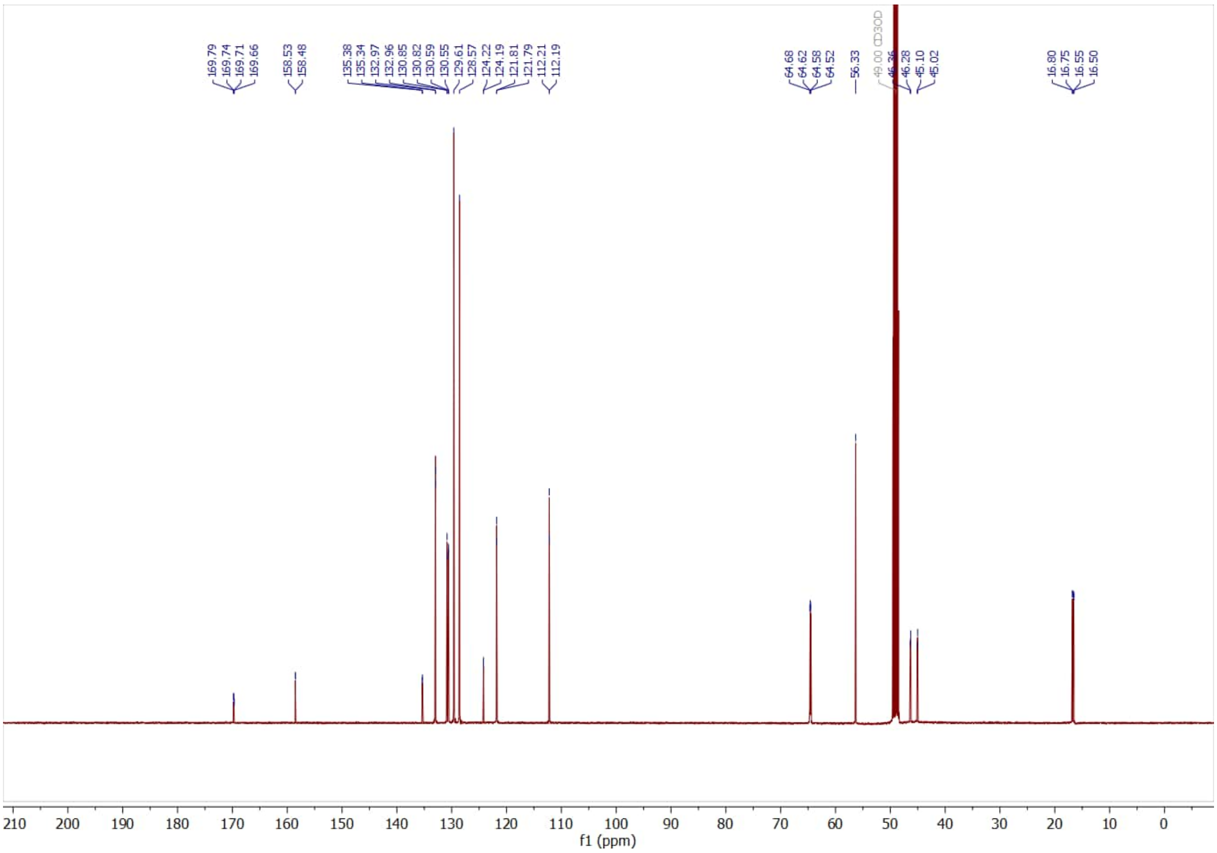

Figure S105. *^{13}C NMR spectrum of **4h** (500 MHz, CD_3OD).*

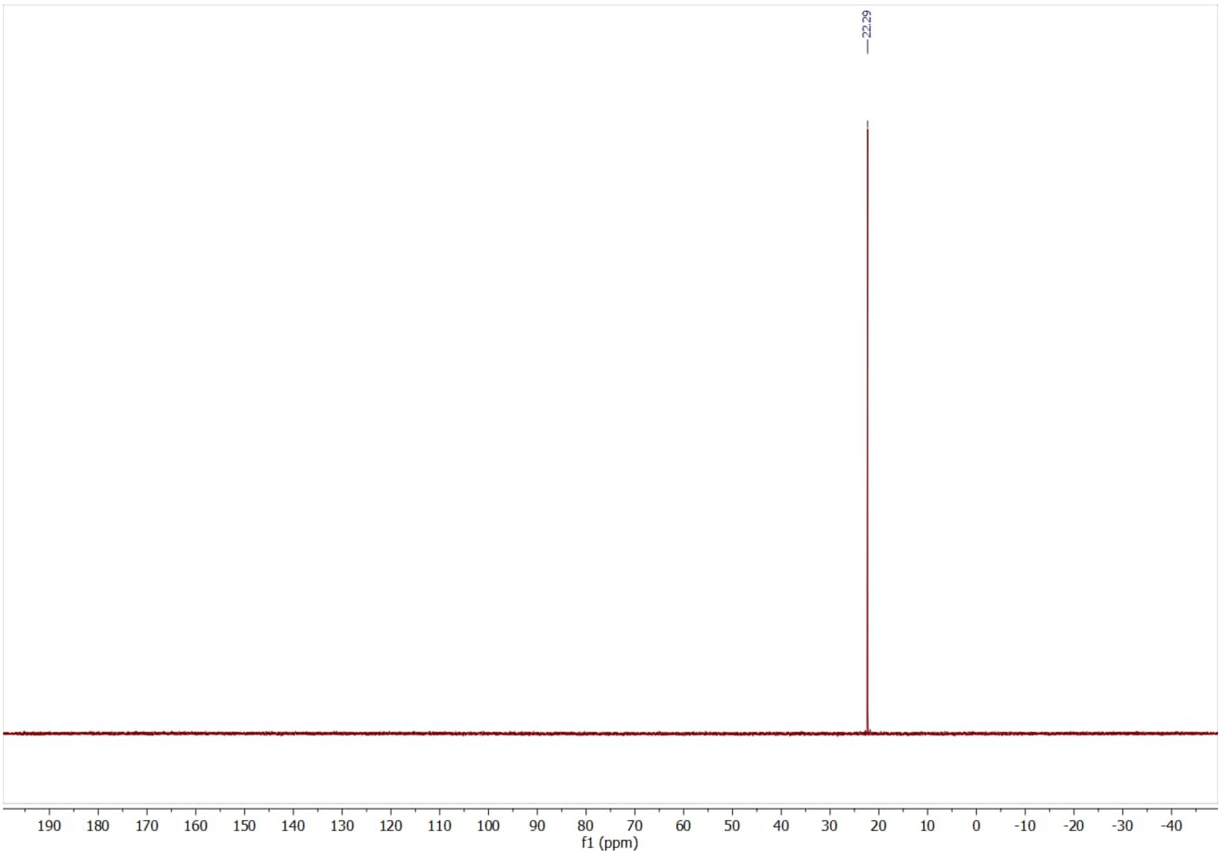

Figure S106. *^{31}P NMR spectrum of **4h** (400 MHz, CD_3OD).*

Diethyl ((2-methoxyphenyl)(2-(thiophen-2-yl)acetamido)methyl)phosphonate (4i)

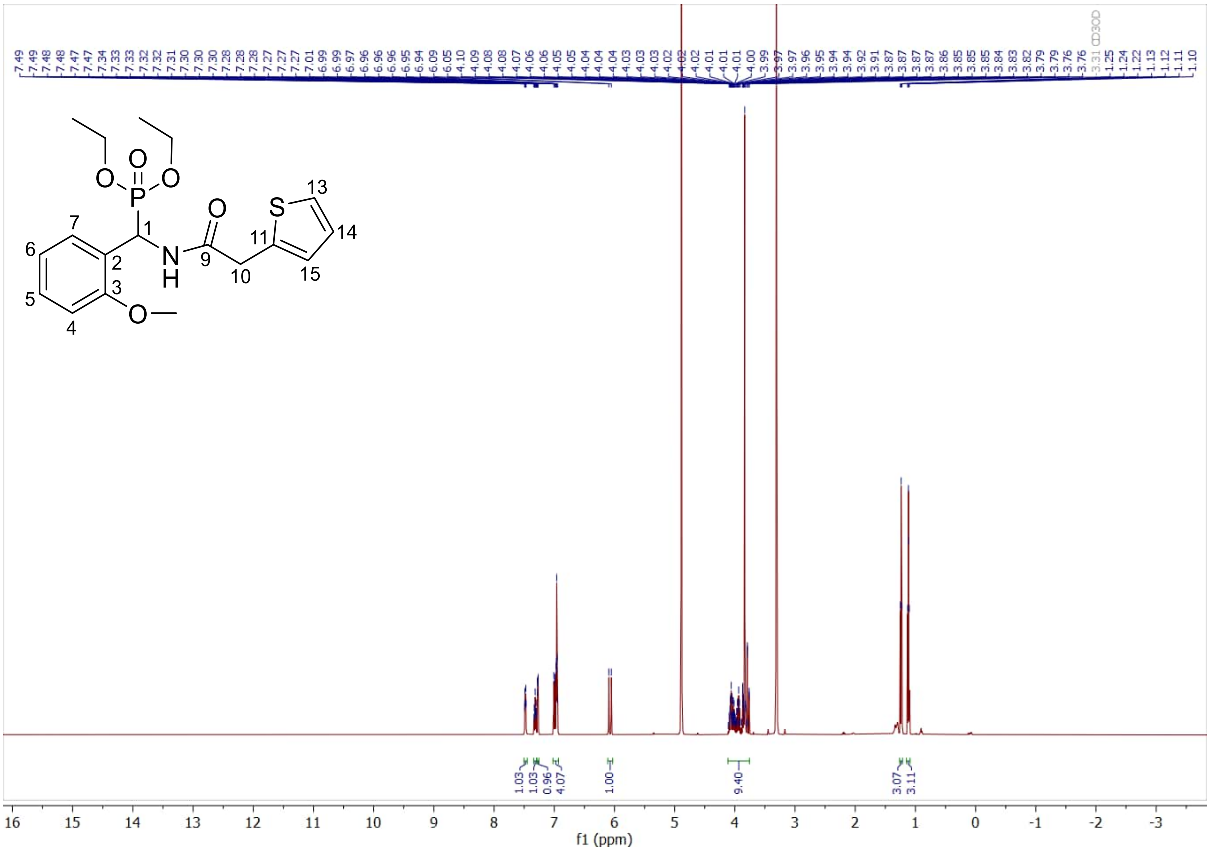

Figure S107. *^{1}H NMR spectrum of **4i** (500 MHz, CD_3OD).*

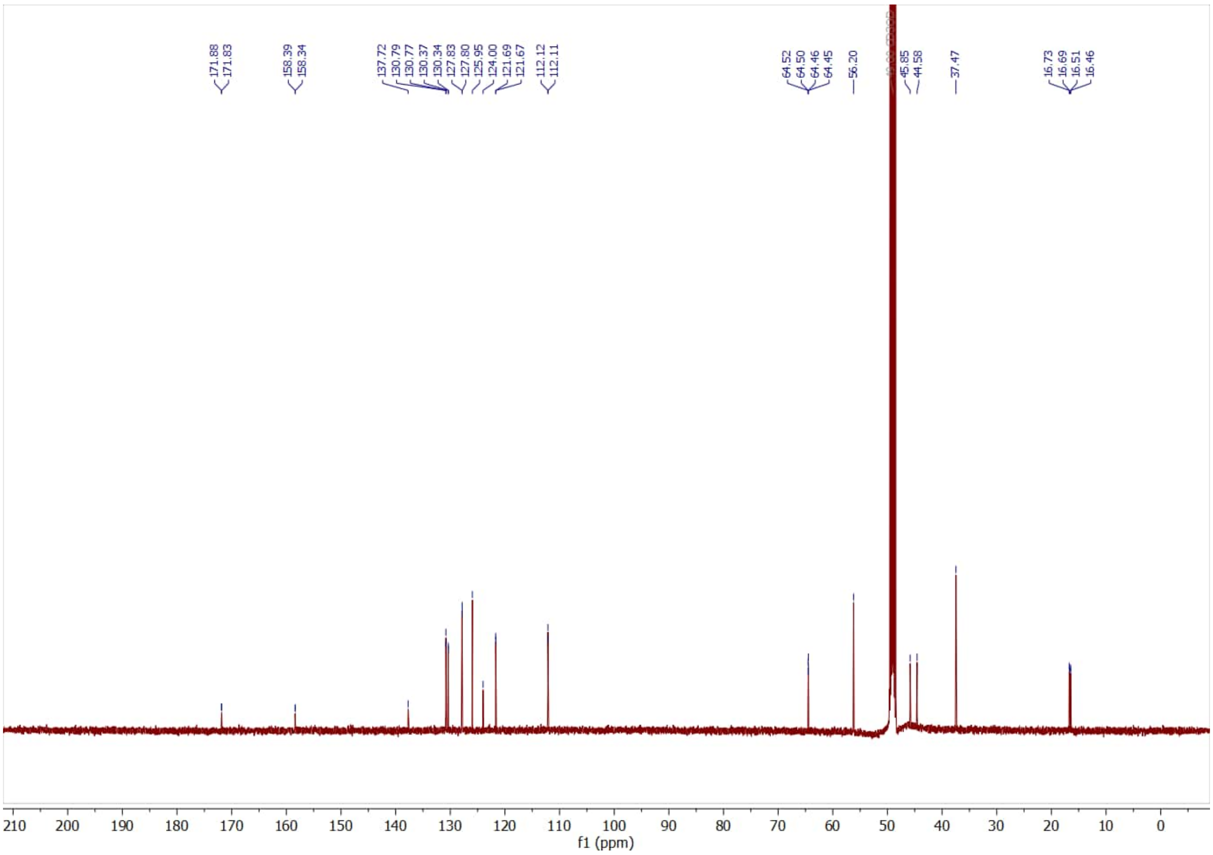

Figure S108. ^{13}C NMR spectrum of **4i** (500 MHz, CD_3OD).

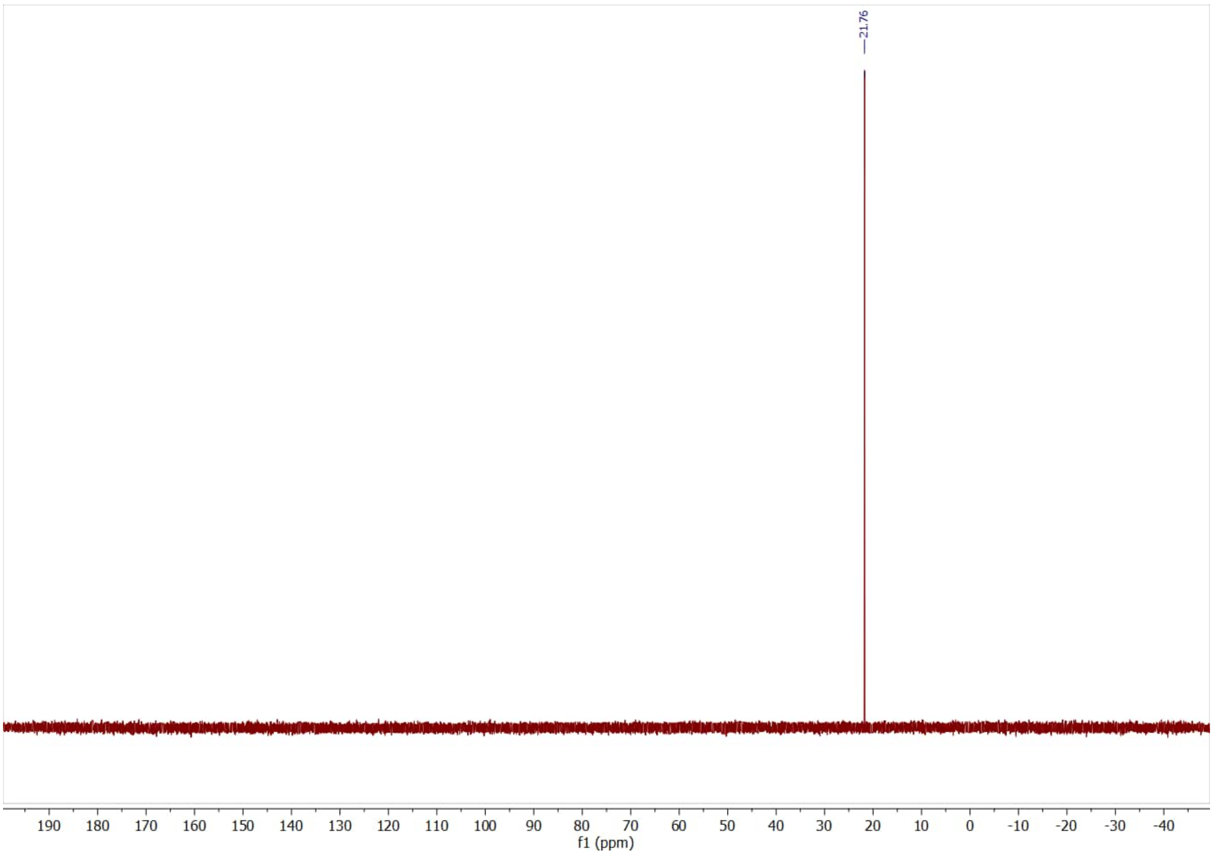

Figure S109. ^{31}P NMR spectrum of **4i** (400 MHz, CD_3OD).

Diethyl ((2-methoxyphenyl)(2-(thiophen-3-yl)acetamido)methyl)phosphonate (4j)

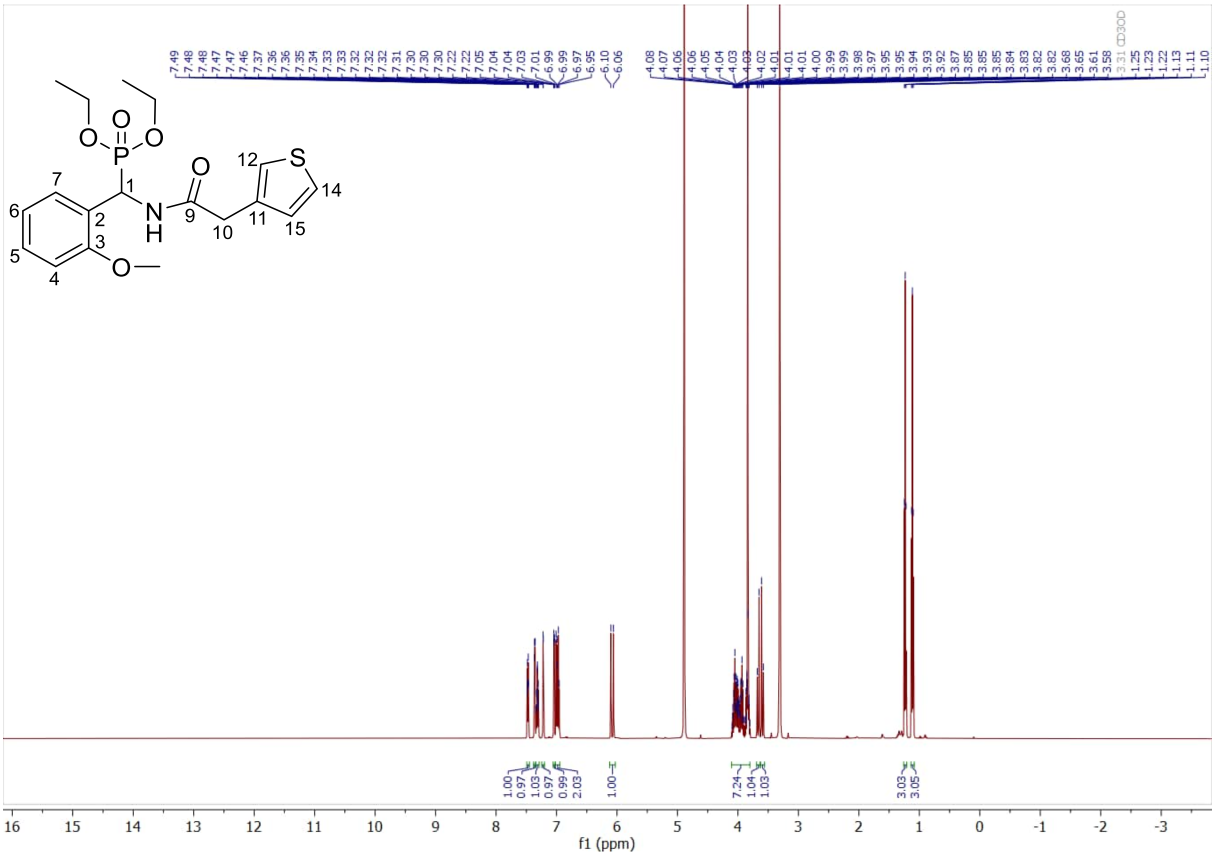

Figure S110. *^{1}H NMR spectrum of* ***4j*** *(500 MHz, CD_3OD).*

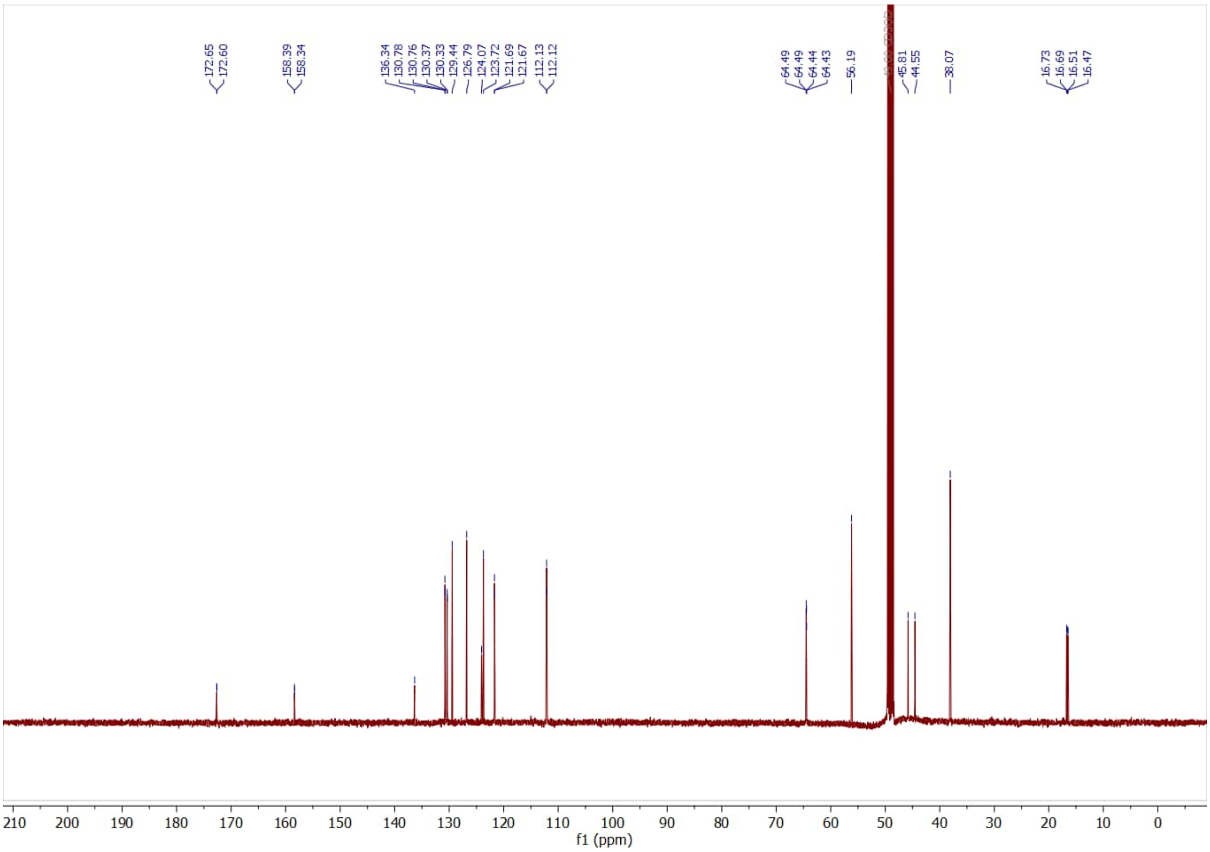

Figure S111. *^{13}C NMR spectrum of **4j** (500 MHz, CD_3OD).*

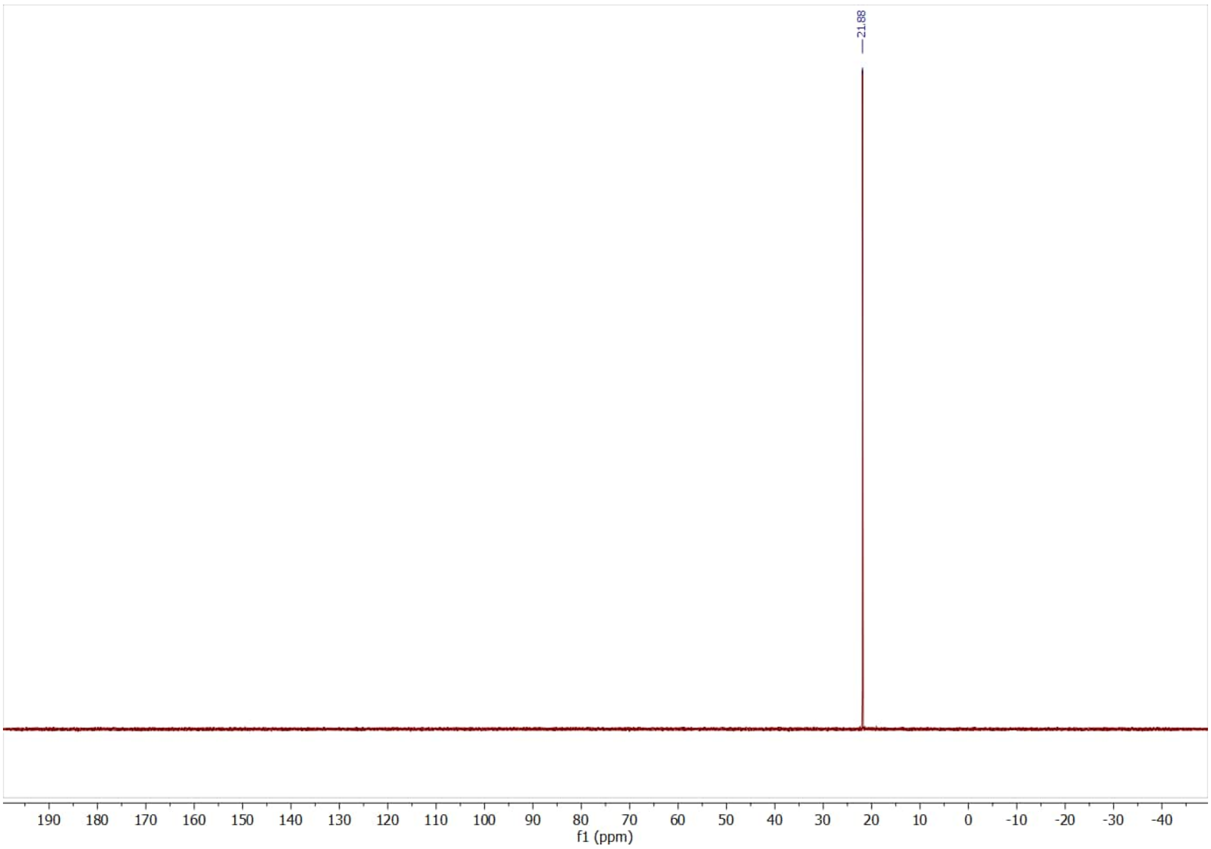

Figure S112. *^{31}P NMR spectrum of **4j** (400 MHz, CD_3OD).*

Diethyl ((2-(benzo[*b*]thiophen-2-yl)acetamido)(2-methoxyphenyl)methyl)phosphonate (4k)

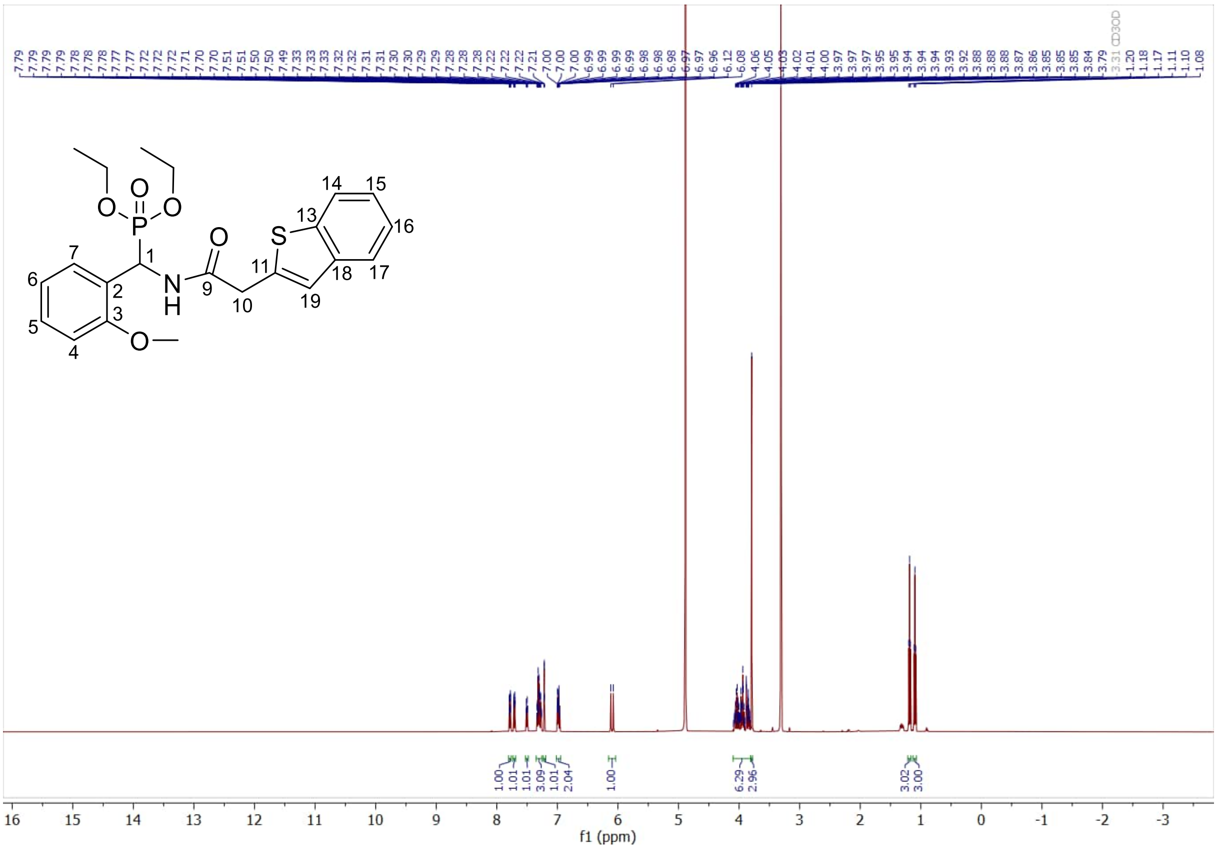

Figure S113. *^{1}H NMR spectrum of* ***4k*** *(500 MHz, CD_3OD).*

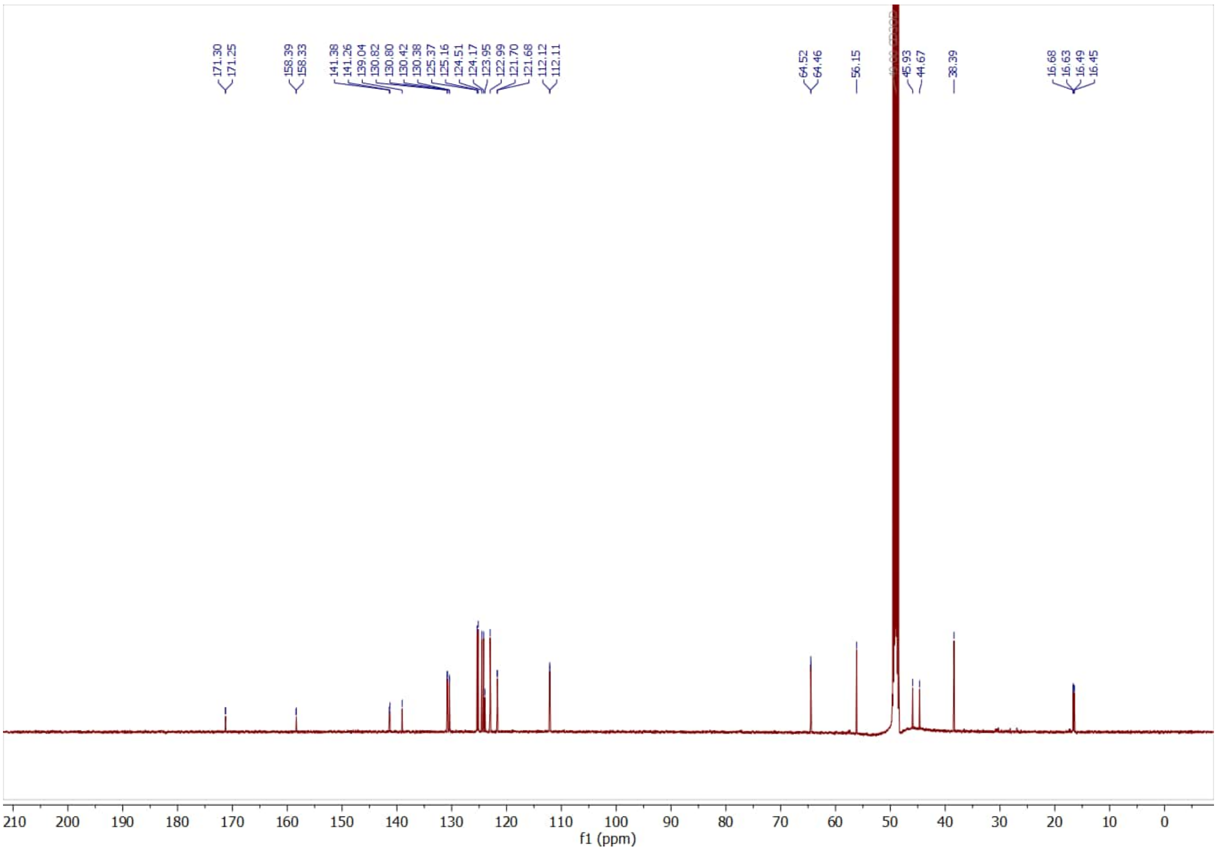

Figure S114. *^{13}C NMR spectrum of* ***4k*** *(500 MHz, CD_3OD).*

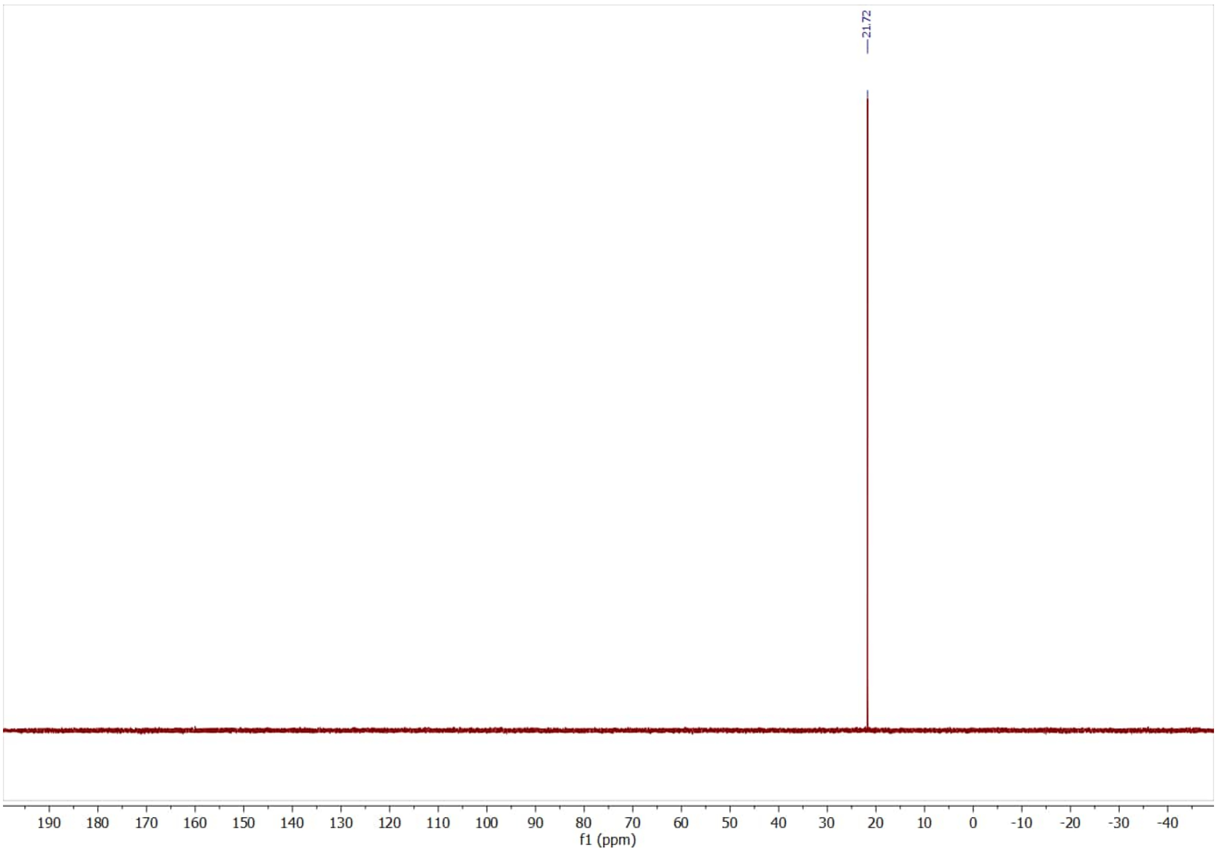

Figure S115. *^{31}P NMR spectrum of* ***4k*** *(400 MHz, CD_3OD).*

Diethyl ((2-(benzo[*b*]thiophen-3-yl)acetamido)(2-methoxyphenyl)methyl)phosphonate (4l)

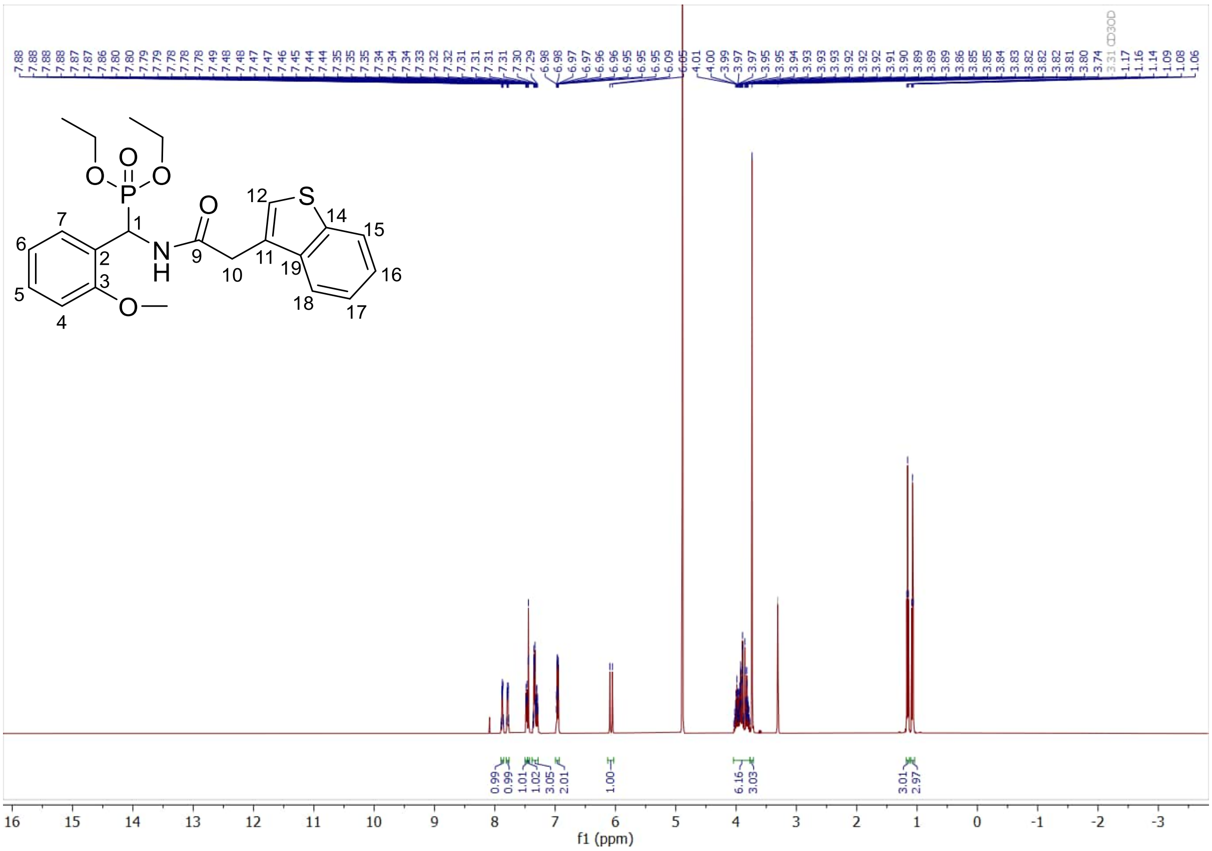

Figure S116. *^{1}H NMR spectrum of **4l** (500 MHz, CD_3OD).*

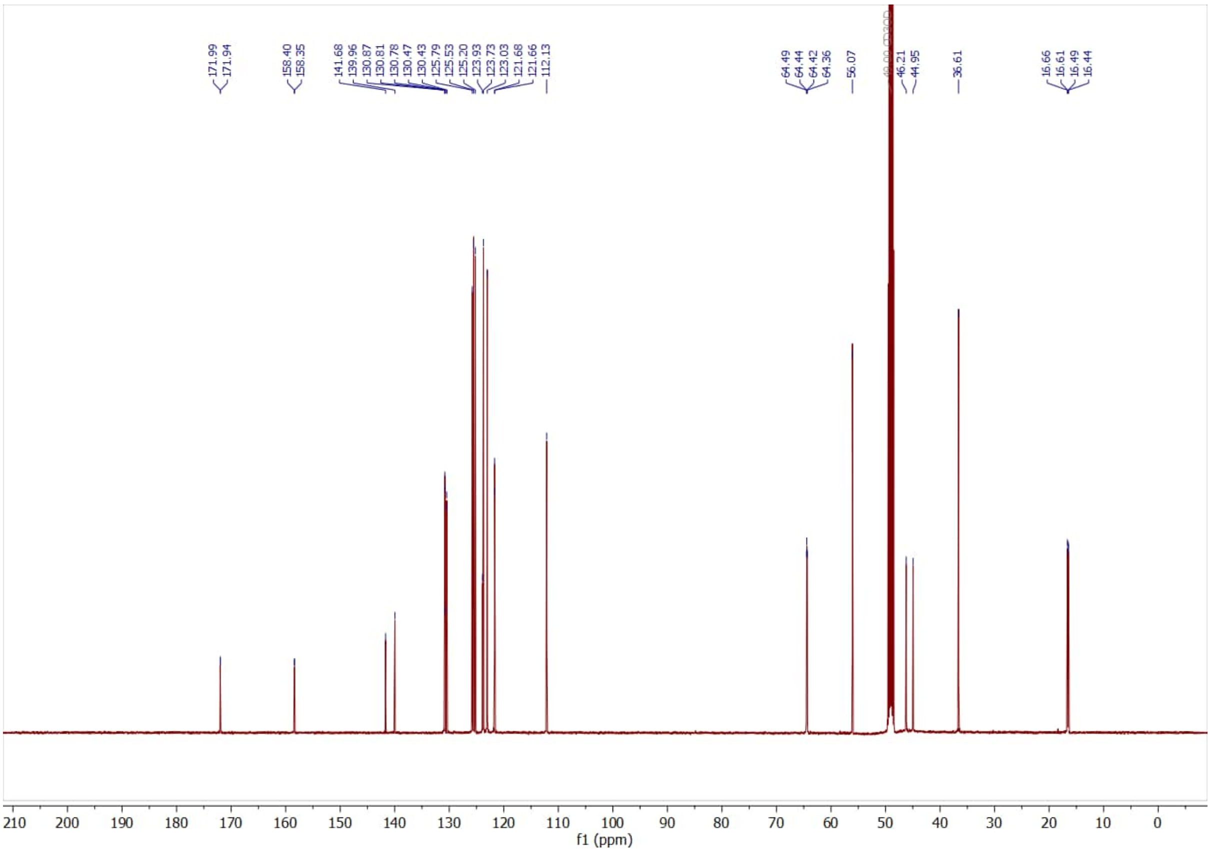

Figure S117. *^{13}C NMR spectrum of **4l** (500 MHz, CD_3OD).*

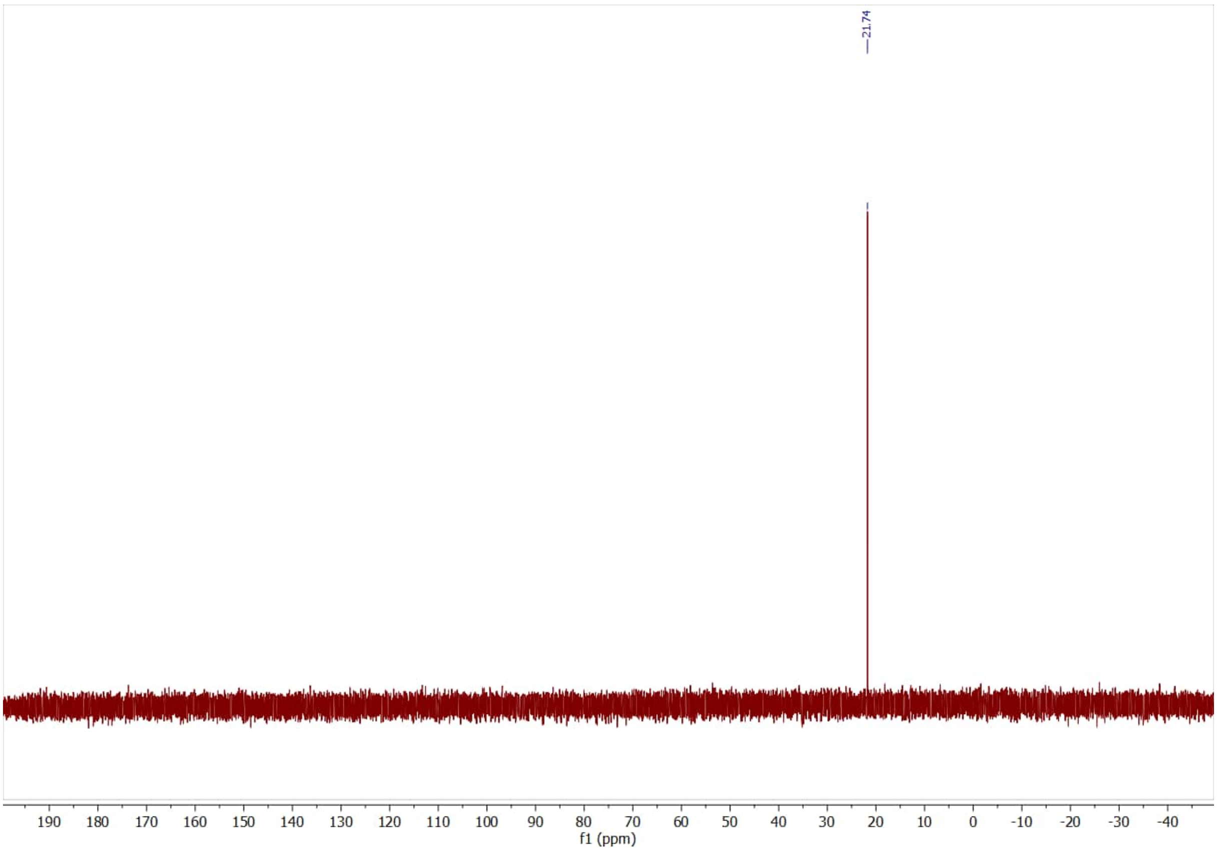

Figure S118. *^{31}P NMR spectrum of **4l** (400 MHz, CD_3OD).*

Diethyl ((2-methoxyphenyl)(2-phenylacetamido)methyl)phosphonate (4m)

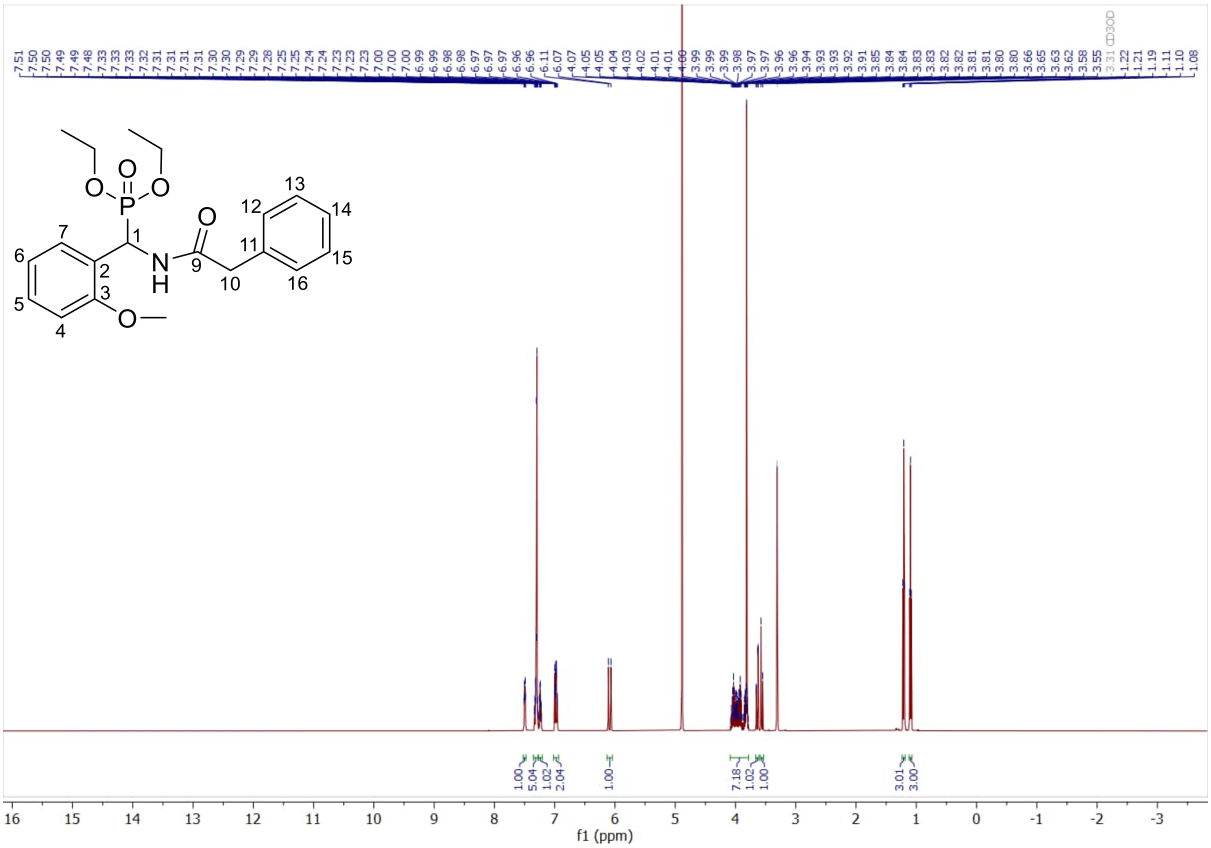

Figure S119. *^{1}H NMR spectrum of* ***4m*** *(500 MHz, CD_3OD).*

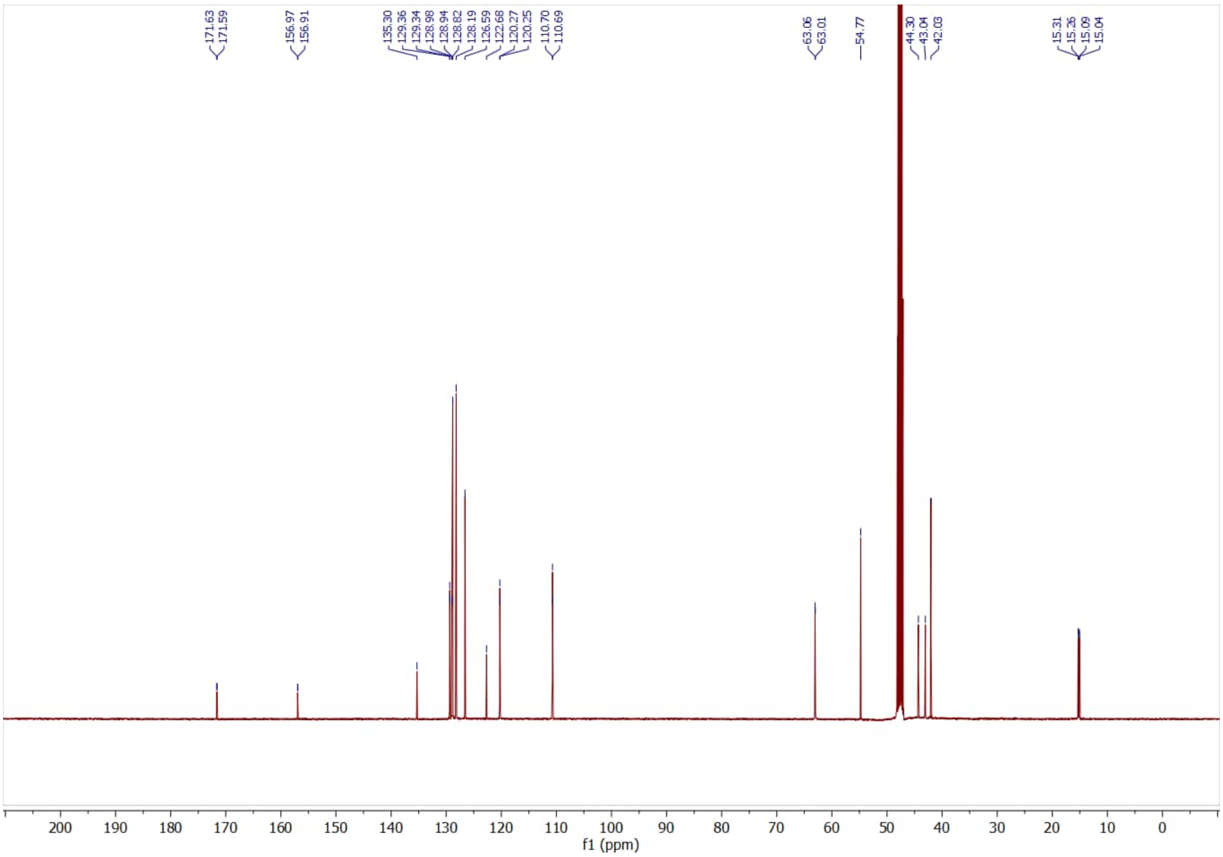

Figure S120. ^{13}C NMR spectrum of **4m** (500 MHz, CD_3OD).

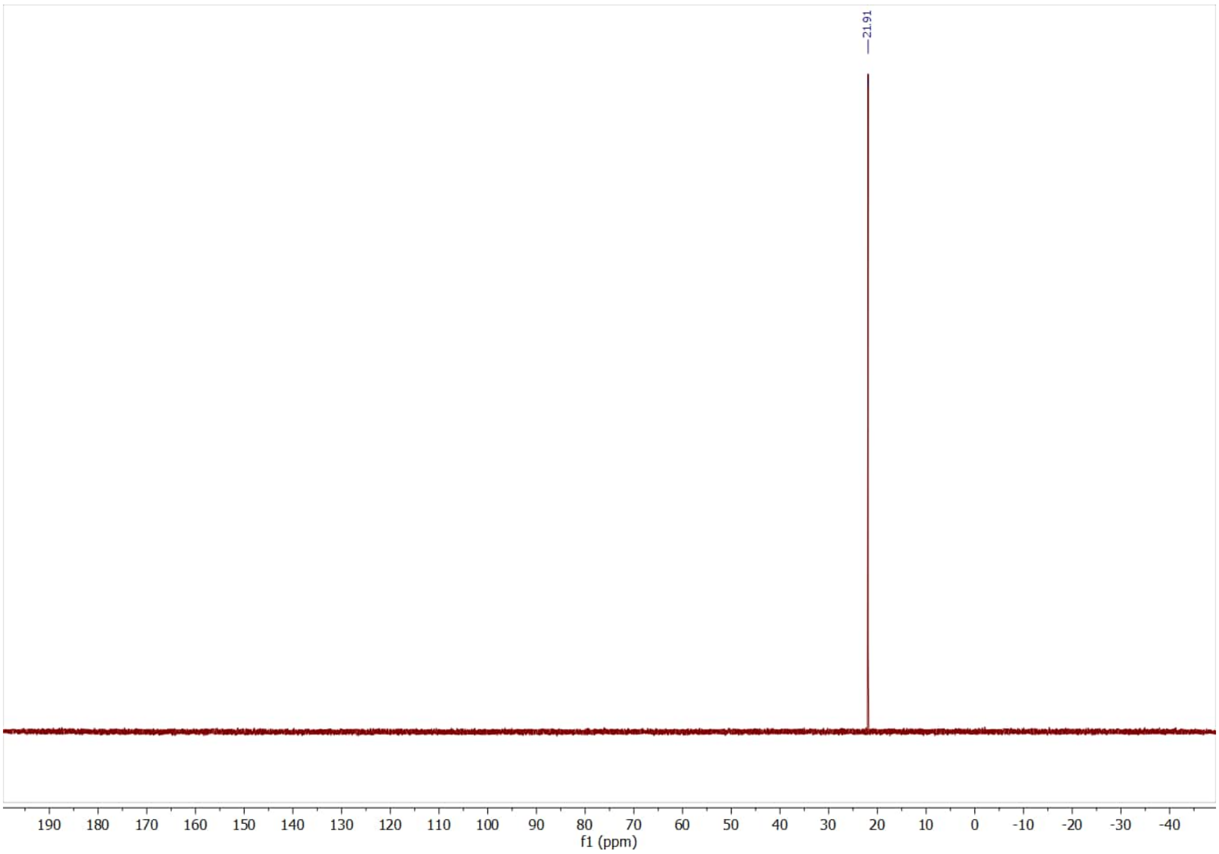

Figure S121. ^{31}P NMR spectrum of **4m** (400 MHz, CD_3OD).

((2-Hydroxyphenyl)(thiophene-2-carboxamido)methyl)phosphonic acid (5a)

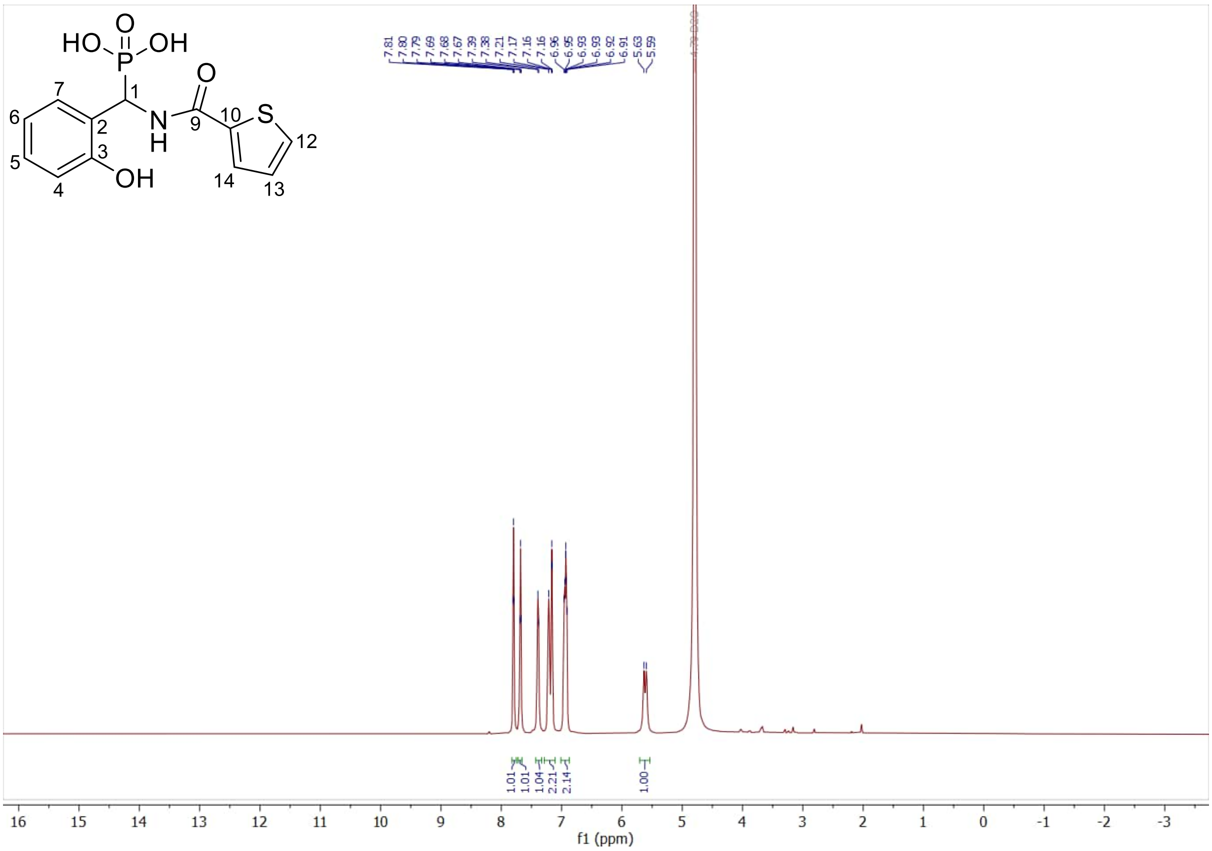

Figure S122. *^{1}H NMR spectrum of* ***5a*** *(500 MHz, D_2O).*

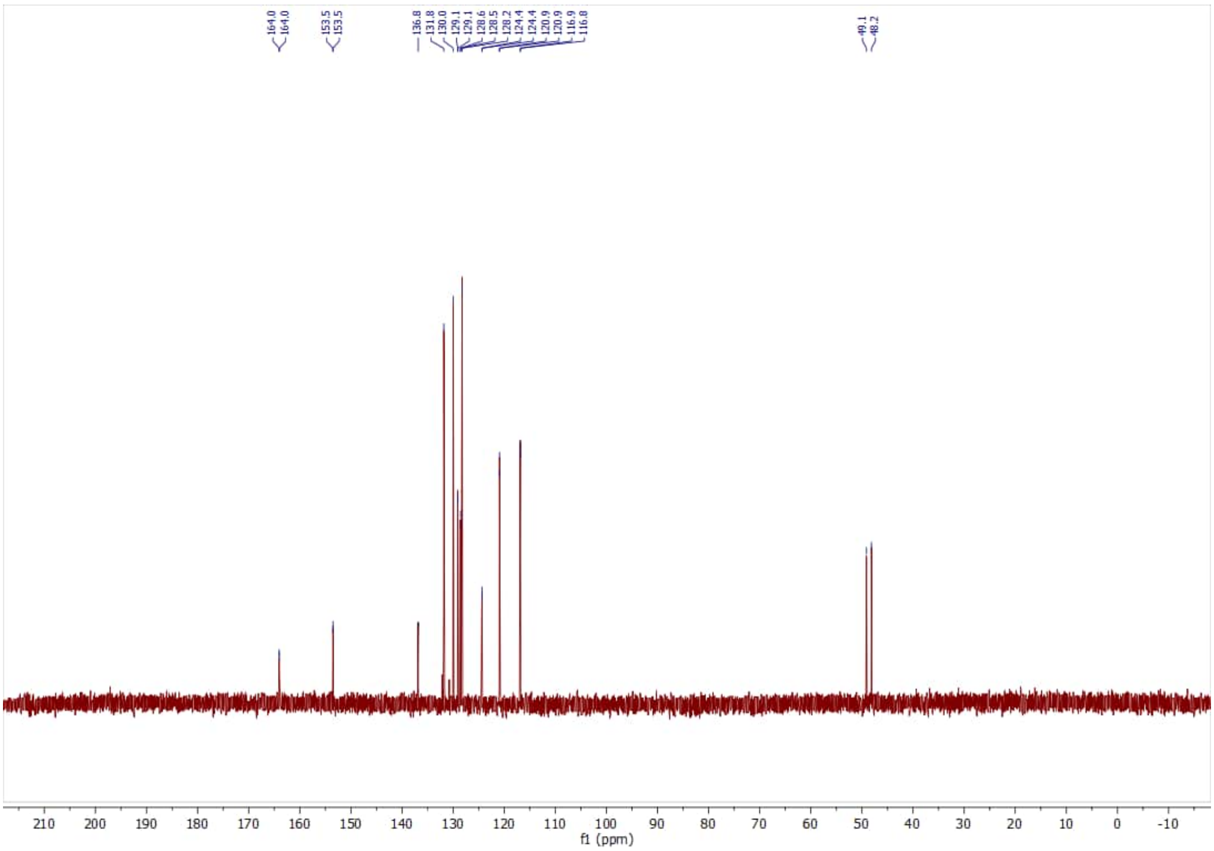

Figure S123. ^{13}C NMR spectrum of **5a** (600 MHz, D_2O).

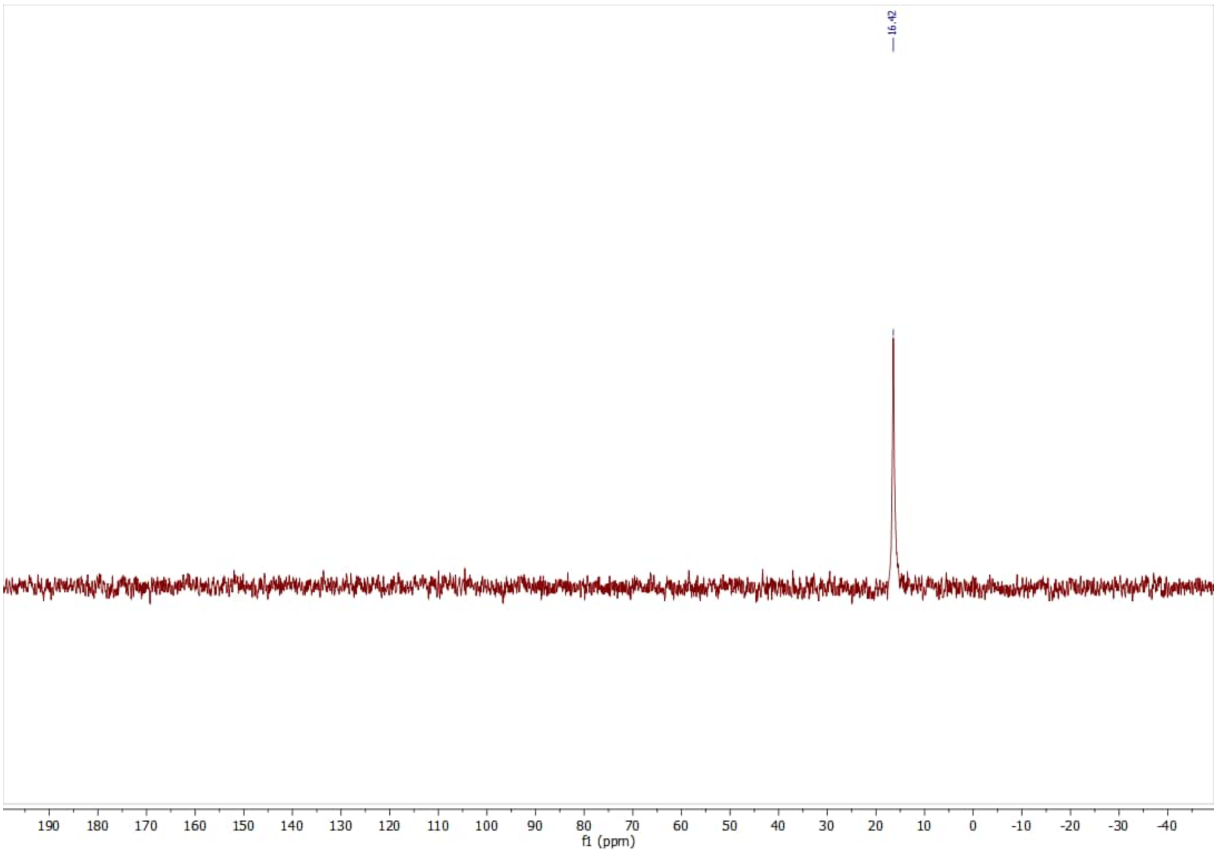

Figure S124. ^{31}P NMR spectrum of **5a** (400 MHz, D_2O).

((2-Hydroxyphenyl)(thiophene-3-carboxamido)methyl)phosphonic acid (5b)

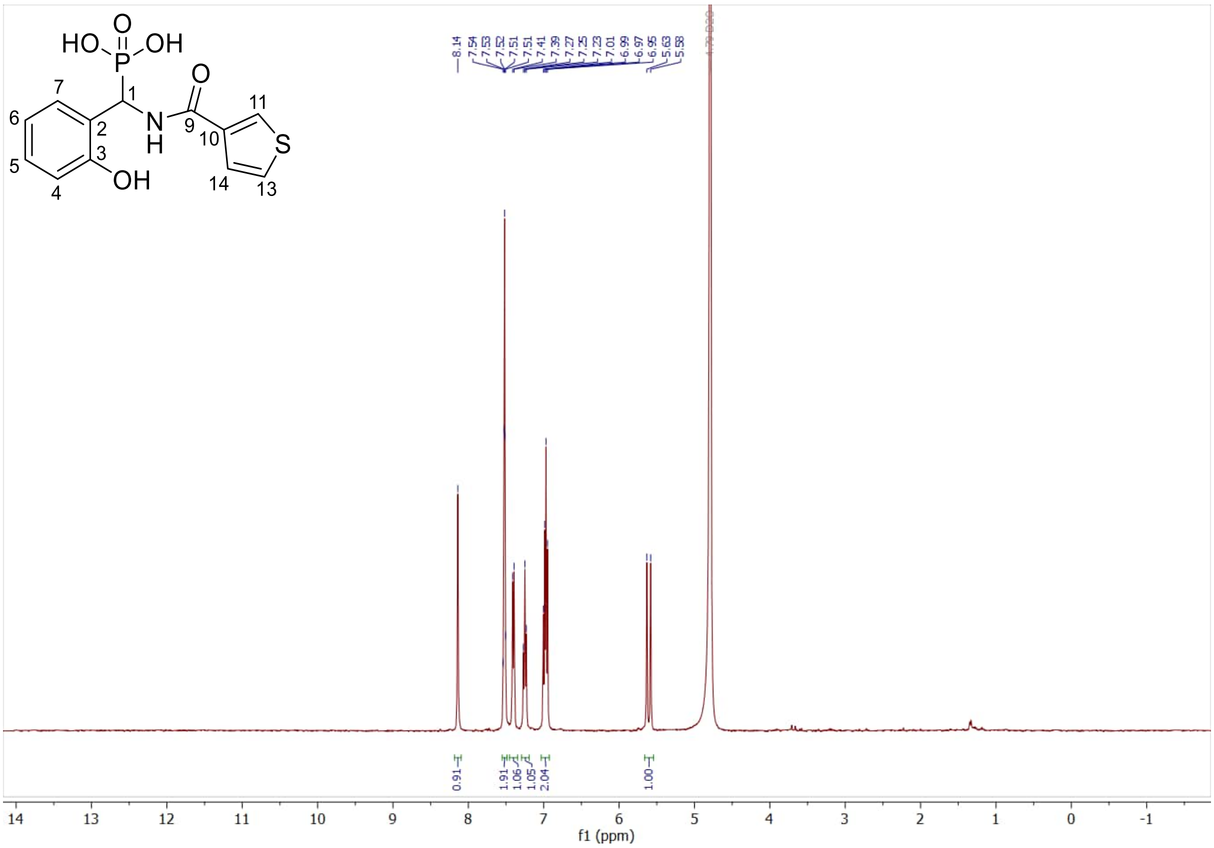

Figure S125. *^{1}H NMR spectrum of* ***5b*** *(400 MHz, D_2O).*

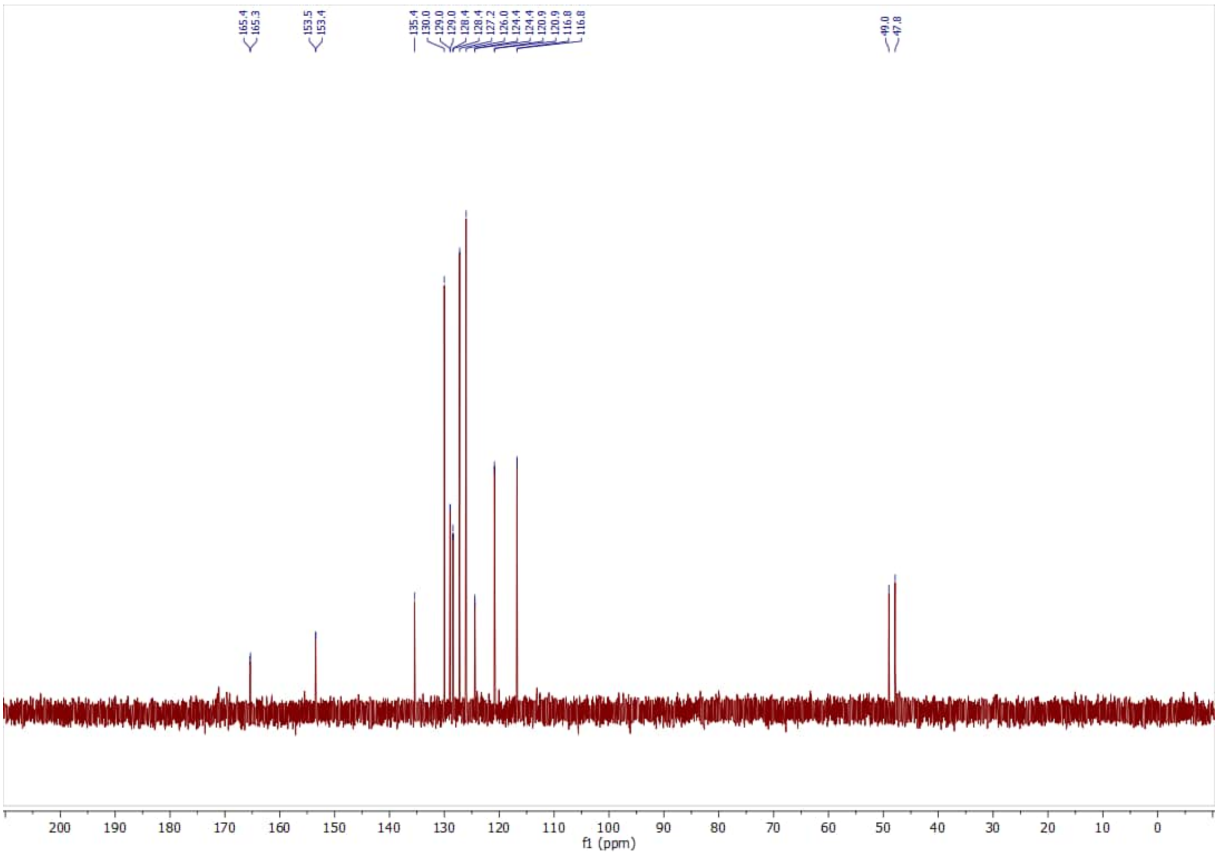

Figure S126. *^{13}C NMR spectrum of* ***5b*** *(500 MHz, D_2O).*

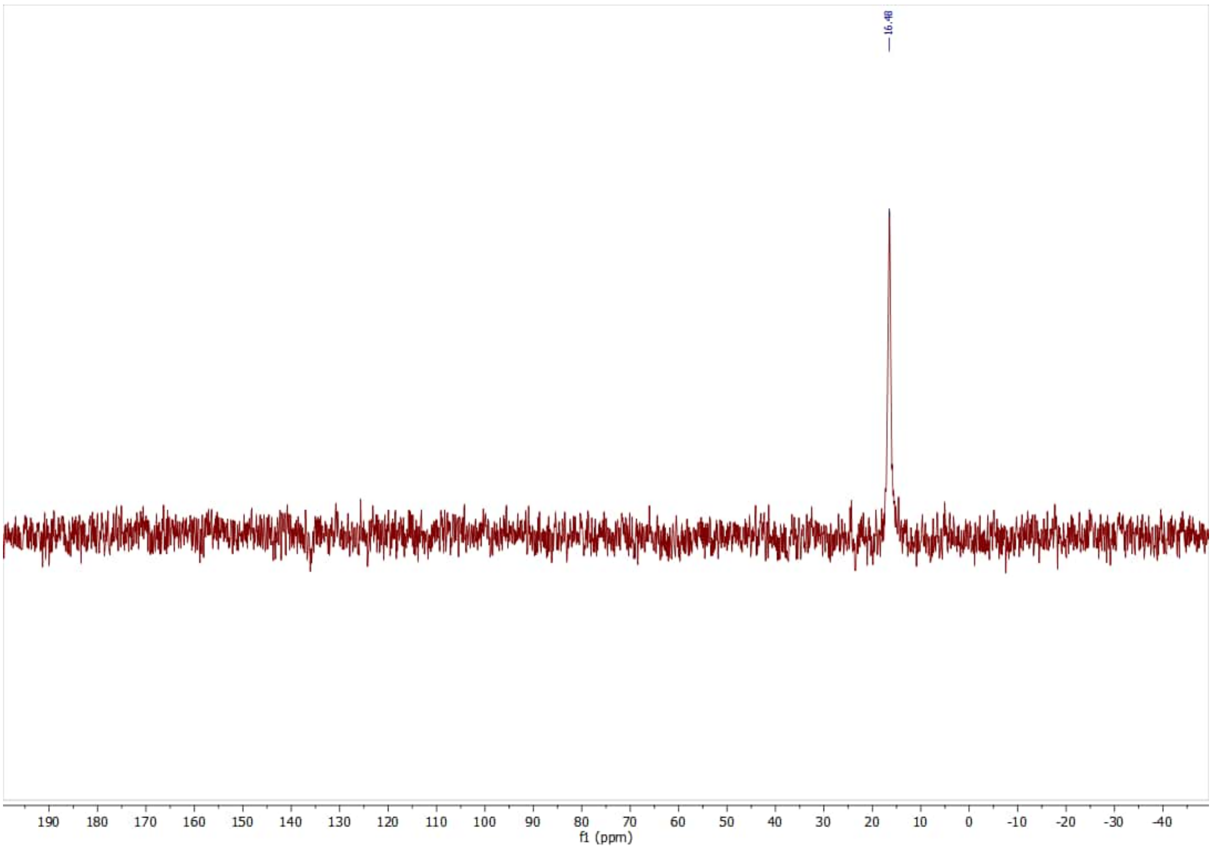

Figure S127. *^{31}P NMR spectrum of* ***5b*** *(400 MHz, D_2O).*

((Benzo[*b*]thiophene-2-carboxamido)(2-hydroxyphenyl)methyl)phosphonic acid (5c)

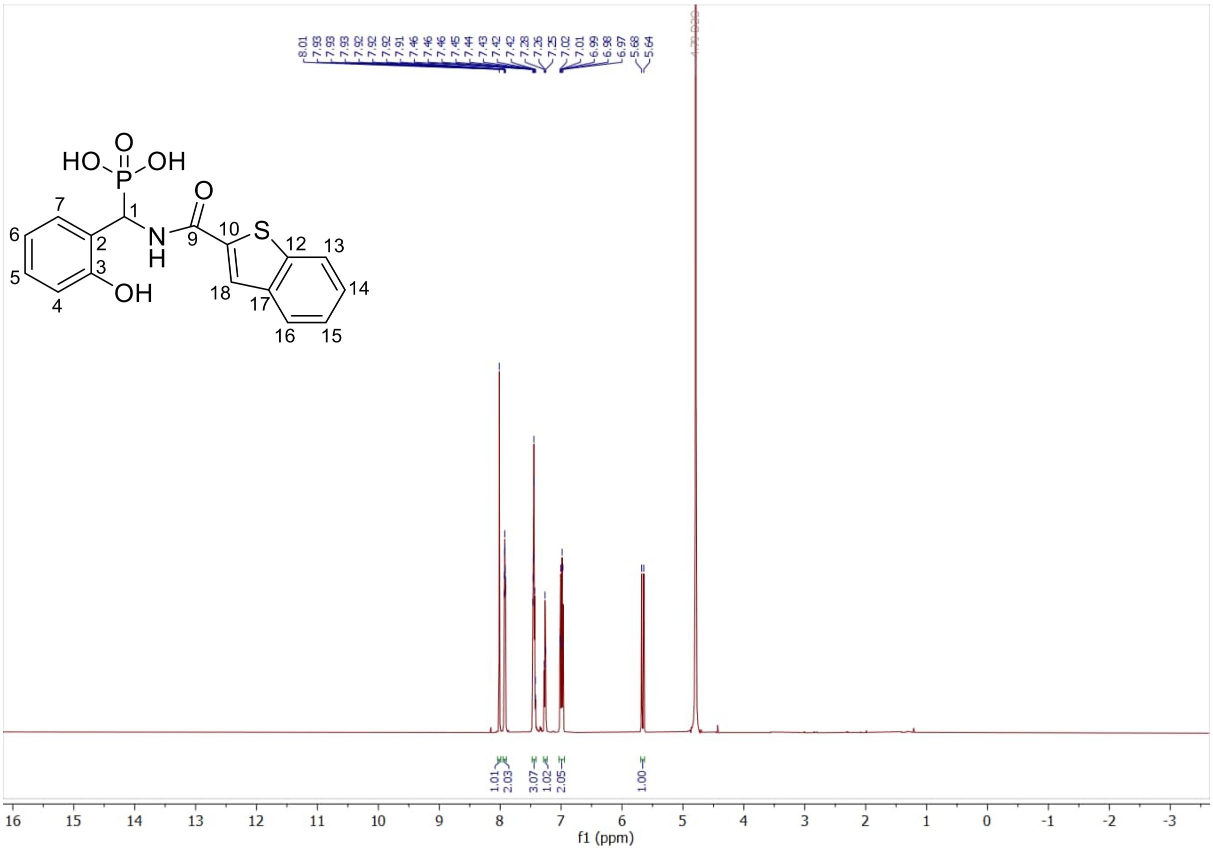

Figure S128. *^{1}H NMR spectrum of* ***5c*** *(600 MHz, D_2O).*

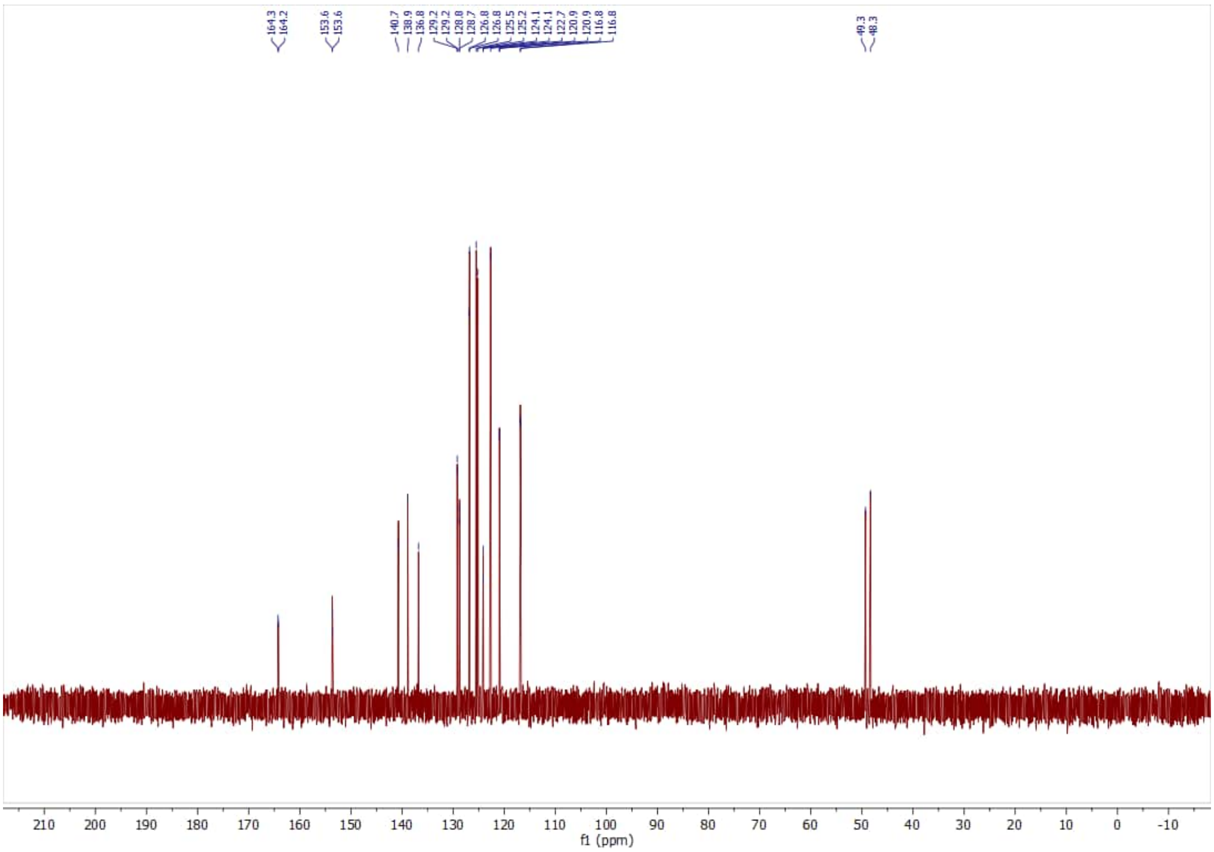

Figure S129. *^{13}C NMR spectrum of **5c** (600 MHz, D_2O).*

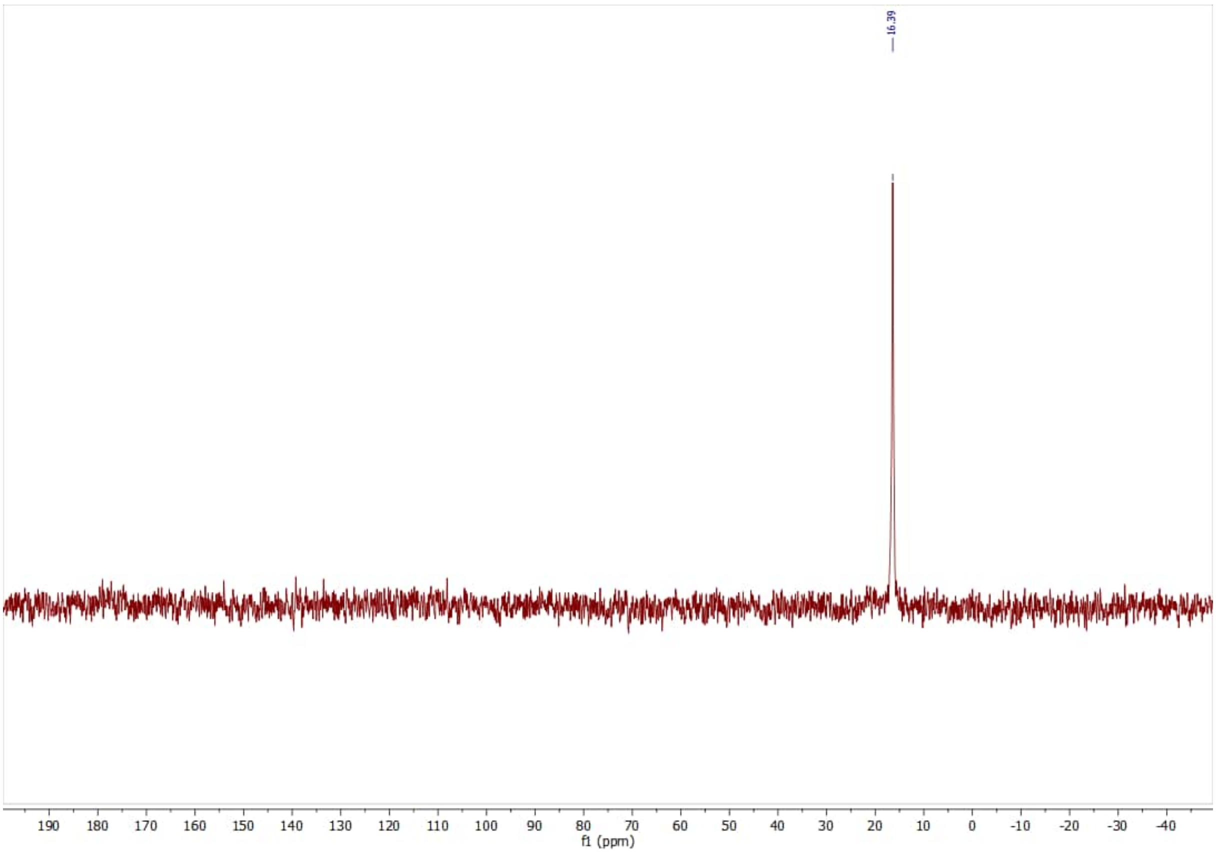

Figure S130. *^{31}P NMR spectrum of **5c** (400 MHz, D_2O).*

((Benzo[*b*]thiophene-3-carboxamido)(2-hydroxyphenyl)methyl)phosphonic acid (5d)

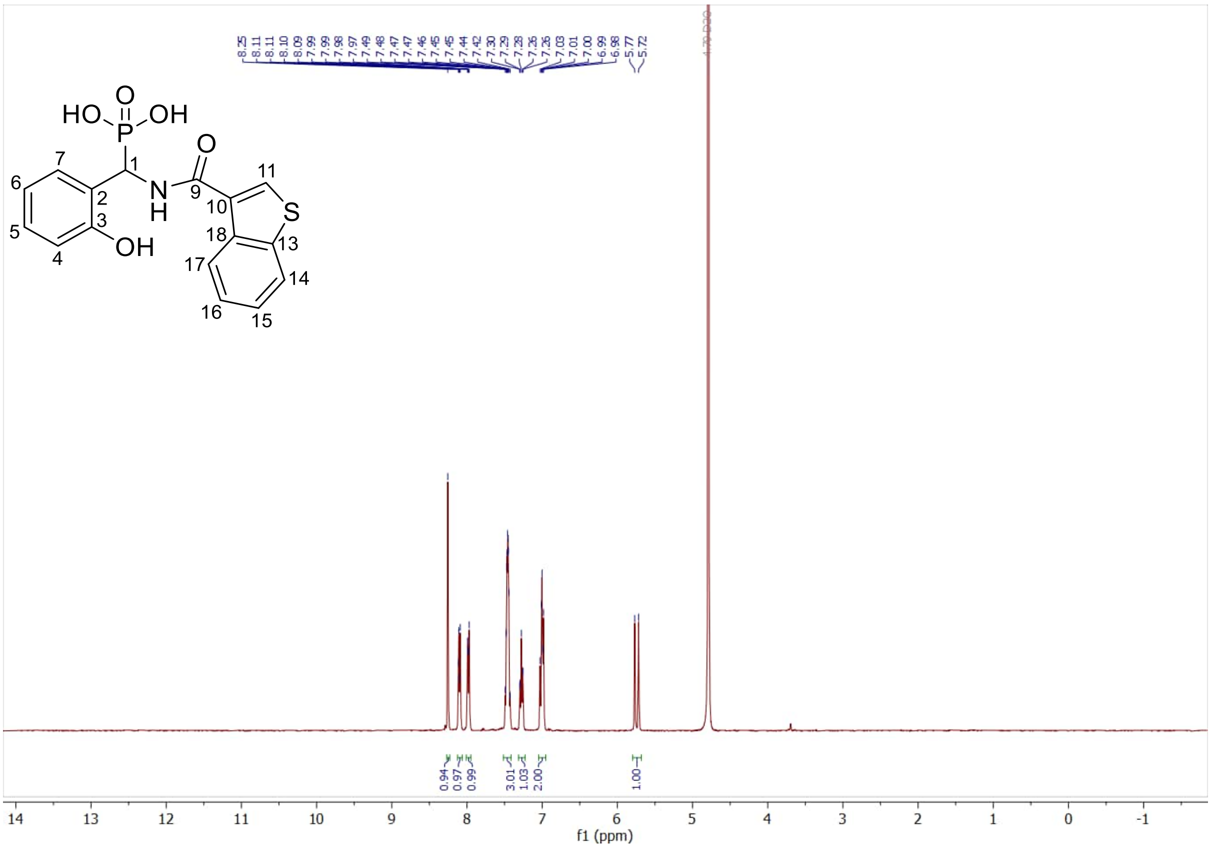

Figure S131. *^{1}H NMR spectrum of* ***5d*** *(400 MHz, D_2O).*

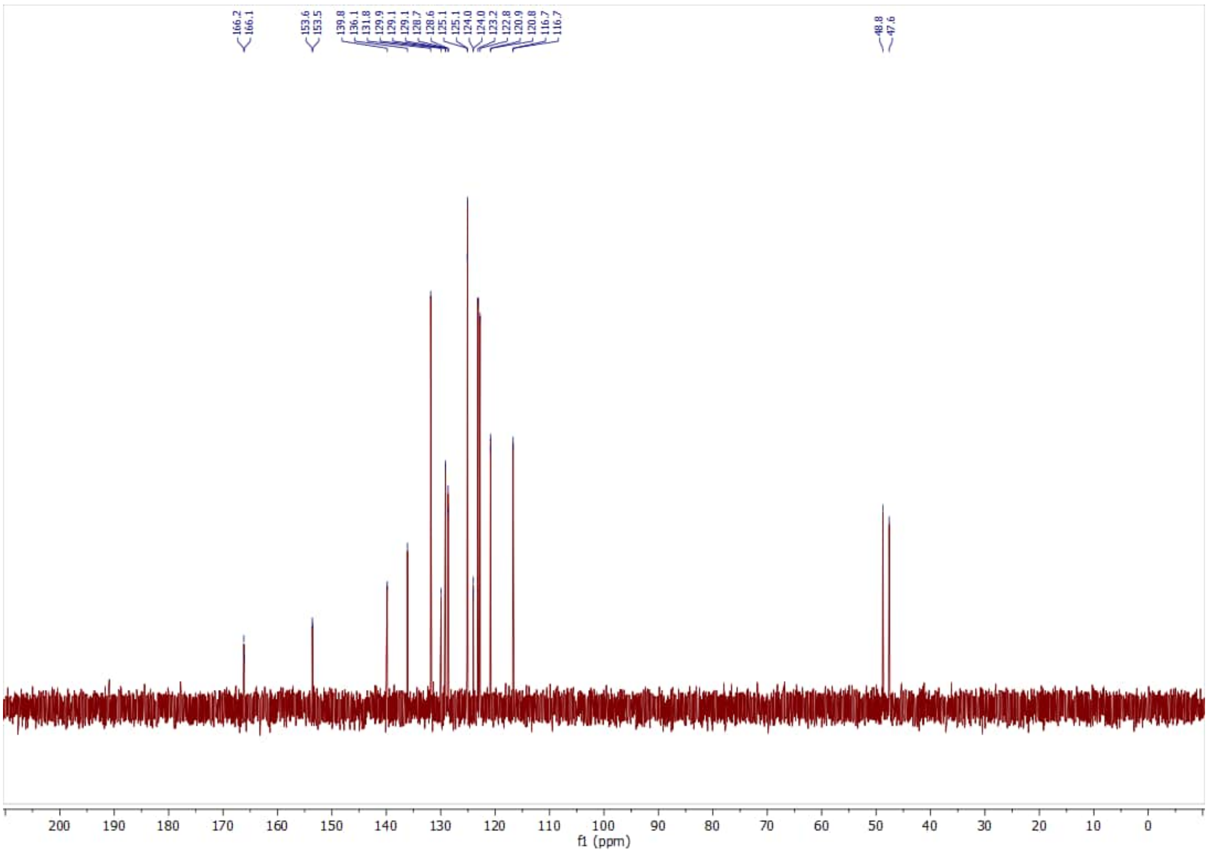

Figure S132. [13]*C NMR spectrum of* ***5d*** *(500 MHz,* D_2O*).*

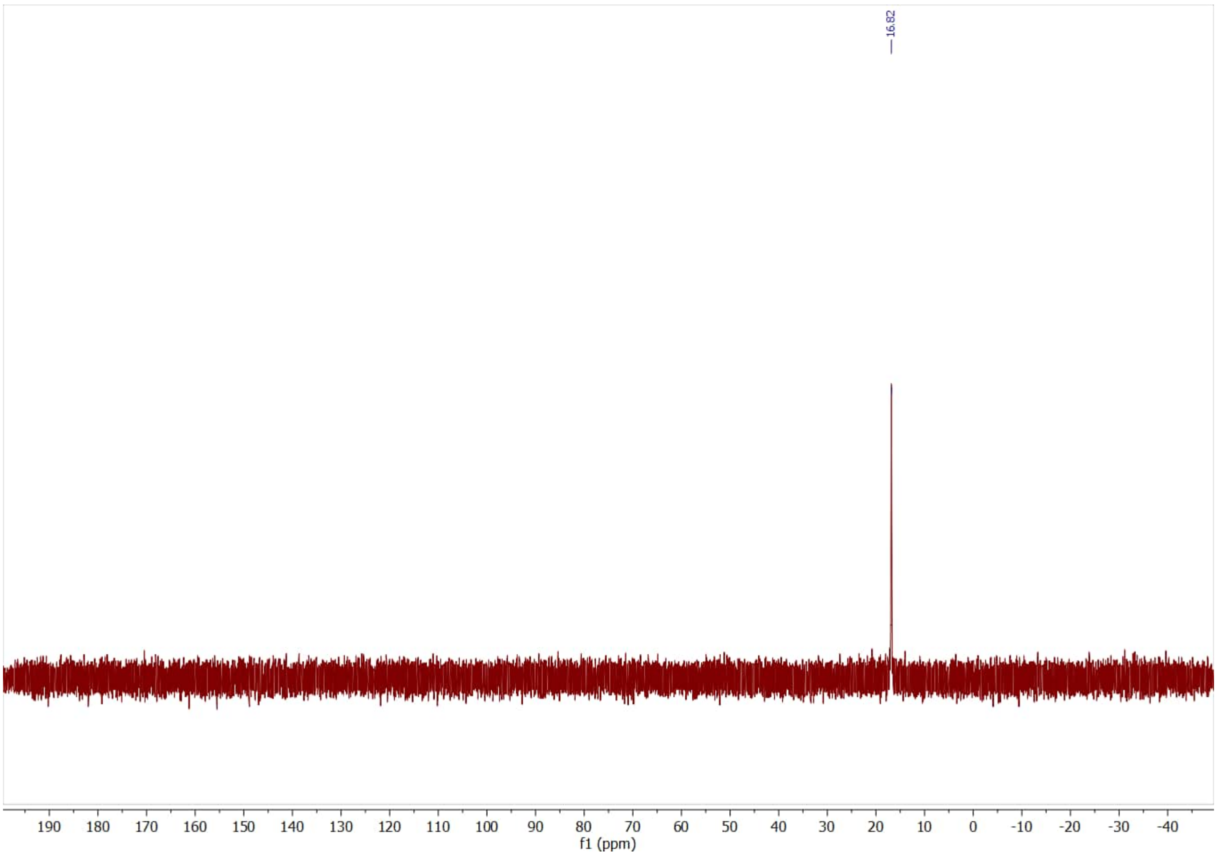

Figure S133. [31]*P NMR spectrum of* ***5d*** *(400 MHz,* D_2O*).*

((2-Hydroxyphenyl)(4,5,6,7-tetrahydrobenzo[*b*]thiophene-2-carboxamido)methyl)phosphonic acid (5e)

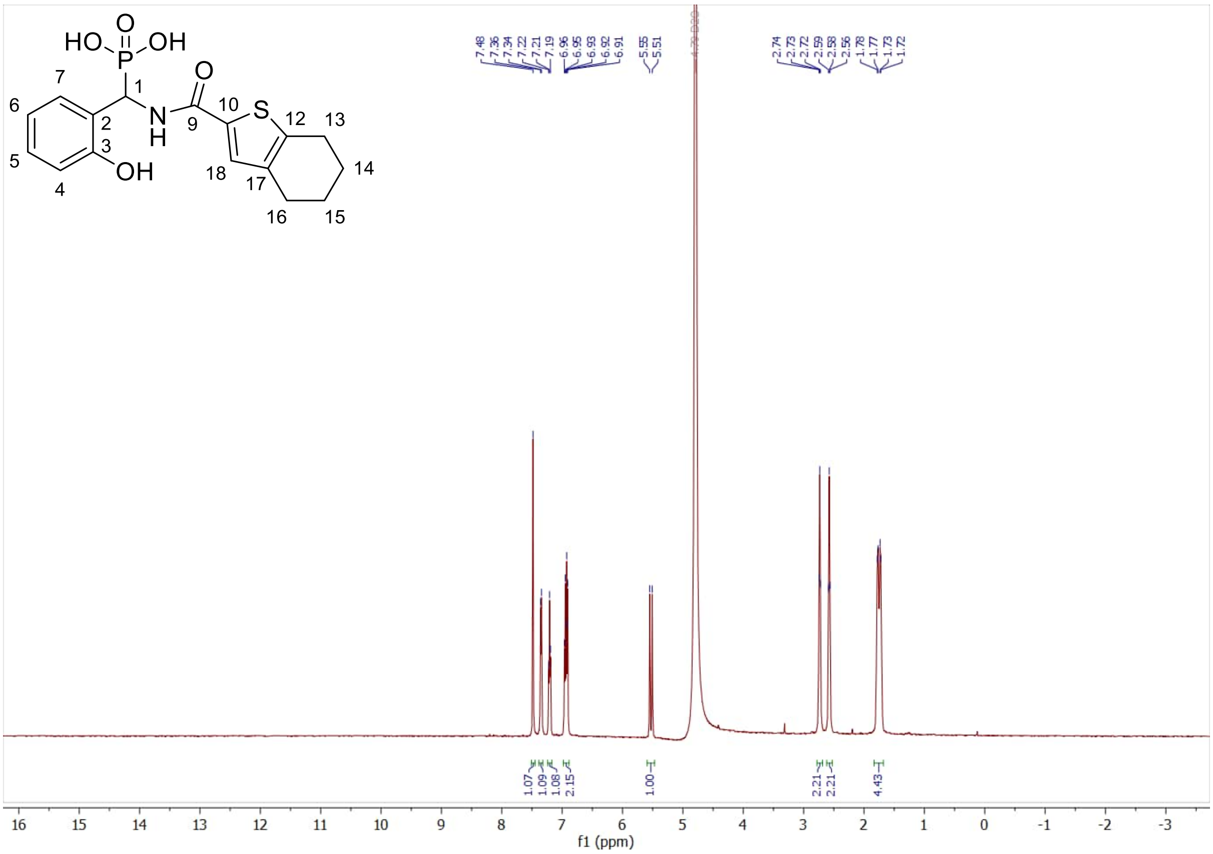

Figure S134. *^{1}H NMR spectrum of* ***5e*** *(500 MHz, D_2O).*

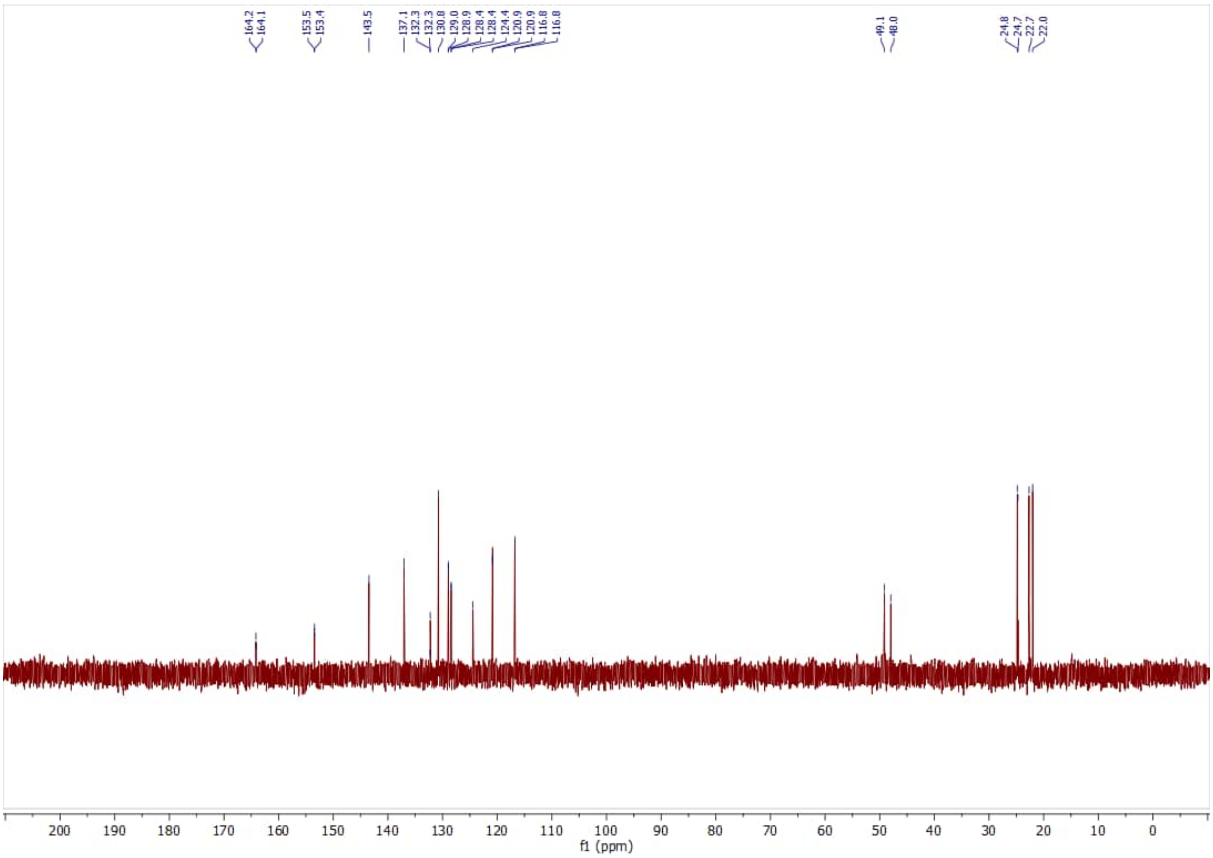

Figure S135. *^{13}C NMR spectrum of **5e** (500 MHz, D_2O).*

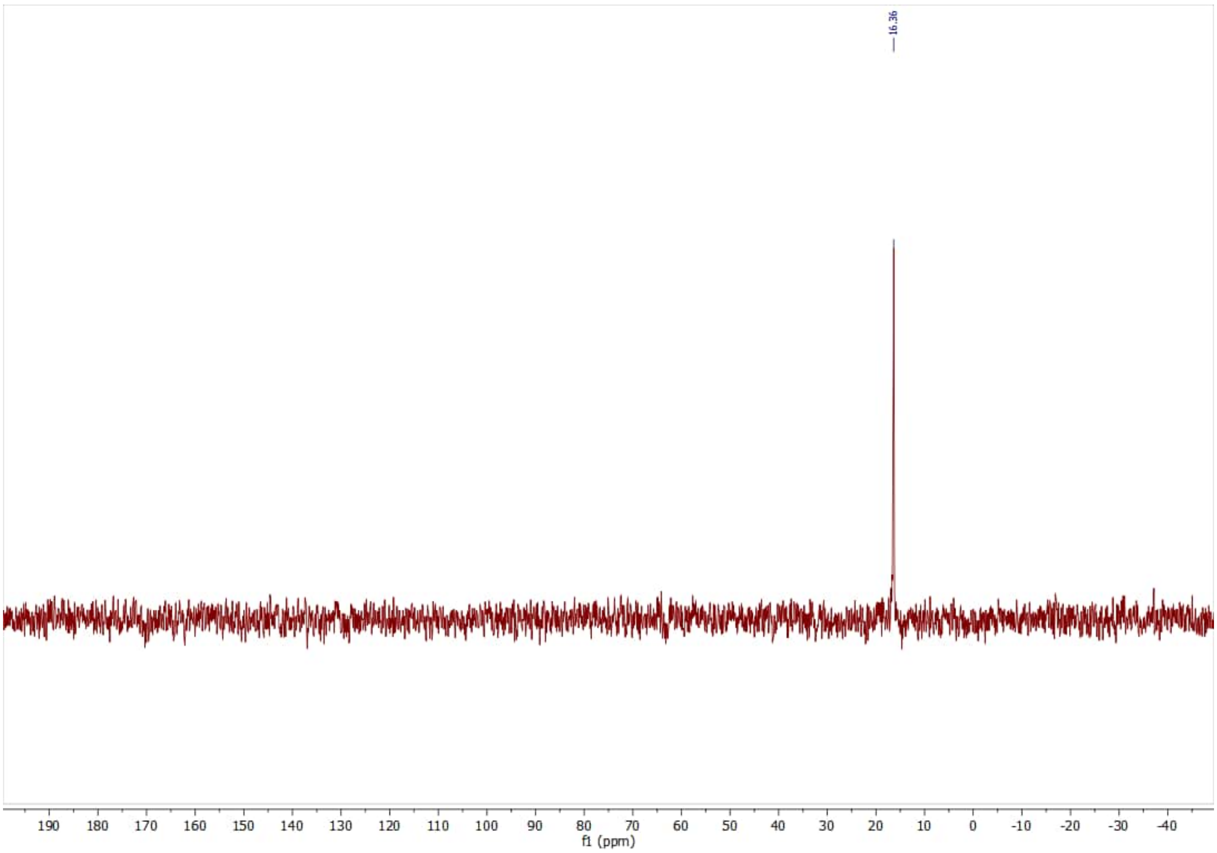

Figure S136. *^{31}P NMR spectrum of **5e** (400 MHz, D_2O).*

((2-Hydroxyphenyl)(4,5,6,7-tetrahydrobenzo[*b*]thiophene-3-carboxamido)methyl)phosphonic acid (5f)

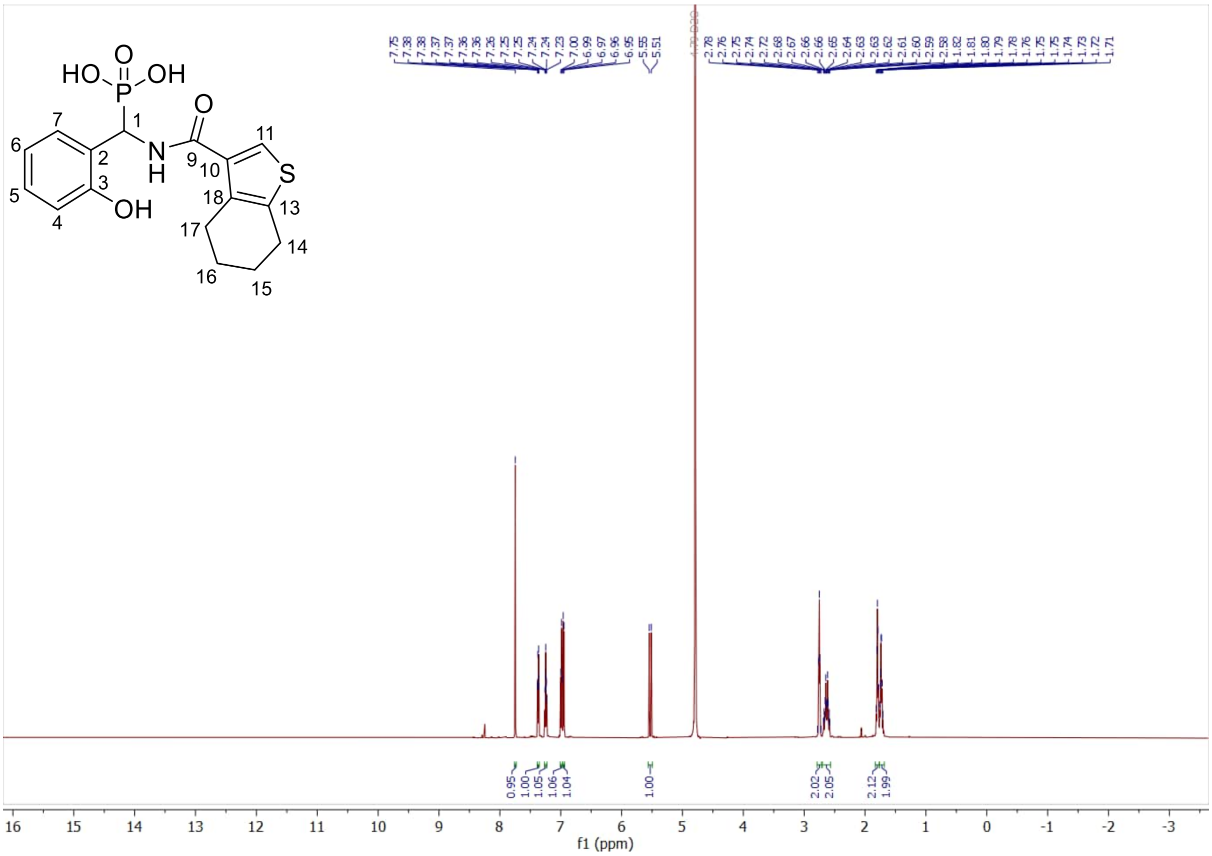

Figure S137. *^{1}H NMR spectrum of* ***5f*** *(600 MHz, D_2O).*

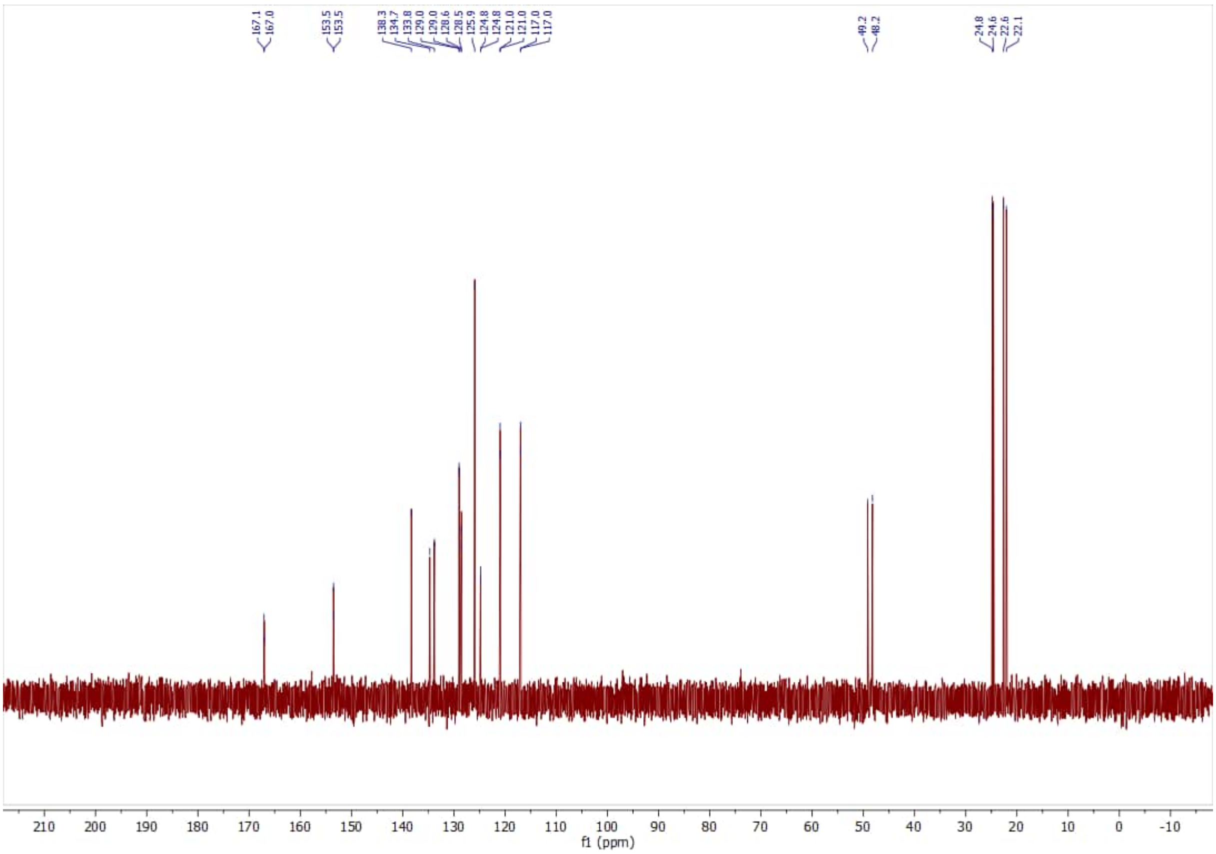

Figure S138. *^{13}C NMR spectrum of **5f** (600 MHz, D_2O).*

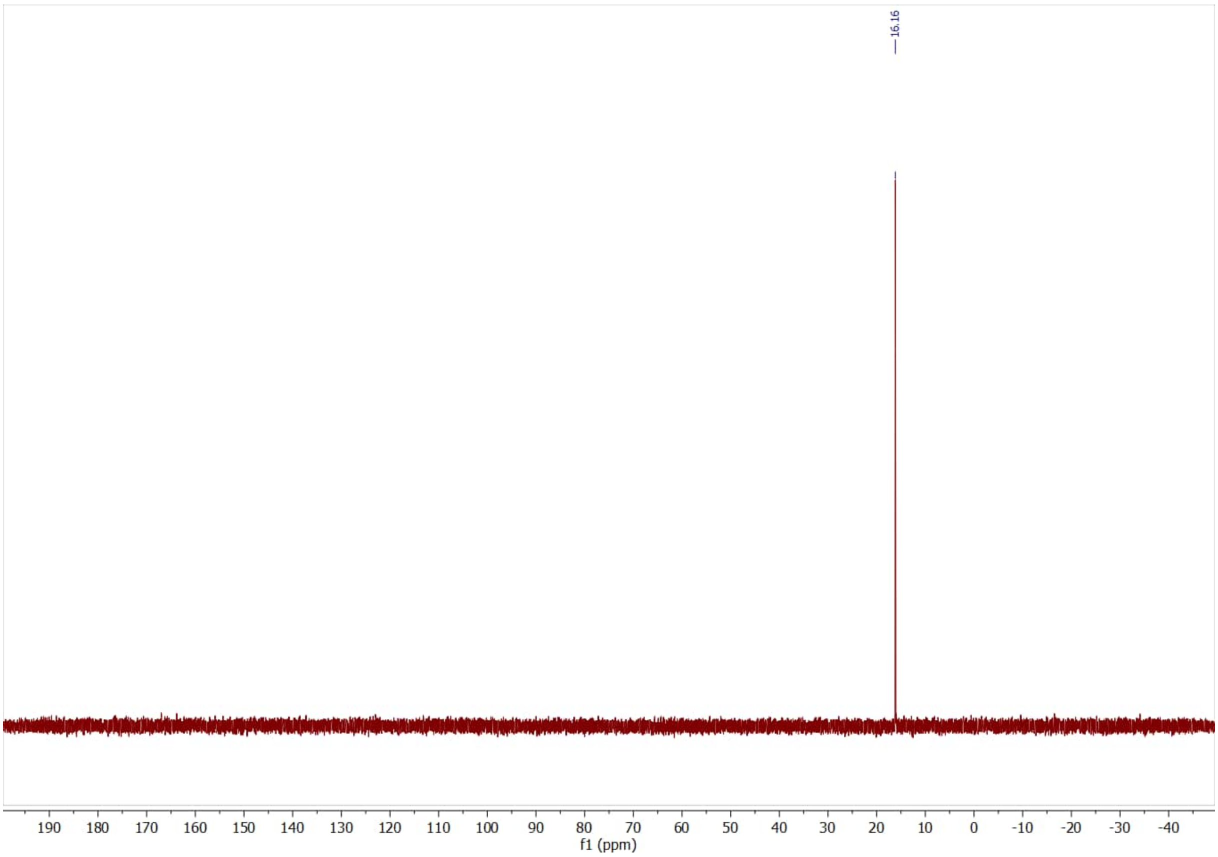

Figure S139. *^{31}P NMR spectrum of **5f** (400 MHz, D_2O).*

((2-Hydroxyphenyl)(4,5,6,7-tetrahydrobenzo[*c*]thiophene-1-carboxamido)methyl)phosphonic acid (5g)

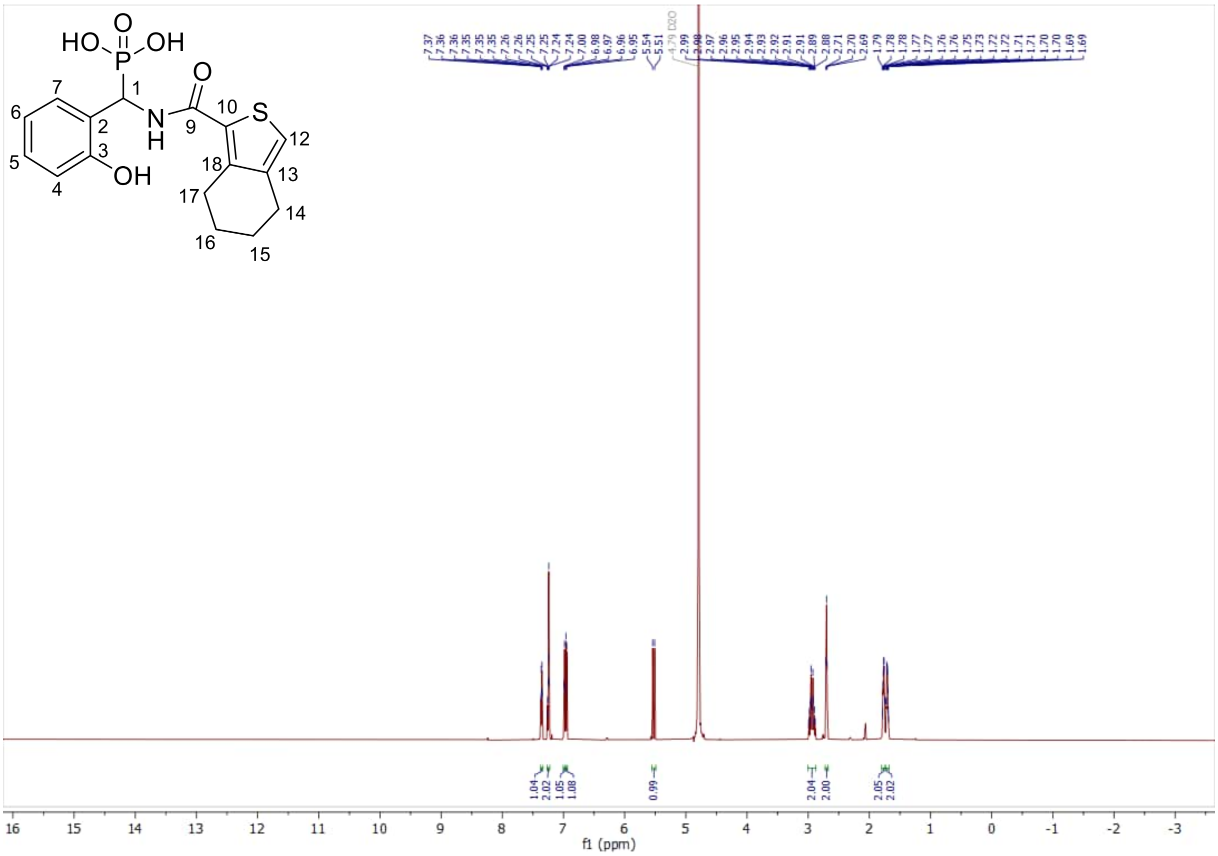

Figure S140. *^{1}H NMR spectrum of* ***5g*** *(600 MHz, D_2O).*

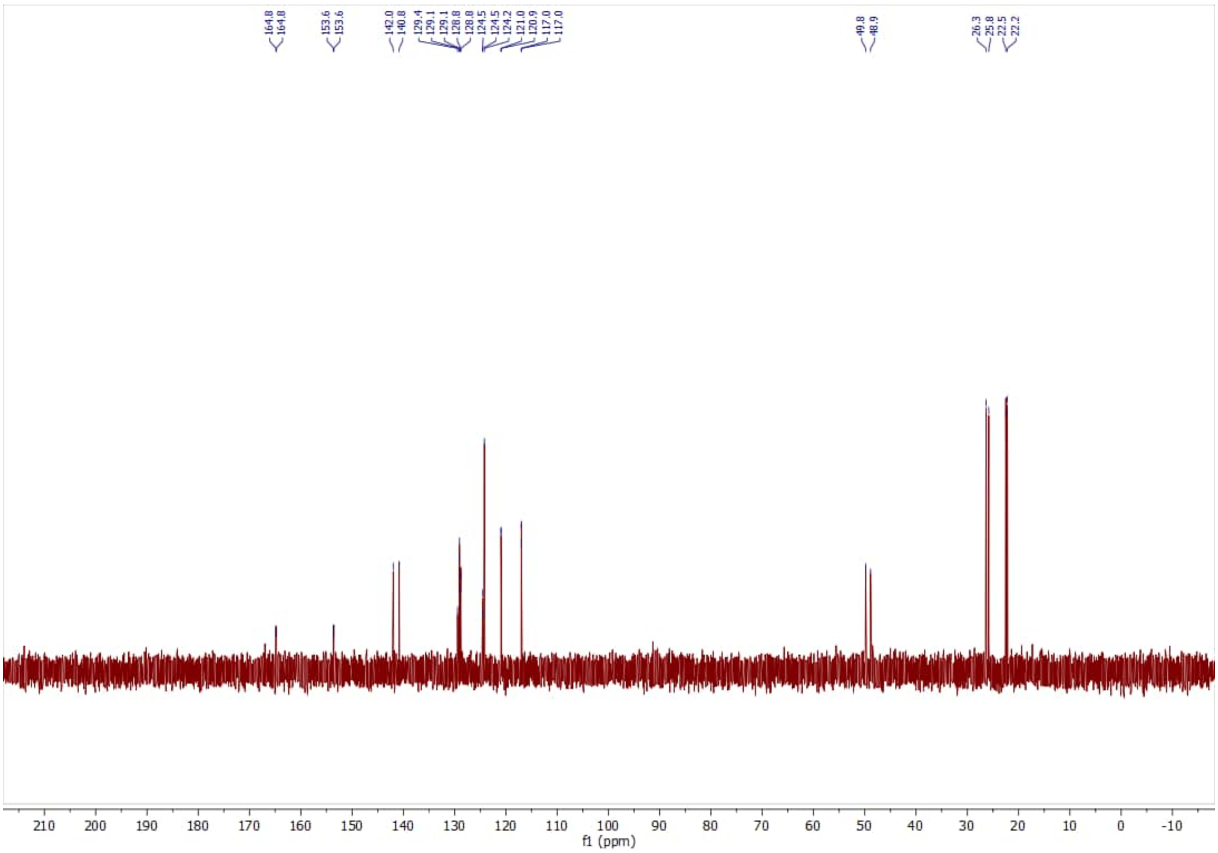

Figure S141. *^{13}C NMR spectrum of **5g** (600 MHz, D_2O).*

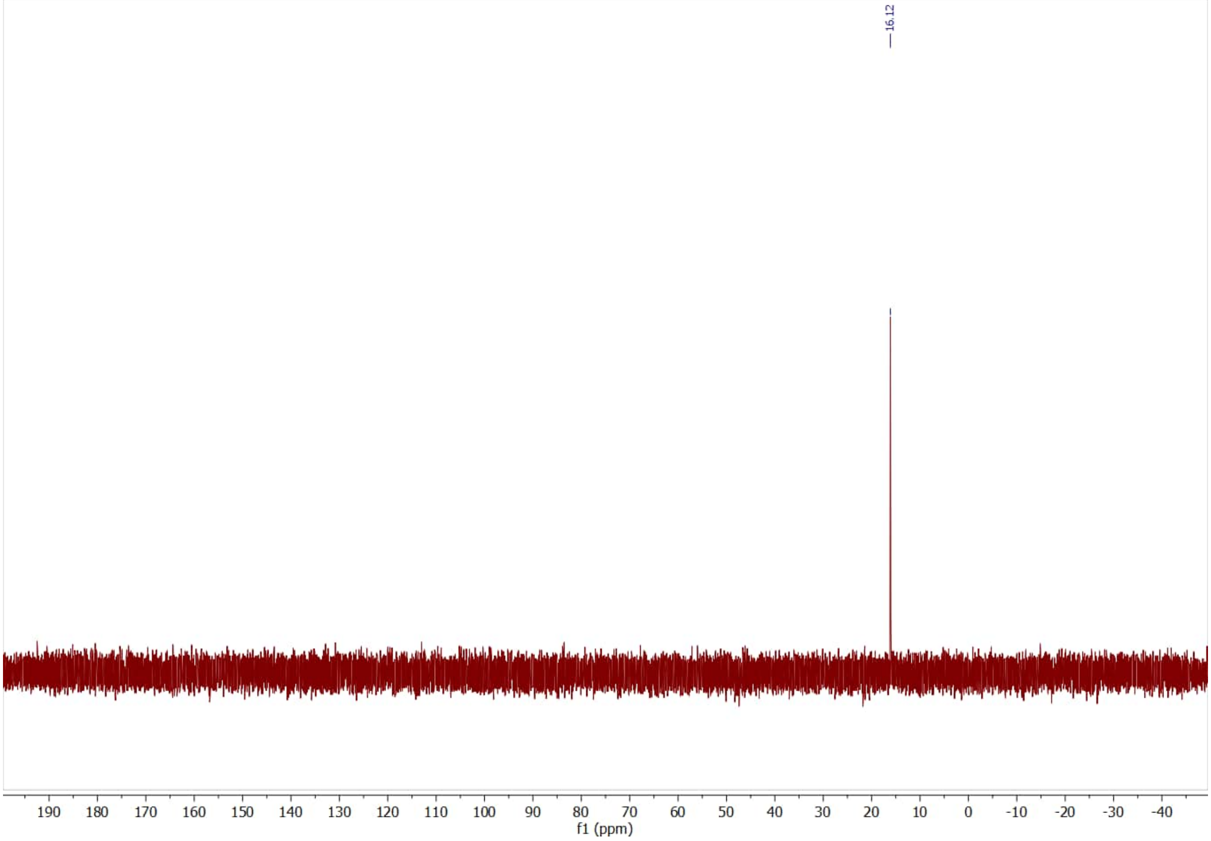

Figure S142. *^{31}P NMR spectrum of **5g** (500 MHz, D_2O).*

(Benzamido(2-hydroxyphenyl)methyl)phosphonic acid (5h)

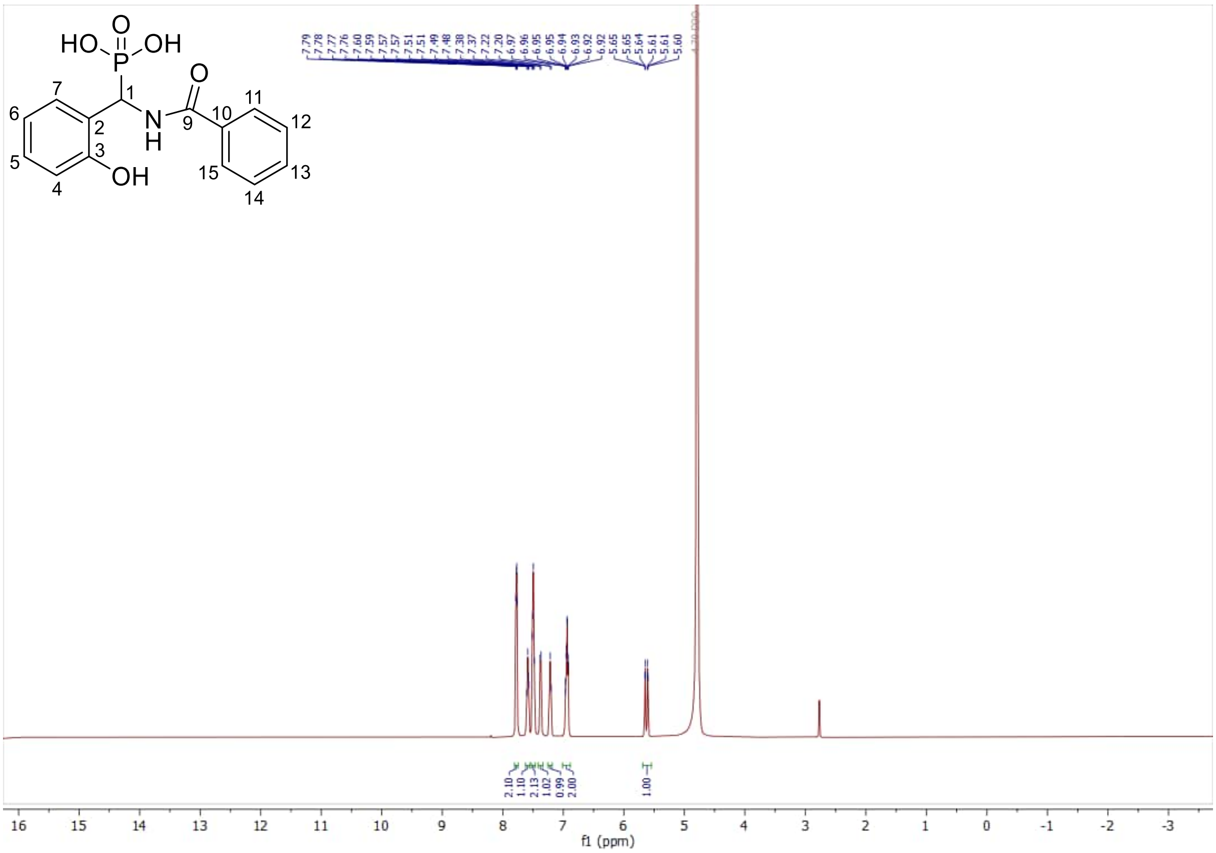

Figure S143. *^{1}H NMR spectrum of **5h** (500 MHz, D_2O).*

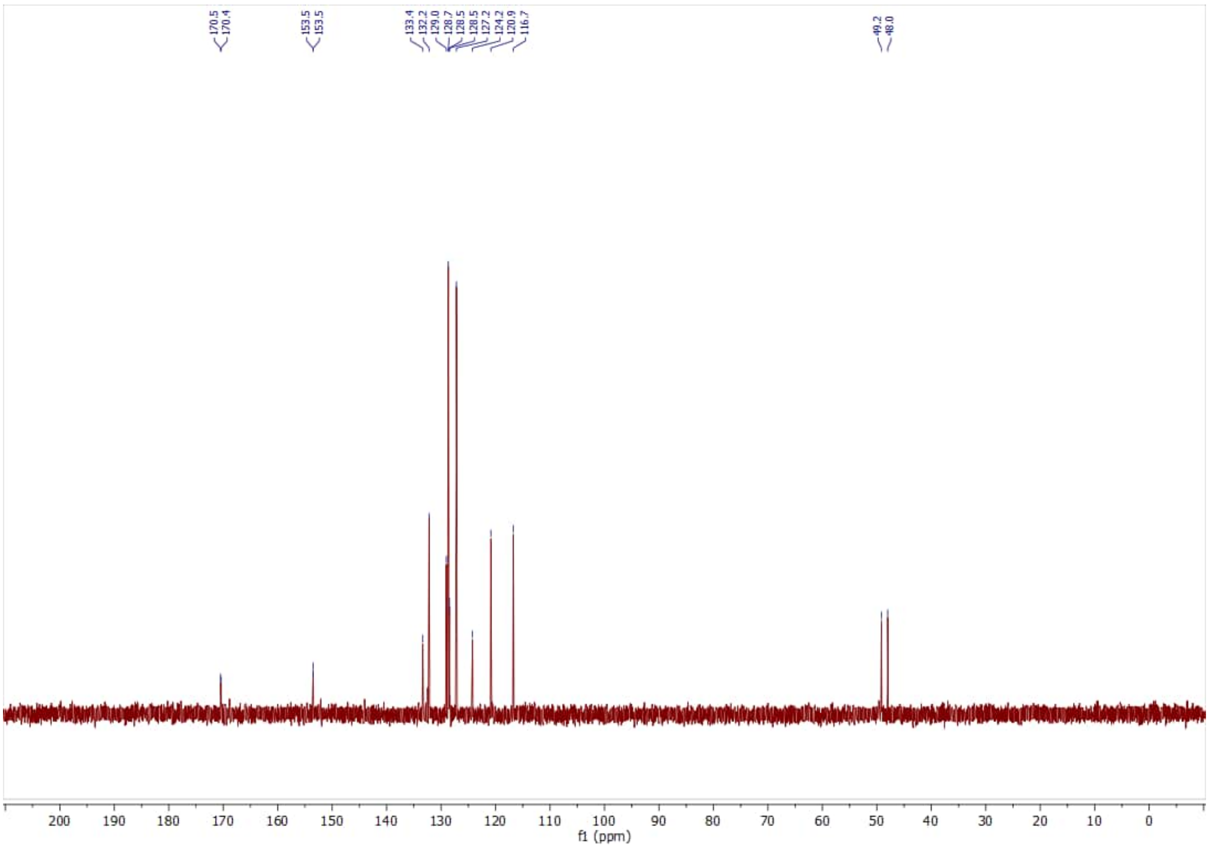

Figure S144. *^{13}C NMR spectrum of **5h** (500 MHz, D_2O).*

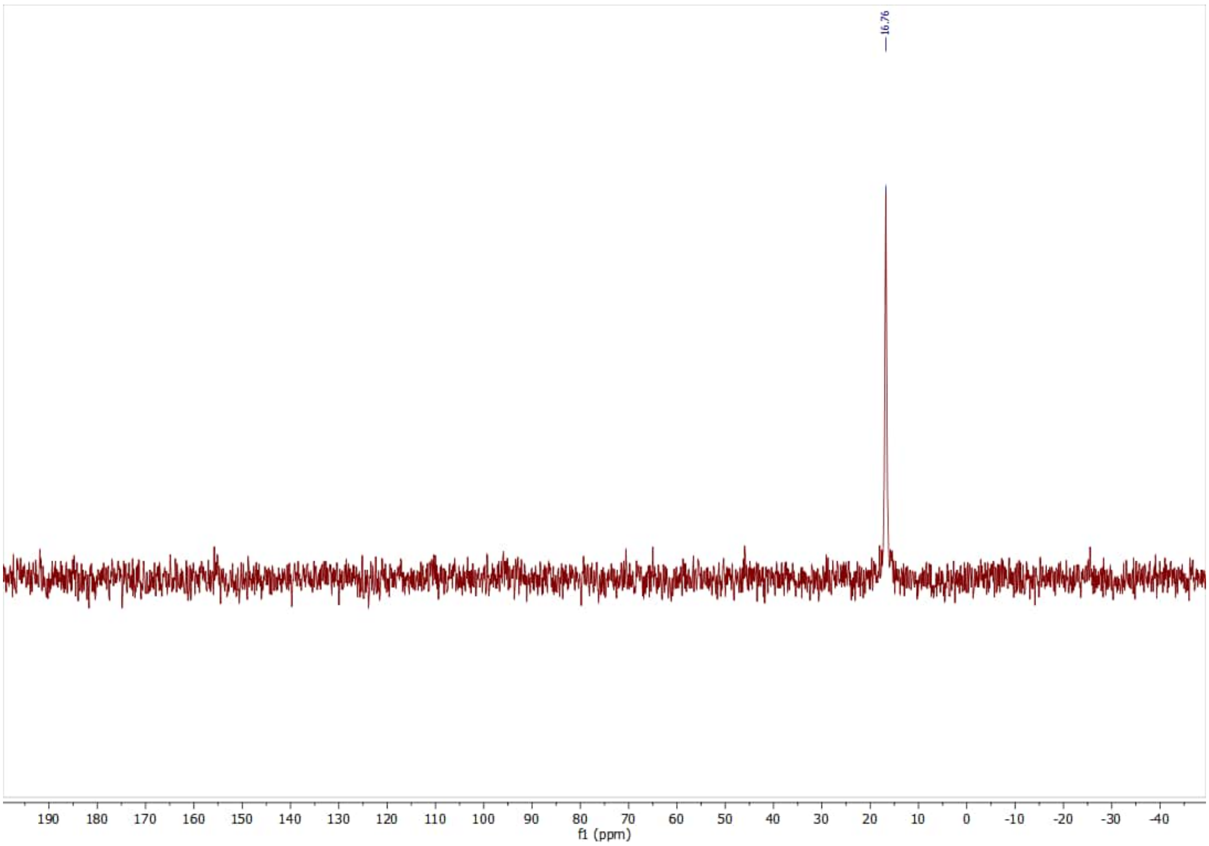

Figure S145. *^{31}P NMR spectrum of **5h** (400 MHz, D_2O).*

((2-Hydroxyphenyl)(2-(thiophen-2-yl)acetamido)methyl)phosphonic acid (5i)

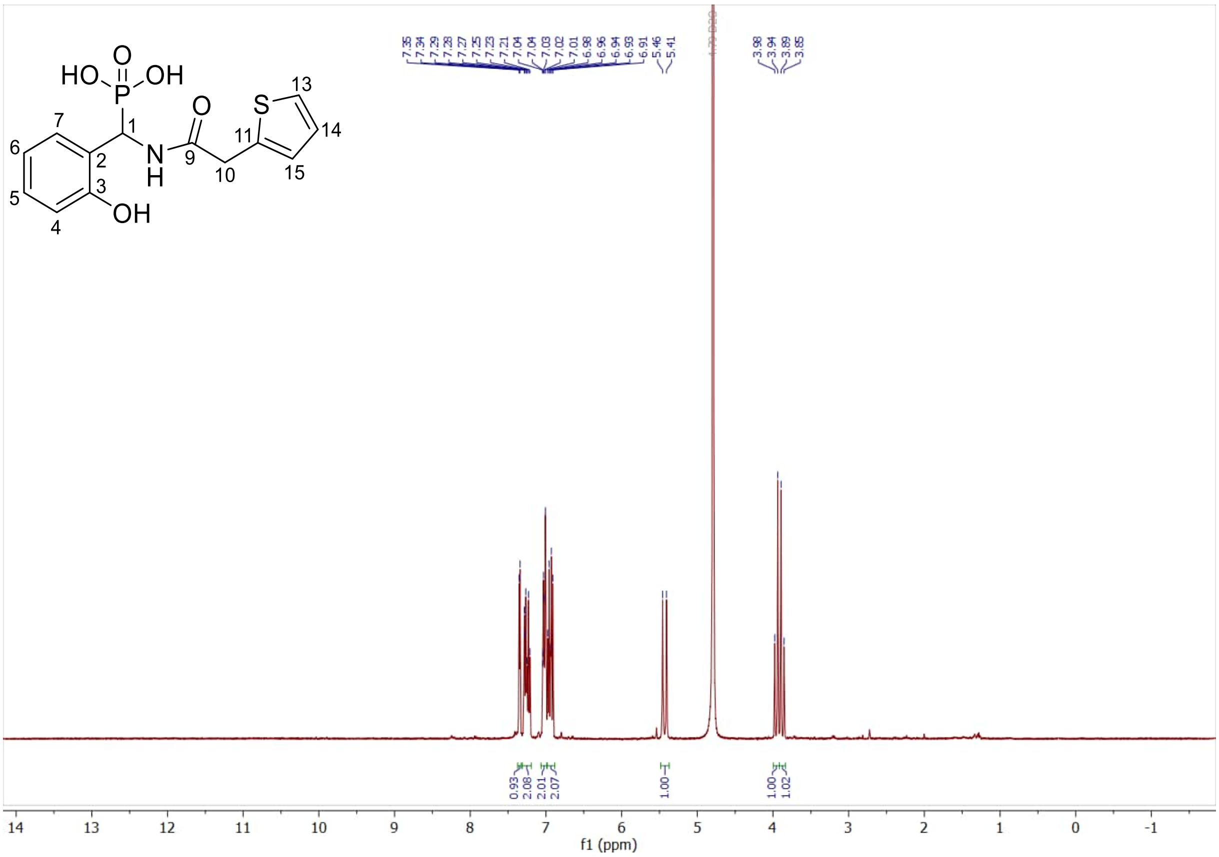

Figure S146. *^{1}H NMR spectrum of* ***5i*** *(400 MHz, D_2O).*

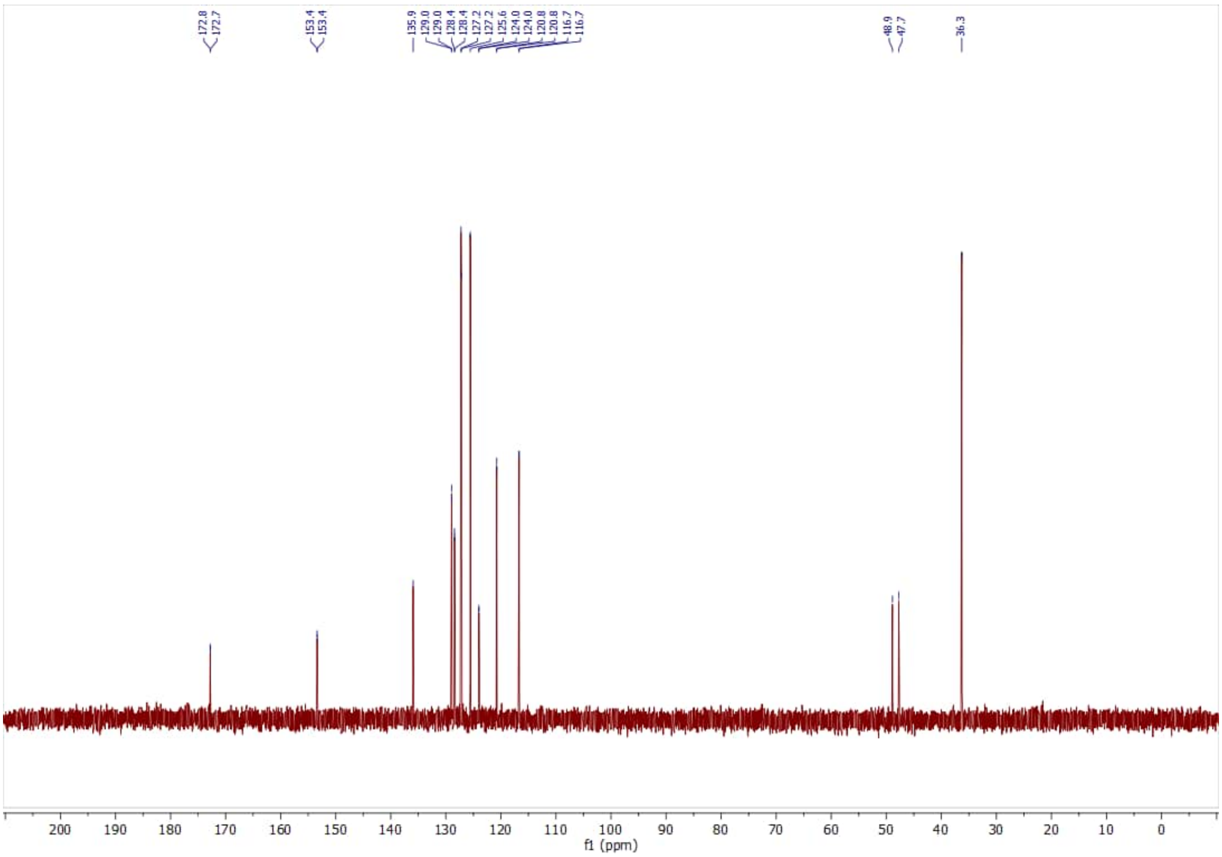

Figure S147. *^{13}C NMR spectrum of **5i** (500 MHz, D_2O).*

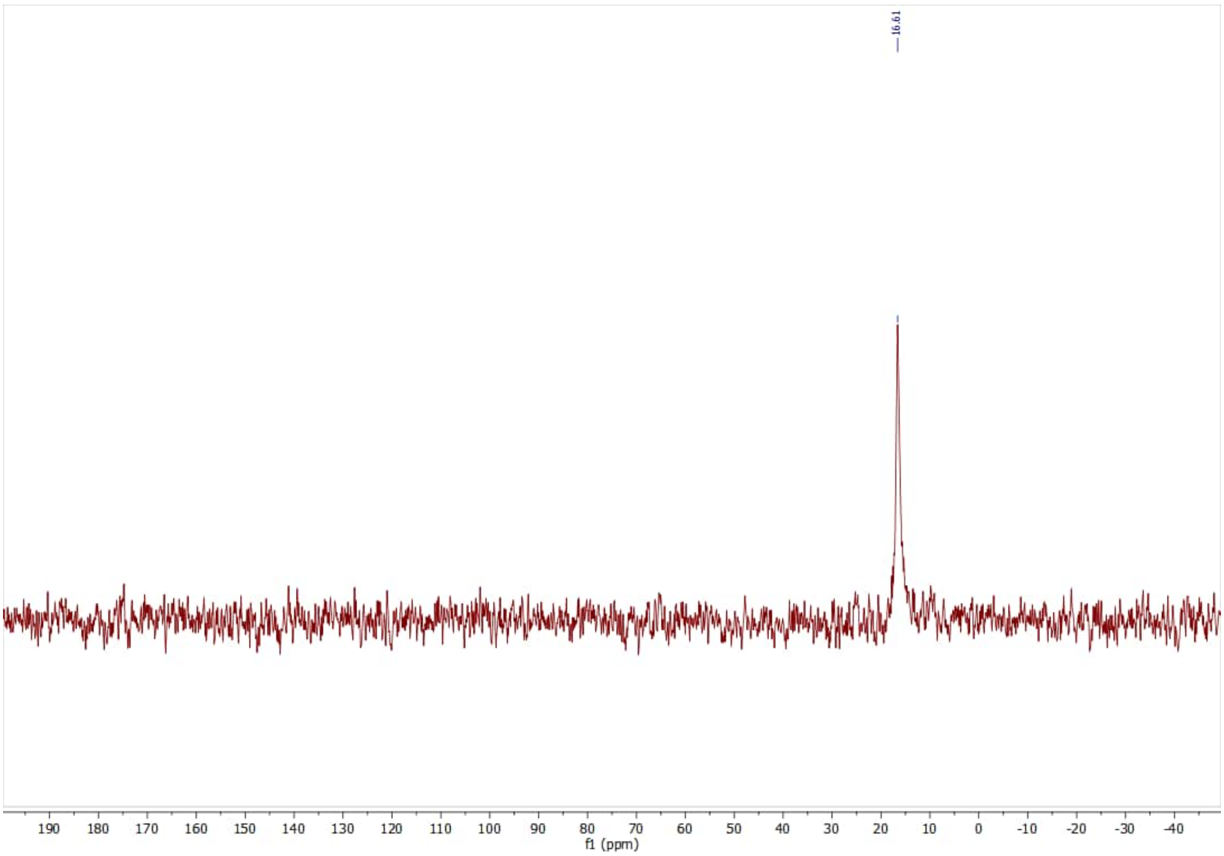

Figure S148. *^{31}P NMR spectrum of **5i** (400 MHz, D_2O).*

((2-Hydroxyphenyl)(2-(thiophen-3-yl)acetamido)methyl)phosphonic acid (5j)

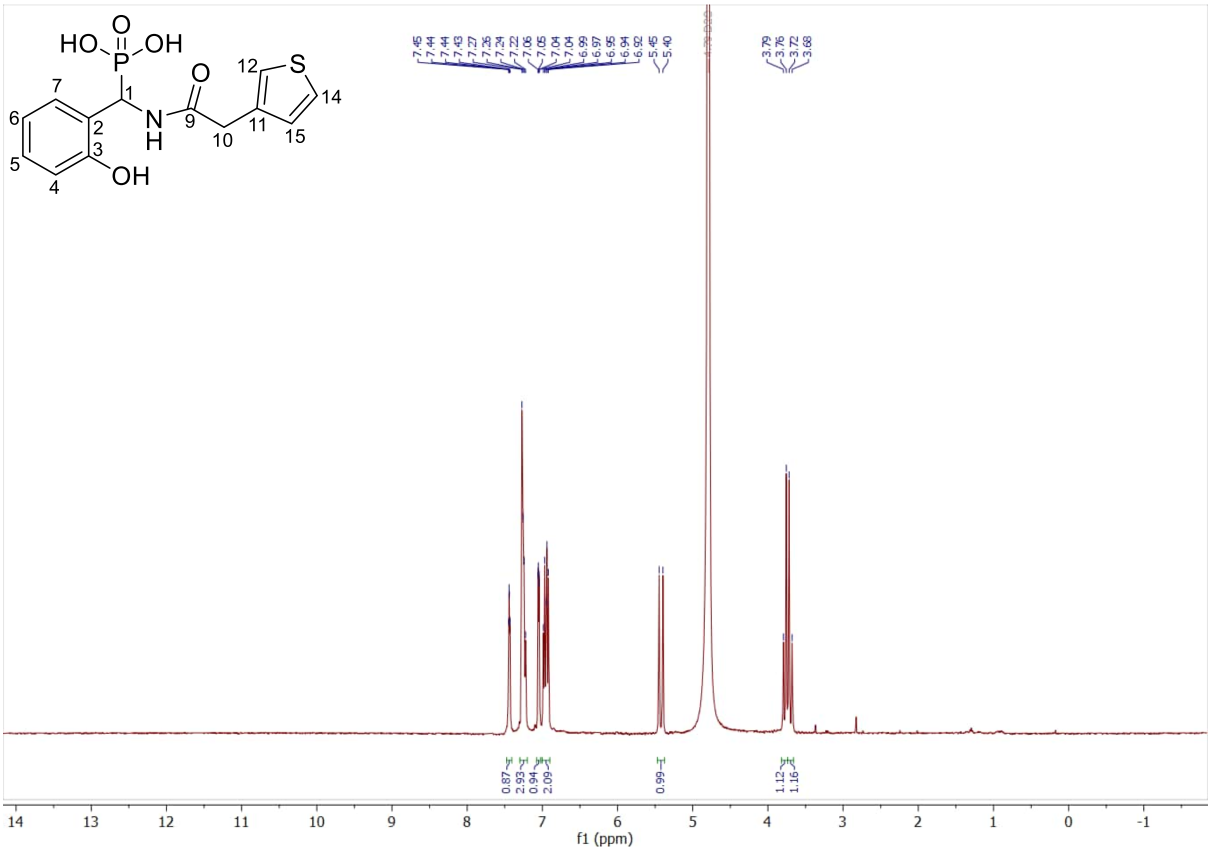

Figure S149. *^{1}H NMR spectrum of* ***5j*** *(400 MHz, D_2O).*

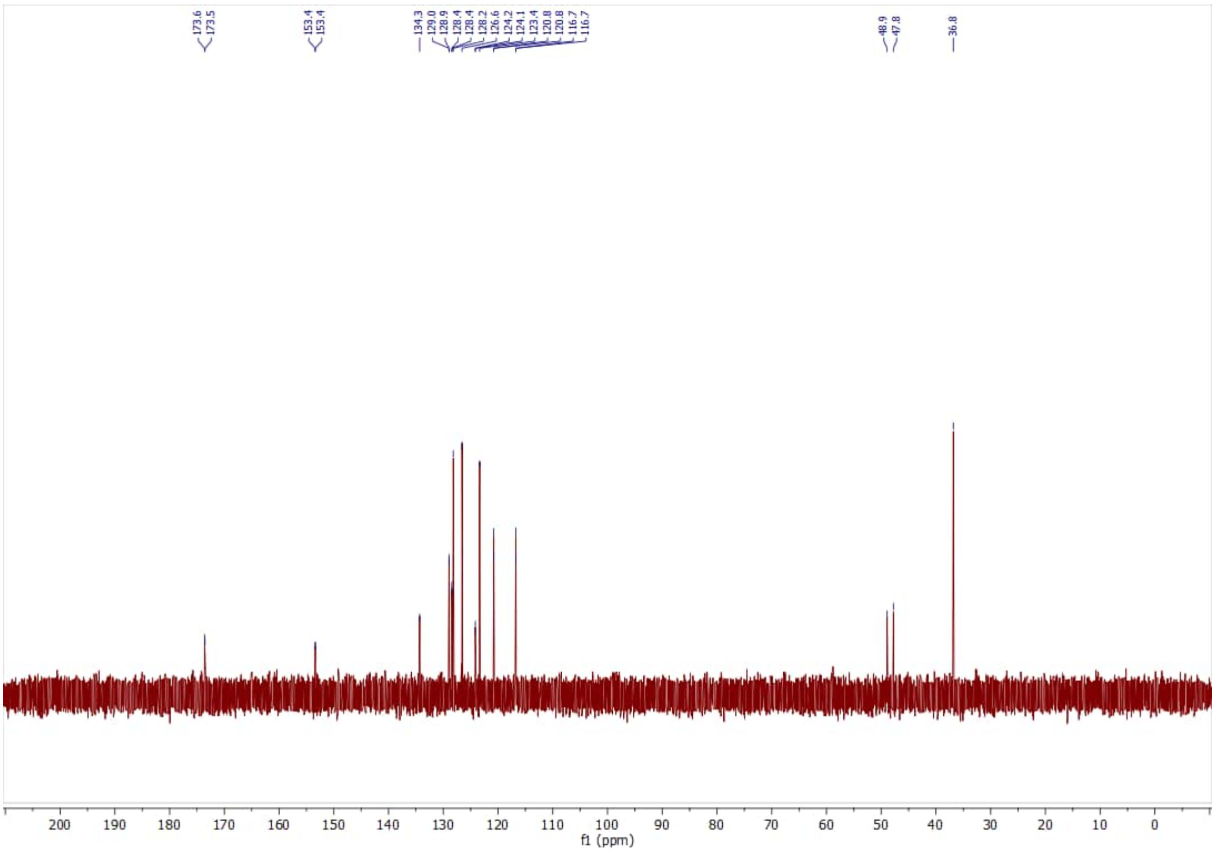

Figure S150. *^{13}C NMR spectrum of* ***5j*** *(500 MHz, D_2O).*

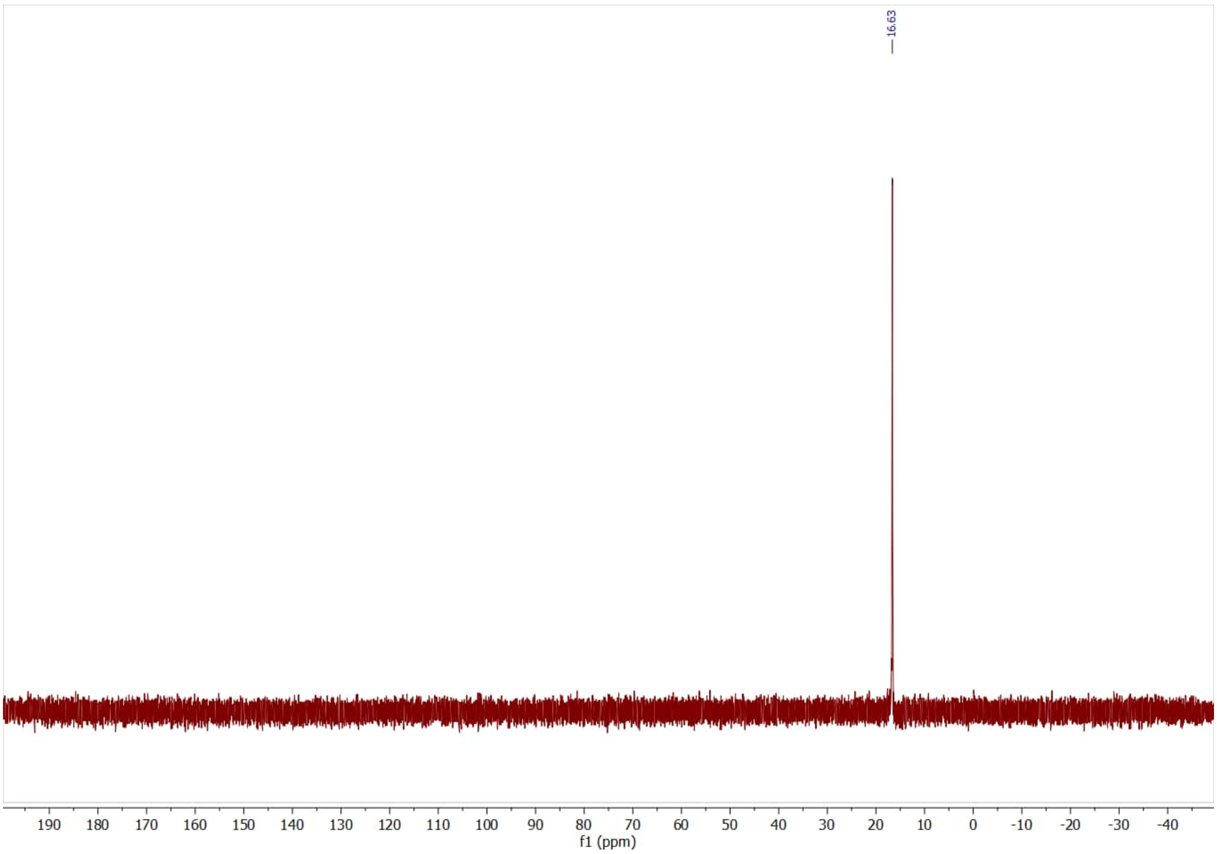

Figure S151. *^{31}P NMR spectrum of* ***5j*** *(400 MHz, D_2O).*

((2-(Benzo[*b*]thiophen-2-yl)acetamido)(2-hydroxyphenyl)methyl)phosphonic acid (5k)

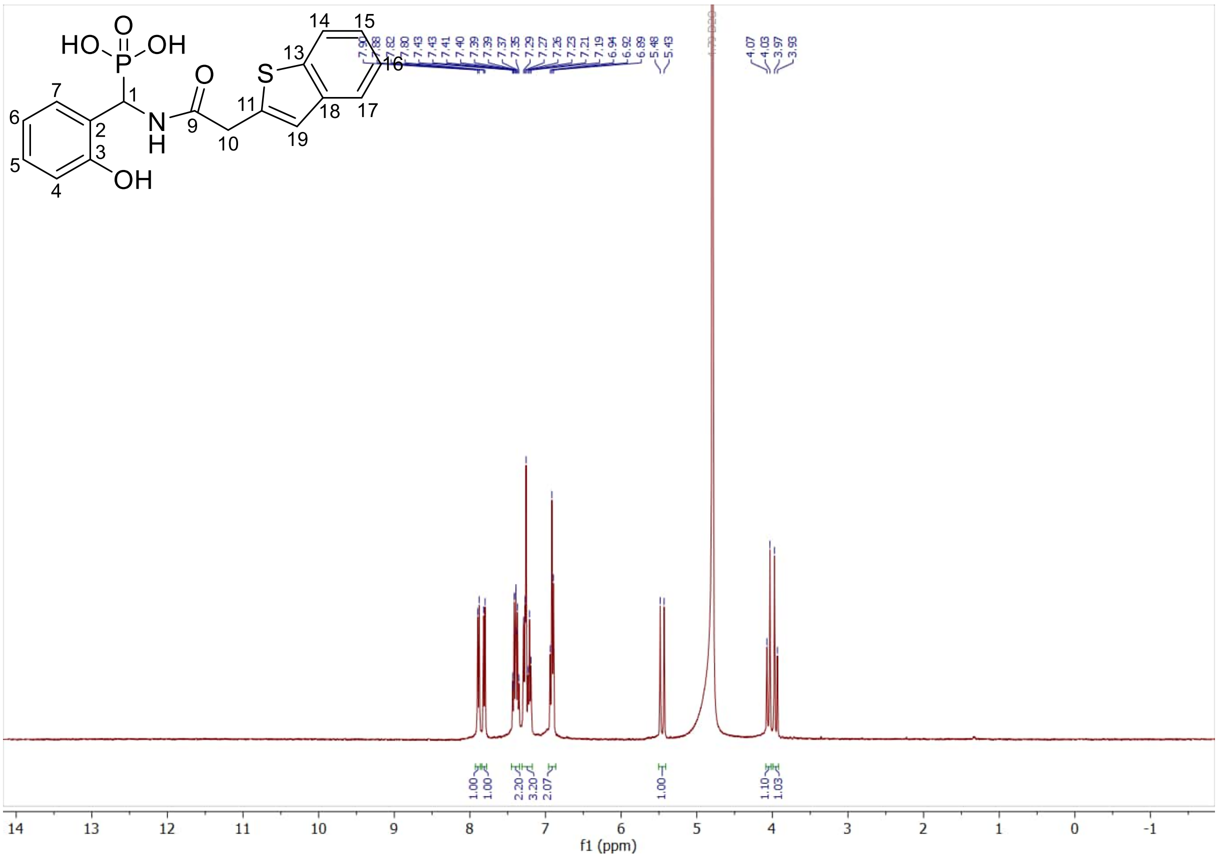

Figure S152. *^{1}H NMR spectrum of* ***5k*** *(400 MHz, D_2O).*

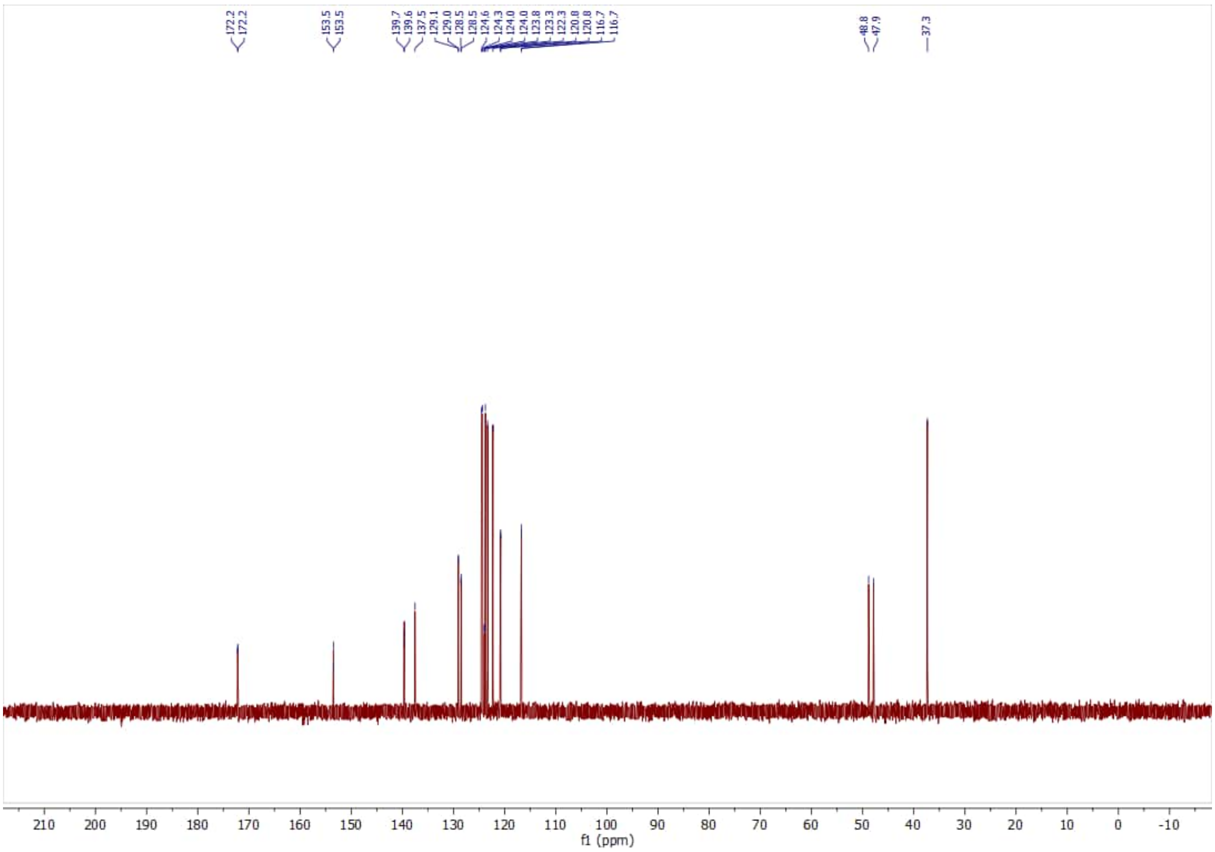

Figure S153. *^{13}C NMR spectrum of **5k** (600 MHz, D_2O).*

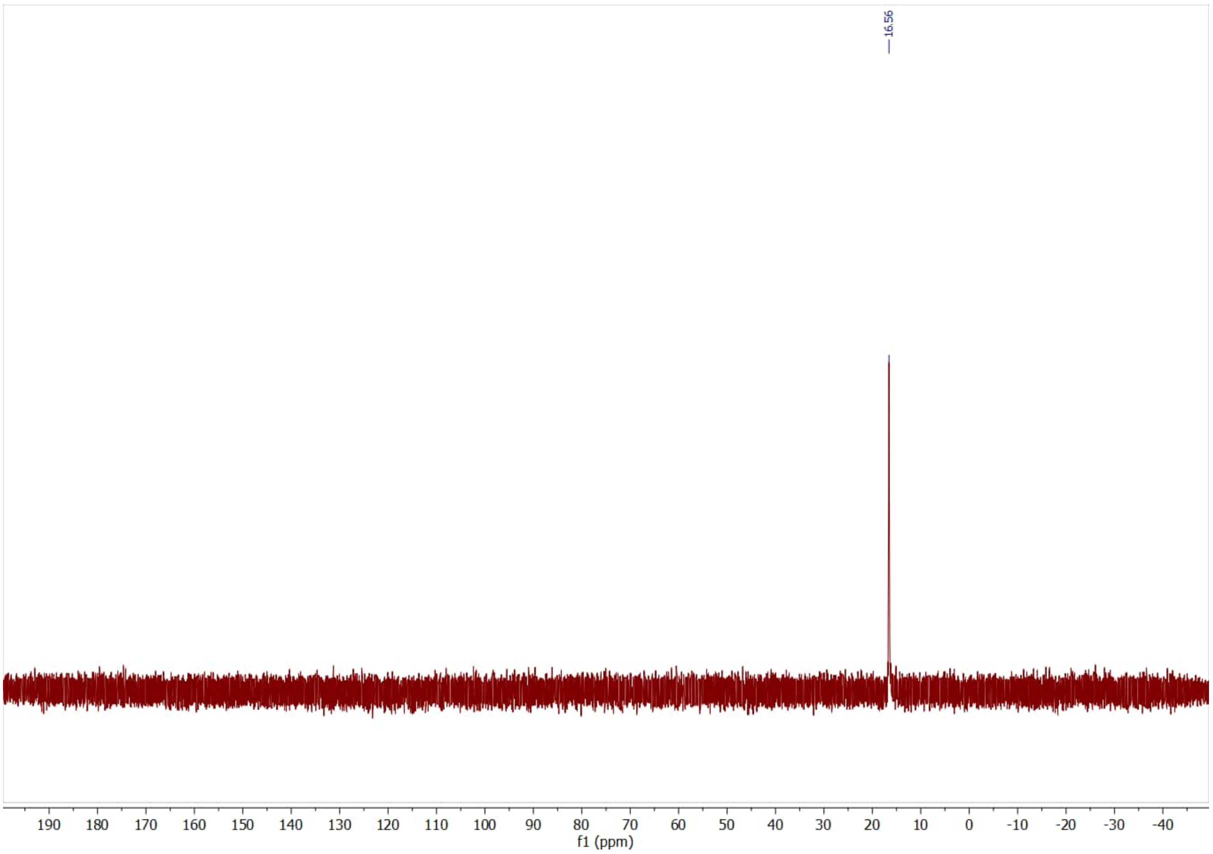

Figure S154. *^{31}P NMR spectrum of **5k** (400 MHz, D_2O).*

((2-(Benzo[*b*]thiophen-3-yl)acetamido)(2-hydroxyphenyl)methyl)phosphonic acid (5l)

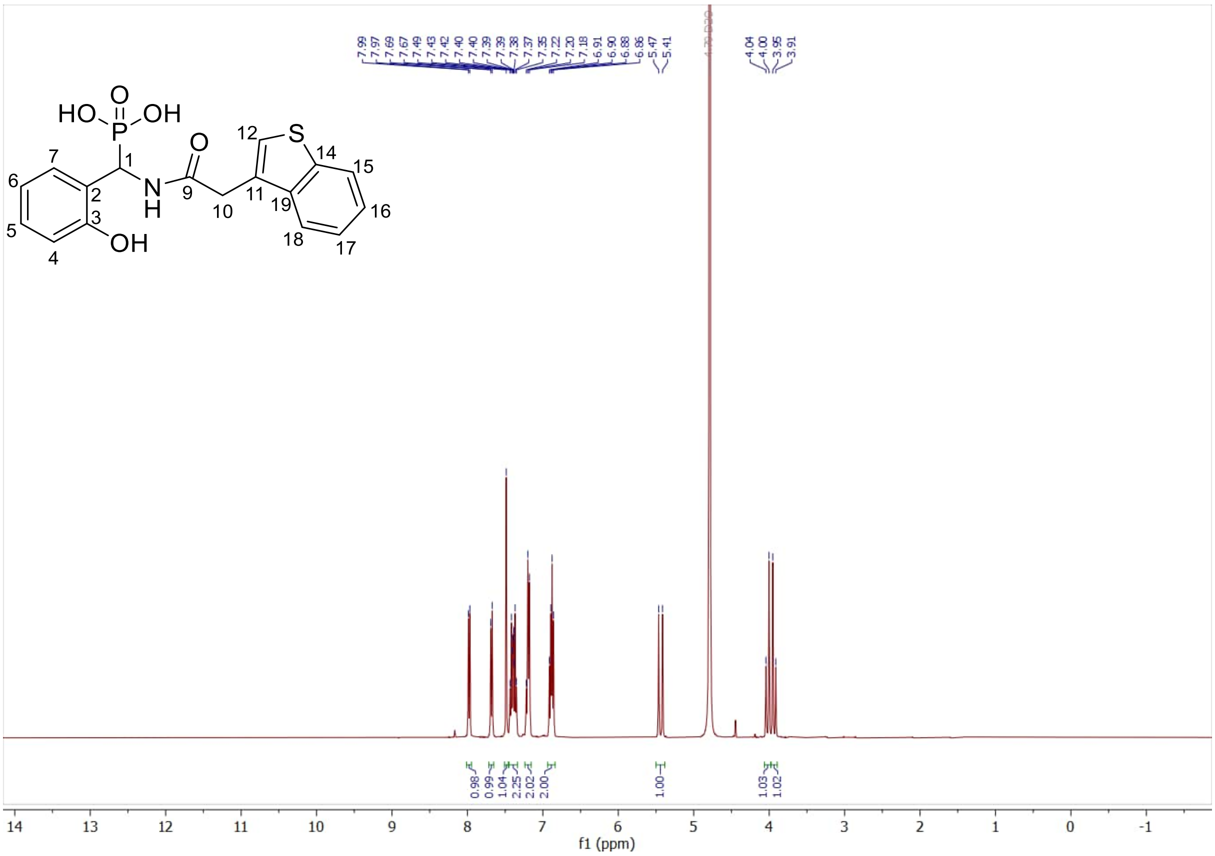

Figure S155. *^{1}H NMR spectrum of* ***5l*** *(400 MHz, D_2O).*

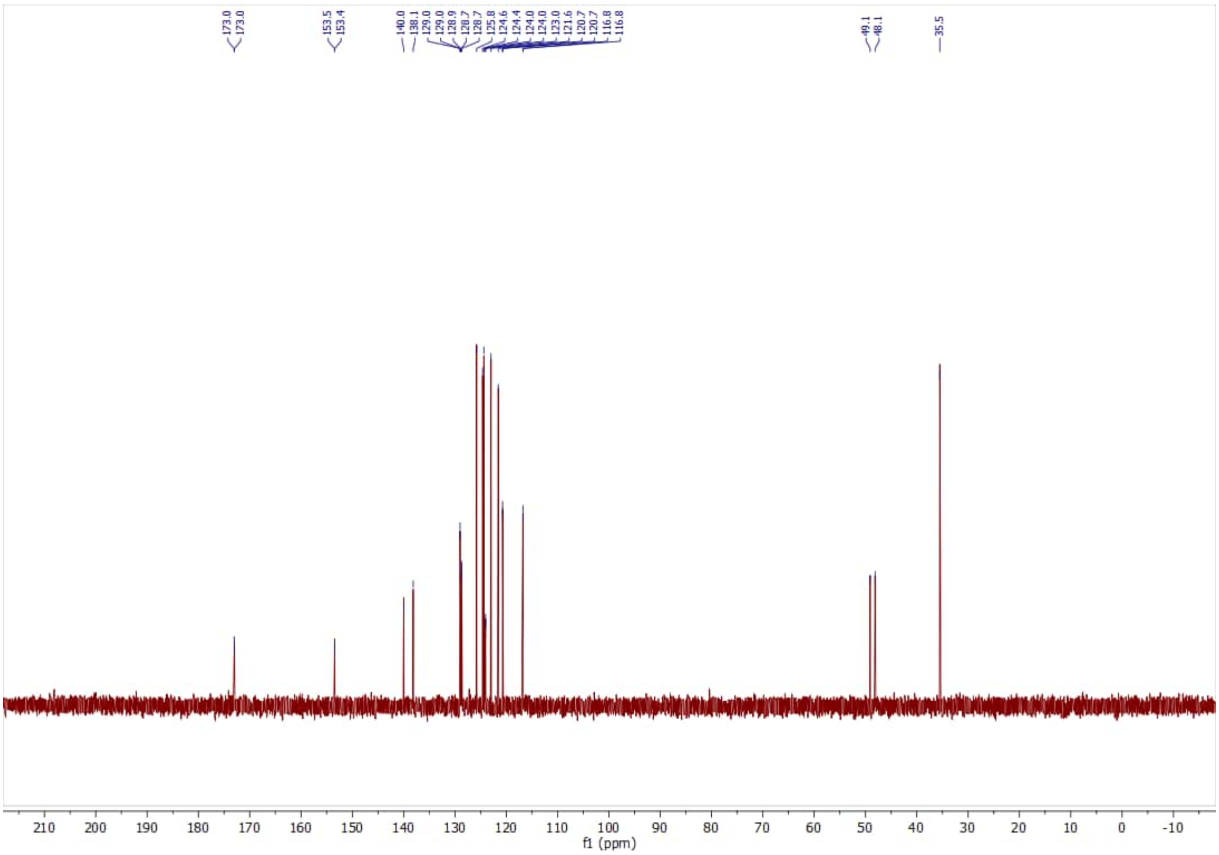

Figure S156. *^{13}C NMR spectrum of* ***5l*** *(600 MHz, D_2O).*

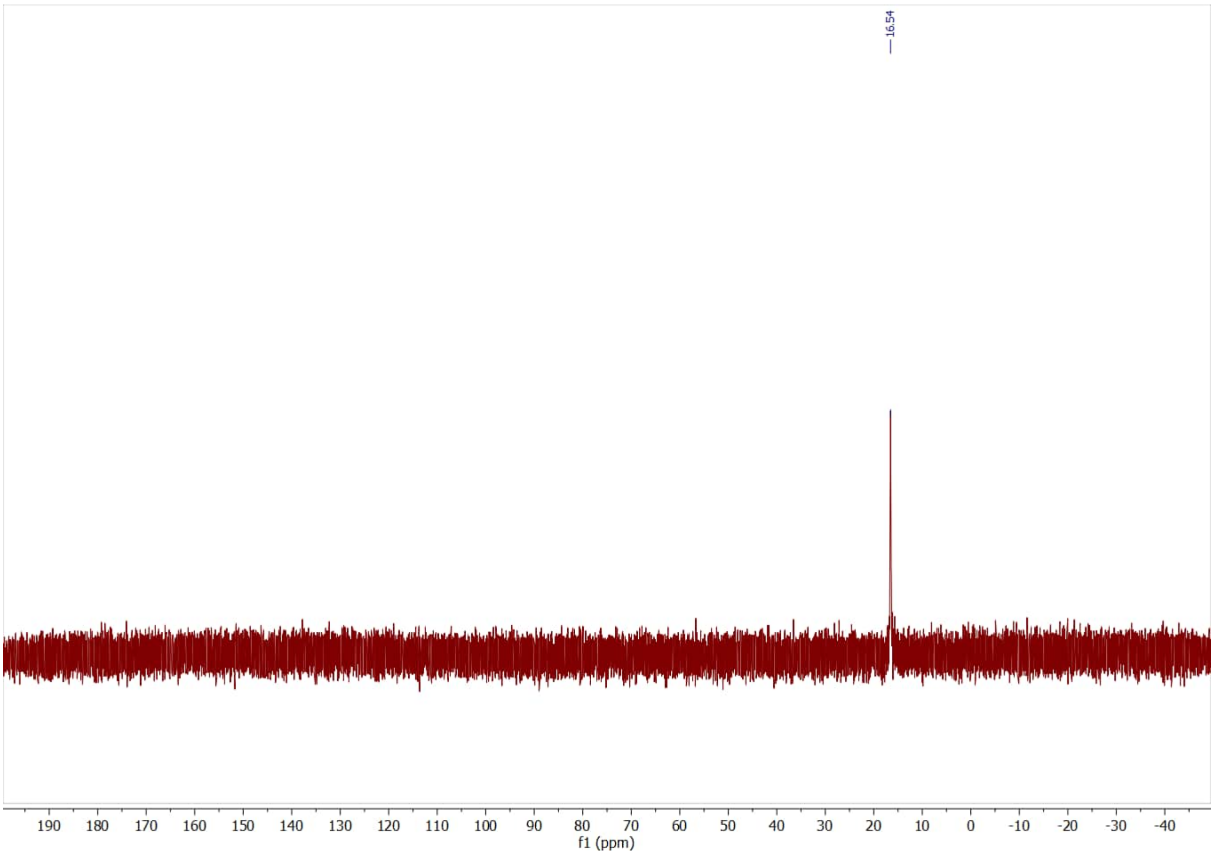

Figure S157. *^{31}P NMR spectrum of* ***5l*** *(400 MHz, D_2O).*

((2-Hydroxyphenyl)(2-phenylacetamido)methyl)phosphonic acid (5m)

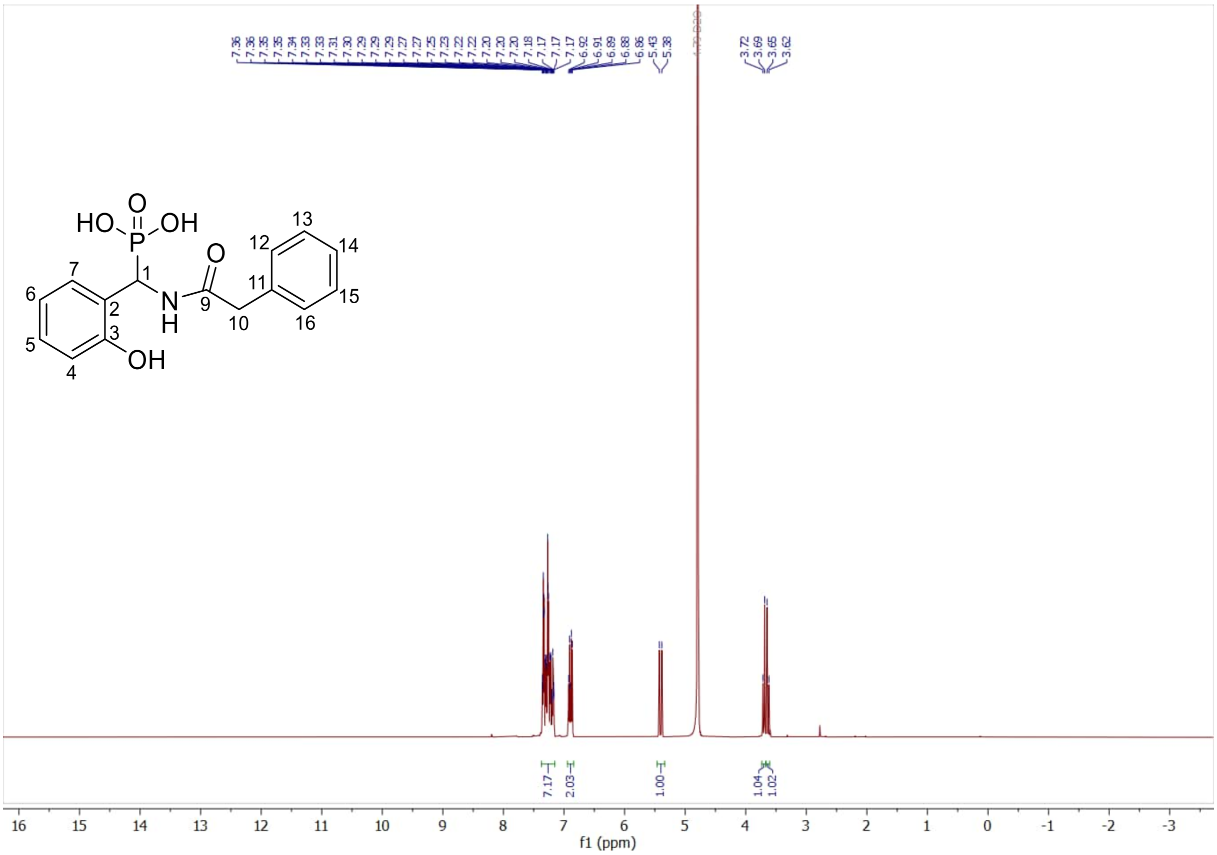

Figure S158. *^{1}H NMR spectrum of* ***5m*** *(500 MHz, D_2O).*

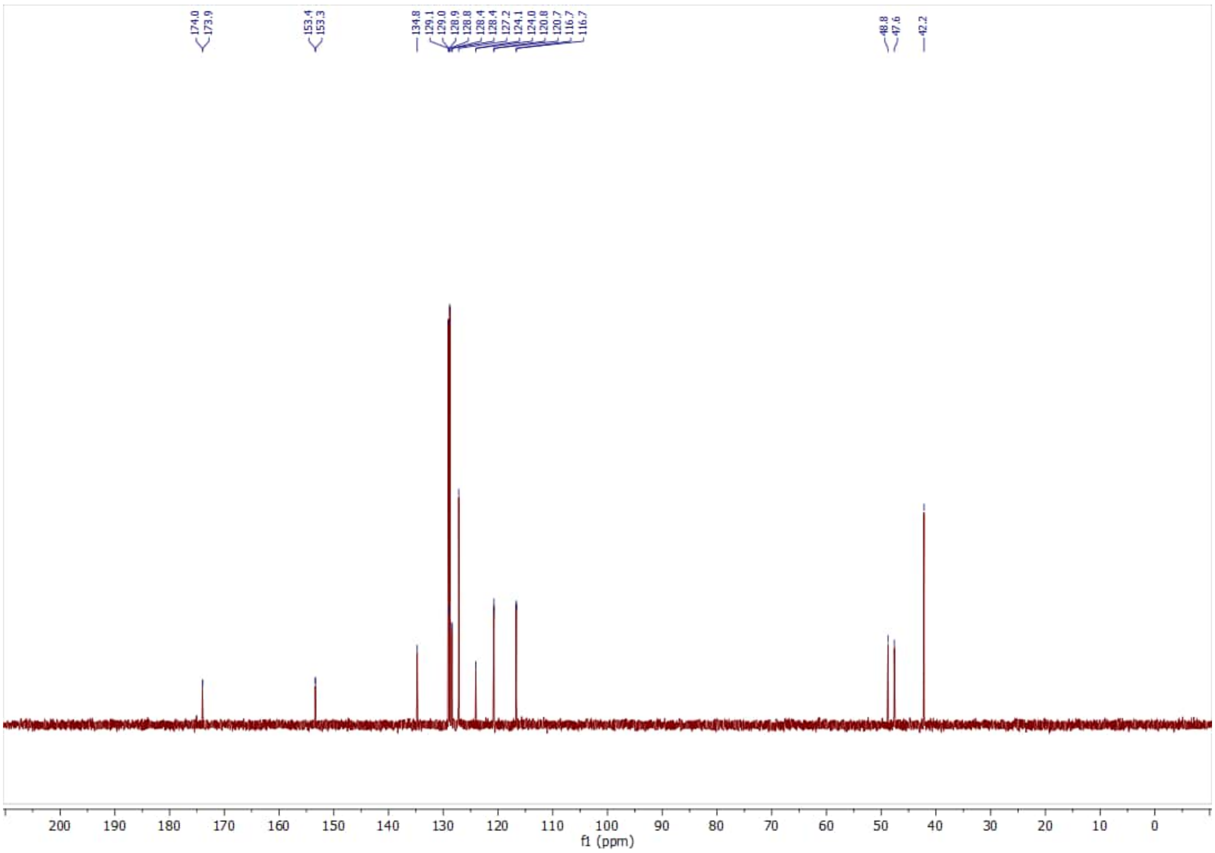

Figure S159. *^{13}C NMR spectrum of **5m** (500 MHz, D_2O).*

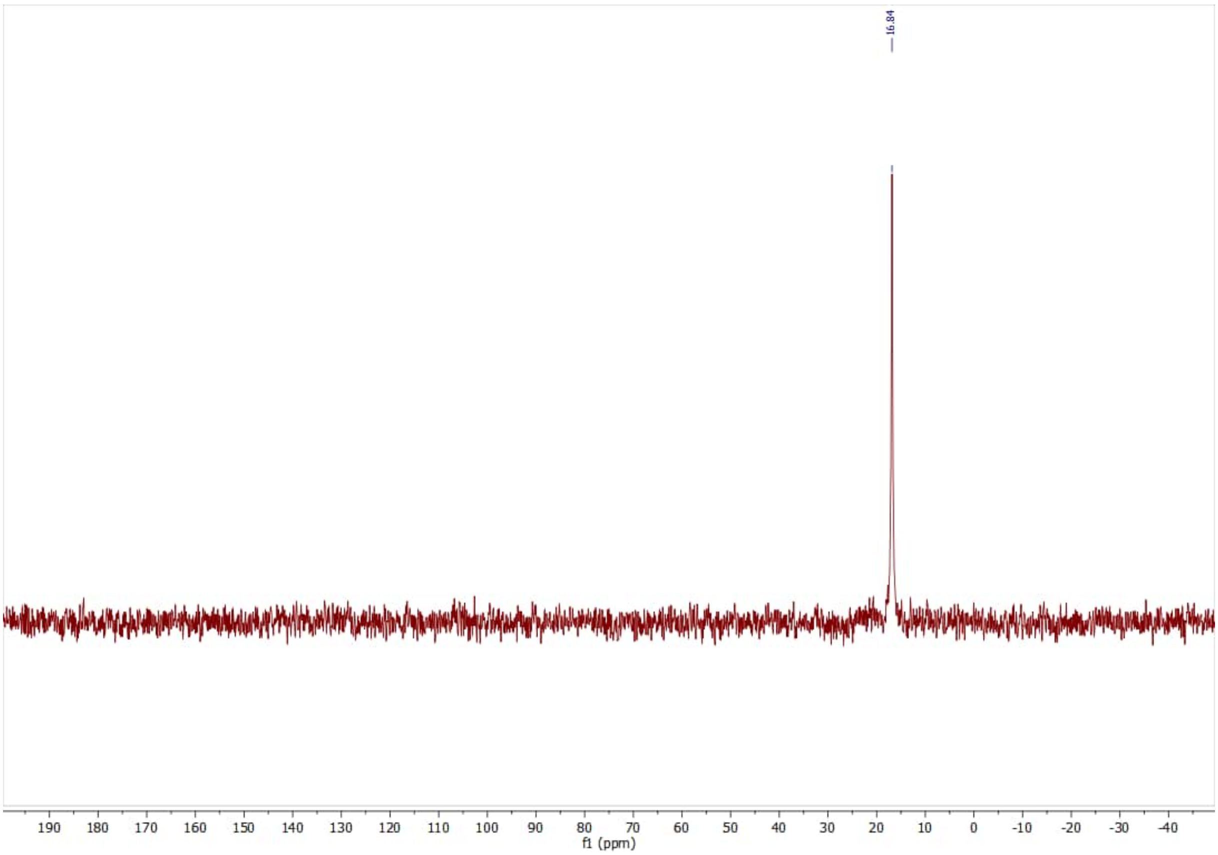

Figure S160. *^{31}P NMR spectrum of **5m** (400 MHz, D_2O).*

17. References

1. Galleni, M. et al. Standard Numbering Scheme for Class B β-Lactamases. *Antimicrob. Agents Chemother.* **45**, 660–663 (2001).
2. Mao, F. et al. Heterogeneous cobalt catalysts for selective oxygenation of alcohols to aldehydes, esters and nitriles. *RSC Adv.* **7**, 1498-1503 (2017).
3. Chen, L., Xu, H., Hu, T. & Wu, Y. Synthesis of spiroketal enol ethers related to tonghaosu and their insecticidal activities. *Pest Manag Sci.* **61**, 477-82 (2005).
4. Palica, K. et al. α-Aminophosphonate inhibitors of metallo-β-lactamases NDM-1 and VIM-2. *RSC. Med. Chem.* **14**, 2277-2300 (2023).
5. Gauvry, N. & Mortier, J. Dealkylation of Dialkyl Phosphonates with Boron Tribromide. *Synthesis* **4**, 553–554 (2001).
6. Wieske, L.H.E. et al. NMR Backbone Assignment of VIM-2 and Identification of the Active Enantiomer of a Potential Inhibitor. *ACS Medicinal Chemistry Letters* **13**, 257-261 (2022).
7. Palica, K. et al. Metallo-β-Lactamase Inhibitor Phosphonamidate Monoesters. *ACS Omega* **7**, 4550–4562 (2022).
8. Mulder, F.A.A., Schipper, D., Bott, R. & Boelens, R. Altered flexibility in the substrate-binding site of related native and engineered high-alkaline Bacillus subtilisins. *J. Mol. Biol.* **292**, 111-123 (1999).
9. Borra, P.S. et al. Crystal Structures of Pseudomonas aeruginosa GIM-1: Active-Site Plasticity in Metallo-β-Lactamases. *Antimicrobial Agents and Chemotherapy* **57**, 848–854 (2013).
10. Pemberton, O.A. et al. Heteroaryl Phosphonates as Noncovalent Inhibitors of Both Serineand Metallocarbapenemases. *J. Med. Chem.* **62**, 8480-8496 (2019).
11. Madhavi Sastry, G., Adzhigirey, M., Day, T., Annabhimoju, R. & Sherman, W. Protein and ligand preparation: parameters, protocols, and influence on virtual screening enrichments. *Journal of Computer-Aided Molecular Design* **27**, 221-234 (2013).
12. Friesner, R.A. et al. Glide: A New Approach for Rapid, Accurate Docking and Scoring. 1. Method and Assessment of Docking Accuracy. *Journal of Medicinal Chemistry* **47**, 1739-1749 (2004).
13. Halgren, T.A. et al. Glide: A New Approach for Rapid, Accurate Docking and Scoring. 2. Enrichment Factors in Database Screening. *Journal of Medicinal Chemistry* **47**, 1750-1759 (2004).
14. Huang, J. & MacKerell, A.D., Jr. CHARMM36 all-atom additive protein force field: validation based on comparison to NMR data. *J Comput Chem* **34**, 2135-45 (2013).
15. Jo, S., Kim, T., Iyer, V.G. & Im, W. CHARMM-GUI: A web-based graphical user interface for CHARMM. *Journal of Computational Chemistry* **29**, 1859-1865 (2008).
16. Lee, J. et al. CHARMM-GUI Input Generator for NAMD, GROMACS, AMBER, OpenMM, and CHARMM/OpenMM Simulations Using the CHARMM36 Additive Force Field. *J Chem Theory Comput* **12**, 405-13 (2016).
17. D.A. Case, H.M.A., K. Belfon, I.Y. Ben-Shalom, S.R. Brozell, D.S. Cerutti, T.E. Cheatham, III, G.A. Cisneros, V.W.D. Cruzeiro, T.A. Darden, R.E. Duke, G. Giambasu, M.K. Gilson, H. Gohlke, A.W. Goetz, R. Harris, S. Izadi, S.A. Izmailov, C. Jin, K. Kasavajhala, M.C. Kaymak, E. King, A. Kovalenko, T. Kurtzman, T.S. Lee, S. LeGrand, P. Li, C. Lin, J. Liu, T. Luchko, R. Luo, M. Machado, V. Man, M. Manathunga, K.M. Merz, Y. Miao, O. Mikhailovskii, G. Monard, H. Nguyen, K.A. O'Hearn, A. Onufriev, F. Pan, S. Pantano, R. Qi, A. Rahnamoun, D.R. Roe, A. Roitberg, C. Sagui, S. Schott-Verdugo, J. Shen, C.L. Simmerling, N.R. Skrynnikov, J.

Smith, J. Swails, R.C. Walker, J. Wang, H. Wei, R.M. Wolf, X. Wu, Y. Xue, D.M. York, S. Zhao, and P.A. Kollman. AMBER 2018, University of California, San Francisco. (University of California, San Francisco, 2018).
18. Ermak, D.L. & McCammon, J.A. Brownian dynamics with hydrodynamic interactions. *The Journal of Chemical Physics* **69**, 1352-1360 (1978).
19. Feller, S.E., Zhang, Y., Pastor, R.W. & Brooks, B.R. Constant pressure molecular dynamics simulation: The Langevin piston method. *The Journal of Chemical Physics* **103**, 4613-4621 (1995).
20. Martyna, G.J., Tobias, D.J. & Klein, M.L. Constant pressure molecular dynamics algorithms. *The Journal of Chemical Physics* **101**, 4177-4189 (1994).
21. Ryckaert, J.-P., Ciccotti, G. & Berendsen, H.J.C. Numerical integration of the cartesian equations of motion of a system with constraints: molecular dynamics of n-alkanes. *Journal of Computational Physics* **23**, 327-341 (1977).
22. Essmann, U. et al. A smooth particle mesh Ewald method. *The Journal of Chemical Physics* **103**, 8577-8593 (1995).
23. Bouysset, C. & Fiorucci, S. ProLIF: a library to encode molecular interactions as fingerprints. *Journal of Cheminformatics* **13**, 72 (2021).
24. Christopeit, T., Yang, K.-W., Yang, S.-K. & S. Leiros, H.-K. The structure of the metallo-*β*-lactamase VIM-2 in complex with a triazolylthioacetamide inhibitor. *Acta Crystallogr F Struct Biol Commun.* **72**, 813–819 (2016).
25. Kabsch, W. XDS. *Acta Crystallogr D Biol Crystallogr.* **66**, 125-132 (2010).
26. Kabsch, W. Automatic processing of rotation diffraction data from crystals of initially unknown symmetry and cell constants. *J. Appl. Cryst.* **26**, 795-800 (1993).
27. Kabsch, W. Integration, scaling, space-group assignment and post-refinement. *Acta Crystallogr D Biol Crystallogr.* **66**, 133-144 (2010).
28. Vonrhein, C. et al. Advances in automated data analysis and processing within *autoPROC*, combined with improved characterisation, mitigation and visualisation of the anisotropy of diffraction limits using *STARANISO*. *Acta Crystallogr. Sect. Found. Adv.* **74**, a360 (2018).
29. Vonrhein, C. et al. Data processing and analysis with the autoPROC toolbox. *Acta Crystallogr. Sect. D. Biol. Crystallogr.* **67**, 293–302 (2011).
30. McCoy, A.J. et al. Phaser crystallographic software. *J Appl Crystallogr.* **40**, 658-674 (2007).
31. Liebschner, D. et al. Macromolecular structure determination using X-rays, neutrons and electrons: recent developments in Phenix. *Acta Crystallogr D Struct Biol.* **75**, 861-877 (2019).
32. Emsley, P., Lohkamp, B., Scott, W. & Cowtan, K. Features and development of Coot. *Acta Crystallogr D Biol Crystallogr.* **66**, 486-501 (2010).
33. Laskowski, R.A. & Swindells, M.B. LigPlot+: multiple ligand-protein interaction diagrams for drug discovery. *J Chem Inf Model.* **51**, 2778-2786 (2011).
34. Zeldin, O.B., Gerstel, M. & Garman, E.F. *RADDOSE-3D*: time- and space-resolved modelling of dose in macromolecular crystallography. *J. Appl. Cryst.* **46**, 1225-1230 (2013).
35. Brem, J. et al. Structural basis of metallo-β-lactamase, serine-β-lactamase and penicillin-binding protein inhibition by cyclic boronates. *Nat Commun* **7**, 12406 (2016).
36. Daina, A., Michielin, O. & Zoete, V. SwissADME: a free web tool to evaluate pharmacokinetics, drug-likeness and medicinal chemistry friendliness of small molecules. *Sci. Rep.* **7:42717**(2017).

37. Ajay, G.W.B. & Murcko, M.A. Designing Libraries with CNS Activity. *J. Med. Chem.* **42**, 4942–4951 (1999).
38. Lobell, M., Molnár, L. & Keserű, G.M. Recent Advances in the Prediction of Blood–Brain Partitioning from Molecular Structure. *Journal of Pharmaceutical Sciences* **92**, 360-370 (2003).
39. Ma, X.-l., Chen, C. & Yang, J. Predictive model of blood-brain barrier penetration of organic compounds. *Acta Pharmacologica Sinica* **26**, 500-512 (2005).
40. Teo, Y.L., Ho, H.K. & Chan, A. Metabolism-related pharmacokinetic drug−drug interactions with tyrosine kinase inhibitors: current understanding, challenges and recommendations. *Br J Clin Pharmacol.* **79**, 241–253 (2015).
41. Zhao, Y.H. et al. Evaluation of human intestinal absorption data and subsequent derivation of a quantitative structure-activity relationship (QSAR) with the Abraham descriptors. *J. Pharm. Sci.* **90**, 749-784 (2001).
42. Yee, S. In Vitro Permeability Across Caco-2 Cells (Colonic) Can Predict In Vivo (Small Intestinal) Absorption in Man—Fact or Myth. *Pharm. Res.* **14**, 763–766 (1997).
43. Yamashita, S. et al. Optimized conditions for prediction of intestinal drug permeability using Caco-2 cells. *Eur. J. Pharm. Sci.* **10**, 195-204 (2000).
44. Irvine, J.D. et al. MDCK (Madin-Darby canine kidney) cells: A tool for membrane permeability screening. *J. Pharm. Sci.* **88**, 28-33 (1999).
45. Smith, D.A., Di, L. & Kerns, E.H. The effect of plasma protein binding on *in vivo* efficacy: misconceptions in drug discovery. *Nat. Rev. Drug Discov.* **9**, 929–939 (2010).
46. Liu, X., Wright, M. & Hop, C.E.C.A. Rational Use of Plasma Protein and Tissue Binding Data in Drug Design. *J. Med. Chem.* **57**, 8238–8248 (2014).
47. Lipinski, C.A., Lombardo, F., Dominy, B.W. & Feeney, P.J. Experimental and computational approaches to estimate solubility and permeability in drug discovery and development settings. *Adv. Drug. Deliv. Rev.* **23**, 3-25 (1997).
48. Ghose, A.K., Viswanadhan, V.N. & Wendoloski, J.J. A Knowledge-Based Approach in Designing Combinatorial or Medicinal Chemistry Libraries for Drug Discovery. 1. A Qualitative and Quantitative Characterization of Known Drug Databases. *J. Comb. Chem.* **1**, 55–68 (1999).
49. Muegge, I., Heald, S.L. & Brittelli, D. Simple Selection Criteria for Drug-like Chemical Matter. *J. Med. Chem.* **44**, 1841–1846 (2001).
50. Ertl, P., Rohde, B. & Selzer, P. Fast Calculation of Molecular Polar Surface Area as a Sum of Fragment-Based Contributions and Its Application to the Prediction of Drug Transport Properties. *J. Med. Chem.* **43**, 3714–3717 (2000).
51. Daina, A., Michielin, O. & Zoete, V. iLOGP: A Simple, Robust, and Efficient Description of n-Octanol/Water Partition Coefficient for Drug Design Using the GB/SA Approach. *J. Chem. Inf. Model.* **54**, 3284–3301 (2014).
52. Wildman, S.A. & M., C.G. Prediction of Physicochemical Parameters by Atomic Contributions. *J. Chem. Inf. Comput. Sci.* **39**, 868–873 (1999).
53. Moriguchi, I., Hirono, S., Nakagome, I. & Hirano, H. Comparison of Reliability of Log P Values for Drugs Calculated by Several Methods. *Chem. Pharm. Bull.* **42**, 976-978 (1994).
54. Moriguchi, I., Hirono, S., Liu, Q., Nakagome, I. & Matsushita, Y. Simple method of calculating octanol/water partition coefficient. *Chem. Pharm. Bull.* **40**, 127–130 (1992).
55. Delaney, J.S. ESOL: Estimating Aqueous Solubility Directly from Molecular Structure. *J. Chem. Inf. Comput. Sci.* **44**, 1000–1005 (2004).

56. Ali, J., Camilleri, P., Brown, M.B., Hutt, A.J. & Kirton, S.B. In Silico Prediction of Aqueous Solubility Using Simple QSPR Models: The Importance of Phenol and Phenol-like Moieties. *J. Chem. Inf. Model.* **52**, 2950–2957 (2012).
57. Hinchliffe, P. et al. Structural and Kinetic Studies of the Potent Inhibition of Metallo-β-lactamases by 6-Phosphonomethylpyridine-2-carboxylates. *Biochem.* **57**, 1880–1892 (2018).
58. Brem, J. et al. Structural Basis of Metallo-β-Lactamase Inhibition by Captopril Stereoisomers. *Antimicrob. Agents Chemother.* **60**, 142-150 (2015).
59. Li, N. et al. Simplified captopril analogues as NDM-1 inhibitors. *Bioorg. Med. Chem. Lett.* **24**, 386-389 (2014).
60. Yang, K.-W. et al. New β-phospholactam as a carbapenem transition state analog: Synthesis of a broad-spectrum inhibitor of metallo-b-lactamases. *Bioorg. Med. Chem. Lett.* **23**, 5855–5859 (2013).
61. Tehrani, K.H.M.E. et al. Small Molecule Carboxylates Inhibit Metallo-β-lactamases and Resensitize Carbapenem-Resistant Bacteria to Meropenem. *ACS Infect. Dis.* **6**, 1366–1371 (2020).
62. Skagseth, S. et al. Metallo-beta-lactamase inhibitors by bioisosteric replacement: Preparation, activity and binding. *Eur. J. Med. Chem.* **135**, 159-173 (2017).
